# Dysregulated lipid metabolism networks modulate T-cell function in people with relapsing-remitting multiple sclerosis

**DOI:** 10.1093/cei/uxae032

**Published:** 2024-04-16

**Authors:** Lucia Martin-Gutierrez, Kirsty E Waddington, Annalisa Maggio, Leda Coelewij, Alexandra E Oppong, Nina Yang, Marsilio Adriani, Petra Nytrova, Rachel Farrell, Inés Pineda-Torra, Elizabeth C Jury

**Affiliations:** Centre for Rheumatology, Division of Medicine, University College London, UK; Centre for Rheumatology, Division of Medicine, University College London, UK; Centre for Rheumatology, Division of Medicine, University College London, UK; Centre for Rheumatology, Division of Medicine, University College London, UK; Centre for Rheumatology, Division of Medicine, University College London, UK; Centre for Rheumatology, Division of Medicine, University College London, UK; Centre for Rheumatology, Division of Medicine, University College London, UK; Department of Neurology and Centre of Clinical, Neuroscience, First Faculty of Medicine, General University Hospital and First Faculty of Medicine, Charles University in Prague, Czech Republic; Department of Neuroinflammation, University College London and Institute of Neurology and National Hospital of Neurology and Neurosurgery, UK; Centre for Experimental & Translational Medicine, Division of Medicine, University College London, UK; Centre for Rheumatology, Division of Medicine, University College London, UK

**Keywords:** multiple sclerosis, CD4 + T cells, lipid metabolism, liver X receptor, lipid rafts

## Abstract

Altered cholesterol, oxysterol, sphingolipid, and fatty acid concentrations are reported in blood, cerebrospinal fluid, and brain tissue of people with relapsing-remitting multiple sclerosis (RRMS) and are linked to disease progression and treatment responses. CD4 + T cells are pathogenic in RRMS, and defective T-cell function could be mediated in part by liver X receptors (LXRs)—nuclear receptors that regulate lipid homeostasis and immunity. RNA-sequencing and pathway analysis identified that genes within the ‘lipid metabolism’ and ‘signalling of nuclear receptors’ pathways were dysregulated in CD4 + T cells isolated from RRMS patients compared with healthy donors. While *LXRB* and genes associated with cholesterol metabolism were upregulated, other T-cell LXR-target genes, including genes involved in cellular lipid uptake (inducible degrader of the LDL receptor, ID*OL*), and the rate-limiting enzyme for glycosphingolipid biosynthesis (UDP-glucosylceramide synthase, *UGCG*) were downregulated in T cells from patients with RRMS compared to healthy donors. Correspondingly, plasma membrane glycosphingolipids were reduced, and cholesterol levels increased in RRMS CD4 + T cells, an effect partially recapitulated in healthy T cells by *in vitro* culture with T-cell receptor stimulation in the presence of serum from RRMS patients. Notably, stimulation with LXR-agonist GW3965 normalized membrane cholesterol levels, and reduced proliferation and IL17 cytokine production in RRMS CD4 + T-cells. Thus, LXR-mediated lipid metabolism pathways were dysregulated in T cells from patients with RRMS and could contribute to RRMS pathogenesis. Therapies that modify lipid metabolism could help restore immune cell function.

## Teaser

Lipid metabolism was dysregulated in CD4 + T cells and serum from patients with relapsing-remitting multiple sclerosis compared to healthy controls, characterized by differential regulation of genes involved in cellular lipid uptake and lipid biosynthesis, altered T-cell plasma membrane lipid profiles and changes in serum lipoprotein expression. Alterations in T-cell plasma membrane lipids (lipid rafts) could be partially recapitulated in healthy T cells by *in vitro* culture with T-cell receptor stimulation in the presence of serum from RRMS patients. Conversely, co-stimulation of RRMS T cells with a liver X receptor (LXR)-agonist (a nuclear receptor important for lipid homeostasis), normalized membrane cholesterol levels, and reduced proliferation and IL17 cytokine production, suggesting that defects in LXR-mediated lipid metabolism pathways could contribute to RRMS pathogenesis.

## Graphical Abstract

**Graphical Abstract:**
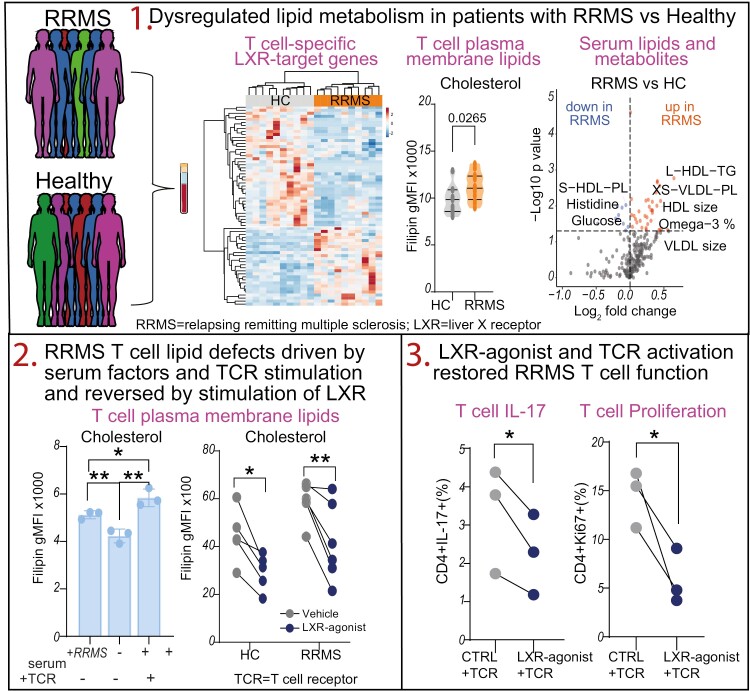

## Introduction

Multiple sclerosis (MS) is a chronic progressive neurological disease driven by both inflammatory and neurodegenerative processes. The disease affects approximately 2.5 million people worldwide [1] and while there is no cure, a number of disease modifying therapies targeting immune cell function are available [2]. Relapsing-remitting MS (RRMS) is characterized clinically by episodes of neurologic dysfunction followed by recovery, and both innate and adaptive immunity contribute to disease pathogenic mechanisms [3]. Peripheral blood autoreactive T-cells (CD4 + T helper (Th)17, Th1, and CD8+) specific for myelin cross the blood brain barrier (BBB) and cause inflammation in the brain. This process involves B-cells acting as antigen presenting cells [4], defects in immunoregulatory mechanisms [5], activation of central nervous system (CNS) resident cells, and the release of inflammatory mediators that exacerbate localized inflammation. Changes in lipid metabolic pathways are also common in RRMS. Altered cholesterol, oxysterol, sphingolipid, and fatty acid concentrations have been reported in the blood, cerebrospinal fluid (CSF), and brain tissue of people with MS. In addition, serum lipid profiles have been linked to disease progression and responses to treatment [6, 7].

Lipid metabolism can influence CD4 + T-cell function by regulating the lipid composition of plasma membranes [8, 9]. Plasma membrane lipids form a dynamic and heterogeneous environment, generating regions of relative stability (order) and fluidity [10, 11]. Regions enriched for glycosphingolipids and cholesterol (lipid rafts) regulate the lateral mobility of membrane proteins and the formation of signalling complexes [12]. Lipid rafts have been shown to influence a plethora of immune cell functions including T-cell and B-cell antigen receptor signalling, toll-like receptor signalling, antigen presentation, chemotaxis, and pathogen entry [13]. Moreover, plasma membrane lipid modification can rectify defective immune cell function in cancer and autoimmune disease [14, 15].

Our recent work identified a role for liver X receptors (LXRs) in modulating CD4 + T-cell function by regulating plasma membrane lipid raft content [8]. LXRs, LXRα (*NR1H3*), and LXRβ (*NR1H2*), are lipid-activated nuclear receptors that mediate lipid metabolism and cholesterol homeostasis [16]. Both isoforms are activated by oxidized derivatives of cholesterol (oxysterols) and intermediates of cholesterol biosynthesis and regulate genes involved in cholesterol efflux and uptake, fatty acid biosynthesis, phospholipid remodelling, and glycosphingolipid biosynthesis [8, 16]. LXRβ is the predominant LXR isotype in T-cells [8, 17] therefore genes regulated by the common LXR-synthetic ligand GW3965 (used extensively to study the function of LXRs) are expected to be predominantly regulated by LXRβ in CD4 + T-cells. Since LXRs have been shown to play a role in regulating human T-cell homeostasis and function [8, 17] it is possible that they could have therapeutic relevance to disorders characterized by defects in T-cell signalling and metabolism [18], including autoimmune diseases such as multiple sclerosis (MS). Here we explored lipid metabolism defects in CD4 + T-cells from patients with RRMS, with a focus on examining the role of LXRβ-regulated pathways on CD4 + T-cell lipid raft composition and function.

## Materials and methods

### Participant recruitment

Peripheral blood was collected from people diagnosed with relapsing-remitting multiple sclerosis (RRMS) according to the Revised McDonald Criteria 2010 [19] or from healthy controls (HCs) between January 2014 and January 2019 as part of the ABIRISK consortium [Anti-Biopharmaceutical Immunization Prediction and Clinical Relevance to Reduce the Risk; www.abirisk.eu] and RELOAD-MS (UCL) studies. All patients with RRMS were included in the study prior to their first disease modifying therapy. Peripheral blood mononuclear cells (PBMCs) were cryopreserved in liquid nitrogen until use. See Supplementary Table S1 for demographic information of participants included in each experiment.

### Cell sorting and isolation

CD4 + T-cells for RNA-sequencing analysis were sorted by fluorescence-activated cell sorting (FACS). Cells were washed in magnetic-assisted cell sorting (MACS) buffer (phosphate-buffered saline [PBS] with 2% foetal bovine serum [Labtech] and 1 mM ethylenediamine tetraacetic acid [Sigma]) before staining with fluorochrome-labelled antibodies: CD14-v450 (clone MφP9, BD Biosciences, Cat# 655114, RRID:AB_2870366), CD8a-FITC (clone RPA-T8, BD Biosciences Cat# 555366, RRID:AB_395769), CD19-APC-Cy7 (clone SJ25C1, Biolegend, Cat# 363010, RRID:AB_2564193), and CD4-BV605 (clone OKT4, Biolegend, Cat# 317435, RRID:AB_11149170) against surface markers for 30 min. Sorting was performed on a BD FACSAria II. CD4 + T-cells for cell culture/functional analysis were negatively isolated using magnetic bead-based separation according to manufactures instructions (EasySep, StemCell Technologies). Post-isolation purity was mean 90.93% (SD 2.75) assessed by flow cytometry for CD4 expression.

### RNA sequencing and analysis

Total RNA was extracted from CD4 + T-cells from RRMS patients (*n* = 10, mean age 42.2; 60% female, all Caucasian; prior to first treatment with interferon-beta therapy and HCs (*n* = 10, mean age 25.8; 50% female, all Caucasian) using TRIzol reagent (Life Technologies) followed by DNA-free DNA Removal Kit (Invitrogen). RNA integrity was confirmed using Agilent’s 2200 Tapestation. UCL Genomics (London) performed library preparation and sequencing.

#### Library preparation

Samples were processed using the SMART-Seq v4 Ultra Low Input RNA Kit (Clontech Laboratories, Inc.). Briefly, cDNA libraries were generated using the SMART (Switching Mechanism at 5ʹ End of RNA Template) technology. Eight cycles of PCR were used to generate cDNA, and integrity and quantity were checked on the Agilent Bioanalyser using the High Sensitivity DNA kit. A total of 200pg of cDNA was then converted to sequencing library using the Nextera XT DNA protocol (Illumina, San Diego, US). This uses a transposon able to fragment and tag double-stranded cDNA (tagmentation), followed by a limited PCR reaction (12 cycles) to add sample-specific indexes to allow multiplex sequencing.

#### Sequencing

Libraries to be multiplexed in the same run are pooled in equimolar quantities, calculated from Qubit and Bioanalyser fragment analysis. Samples were sequenced on the NextSeq 500 instrument (Illumina, San Diego, US) using a 43bp paired end run.

#### Data analysis

Run data were demultiplexed and converted to fastq files using Illumina’s bcl2fastq Conversion Software v2.19. Samples were grouped by disease status. To establish differences in gene expression between groups, sequence reads were aligned using STAR v2.5.0b to the human hg19 reference genome. Gene count abundance was quantified using Partek E/M Annotation Model with default settings in Partek’s RNA Flow software as in [8]. Differential expression analysis was performed using DESeq2 option in RNA Flow, which uses the Benjamin–Hochberg method for multiple testing correction (Supplementary Data File 1A–B). Pathway enrichment analysis was performed using Metascape [http://metascape.org] [20]. Pathways were considered enriched according to a significance threshold of *P* < 0.01, minimum overlap of three genes, and minimum enrichment score of 1.5 from KEGG, Reactome Gene Sets, Canonical pathways, and Corum structural complex repositories. Clustered heatmaps were generated in ClustVis [http://biit.cs.ut.ee/clustvis/], using correlation distance and average linkage for hierarchical clustering [21]. Volcano plots were created in R Studio (RStudio Team (2021). RStudio: Integrated Development Environment for R. RStudio, PBC, Boston, MA, http://www.rstudio.com/) using the EnhancedVolcano package (Blighe *et al*., 2022, https://github.com/kevinblighe/EnhancedVolcano).

### Immunophenotyping

Flow cytometry staining was performed as previously described [8, 22]. The following fluorochrome-labelled anti-human antibodies were used: CD3-BV785 (clone OKT3, Biolegend, Cat# 317329, RRID:AB_11219196), CD4-AF700 (clone OKT4, BioLegend Cat# 317425, RRID:AB_571942), CD8a-BV711 (clone RPA-T8, BioLegend Cat# 301043, RRID:AB_11218793), CD25-PE (clone M-A251, Biolegend, Cat# 985802, RRID:AB_2894588) CD127-PE-Cy7s (clone A019D5, Biolegend, Cat# 351319, RRID:AB_10899414). Plasma membrane lipids were detected using Cholera Toxin B subunit FITC conjugate (Sigma–Aldrich, Cat# C1655) for glycosphingolipids; filipin complex from *Streptomyces filipinensis* (Sigma–Aldrich, Cat# F9765) for cholesterol; and di-4-ANEPPDHQ (Thermo Fisher Scientific) for plasma membrane lipid order using methods described previously in references [10, 22]. Intracellular staining was performed using anti-human: IFNγ-Pacific Blue (clone 4S.B3, Thermo Fisher Scientific Cat# 57-7319-73, RRID:AB_494152), IL-4-APC (clone MP4-25D2, Biolegend Cat# 500812, RRID:AB_315131), IL-17A-AlexaFluor488 (clone eBio64Dec17, Thermo Fisher Scientific, Cat# 53-7179-73, RRID:AB_1633404), Ki67-PE (BD Biosciences, Cat# 556027, AB_2266296). Cytometer Setup and Tracking (BD Biosciences, Cat# 642412) beads were run to assess cytometer performance. Compensation was performed using anti-mouse IgGκ/negative control compensation particles set (BD Biosciences) or OneComp eBeads (Thermo Fisher Scientific, Cat# 01-1111-42), except for viability dyes and filipin which were performed with single stained and unstained cells. Samples were read on a BD LSR Fortessa X-20 cytometer using BD FACSDiva software. Data was analysed using FlowJo (Tree Star).

### Cell culture

Cells were cultured in 96 well plates (1 × 10^6^ cells/well) in complete media (RPMI 1640 medium (Thermo Fisher) supplemented with 10% foetal bovine serum (Thermo Fisher), Penicillin (100 IU/ml), and Streptomycin (100 μg/ml) (Gibco)). CD4 + T-cells treated with GW3965 (GW) (1 µM, Sigma-Aldrich, Cat# G6295) were compared to vehicle (dimethylsulfoxide, Sigma) for 24 h. Serum culture: negatively isolated CD4 + T-cells from a single healthy donor were cultured for 48 h ± 1 µg/ml plate-bound anti-CD3 (UCHT1, Thermo Fisher Scientific Cat# 16-0038-050, RRID:AB_2865589) and 1 µg/ml anti-CD28 (CD28.2, Thermo Fisher Scientific, Cat# 16-0289-025, RRID:AB_2865687) ± 10% serum from RRMS patients or HCs. Functional assays: negatively isolated CD4 + T-cells were stimulated with 1 µg/ml plate-bound anti-CD3 (UCHT1) and 1 µg/ml anti-CD28 (CD28.2) in solution for 72 h. To measure intracellular cytokine production cells were additionally stimulated with 50 ng/ml phorbol 12-myristate 13-acetate (Sigma–Aldrich, Cat# P1585) and 250 ng/ml ionomycin (Sigma–Aldrich, Cat# I3909) and GolgiPlug (BDBiosciences, Cat# 555029) for 5 h.

### Serum metabolomics

Measures of 228 serum biomarkers were acquired with an established NMR-spectroscopy platform (Nightingale Health) as we have described previously [23] (See https://research.nightingalehealth.com/uploads/documents/Nightingale-Blood-Analysis_List-of-Biomarkers.pdf). These included both absolute concentrations (mmol/l), ratios, and percentages (%) of lipoprotein composition. Serum lipids measured included apolipoproteins and very low, low, intermediate and high density lipoprotein (VLDL, LDL, IDL, and HDL) particles of different sizes ranging from chylomicrons and extremely large (XXL), very large, large (L), medium (M), small (S), and very small (XS). Lipids within each lipoprotein subclass included total lipid, phospholipids, total cholesterol, cholesterol esters (CE), free cholesterol (FC), and triglycerides. The distribution of lipids was expressed as a ratio or percentage (%) of total lipid content for each lipoprotein subclass.

### Statistical analysis

Statistical tests were performed in GraphPad Prism 8 (GraphPad Software, La Jolla, California, RRID:SCR_002798, https://www.graphpad.com/) unless otherwise stated. The D’Agostino–Pearson omnibus K2 test was used to check whether datasets were normally distributed. Unpaired two-tailed *t*-tests were used to compare between independent groups and are represented as bar charts (mean ± SE) or violin plots (median and interquartile range). In line with previous studies on LXR agonism in human cells [14, 24], paired two-tailed *t*-tests were used where cells from the same donor sample were exposed to different treatments (e.g. GW3965 versus control). This minimizes the impact of donor-to-donor heterogeneity at baseline. Where paired tests were applied, data are presented as paired line graphs.

## Results

### CD4 + T-cell activation, cytokine signalling, and lipid metabolism pathways are dysregulated between RRMS patients and healthy donors

**Figure 1.**
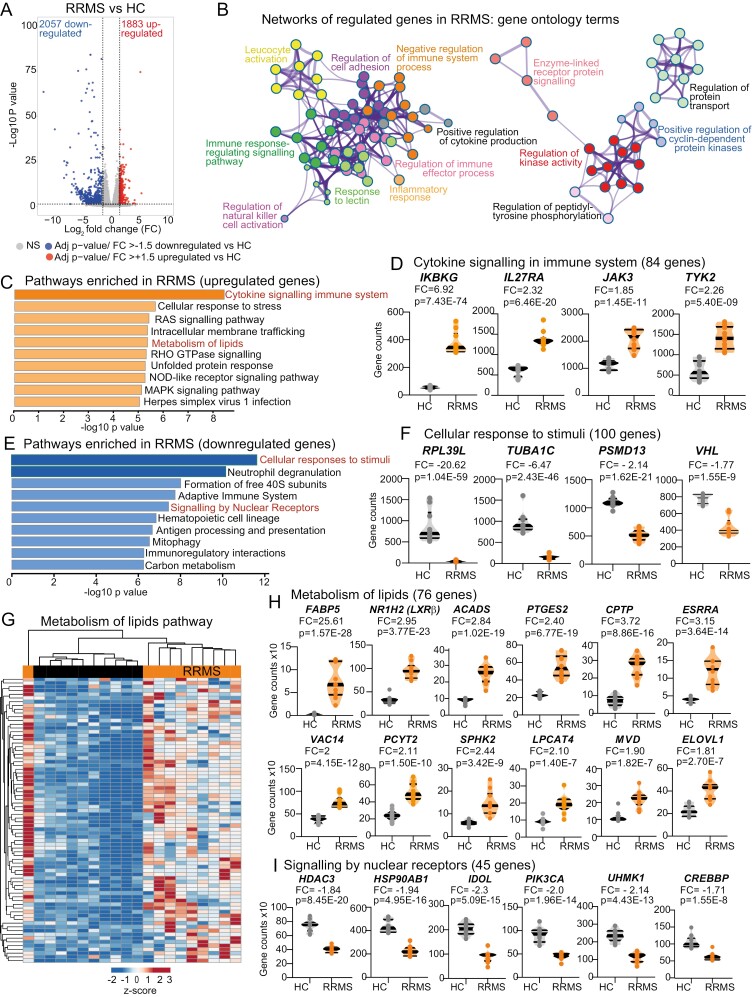
CD4 + T-cell activation, cytokine signalling, and lipid metabolism pathways are dysregulated between HCs and RRMS patients. FACS-sorted CD4 + T-cells from healthy controls (HC, *n* = 10) or RRMS patients (RRMS, *n* = 10) were analysed by RNA-sequencing to assess differentially expressed genes (DEGs). **(A)** Volcano plot showing Log_2_ fold change (> 1.5 or < -1.5) and FDR-adjusted *P*-value < 0.05. Significantly up- (right) and down- (left) regulated genes in RRMS compared to HCs. **(B–I)** Pathway enrichment analysis of DEGs analysed by Metascape [20] to identify regulated pathways. (B) Network diagram illustrates significantly regulated pathways enriched in up and down regulated genes combined using gene ontology terms. Each node represents a significantly enriched term, with node size proportional to the number of contributing genes. Annotations refer to gene ontology (GO) biological processes, similar terms were clustered, as labelled. (C) Bar chart of top 10 significantly enriched pathways upregulated in RRMS patients (Kegg and Reactome terms). Pathways are ranked by *P*-value and (D) violin plots of normalized RNAseq gene counts of the top four DEGs in the cytokine signalling pathway. (E). Bar chart of top 10 significantly enriched pathways downregulated in RRMS patients and (F) violin plots of normalized RNAseq gene counts for the top four DEGs in the ‘Cellular response to stimuli’ pathway. (G) Clustered heatmap and (H) violin plots of normalized gene counts showing top 10 DEGs in the ‘Metabolism of Lipids’ pathway from (C). (I) Violin plots of normalized gene counts showing top six DEGs in the ‘signalling by nuclear receptors’ pathway from (E). Violin plots show median and interquartile range, fold change and adjusted *P* value from RNA-seq analysis

CD4^+^ T-cell gene expression was substantially different in RRMS patients compared to healthy controls (HCs) (Supplementary Table S1 for patient and HC demographics and Supplementary Fig. S1A). RRMS patients were also correctly clustered from HCs when a 42-gene diagnostic classifier for MS (developed previously in PBMCs to identify MS patients from non-MS controls [25]) was applied (Supplementary Fig. S1B). In total, 3940 differentially expressed transcripts were identified with a roughly equal split of upregulated and downregulated genes (47.8% up and 52.2% down) (Fig. 1A and Supplementary Data File 1A and B). Significantly enriched gene expression pathways were hierarchically clustered into functionally related groups using gene ontology terms (Fig. 1B), although of the top 10 most significantly up- and downregulated genes only a small portion were protein coding (Supplementary Table S2).

Overall, upregulated genes were enriched in pathways involving cytokine and RAS signalling, response to stress, membrane trafficking, and lipid metabolism (Fig. 1C). CD4 + T-cells from RRMS patients had significantly increased expression of signalling kinases *JAK3, TYK2,* and *ZAP70,* transcription factors *IKBKG, GATA3,* and *Foxp3*, and cytokines/cytokine receptors (*TGFB, IL2RG, IL4R, IL21R,* and *IL27RA*), supporting an ongoing T-cell response [26] (Fig. 1D and Supplementary Fig. S2A). Downregulated pathways were predominantly related to cellular response to stimuli, neutrophil degranulation, ribosomal/RNA metabolism (formation of 40S subunits), adaptive immune response, and signalling of nuclear receptors (Fig. 1E and F, Supplementary Fig. S2B and C).

Of note, genes associated with lipid metabolism, were significantly dysregulated in CD4 + T-cells from RRMS patients compared with HCs including *NR1H2* (*LXRB*), which was significantly upregulated (Fig. 1G and H). However, despite the increased expression of *LXRB*, the downstream LXR-regulated pathway (Supplementary Fig. S2C) and LXR-target genes including the inducible degrader of the LDL receptor (ID*OL*) which regulates cholesterol uptake via degradation of the low-density lipoprotein receptor (*LDLR*) (Fig. 1I) were downregulated. LXR binding motifs were also identified amongst the genes downregulated in RRMS (Supplementary Table S3). Of interest, *RXRB* (Retinoid X Receptor β), a nuclear receptor regulated by retinoic acid which forms heterodimer with LXR, is also upregulated in RRMS patients (Supplementary Data File 1A). Thus, lipid metabolism, known to be associated with disease pathogenesis [6, 7] was dysregulated in CD4 + T-cells from patients with RRMS. Furthermore, the gene expression pathways enriched in this study, including lipid metabolism, cytokine signalling, signalling by nuclear receptors, and response to stimuli, were also identified in an independent gene expression dataset comparing CD4 + T-cells from RRMS patients with HCs, (Supplementary Fig. 3A-B) [27].

### LXR signalling is altered in CD4 + T-cell from RRMS patients

**Figure 2.**
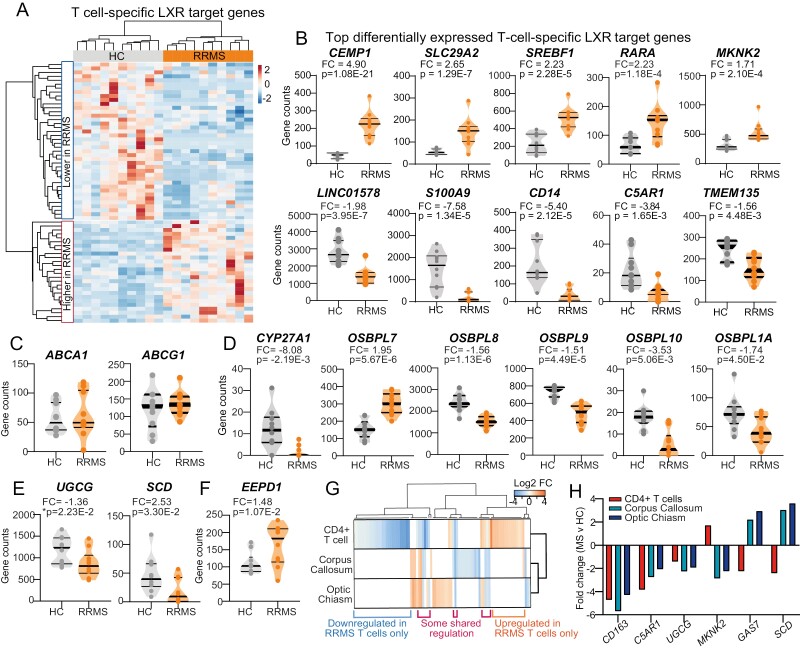
Lipid metabolism is dysregulated in patients with RRMS. CD4^+^ T-cells from HCs (*n* = 10) and people with RRMS (*n* = 10) were analysed by RNA-seq. (**A**) Heatmap showing unsupervised clustering of LXR-ligand responsive genes identified in [8] (key showing fold change) with (**B**) 10 most significantly different genes shown as violin plots. **(C)** Violin plots of known LXR-target genes ABCA1 and ABCG1; (**D**) genes related to the trafficking production and of endogenous LXR ligands (oxysterols); (**E and F**) genes involved in glycosphingolipid and cholesterol metabolism. Violin plots show median and interquartile range, fold change and adjusted *P* value from RNA-seq analysis. *Unadjusted *P* values. **(G)** Clustered heatmap to compare LXR-responsive genes found to be up or down regulated (key showing fold change) in RRMS compared to HCs in either human CD4 + T-cells (this paper) or brain tissue (corpus callosum and optic chiasm [28]). White space indicates that a gene was not significantly regulated in that tissue type. **(H)** Bar chart of the fold change for each tissue-type for a subset of genes regulated in all three datasets (CD4—human CD4 + T-cells, CC—corpus callosum, OC—optic chiasm)

Genes known to be regulated by LXR activation in healthy human CD4 + T-cells [8] were both up (*n* = 26) and down (*n* = 40) regulated in RRMS patients (Fig. 2A and B). Others remained unchanged, including the classic LXR target genes *ABCA1* and *ABCG1* (Fig. 2C), indicating a gene specific rather than a global change in LXR activity or that these targets were more sensitive to LXRβ activation [29] since LXRβ is the predominant isotype in T-cells [8, 17].

The most significantly upregulated LXR-target genes in RRMS CD4 + T-cells included other transcription factors, namely the nuclear receptor retinoic acid receptor alpha (*RARA*) and sterol response element binding factor 1 and 2 (*SREBF1/2*) (Fig. 2B, Supplementary Table S4). LXRs are ligand-activated transcription factors, responding to oxysterols and intermediates of cholesterol biosynthesis to regulate expression of its target genes [16]. Endogenous LXR ligands block entry of SREBF1 into the nucleus; therefore, reduced oxysterol availability could lead to upregulation of SREBF1 activity independent of induction by LXR. Gene expression of enzymes involved in oxysterol synthesis (*CYP27A1)* and transport (*OSBPL10, OSBPL1A, OSBPL7, OSBPL8*, and *OSBPL9)* were also dysregulated in RRMS, suggestive of a change in intracellular oxysterol availability and metabolism (Fig. 2D).

A number of the T-cell LXR-target genes that were reduced in RRMS are linked to cellular lipid metabolism including ID*OL* [30] (Fig. 1I), the rate-limiting enzyme for glycosphingolipid biosynthesis UDP-glucosylceramide synthase (*UGCG)* [8] and fatty acid desaturase *(SCD*) [31] (Fig. 2E). Other genes involved in cellular homeostasis of cholesterol (including *EEPD1* which positively regulates ABCA1-mediated cholesterol efflux) [32] (Fig. 2F and Supplementary Table S5), and glycosphingolipids (Supplementary Table S6), were also differentially expressed, supporting a global dysregulation of lipid metabolism in CD4 + T-cells from RRMS patients (Supplementary Fig. S3C). Notably, many of these DEGs have been associated previously with neurological disease including *SPHK2* (sphingosine kinase 2); *P2RX7* (purinergic receptor P2X 7); *SMPD4* (sphingomyelin phosphodiesterase 4); *ASAH1* (N-acylsphingosine amidohydrolase 1) (Supplementary Tables S5 and 6).

Finally, since genes controlling cholesterol metabolism are also dysregulated in the brains of people with MS [28], we cross-referenced the genes differentially expressed between HC and RRMS patients in six regions of the brain with the LXR-responsive genes identified in RRMS CD4 + T-cells. Differences in LXR-regulated genes occurred in two brain areas: the corpus callosum and optic chiasm (Fig. 2G). Although most genes did not share their expression pattern between these tissues and CD4 + T-cells, a subgroup of genes was dysregulated in all three, including the novel LXR target *UGCG* [8] (Fig. 2H), supporting that glycosphingolipid as well as cholesterol metabolism may be important in people with RRMS.

### Plasma membrane lipid metabolism is dysregulated in CD4 + T-cells from people with RRMS

**Figure 3.**
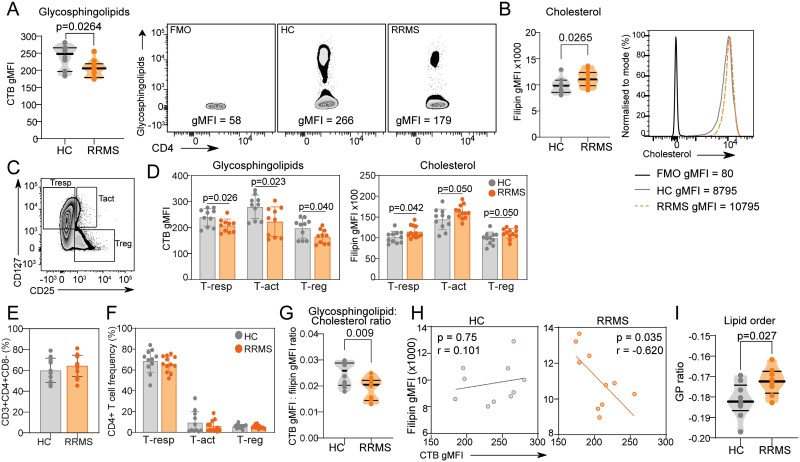
CD4 + T-cell plasma membrane lipids are dysregulated in patients with RRMS. Cells from healthy controls (HC; *n* = 10) or people with RRMS (*n* = 10) were stained with cholera toxin B (CTB) and filipin to measure CD4^+^ T cell expression of glycosphingolipid and cholesterol, respectively, in four independent experiments. Cumulative data shows CTB gMFI with representative flow cytometry plots (**A**), filipin gMFI with representative histogram (**B**). (**C**) CD4 + T-cells were defined by flow cytometry into regulatory (T-reg, CD4 + CD25 + CD127−), responder (T-resp, CD4 + CD25loCD127+) and activated (T-act, CD4 + CD25 + CD127+) subsets. (**D**) Bar graphs showing expression of glycosphingolipid and cholesterol levels across CD4 + T-cell subsets in HCs vs RRMS patients and CD4 + T-cell (**E**) or CD4 + T-cell subset (**F**) frequency between HCs and RRMS patients. (**G**) Violin plot showing the ratio of glycosphingolipid (CTB) to cholesterol (filipin) in HCs and RRMS patients. (**H**) Correlation between glycosphingolipid (CTB) and cholesterol (filipin) in HCs and RRMS patients, Pearson’s correlation. (**I**) Violin plot showing plasma membrane lipid order (GP ratio) assessed using di-4-ANEPPDHQ and measured by flow cytometry in cells. Mean ± SE, two-tailed *t*-test

Altered lipid metabolism gene expression was associated with changes in T-cell plasma membrane lipid content. Glycosphingolipid levels were lower and cholesterol levels were higher in CD4^+^ T-cells from RRMS patients compared with HCs (Fig. 3A and B) which was not related to the proportion of CD4^+^ T-cells or distribution of T-cell subsets (Fig. 3C and F). This resulted in an altered dynamic between plasma membrane lipids indicated by a decreased glycosphingolipid:cholesterol ratio, a significant negative correlation between glycosphingolipid and cholesterol levels in T-cells from RRMS patients and an increased membrane lipid order (a measure of membrane fluidity that influences immune cell signalling during activation [10, 11]) (Fig. 3G–I). Neither glycosphingolipid nor cholesterol levels correlated with clinical features including age, body mass index, vitamin D levels, expanded disability status score (EDSS), or number of relapses (all *P* > 0.23, Supplementary Table S7). Interestingly, changes in T-cell plasma membrane lipids were not seen in patients with secondary progressive MS (Supplementary Fig. S4A–E) potentially reflecting differences in pathogenic mechanisms.

### Changes in lipid metabolism in CD4 + T-cells in RRMS compared with healthy controls could be driven by T-cell activation and serum factors

**Figure 4.**
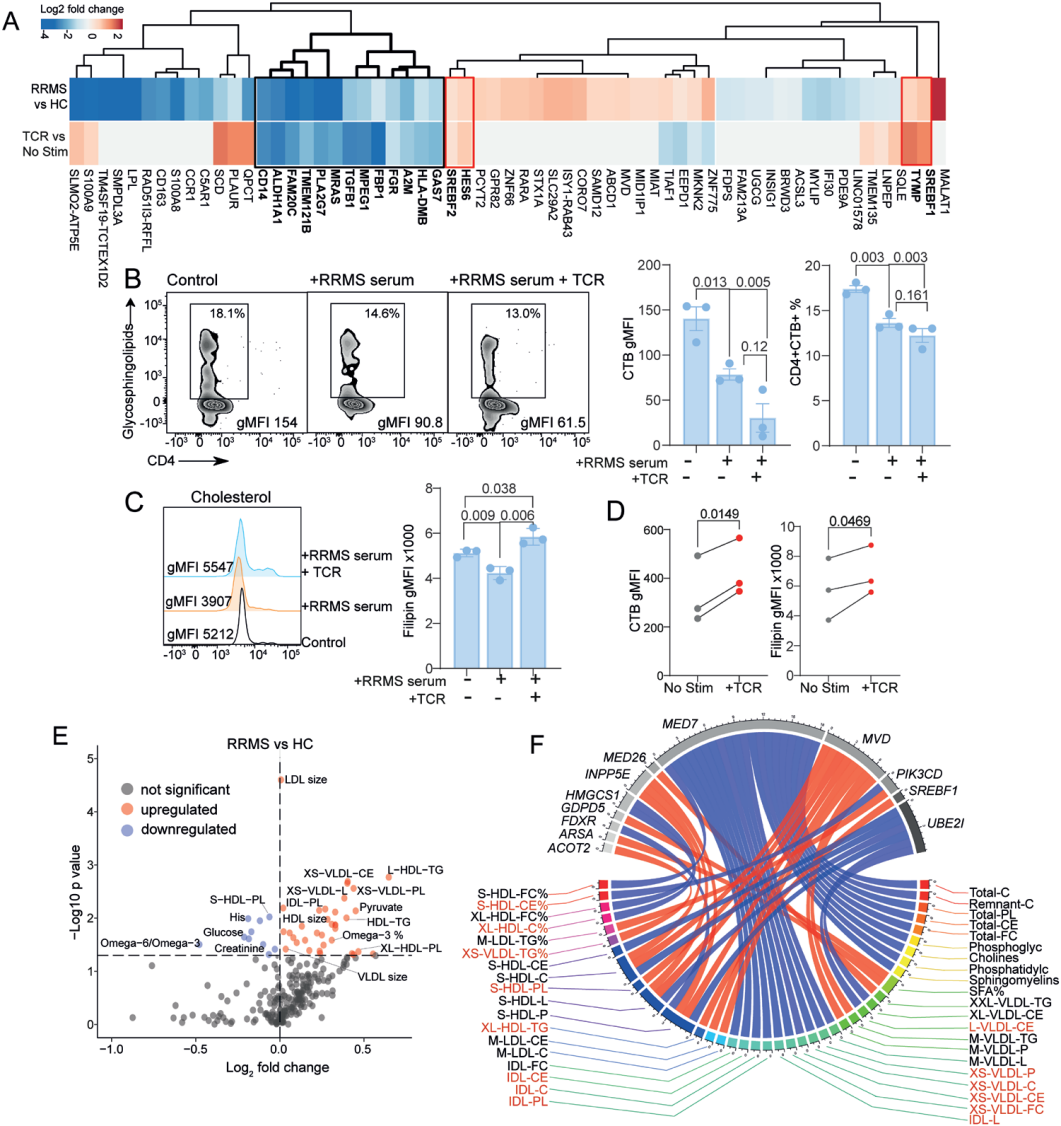
T-cell activation and elevated serum lipids could contribute to altered CD4 + T-cell lipid metabolism in RRMS patients. (**A**) Clustered heatmap to compare the fold changes of LXR-target genes differentially expressed in HC and MS CD4 + T-cells to the fold changes between activated and resting CD4 + T-cells (TCR v no stim) [8]. White space shows genes that were not significantly regulated by TCR stimulation. Genes commonly down-regulated (black box) or upregulated (red box) are indicated. **(B)** CD4 + T-cells from a single donor were negatively isolated using magnetic beads and cultured for 48 h ± RRMS serum (*n* = 3 different RRMS patients) ± TCR stimulation. HC serum was used as the control (*n* = 3). Glycosphingolipid (CTB) **(B)** and cholesterol (filipin) **(C)** levels were assessed by flow cytometry. Quantitative data and representative flow cytometry plots are shown. Paired and unpaired *t* tests. **(D)** HC CD4 + T-cells (*n* = 3) were stimulated ± TCR-stimulation for 48 h. Glycosphingolipid (CTB) and cholesterol (filipin) levels were assessed by flow cytometry. Paired *t* tests. **(E)** Serum metabolomics were assessed using a nuclear magnetic resonance platform in HC (*n* = 30) and RRMS patients (*n* = 20) from this study. Volcano plot showing Log_2_ fold change and −Log^10^*P*-value, horizontal dotted line = *P*-value 0.05. Unpaired *t* tests. Coloured points represent significantly up- (red) and down- (blue) regulated metabolites in RRMS compared to HCs. **(F)** Chord plot showing significant correlations between gene counts from DEGs in lipid metabolism pathway and metabolomic marker concentrations. Only significant associations (*P*-value < 0.001) with a Pearson coefficient > 0.6 are plotted; red lines = positive correlation; blue lines = negative correlations. Significantly differentially regulated metabolites identified in Fig. 4F labelled in red. For colour version of this figure refer to online version

We explored what mechanisms were driving altered CD4 + T-cell lipid metabolism in patients with RRMS. Overall, T-cell LXR target gene expression in RRMS did not resemble T-cell receptor (TCR) activation [8] since most genes were differently regulated in RRMS versus HCs compared to resting (no stim) versus TCR-activated (+ TCR) CD4 + T-cells from HCs (Fig. 4A). However, a cluster of genes were down-regulated in both RRMS and TCR-stimulated CD4 + T-cells, many of which were associated with immune cell activation and signalling (e.g. *HLA-DMB*, *A2M, FGR, TGFB1*, and *MRAS*) and sterol regulation. *SREBF1/SREBF2,* key transcription factors in fatty acid and cholesterol synthesis were upregulated in both datasets suggesting that CD4 + T-cell activation could contribute to some of the lipid metabolism abnormalities seen in patients with RRMS (Supplementary Table S8).

Next, we assessed the influence of serum, a potential source of cytokines and/or lipids known to influence immune cell lipid metabolism [33, 34]. CD4 + T-cells from a single healthy donor were cultured for 48 h with serum from either HCs or RRMS patients. Both plasma membrane glycosphingolipid and cholesterol levels were significantly reduced in the presence of serum from RRMS patients (Fig. 4B and C). The combination of T-cell stimulation in the presence of RRMS serum significantly increased cholesterol levels compared to controls but had no significant effect on glycosphingolipid levels. TCR-stimulation alone (using anti-CD3 and anti-CD28 antibody stimulation in the absence of serum) increased both glycosphingolipids and cholesterol (Fig. 4D). These data suggest that serum factors influence CD4 + T-cell lipid metabolism in RRMS patients. Surprisingly, serum cytokines from RRMS patients were not different from HCs in this study (Supplementary Fig. 5), however, serum lipid metabolites were significantly elevated in RRMS patients compared to HCs (Fig. 4E and Supplementary Table S9) and were significantly correlated with DEGs in the lipid metabolism pathway (Fig. 4F).

Thus, changes in cellular lipid metabolism could be driven by a combination of factors involving altered serum lipid metabolites and T-cell activation, leading to reduced CD4 + T-cell plasma membrane glycosphingolipid levels and increased cholesterol levels with potential effects on RRMS T-cell function.

### LXR agonist GW3965 reversed some abnormalities in CD4 + T-cells from RRMS patients

**Figure 5.**
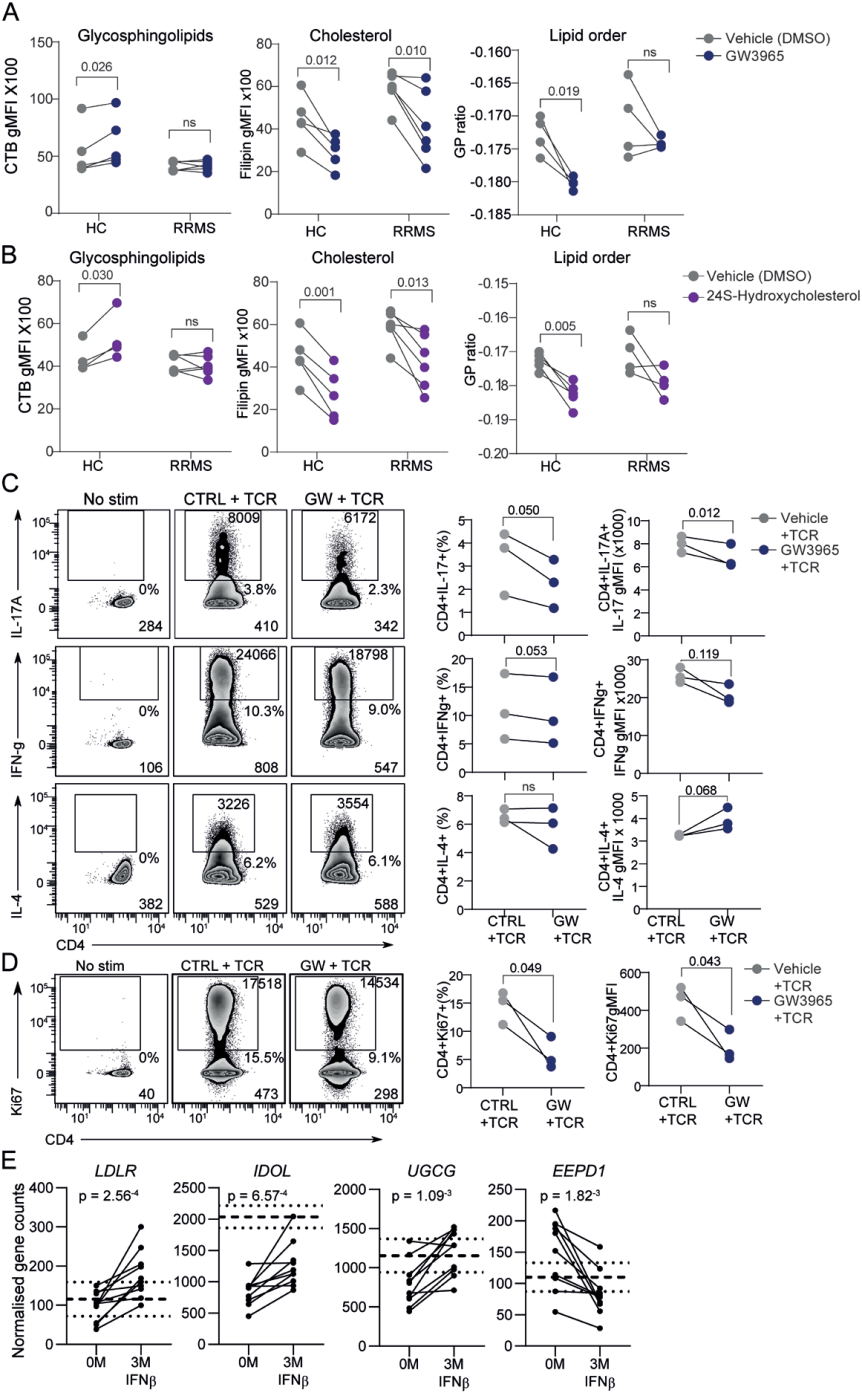
Activation of LXR via LXR agonist GW965 can reduce CD4 + T-cell activation in RRMS. **(A-B)** CD4 + T-cell membrane lipids, glycosphingolipids (CTB), cholesterol (filipin) (HCs *n* = 5 and RRMS *n* = 6) and lipid order (di-4-ANEPPDHQ) (HCs *n* = 4 and RRMS *n* = 4) were assessed after 24 h treatment with LXR-agonist GW3963 **(A**) or oxysterol 24S-Hydroxycholesterol **(B)** compared to DMSO vehicle. Paired *t* tests. **(C and D)** CD4 + T-cells were activated with anti-CD3/28 (+ TCR) with or without GW3965 (*n* = 3). **(C)** Cytokine production and **(D)** proliferation assessed by Ki67 expression were analysed by flow cytometry. Representative plots are labelled with the percentage of cytokine-producing/Ki67 + cells, and gMFI of the positive and whole population. Dot plots show the change in percentage and gMFI of populations. Paired *t* tests. **(E)** CD4 + T-cells were isolated from MS patients before (0 months; 0M) and after 3 months of treatment with beta interferons (3M + IFNβ) (*n* = 10). Graphs show normalized RNA-seq gene counts, dashed line represents the average expression and dotted lines the SD of each gene in HCs

Previously we found that T-cell plasma membrane lipids and T-cell function could be altered by stimulation with a LXR agonist (GW3695) [8]. Specifically, GW3965 increases plasma membrane glycosphingolipids and reduces cholesterol and lipid order in healthy CD4 + T-cells [8]. However, while GW3965 stimulation significantly reduced cholesterol levels in T-cells isolated from RRMS patients, it did not influence glycosphingolipid levels and had an inconsistent effect on membrane lipid order (Fig. 5A). This effect was recapitulated when T-cells were stimulated with the endogenous LXR-oxysterol agonist 24S-hydroxycholesterol (Fig. 5B). Notably, LXR activation did alter function in CD4 + T-cells from RRMS patients demonstrated by significantly reduced intracellular IL-17A expression, reduced proliferation (Ki67), and increased IL-4 production in (Fig. 5C and D), as we observed previously in HCs [8], although interferon-γ production was unaffected. This supports that LXR activation could ameliorate T-cell dysfunction in MS, as shown in mice [35]. The lipid metabolism defects identified in CD4 + T-cells isolated from RRMS patients are summarized in Supplementary Fig. S6.

Finally, comparing the expression of LXR-regulated genes before and after 3 months of treatment with the disease modifying drug IFNβ, identified changes in four lipid metabolism genes (*UGCG,* ID*OL, LDLR*, and *EEPD1*) which were normalized towards HC levels (dashed lines) (Fig. 5E), suggesting that correcting defects in lipid metabolism may contribute to the therapeutic effect of IFNβ on CD4 + T-cells in this context.

## Discussion

Here we describe changes in lipid metabolism in CD4 + T-cells from patients with RRMS compared to HCs, characterized by upregulation of genes associated with lipid metabolism pathways including the nuclear receptor *LXRB*; differential expression of T-cell LXR-target genes; and an altered T-cell plasma membrane lipid profile including increased glycosphingolipids and reduced cholesterol levels. Some of these lipid metabolism defects were driven by a combination of T-cell activation and dyslipidaemia and could be partially reversed by stimulation with the LXR-agonist GW3965 or one of its endogenous agonists, the oxysterol 24S-hydroxycholesterol. Therefore, this data supports a role for defects in lipid metabolism pathways in immune dysregulation in patients with RRMS.

Other studies have previously investigated differential gene expression in CD4 + T-cells from RRMS patients [36]. One study investigated the peripheral blood CD4 + T-cell transcriptome in RRMS patients during relapse vs HCs [27]. We used the data from this study to validate our results and identified multiple similarities including ‘metabolism of lipids’ and ‘signalling by nuclear receptors’ as well as pathways associated with regulation of transcription and immune cell activation and signalling. Another study did not observe differential gene expression profiles in CD4 + T-cells from untreated RRMS patients compared with HCs potentially reflecting RRMS patients recruited at different stages of their disease [37]. Recently, gene expression analysis in CD4 + T-cells in the blood and CSF from patients with RRMS and non-inflammatory conditions identified unique CD4 + T-cell gene expression profiles in the CSF [38]. This study identified activation and migration mechanisms in CD4 + T-cells, as well as cholesterol uptake and biosynthesis, suggesting that defects we identified in peripheral blood CD4 + cells from RRMS patients compared with HCs are maintained and most likely elevated still further in CD4 + T-cells in the CSF.

We aimed to focus on changes in lipid metabolism in CD4 + T-cells from patients with RRMS, which could drive dysregulated immune cell activation, differentiation, and function, and may contribute to MS pathogenesis and disease course [39]. Lipid metabolism plays a crucial role in orchestrating CD4 + T-cell differentiation into effector cells and we and others have shown that this is in part mediated by modulation of plasma membrane lipid composition [8, 10, 14, 15]. Plasma membrane cholesterol [40], glycosphingolipid [41], and fatty acid levels [42] are increased in activated T-cells. The pattern of CD4 + T-cell plasma membrane lipid expression was complex in RRMS patients characterised by reduced glycosphingolipids and increased cholesterol levels compared to HCs. Notably, LXRs are important transcriptional regulators of lipid homeostasis and immunity and we and others have demonstrated that LXR activation can modulate T-cell function by regulating plasma membrane lipid composition [8, 9]. In healthy human T-cells LXR activation increased plasma membrane glycosphingolipids reduced cholesterol levels and plasma membrane lipid order. This modulated immune synapse formation and proximal T-cell signalling in the context of TCR activation [8].

We proceeded to investigate whether the regulation of T-cell plasma membrane lipids by LXR was relevant in RRMS. We observed increased *LXRB* expression in RRMS T-cells in line with previous results showing LXRβ was increased in immune cells from people with MS [43] and upregulated LXR signalling in T-cells during the adoptive transfer model of experimental autoimmune encephalitis (EAE) [44]. LXR agonist treatment can ameliorate the severity of EAE, potentially by reducing infiltration of T-cells into the central nervous system [45] and inhibits IL-17 production [35]. Furthermore, our previous work in healthy human CD4 + T-cells shows that LXR could play a role in the function of regulatory T-cells by modulation of plasma membrane lipids [8]. Thus, further research is needed to understand the role of T-cell LXR signalling in MS in humans.

We demonstrate that LXR-responsive genes and lipids are dysregulated in circulating T-cells from people with RRMS. These effects were gene specific, suggesting the mechanism is more complex than a simple up or downregulation of LXR activity. Like other nuclear receptors, LXR function is orchestrated by a complex combination of factors including interactions with co-activator and co-repressor complexes, cross-talk with other nuclear receptors, changes in LXRE accessibility due to chromatin remodelling, availability of endogenous ligands, and post-translational modifications of receptor activity [46, 47]. Furthermore, the endogenous oxysterol ligands of LXR modulate the activity of other receptors and transcription factors including the SREBPs, oestrogen receptors, and retinoic acid receptor-related orphan receptors [48–50]. Consequently, it is probable that other lipid-regulated transcription factors influence the expression of these genes in this context. Additionally, different biological settings (such as T-cells and RRMS in this study) could reflect specific *LXRB* activation and result in differences in transcriptional regulation, as shown recently in a murine macrophage model overexpressing the LXR receptors [29]. In addition, our own previous results in the context of fatty liver show that disease and metabolic context alters target gene regulation by LXR in mice [51]. Thus, the specific role of LXR in the context of T-cell function and RRMS pathogenesis needs to be explored in more detail using experimental models.

Of the changes observed, it was notable that the expression of both *UGCG* and glycosphingolipids were significantly reduced in RRMS. Supporting this observation, *UGCG* was also reduced in CD4 + T-cells from people with clinically isolated syndrome compared to healthy donors (FC = −2.14, *P* = 2E−07 [52]). Moreover, UGCG mRNA is upregulated in T-cells in response to treatment with IFNβ and fingolimod [53] - another immunomodulatory drug used for the treatment of RRMS—strongly suggesting that therapeutic intervention modulates glycosphingolipid biosynthesis. Altered levels of ceramides and their downstream metabolites (hexosylceramides and lactosylceramide) have been reported in the serum, plasma, and immune cells from MS patients [54, 55]. Interestingly, Kurz *et al*. [55] reported elevated expression of *UGCG* in a whole white blood cell population (of which the majority are neutrophils), suggesting *UGCG* expression may be perturbed in cell type-specific manner. Indeed, glycosphingolipid synthesis was shown to drive pathogenic inflammatory processes in astrocytes in a murine model of secondary progressive MS, and administration of a UGCG inhibitor significantly reduced its progression [56]. This supports that glycosphingolipid metabolism is important in MS but may be beneficial or detrimental depending on the cell type, tissue, or disease stage.

Genes associated with fatty acid metabolism were also differentially expressed in T-cells from RRMS patients. Pro-inflammatory Th17 cells require fatty acid synthesis for plasma membrane phospholipid biosynthesis whereas regulatory T-cells do not, thus fatty acid metabolism influences proinflammatory T-cell lineage differentiation [57]. Fatty acid binding protein 5 (FABP5) was upregulated in T-cells from RRMS patients. FABPs are lipid chaperones that regulate fatty acid metabolism and inflammatory pathways [58]. In EAE, FABP5 deficiency resulted in reduced production of pro-inflammatory cytokines and reduced differentiation of Th17 and Th1 cells [59], an effect that was recapitulated using a FABP5 inhibitor, suggesting that FABP inhibition could be a novel therapeutic strategy in MS [60, 61]. Fatty acid metabolism can also influence regulatory T-cell (Treg) function. Defects in the ability of Tregs to control proinflammatory effector T-cell activation (e.g. Th1 and Th17 cells) have long been associated with RRMS pathogenesis [5]. A recent *in vitro* study showed that defective suppressive capacity in Tregs from RRMS patients could be restored by culture with oleic acid, a free fatty acid that supports Treg fatty acid β-oxidation-mediated oxidative phosphorylation metabolism and subsequent Treg differentiation and function. This effect was not observed when arachidonic acid was used and suggests that the lipid environment could influence Treg function [62]. LXR could play a role in Treg polarisation via increased expression of FoxP3 and increased Treg frequency in mice [63]. Notably LXR-deficient Tregs had reduced Treg function associated with defects in cholesterol metabolism in a murine knockout model [64]. However, our recent *in vitro* analysis of healthy CD4 + T-cells showed no difference in Foxp3 expression or IL10 production following TCR stimulation in the presence of GW3965, although CD4 + T-cell IL-2 production was increased which could support enhanced Treg function [8].

As expected, in addition to lipid metabolism, several pathways associated with the regulation and function of the immune system were identified, and as defined previously in other studies in peripheral blood from RRMS patients compared to HCs [5]. Among the top upregulated genes was *EEF1A1.* Acetylated EEF1A1 negatively regulates remyelination in the peripheral nervous system and CNS, whereas deacetylated EEF1A1 can promote remyelination [65], therefore, upregulation of EEF1A1 could disrupt the remyelination process in RRMS patients. Examples of other differentially regulated genes identified in this analysis and known to play a role in RRMS pathogenesis include tyrosine kinase 2 (*TYK2*); a polymorphism of *TYK2* (rs34536443 variant) is an established genetic risk factor for MS and T-cells expressing the protective *TYK2* (GC) genotype have decreased TYK2 activity associated with a TH2 cytokine profile compared with the MS-associated *TYK2* (GG) genotype [66]. IL-27 Receptor Subunit Alpha (IL27RA) plays a role in TH1 differentiation. IL-27 can dampen the severity of the disease in EAE mouse models and in RRMS patients IL-27 and soluble IL-27Rα are elevated and could reduce the effect of IL-27 on immune cell function [67]. Notable down-regulated genes include the *VHL* (Von Hippel-Lindau Tumor Suppressor) gene involved in the ubiquitination and degradation of hypoxia-inducible-factor (HIF), a transcription factor with a central role in many cellular processes including cytokine signalling. Reduced VHL could contribute to increased HIF activity in T-cells and support Th17 differentiation [68]. One study identified VHL deficiency in patients with MS [69] and another reported a role for VHL in the remyelination process [70]. Notably, the VHL gene is known to be the primary regulator of Nrf2, the target of dimethyl fumarate, an established therapy for patients with RRMS [71]. It is also likely that some of the above-mentioned processes are influenced by dysregulated plasma membrane lipid profiles via downstream intracellular signalling [10, 11].

While this study shows that CD4 + T-cell lipid metabolism and immune pathways could be disrupted in patients with RRMS, there are some limitations. All the patients were white (therefore the effect of race was not explored), there was no data available assessing BMI of healthy donors and pre- and post-sorted CD4 + T-cells were not assessed for potential loss of small subset populations such as CD56 + CD4 + T-cells, known to be dysregulated in some patients with MS, that could potentially influence the data [72]. In addition, there were differences between the age of patients and controls for the RNA-sequencing experiment. Age is known to influence lipid metabolism in health and disease (reviewed in detail in [73–75]). Of relevance to the work presented in this study, it has been suggested that decreased plasma membrane fluidity (influenced by the lipid composition of plasma membranes) may be associated with ageing [74]. This is supported by observations that plasma lipids including triglycerides and cholesterol alter over age (humans and mice) and levels of membrane phospholipid/fatty acid unsaturation and consequently membrane fluidity are decreased with age in rodent brain/liver/heart. Interestingly, longevity is associated with increased levels of unsaturated fatty acids, which helps to maintain membrane fluidity. For example, lymphocytes from centenarian donors have elevated membrane polyunsaturated fatty acids (associated with increased fluidity and decreased susceptibility to lipid peroxidation) compared with younger age groups; and dietary restriction, which protects from age-associated reduction in membrane fluidity, and promotes longevity in mice. Also relevant to this study, activation of LXR using GW3965 stimulated macrophage cholesterol/lipid metabolism in aged mice and delayed age-related nerve damage [76]. While most research examining the relationship between age and lipid metabolism is performed in experimental models (yeast, worms, and rodents) some studies have been performed comparing lipid metabolism between older human donors (> 90 years) and younger groups. These studies have shown increased sphingomyelin levels with age in females, and changes in plasma cholesterol levels with age including changes in HDL composition (reduced cholesterol and increased sphingomyelin) which could affect HDL antioxidant and cholesterol transport function in older adults. Of note, reduced plasma HDL levels are associated with cognitive dysfunction in adults > 100 years [75]. Thus, while changes in lipid metabolism are evident when comparing older adults (> 90 years old) with younger age groups, it is less clear how age influences lipid metabolism longitudinally in younger adults. Therefore, while we cannot rule out an effect of age on the RNA-sequencing data presented in this study, it is likely not the sole contributor to the RRMS phenotype identified and MS pathology likely plays an important role. Our findings were validated using another CD4 + T-cell RNA-sequencing dataset [27] and support previous results from multiple sources showing that lipid metabolism is dysregulated in patients with MS or EAE [35, 43, 44, 52, 59]. Furthermore, in a recent study investigating lipid-focused metabolites in patients with RRMS (median age 34, range 19–59) compared with older secondary progressive MS patients (median age 54, range 35–78), while age was identified in the machine learning models used for classification, lipid metabolites were also key important features for patient stratification [77]. It will be important for future studies to further validate the RNA-sequencing findings and investigate the specific role of LXR and other nuclear receptors in controlling lipid metabolism pathways and how this influences the balance between regulatory and effector T-cell function in the context of MS. In addition, further studies are needed to assess whether the normalization of LXR-regulated gene expression by IFNβ is accompanied by a restoration of CD4 + T-cell membrane lipid expression and T-cell function.

In conclusion, lipid metabolism is likely to play a crucial role in the pathogenic immune processes driving MS and is a potential treatment target. Indeed, statins (widely used therapy for lowering serum lipids) have been extensively studied in MS [78] and can promote Th2 differentiation and inhibit Th1-mediated damage. Simvastatin also inhibits secretion of cytokines necessary for Th1 and Th17 differentiation in RRMS patients by inhibiting the interferon regulatory factor-4 transcription factor [79]. As well as direct inhibition of cholesterol biosynthesis, statins also influence immune activation by altering the membrane association of certain signalling proteins via inhibition of mevalonate pathway-derived isoprenoids [80]. Thus, our findings support the increased interest in lipid metabolites in RRMS pathology [81] and highlight the complex interplay between lipid metabolism and immune cell signalling and function at both cellular and systemic levels. A more detailed understanding of the alterations to lipid metabolism in both peripheral blood and the CNS in patients affected by MS is needed.

## Supplementary Data

Supplementary data is available at *Clinical and Experimental Immunology* online.

uxae032_suppl_Supplementary_Data_S1

uxae032_suppl_Supplementary_Data_S2

uxae032_suppl_Supplementary_Materials

## Data Availability

The data underlying this article will be shared on reasonable request to the corresponding author. Publicly available datasets were also analysed in this study. This data can be found here: Gene Ex-pression Omnibus (GEO); (http://www.ncbi.nlm.nih.gov/geo/) repository [accession number: GSE172009] [27] and https://www.ncbi.nlm.nih.gov/geo [accession nos. GSE123496] (women with multiple sclerosis and age matched healthy controls: hippocam-pus, frontal cortex, internal capsule, corpus callosum, and parietal cortex), GSE100297(MS and control: optic chiasm) [28].

## Abbreviations:

BBB: blood brain barrier
CE: cholesterol esters
CNS: central nervous system
CSF: cerebrospinal fluid
CTB: cholera toxin B
DEGs: differentially expressed genes
EAE: experimental autoimmune encephalitis
EDSS: expanded disability status score
FABP: fatty acid binding protein
FC: free cholesterol
GO: gene ontology
HC: healthy controls
HDL: high density lipoprotein
IDL: intermediate density lipoprotein
*IDOL*: inducible degrader of the LDL receptor
L: large
LDL: low density lipoprotein
*LDLR*: low-density lipoprotein receptor
LXR: liver X receptors
M: medium
MS: multiple sclerosis
PBMCs: peripheral blood mononuclear cells
RRMS: relapsing-remitting MS
*RXRB*: Retinoid X Receptor β
S: small
*SREBF1/2*: sterol response element binding factor 1 and 2
TCR: T-cell receptor
Th: T helper
*UGCG*: UDP-glucosylceramide synthase
VLDL: very low density lipoprotein
XL: very large
XS: very small
XXL: chylomicrons and extremely large
