# Supplementary material for: Dysregulated lipid metabolism networks modulate T-cell function in people with relapsing-remitting multiple sclerosis
Source: Clin Exp Immunol. 2024 Apr 16;217(2):204–18. doi: 10.1093/cei/uxae032 (PMC11239565; doi:10.1093/cei/uxae032)
Supplement: uxae032_suppl_Supplementary_Data_S1 [file uxae032_suppl_supplementary_data_s1.pdf]

RefSeq	Total counts	P-value (RRMS vs. HEALTHY)	FDR step up (RRMS vs. HEALTHY)	Fold change (RRMS vs. HEALTHY)
SMPD4	8166	3.95E-05	3.17E-04	1.50
FAIM	1558	4.82E-03	1.94E-02	1.50
SMYD4	7511	5.32E-03	2.10E-02	1.50
COX19	16613	7.96E-08	1.23E-06	1.50
HMGCS1	2981	8.87E-03	3.20E-02	1.50
WDR55	7191	6.08E-03	2.35E-02	1.50
NAGLU	3236	7.51E-03	2.79E-02	1.50
ASB6	8471	7.49E-05	5.57E-04	1.50
MTG2	2819	1.14E-03	5.81E-03	1.50
RBM10	6295	3.47E-06	3.71E-05	1.50
PRMT7	7954	8.00E-06	7.82E-05	1.50
ZDHHC12	4721	2.68E-04	1.68E-03	1.50
IKBKB	12707	7.63E-04	4.12E-03	1.50
TP53BP1	9806	9.02E-05	6.52E-04	1.50
CARHSP1	4346	4.03E-05	3.22E-04	1.51
NFYC-AS1	2451	9.54E-03	3.40E-02	1.51
APOBEC3D	1889	7.81E-03	2.88E-02	1.51
PUSL1	1792	1.76E-03	8.36E-03	1.51
EXD3	1977	2.30E-03	1.04E-02	1.51
CENPBD1P1	3366	1.03E-03	5.33E-03	1.51
LMBR1L	11520	1.94E-03	9.10E-03	1.51
GDF11	1368	8.46E-03	3.07E-02	1.51
RNF126	12828	4.12E-04	2.42E-03	1.51
NRBP2	5212	3.30E-03	1.42E-02	1.51
HSPB1	4847	1.39E-02	4.61E-02	1.51
BLOC1S4	16460	3.98E-05	3.19E-04	1.51
EMP3	51277	1.91E-03	8.95E-03	1.51
PANK4	6901	1.89E-04	1.24E-03	1.51
RAB24	7890	1.13E-04	7.91E-04	1.51
MOV10	14193	8.73E-04	4.62E-03	1.51
ZNF691	1558	1.02E-02	3.57E-02	1.51
ASB16-AS1	4440	1.34E-05	1.23E-04	1.51
N6AMT1	2937	2.76E-04	1.72E-03	1.51
MGAT1	16284	1.09E-03	5.58E-03	1.51
MED25	2136	8.34E-03	3.04E-02	1.51
LINC00861	81751	1.81E-04	1.20E-03	1.51
PEX10	954	3.14E-03	1.36E-02	1.51
DNAJC19	12558	3.72E-04	2.22E-03	1.51
OPA3	2534	4.43E-04	2.57E-03	1.51
VARS2	2090	7.49E-03	2.79E-02	1.51
IL2RG	132634	2.83E-06	3.08E-05	1.51
TCEAL1	2449	4.65E-03	1.88E-02	1.51
MON1A	1619	6.05E-03	2.34E-02	1.51

PACS2	4449	9.31E-03	3.33E-02	1.52
RPSAP58	40414	2.05E-04	1.33E-03	1.52
LOC101927954	1466	1.26E-02	4.26E-02	1.52
PRR29	2476	9.28E-04	4.86E-03	1.52
PPP1R35	9415	1.04E-04	7.38E-04	1.52
ZNF562	2993	7.98E-04	4.28E-03	1.52
PTPN7	14482	1.64E-05	1.47E-04	1.52
SSNA1	8815	6.68E-05	5.04E-04	1.52
HSD17B12	7874	4.48E-03	1.82E-02	1.52
JRKL	2441	4.94E-03	1.98E-02	1.52
RTL10	3472	4.39E-04	2.55E-03	1.52
SFR1	2977	1.23E-03	6.15E-03	1.52
LOC401261	5407	1.14E-02	3.93E-02	1.52
AKAP17A	32965	1.44E-03	7.03E-03	1.52
MLST8	6421	5.33E-04	3.01E-03	1.52
FKBP14	1397	1.41E-02	4.67E-02	1.52
CDK20	1386	8.07E-03	2.96E-02	1.52
HNRNPA1	53958	1.32E-06	1.57E-05	1.52
VAMP4	19554	3.85E-05	3.10E-04	1.52
C1QTNF6	1227	5.56E-04	3.12E-03	1.52
TMEM223	5346	2.09E-04	1.35E-03	1.52
SLC25A45	9680	1.62E-05	1.46E-04	1.52
ROGDI	2005	1.52E-03	7.38E-03	1.52
CYTH2	8168	2.71E-06	2.97E-05	1.52
GGPS1	12346	8.51E-06	8.27E-05	1.52
ZNF382	2462	5.72E-03	2.23E-02	1.52
JMJD8	14731	3.23E-05	2.66E-04	1.52
SUCLA2	4173	2.48E-05	2.12E-04	1.52
HYOU1	4136	8.68E-03	3.15E-02	1.52
AK6	6305	6.82E-04	3.74E-03	1.52
OTUB1	34645	8.69E-09	1.63E-07	1.52
LOC100132057	1382	1.00E-02	3.53E-02	1.52
DTNB	7160	2.95E-04	1.81E-03	1.52
NCKIPSD	2403	2.65E-04	1.66E-03	1.53
LOC100506127	1001	6.95E-03	2.62E-02	1.53
RPTOR	4486	2.60E-04	1.63E-03	1.53
WAS	3788	6.18E-04	3.42E-03	1.53
IL21R	10544	9.52E-06	9.11E-05	1.53
RRN3P3	3137	8.50E-04	4.51E-03	1.53
DBT	4992	5.88E-04	3.27E-03	1.53
TRIM68	2778	1.23E-02	4.18E-02	1.53
USP36	17980	1.64E-05	1.47E-04	1.53
GTF2H4	1258	2.96E-03	1.30E-02	1.53
WDR92	2928	1.52E-03	7.38E-03	1.53
TUT1	5924	2.81E-04	1.74E-03	1.53
SDR39U1	24268	1.51E-06	1.77E-05	1.53

C6orf203	3141	7.79E-04	4.20E-03	1.53
SIGIRR	23374	4.53E-06	4.71E-05	1.53
FGFBP3	1444	2.78E-03	1.23E-02	1.53
RNH1	21025	2.24E-06	2.51E-05	1.53
ITPK1	3115	3.01E-03	1.31E-02	1.53
CDK5RAP3	26857	4.54E-07	6.02E-06	1.53
DNM2	19263	2.18E-03	1.00E-02	1.53
UBE2I	18235	7.22E-05	5.38E-04	1.53
MAST3	8783	1.78E-06	2.05E-05	1.53
ZDHHC18	2991	8.64E-05	6.28E-04	1.53
TMEM140	2576	1.43E-02	4.71E-02	1.53
TRNT1	6451	6.86E-04	3.76E-03	1.53
HMG20B	6683	9.21E-06	8.84E-05	1.53
RAB40B	867	6.41E-03	2.45E-02	1.53
PARD6B	2215	1.83E-03	8.66E-03	1.53
MOB3C	2324	3.30E-03	1.42E-02	1.54
DDX39B	13259	4.63E-09	9.17E-08	1.54
BCL9L	6280	1.62E-06	1.89E-05	1.54
SGTA	6785	9.02E-05	6.52E-04	1.54
MCRS1	8453	1.25E-07	1.86E-06	1.54
LINC00402	12871	5.53E-03	2.17E-02	1.54
RNF166	24588	1.67E-05	1.50E-04	1.54
HELB	14989	1.52E-04	1.03E-03	1.54
LOC100287896	1861	1.07E-02	3.74E-02	1.54
LAMP1	6548	7.79E-04	4.20E-03	1.54
CCDC107	7897	4.77E-04	2.73E-03	1.54
TMEM104	2387	9.04E-04	4.76E-03	1.54
EIF2S2	10038	3.36E-04	2.03E-03	1.54
C19orf66	19547	1.95E-05	1.72E-04	1.54
MAPKBP1	1889	5.71E-03	2.23E-02	1.54
ZNF391	1338	8.97E-03	3.23E-02	1.54
MSTO1	9618	5.94E-06	6.01E-05	1.54
ZNF524	2268	2.93E-04	1.81E-03	1.54
MXD4	14813	1.93E-05	1.70E-04	1.54
USP11	15256	3.27E-05	2.69E-04	1.54
SLX1A	1769	1.61E-03	7.73E-03	1.54
SLX1B	1769	1.61E-03	7.73E-03	1.54
TUBGCP4	3579	4.08E-03	1.69E-02	1.54
ARHGEF19	2071	1.26E-03	6.28E-03	1.55
AMPD2	10275	7.31E-04	3.97E-03	1.55
LINC00954	3125	8.21E-03	3.00E-02	1.55
C17orf67	1602	5.38E-03	2.13E-02	1.55
VASP	5748	2.73E-05	2.30E-04	1.55
RPL13	187018	2.18E-04	1.40E-03	1.55
SPDYE1	1055	6.08E-03	2.35E-02	1.55
P4HTM	12712	1.16E-06	1.39E-05	1.55

ZNF813	2455	4.18E-03	1.73E-02	1.55
DYNLT3	17832	1.87E-06	2.15E-05	1.55
TMEM30B	2973	6.11E-03	2.36E-02	1.55
CLN3	10325	3.16E-06	3.39E-05	1.55
PKM	52998	7.36E-05	5.48E-04	1.55
CD84	21695	7.30E-04	3.97E-03	1.55
LINC00869	9185	2.22E-05	1.93E-04	1.55
HM13	15021	1.58E-07	2.29E-06	1.55
TMSB10	349418	1.15E-03	5.84E-03	1.55
NXF1	32318	3.82E-06	4.05E-05	1.55
GIMAP7	121571	1.26E-03	6.28E-03	1.55
ITPKC	866	1.41E-02	4.66E-02	1.55
LOC100133091	1549	2.48E-03	1.12E-02	1.55
KLHDC7B	1688	4.74E-03	1.91E-02	1.55
MAP3K3	11907	3.00E-04	1.84E-03	1.55
TYSND1	6384	7.04E-05	5.28E-04	1.55
GTPBP6	6400	9.15E-06	8.79E-05	1.55
NAT9	6706	1.96E-03	9.16E-03	1.55
KDF1	591	1.26E-02	4.26E-02	1.56
DNASE1L1	2282	7.52E-03	2.79E-02	1.56
SREBF2	8070	2.82E-03	1.24E-02	1.56
RINL	6632	2.77E-04	1.72E-03	1.56
TMEM120B	4163	2.26E-05	1.97E-04	1.56
NUAK2	7288	1.87E-03	8.82E-03	1.56
SLC12A9	2594	1.81E-04	1.20E-03	1.56
BAZ1B	18281	5.97E-03	2.31E-02	1.56
CEP72	1472	4.30E-03	1.76E-02	1.56
SHISA5	55583	1.30E-06	1.55E-05	1.56
XAF1	27365	1.06E-04	7.49E-04	1.56
PTMA	144277	5.20E-04	2.95E-03	1.56
GZMM	5225	1.73E-03	8.23E-03	1.56
SLC39A13	9687	1.12E-05	1.05E-04	1.56
GID4	1469	1.18E-02	4.05E-02	1.56
HTRA2	5478	1.48E-07	2.16E-06	1.56
YDJC	7024	4.48E-05	3.54E-04	1.56
AKTIP	19467	5.09E-06	5.22E-05	1.56
DOT1L	1848	2.82E-03	1.25E-02	1.56
ZNF678	4041	1.09E-02	3.78E-02	1.56
ZNF564	9206	6.01E-04	3.34E-03	1.56
GIMAP4	73189	1.27E-04	8.78E-04	1.57
MED24	7920	1.53E-06	1.79E-05	1.57
ELF4	4150	2.26E-03	1.03E-02	1.57
GEMIN6	1219	8.48E-03	3.08E-02	1.57
TMEM41B	7429	3.92E-06	4.15E-05	1.57
LAT	59114	4.67E-11	1.33E-09	1.57
RALGPS1	2123	1.80E-03	8.54E-03	1.57

ZNF76	10728	3.26E-04	1.98E-03	1.57
UBXN11	8101	8.13E-06	7.94E-05	1.57
NLRC3	20585	4.39E-05	3.48E-04	1.57
PIGV	1832	5.09E-03	2.03E-02	1.57
RRP9	3140	6.42E-05	4.86E-04	1.57
TWF2	11097	1.18E-08	2.17E-07	1.57
IP6K1	6004	1.03E-03	5.31E-03	1.57
PLCB2	16851	4.50E-04	2.61E-03	1.57
PSTPIP1	13580	5.75E-07	7.42E-06	1.57
STARD3	8441	4.40E-07	5.86E-06	1.57
FAM86DP	981	8.18E-03	3.00E-02	1.57
CEACAM21	4902	4.82E-06	4.98E-05	1.57
DHX30	13506	5.33E-06	5.43E-05	1.57
CTDNEP1	3499	1.57E-04	1.05E-03	1.57
AMT	2771	2.27E-03	1.03E-02	1.57
MYO1G	28071	6.24E-05	4.75E-04	1.57
FGD3	44319	8.42E-08	1.30E-06	1.57
SARM1	2854	8.20E-04	4.37E-03	1.57
SHARPIN	9259	3.64E-08	5.99E-07	1.57
TMEM173	26094	2.59E-06	2.86E-05	1.57
ACADVL	31017	1.91E-05	1.69E-04	1.57
RPL13AP20	14731	1.54E-04	1.04E-03	1.57
AP5S1	2224	4.12E-04	2.42E-03	1.58
IQSEC1	26099	1.01E-08	1.87E-07	1.58
PLA2G12A	7251	6.49E-05	4.91E-04	1.58
TLDC1	1948	2.50E-03	1.12E-02	1.58
KIF1BP	3037	2.02E-04	1.32E-03	1.58
ATG9B	4318	8.91E-06	8.59E-05	1.58
TRMU	6720	3.83E-09	7.73E-08	1.58
ZNF550	8127	1.36E-03	6.68E-03	1.58
TMEM143	3304	7.42E-05	5.52E-04	1.58
EXOSC6	6325	4.72E-03	1.91E-02	1.58
KIAA1143	7930	3.86E-07	5.20E-06	1.58
DGAT1	8876	3.63E-05	2.94E-04	1.58
ZBTB48	3115	6.51E-05	4.93E-04	1.58
TSPAN31	4947	2.18E-04	1.40E-03	1.58
CPT1B	5787	2.31E-03	1.05E-02	1.58
CEP128	4730	3.88E-03	1.62E-02	1.58
VTA1	14842	1.76E-08	3.09E-07	1.58
CLUH	2628	8.47E-04	4.50E-03	1.58
DUSP18	3693	1.82E-04	1.21E-03	1.58
PIGO	6840	1.38E-04	9.43E-04	1.58
MCAT	835	9.56E-03	3.40E-02	1.58
FRMD8	7627	3.21E-04	1.96E-03	1.58
RPP25L	2775	1.36E-03	6.68E-03	1.58
LTA	5040	6.49E-03	2.47E-02	1.58

ZDHHC16	3426	1.50E-07	2.19E-06	1.59
TAGLN	1374	9.19E-04	4.82E-03	1.59
FUK	2755	7.31E-06	7.22E-05	1.59
PHRF1	5253	7.47E-04	4.05E-03	1.59
MPHOSPH8	40090	1.40E-06	1.65E-05	1.59
TAOK2	2907	3.88E-04	2.30E-03	1.59
TRAPPC9	7819	1.20E-03	6.02E-03	1.59
CYB561D1	6408	2.47E-05	2.12E-04	1.59
LINC01128	4681	2.30E-05	2.00E-04	1.59
RNF40	6730	4.13E-04	2.42E-03	1.59
TRMT1L	4971	1.76E-03	8.36E-03	1.59
CCDC124	5154	8.48E-04	4.50E-03	1.59
ZER1	11559	4.77E-03	1.92E-02	1.59
FARSA	7354	4.30E-09	8.55E-08	1.59
CHST12	3819	1.84E-03	8.69E-03	1.59
KNOP1	6312	6.85E-04	3.75E-03	1.59
GRK2	29422	9.63E-03	3.42E-02	1.59
ANKRD36B	45676	2.03E-04	1.32E-03	1.59
WIPF2	5045	3.78E-05	3.05E-04	1.59
NUCB1	13038	1.32E-05	1.22E-04	1.59
INO80E	2456	1.17E-03	5.92E-03	1.59
LTB4R	4243	1.86E-05	1.65E-04	1.59
NOC2L	8755	1.39E-03	6.81E-03	1.59
CRYBB2P1	4160	3.88E-07	5.23E-06	1.59
BCKDK	4331	4.52E-05	3.57E-04	1.59
GCSAM	5978	9.96E-04	5.16E-03	1.59
PTDSS2	1574	3.01E-04	1.85E-03	1.59
ZBTB17	4490	8.38E-05	6.11E-04	1.59
MAPK8IP3	7425	5.28E-04	2.98E-03	1.59
ZC3H12D	6575	6.97E-05	5.23E-04	1.60
POR	6879	2.44E-06	2.71E-05	1.60
TRAPPC2	10010	7.31E-06	7.22E-05	1.60
CYB5RL	1095	2.08E-03	9.63E-03	1.60
POM121	19832	1.29E-07	1.92E-06	1.60
SNRNP70	22367	5.66E-07	7.33E-06	1.60
PPME1	4007	9.53E-06	9.11E-05	1.60
ZFYVE21	2408	8.64E-04	4.58E-03	1.60
ABCF3	7548	3.66E-04	2.19E-03	1.60
NOP9	2110	1.01E-05	9.64E-05	1.60
RIPOR1	2155	5.88E-05	4.50E-04	1.60
C1GALT1C1	4415	7.67E-04	4.14E-03	1.60
C1orf216	2595	1.66E-03	7.95E-03	1.60
UTP11	2971	2.88E-04	1.78E-03	1.60
NUDT14	1711	2.40E-04	1.52E-03	1.60
CNOT11	16936	1.83E-11	5.57E-10	1.60
CFL1	165895	2.99E-09	6.10E-08	1.60

SCLY	2047	5.48E-03	2.16E-02	1.60
URGCP	5380	1.18E-05	1.10E-04	1.60
BRD4	16836	3.51E-05	2.86E-04	1.60
GPAA1	12470	3.11E-06	3.34E-05	1.60
WDTC1	2351	1.97E-03	9.19E-03	1.60
MEGF6	9306	7.72E-03	2.86E-02	1.60
WDR18	5364	9.17E-06	8.81E-05	1.60
PRKX	17253	2.84E-04	1.76E-03	1.60
FAM35A	7843	1.94E-06	2.21E-05	1.60
EEF1B2	40722	2.68E-04	1.67E-03	1.60
TNFRSF1A	3052	3.26E-04	1.98E-03	1.60
TNFSF12	3331	1.72E-08	3.03E-07	1.60
PHF1	15162	3.07E-07	4.22E-06	1.60
MAVS	17174	1.96E-04	1.28E-03	1.60
TMEM109	18484	1.89E-06	2.16E-05	1.61
NUS1	6788	4.48E-06	4.67E-05	1.61
NFATC2IP	15101	2.84E-05	2.38E-04	1.61
MRPL12	2689	1.26E-04	8.72E-04	1.61
APOL2	3797	2.66E-03	1.18E-02	1.61
GPR137	1149	1.37E-03	6.74E-03	1.61
CETN3	3924	2.66E-03	1.18E-02	1.61
KLF6	114521	1.11E-05	1.05E-04	1.61
LYRM7	18716	1.78E-08	3.12E-07	1.61
IL18BP	4331	4.98E-05	3.89E-04	1.61
SKIV2L	4802	9.19E-04	4.82E-03	1.61
CARD11	13782	2.87E-08	4.80E-07	1.61
KCNAB2	9946	2.08E-06	2.35E-05	1.61
URM1	7237	4.01E-06	4.23E-05	1.61
KCNQ1	4226	3.10E-04	1.89E-03	1.61
ZNF181	5491	8.85E-03	3.20E-02	1.61
SSSCA1	3731	8.65E-05	6.28E-04	1.61
ZBP1	6329	1.95E-05	1.72E-04	1.61
GRAMD1A	9070	5.34E-04	3.01E-03	1.61
CHMP1A	7767	3.50E-07	4.74E-06	1.61
PAQR8	8695	8.38E-03	3.05E-02	1.61
BIN3	9373	8.49E-10	1.89E-08	1.61
SH3GLB2	7493	1.94E-04	1.27E-03	1.62
MED7	4603	1.09E-04	7.72E-04	1.62
C14orf119	8312	6.81E-04	3.74E-03	1.62
NPRL3	2879	1.93E-03	9.03E-03	1.62
MTHFSD	2351	1.76E-04	1.17E-03	1.62
TMEM234	1592	8.02E-05	5.88E-04	1.62
PIP4P1	9952	1.47E-04	9.96E-04	1.62
TRAPPC12	4762	9.05E-07	1.12E-05	1.62
C20orf27	3424	1.70E-04	1.13E-03	1.62
CYHR1	7790	2.91E-06	3.16E-05	1.62

GBP1	17964	2.36E-03	1.07E-02	1.62
XRCC3	1092	1.16E-03	5.87E-03	1.62
ZFP36	48295	3.36E-04	2.03E-03	1.62
TRAPPC1	13429	2.48E-07	3.47E-06	1.62
TTC31	11503	8.02E-05	5.88E-04	1.62
H2AFX	2118	1.43E-02	4.71E-02	1.62
TCOF1	11127	6.29E-05	4.78E-04	1.62
PODXL2	999	1.65E-03	7.89E-03	1.62
OTUD5	16359	5.68E-06	5.76E-05	1.63
SPDYE2	1160	2.53E-03	1.13E-02	1.63
SPDYE2B	1160	2.53E-03	1.13E-02	1.63
EID2B	1538	3.44E-03	1.47E-02	1.63
SLC35A2	2723	7.75E-05	5.72E-04	1.63
PLD2	1396	9.66E-03	3.43E-02	1.63
GLRX2	1378	6.68E-03	2.53E-02	1.63
CHORDC1	8073	1.38E-07	2.04E-06	1.63
ZFP3	1469	1.48E-02	4.83E-02	1.63
PPIL1	3807	5.43E-04	3.05E-03	1.63
C1orf109	3608	1.88E-04	1.24E-03	1.63
C17orf51	999	3.80E-04	2.26E-03	1.63
FAM89B	11498	3.93E-05	3.16E-04	1.63
ZSWIM8	4062	2.64E-03	1.18E-02	1.63
BAHD1	1478	3.35E-04	2.03E-03	1.63
LOC101928673	1121	5.77E-04	3.22E-03	1.63
SSBP3-AS1	960	1.33E-02	4.46E-02	1.63
RIPK3	4832	1.37E-06	1.63E-05	1.63
UBAP1	8230	4.20E-04	2.46E-03	1.63
AFG3L1P	6461	5.09E-04	2.89E-03	1.63
KLHL21	7710	7.18E-05	5.36E-04	1.63
SLC48A1	2829	1.06E-04	7.48E-04	1.63
MGAT4B	3826	8.98E-07	1.11E-05	1.63
NELFB	3813	2.09E-04	1.35E-03	1.63
RRN3P2	5003	6.33E-04	3.49E-03	1.63
ZNHIT2	2038	1.20E-05	1.12E-04	1.63
SDHAF1	3222	3.89E-04	2.30E-03	1.63
MGME1	2438	8.86E-03	3.20E-02	1.63
SLF2	14399	1.22E-06	1.46E-05	1.63
AGPAT2	2240	2.98E-03	1.31E-02	1.63
PIM3	8005	1.22E-03	6.10E-03	1.64
PSMC1	8633	4.51E-06	4.70E-05	1.64
FAM193B	9114	1.87E-04	1.23E-03	1.64
ZNF598	2290	2.46E-04	1.56E-03	1.64
CYTH4	13416	1.64E-06	1.90E-05	1.64
AARS2	3806	2.16E-04	1.39E-03	1.64
LINC02486	2743	1.29E-02	4.35E-02	1.64
CROCCP2	8050	1.54E-04	1.04E-03	1.64

STRN4	5170	2.32E-04	1.48E-03	1.64
RGPD8	2984	1.33E-02	4.46E-02	1.64
BORCS6	2939	5.56E-05	4.29E-04	1.64
SLC22A18	1583	9.25E-04	4.85E-03	1.64
RABL6	4238	1.02E-06	1.24E-05	1.64
ASF1A	20286	6.83E-06	6.79E-05	1.64
CD40LG	15025	3.73E-03	1.57E-02	1.64
PTPA	1913	1.19E-03	5.98E-03	1.64
SERF2	106454	2.62E-05	2.22E-04	1.64
TAF1A	1514	1.14E-03	5.81E-03	1.64
C17orf62	27865	1.70E-10	4.39E-09	1.64
ARL10	1209	4.75E-04	2.72E-03	1.65
NLRX1	1688	1.00E-02	3.53E-02	1.65
ENSA	10574	3.05E-08	5.07E-07	1.65
PCNT	16056	3.50E-03	1.49E-02	1.65
NEURL4	1476	1.26E-02	4.26E-02	1.65
RAD51-AS1	10519	3.64E-03	1.54E-02	1.65
LOC652276	3370	6.51E-03	2.48E-02	1.65
TMEM91	1099	1.84E-03	8.69E-03	1.65
ARMC6	4220	3.76E-04	2.24E-03	1.65
ZNF408	2318	1.19E-03	5.98E-03	1.65
TRIM74	549	3.96E-03	1.65E-02	1.65
RXRB	3355	5.24E-06	5.35E-05	1.65
TCAF2	7745	1.85E-04	1.22E-03	1.65
PRR13	46763	2.26E-08	3.88E-07	1.65
YIF1B	3772	9.63E-07	1.18E-05	1.65
FBXO48	1130	3.13E-03	1.36E-02	1.65
UBB	144143	1.59E-05	1.44E-04	1.66
PET100	9682	1.00E-03	5.19E-03	1.66
NUDT22	5557	2.41E-05	2.07E-04	1.66
SORD	894	2.92E-03	1.28E-02	1.66
NDUFAF1	3199	1.88E-04	1.24E-03	1.66
MCRIP2	3005	1.25E-04	8.64E-04	1.66
ACOT2	3230	6.27E-03	2.41E-02	1.66
LINC01560	784	7.77E-03	2.87E-02	1.66
IFFO2	1452	3.94E-03	1.64E-02	1.66
DUS1L	18535	6.69E-05	5.05E-04	1.66
NUDT18	2122	1.08E-04	7.63E-04	1.66
RAB4B	8374	3.25E-06	3.48E-05	1.66
TAGLN2	90675	1.12E-05	1.05E-04	1.66
DPY30	10620	6.41E-05	4.86E-04	1.66
THOC3	7403	3.93E-05	3.16E-04	1.66
TOLLIP	4919	1.67E-06	1.93E-05	1.66
CREBZF	64774	8.37E-06	8.16E-05	1.66
MIAT	22266	1.68E-03	8.05E-03	1.66
RNF31	8527	5.41E-08	8.62E-07	1.66

MTRNR2L9	2148	4.30E-03	1.76E-02	1.67
ACTB	1242610	4.23E-07	5.65E-06	1.67
AP1G2	48464	4.16E-05	3.32E-04	1.67
RUSC1	2964	6.78E-06	6.76E-05	1.67
MAPK7	1722	2.63E-04	1.65E-03	1.67
PSD	427	1.50E-02	4.90E-02	1.67
TRIP11	23372	1.68E-04	1.12E-03	1.67
ZNF544	12784	1.24E-05	1.15E-04	1.67
PIGM	6596	2.04E-05	1.79E-04	1.67
GUSBP11	9792	1.58E-04	1.06E-03	1.67
ZNF879	3779	5.72E-03	2.23E-02	1.67
ENDOV	1013	3.56E-03	1.51E-02	1.67
SLC16A1-AS1	1493	2.11E-06	2.38E-05	1.67
UQCC1	4115	1.83E-05	1.62E-04	1.67
ABI3	2788	2.26E-03	1.03E-02	1.67
UBQLN4	2050	5.60E-03	2.19E-02	1.67
ZNF316	3739	2.20E-03	1.01E-02	1.67
SFI1	23162	4.23E-05	3.36E-04	1.67
MID1IP1	3199	8.07E-04	4.32E-03	1.67
DDTL	1124	1.28E-02	4.32E-02	1.68
ZNF791	10792	1.02E-04	7.26E-04	1.68
FBRS	2677	1.09E-05	1.02E-04	1.68
TESK1	1440	3.98E-04	2.35E-03	1.68
GYS1	6437	8.88E-06	8.57E-05	1.68
TMUB2	14161	1.20E-10	3.17E-09	1.68
PPP1R18	9474	1.50E-08	2.69E-07	1.68
MKLN1-AS	659	1.76E-03	8.36E-03	1.68
DLGAP4	5622	4.99E-06	5.14E-05	1.68
UNC93B1	6167	1.90E-03	8.93E-03	1.68
CARNS1	1166	7.76E-04	4.19E-03	1.68
IRF9	23821	8.50E-07	1.06E-05	1.68
B4GALT7	2569	5.57E-05	4.30E-04	1.68
GRAMD4	1517	3.02E-03	1.32E-02	1.68
MARK2	9795	1.03E-03	5.32E-03	1.68
IFI44L	6690	5.07E-03	2.02E-02	1.68
RBM42	5682	5.72E-05	4.40E-04	1.69
OXLD1	16108	2.83E-06	3.08E-05	1.69
PHLDB3	743	7.83E-03	2.89E-02	1.69
DOCK9-AS2	977	9.93E-03	3.51E-02	1.69
EVA1C	2293	3.02E-03	1.32E-02	1.69
ABTB1	14568	2.28E-05	1.98E-04	1.69
C12orf43	4377	1.11E-06	1.34E-05	1.69
FBXO46	1732	4.81E-04	2.75E-03	1.69
RGPD6	2281	6.59E-04	3.62E-03	1.69
ATG7	7165	1.52E-05	1.38E-04	1.69
SYMPK	4288	1.26E-04	8.70E-04	1.69

TRAF7	1703	1.36E-05	1.25E-04	1.69
MSANTD2	2441	1.69E-05	1.51E-04	1.69
ARMT1	4164	9.27E-05	6.67E-04	1.69
UST	1393	1.80E-03	8.51E-03	1.70
PPP1R11	15836	3.29E-08	5.44E-07	1.70
CSRNP2	3505	8.76E-04	4.63E-03	1.70
COX10-AS1	3465	2.62E-06	2.88E-05	1.70
LINC00921	1697	9.64E-03	3.42E-02	1.70
PLA2G4B	3841	4.25E-03	1.75E-02	1.70
TTLL3	4195	1.37E-03	6.76E-03	1.70
RPL18A	115952	5.70E-05	4.38E-04	1.70
KIAA1841	1288	1.35E-02	4.52E-02	1.70
ATG4D	3389	2.66E-05	2.25E-04	1.70
FBXL15	7326	8.42E-07	1.05E-05	1.70
RALGDS	20381	8.31E-04	4.42E-03	1.70
ARIH2OS	1758	2.53E-04	1.59E-03	1.70
HDAC6	9048	3.75E-04	2.23E-03	1.70
LOC642361	2191	2.05E-04	1.33E-03	1.70
PDCD7	6329	2.60E-12	8.89E-11	1.70
ATF6B	8152	4.32E-07	5.76E-06	1.70
SH2D2A	3751	2.65E-04	1.66E-03	1.70
POLG	13616	4.42E-05	3.50E-04	1.71
MKNK2	8460	2.45E-05	2.10E-04	1.71
SMARCA4	16036	8.78E-11	2.36E-09	1.71
ELOC	8585	2.72E-06	2.97E-05	1.71
GRK6	29175	7.48E-09	1.42E-07	1.71
VARS	7036	6.62E-04	3.64E-03	1.71
ARRDC1-AS1	4198	2.86E-16	1.65E-14	1.71
TJP3	2246	4.73E-03	1.91E-02	1.71
MGC12916	1616	1.71E-03	8.19E-03	1.71
GLUD1P3	2152	9.10E-05	6.57E-04	1.71
ALKBH6	4382	6.00E-09	1.16E-07	1.71
STX16-NPEPL1	4542	8.47E-05	6.17E-04	1.71
LENG8	12947	1.05E-09	2.31E-08	1.71
CSK	11655	4.96E-10	1.16E-08	1.71
ISYNA1	4994	2.00E-07	2.85E-06	1.71
C5orf15	13439	2.54E-11	7.54E-10	1.71
TRIM8	8454	1.42E-07	2.08E-06	1.71
HMGA1	5663	1.06E-07	1.61E-06	1.71
TOMM40	3264	3.74E-06	3.97E-05	1.71
CXXC1	16738	6.25E-09	1.20E-07	1.72
IL4R	19120	1.52E-06	1.79E-05	1.72
STK25	17224	2.52E-15	1.30E-13	1.72
FAM87B	594	6.78E-03	2.56E-02	1.72
ZNF626	3539	1.44E-04	9.76E-04	1.72
PRR14	3431	5.52E-09	1.08E-07	1.72

FAM89A	1987	3.08E-04	1.88E-03	1.72
FXR2	2322	1.02E-04	7.27E-04	1.72
TJAP1	3255	1.43E-06	1.69E-05	1.72
PDK2	7058	2.23E-06	2.50E-05	1.72
COX17	5751	4.03E-03	1.67E-02	1.72
DPH2	7225	2.97E-06	3.21E-05	1.72
APIP	3407	2.01E-04	1.31E-03	1.72
PROCA1	616	3.14E-03	1.36E-02	1.72
KEAP1	3836	5.76E-05	4.42E-04	1.72
BSG	34787	5.55E-07	7.20E-06	1.72
ANKMY1	5470	3.34E-06	3.58E-05	1.72
CEP250	7215	2.43E-06	2.70E-05	1.72
CYB561D2	9985	4.56E-07	6.04E-06	1.72
HAPLN3	10150	8.85E-05	6.41E-04	1.72
FDX1L	1206	2.10E-04	1.36E-03	1.72
PRKAG2-AS1	867	1.51E-03	7.36E-03	1.73
IL17RC	460	1.44E-02	4.75E-02	1.73
EHBP1L1	4674	1.01E-05	9.64E-05	1.73
SPECC1L-ADORA2A	9729	7.08E-05	5.29E-04	1.73
BATF	4698	2.23E-03	1.02E-02	1.73
NBPF26	7397	2.78E-06	3.04E-05	1.73
REEP4	2968	7.08E-05	5.29E-04	1.73
DOK2	20214	2.08E-10	5.25E-09	1.73
PCSK7	29868	2.27E-05	1.98E-04	1.73
KLHL34	734	8.72E-03	3.16E-02	1.73
DCUN1D3	805	3.98E-03	1.65E-02	1.73
RSRP1	113562	2.92E-07	4.03E-06	1.73
PTPN23	1950	1.88E-06	2.15E-05	1.73
DHFR2	1973	7.41E-04	4.02E-03	1.74
GSTM2	8465	1.48E-03	7.25E-03	1.74
JUND	27903	5.09E-03	2.03E-02	1.74
EXOSC4	1419	5.63E-04	3.15E-03	1.74
SDHD	20324	3.85E-07	5.19E-06	1.74
MED26	1517	2.56E-04	1.61E-03	1.74
DHRS13	3355	9.46E-07	1.16E-05	1.74
PICK1	2022	2.86E-04	1.77E-03	1.74
TTLL12	3588	6.44E-05	4.88E-04	1.74
CCDC71	434	9.11E-03	3.27E-02	1.74
GCLM	4525	1.05E-06	1.27E-05	1.74
VPS9D1	1556	1.42E-03	6.97E-03	1.74
CTDP1	4360	7.71E-06	7.58E-05	1.74
RNF185	5148	1.12E-06	1.34E-05	1.74
SLC8B1	18364	5.19E-06	5.31E-05	1.74
TMEM94	5355	7.90E-05	5.82E-04	1.74
GAK	12172	1.28E-09	2.76E-08	1.74

EFHD2	16409	1.86E-04	1.23E-03	1.74
PLGLB1	796	7.58E-04	4.10E-03	1.74
SMG5	7582	6.15E-05	4.69E-04	1.74
PPP1R9B	1130	1.67E-04	1.12E-03	1.75
PLGLB2	745	7.73E-04	4.17E-03	1.75
AEBP2	11532	2.12E-08	3.69E-07	1.75
TRMT1	14773	1.56E-07	2.27E-06	1.75
SH3BP1	2927	5.47E-06	5.56E-05	1.75
MAFG	1935	1.02E-05	9.65E-05	1.75
TMIE	530	4.88E-03	1.95E-02	1.75
PI4KAP1	4301	2.40E-03	1.08E-02	1.75
CCZ1	7196	4.62E-07	6.10E-06	1.75
LSP1	55003	2.14E-07	3.03E-06	1.75
IKBIP	2258	7.83E-04	4.21E-03	1.75
ZNF17	1873	9.04E-04	4.76E-03	1.75
SPOUT1	8200	3.10E-08	5.15E-07	1.75
PLEKHM1P1	6400	1.70E-05	1.52E-04	1.75
ZNF516	3841	9.69E-05	6.94E-04	1.75
AGPAT4-IT1	509	1.11E-02	3.85E-02	1.75
DDX11	6217	7.00E-04	3.82E-03	1.75
ZFPL1	2544	1.33E-08	2.42E-07	1.75
LOC645967	433	3.93E-03	1.64E-02	1.75
ASPSCR1	4252	4.11E-09	8.20E-08	1.76
GGA1	6691	7.42E-13	2.73E-11	1.76
GCDH	3731	6.17E-07	7.90E-06	1.76
MAP4K1	19325	1.68E-08	2.97E-07	1.76
ZNF212	3294	3.19E-07	4.37E-06	1.76
RALGAPA1	14015	2.91E-05	2.44E-04	1.76
SBF1	12136	4.71E-06	4.88E-05	1.76
NT5DC3	1778	5.67E-04	3.17E-03	1.76
CBX7	6565	5.93E-05	4.53E-04	1.76
ABHD8	845	6.33E-03	2.42E-02	1.76
KAT2A	6554	2.11E-04	1.36E-03	1.76
PTPRCAP	58699	2.95E-06	3.20E-05	1.76
TMEM184B	5018	3.82E-10	9.19E-09	1.76
ZNF37A	10910	8.98E-04	4.73E-03	1.76
MEN1	8861	8.54E-08	1.32E-06	1.76
IL32	125318	3.10E-05	2.57E-04	1.76
GUSBP4	2241	4.53E-04	2.63E-03	1.76
NPHP4	1207	4.27E-03	1.75E-02	1.76
RASA4CP	2946	6.64E-07	8.43E-06	1.76
IMPDH1	7287	2.96E-06	3.21E-05	1.76
TSPAN17	3866	3.93E-05	3.16E-04	1.76
LRSAM1	3297	2.50E-05	2.14E-04	1.77
HSPBP1	3527	3.75E-06	3.98E-05	1.77
CDK11A	11108	4.22E-03	1.74E-02	1.77

IFIT1	1341	3.15E-03	1.37E-02	1.77
TSNARE1	3034	6.21E-06	6.25E-05	1.77
CYCS	22683	2.54E-08	4.28E-07	1.77
THOP1	3505	2.98E-05	2.49E-04	1.77
PROSER3	1105	1.42E-05	1.30E-04	1.77
CHST7	1754	5.86E-04	3.27E-03	1.77
FKBP1A	6654	1.57E-08	2.80E-07	1.77
LOC100507250	830	2.40E-03	1.08E-02	1.77
TMEM63A	30876	1.12E-05	1.05E-04	1.77
AMACR	1742	1.05E-03	5.41E-03	1.77
SOGA3	2435	1.44E-02	4.75E-02	1.77
SLC25A25	4085	1.52E-03	7.39E-03	1.77
NAA60	8375	1.31E-07	1.95E-06	1.77
MBD3	4061	1.22E-07	1.83E-06	1.77
ZNF680	5278	2.36E-08	4.02E-07	1.77
MAZ	8567	7.25E-06	7.17E-05	1.77
SLC26A6	4181	3.25E-05	2.67E-04	1.77
PIK3CD	38674	7.19E-08	1.13E-06	1.77
NAT6	2918	2.19E-07	3.09E-06	1.77
ATG9A	7818	4.36E-06	4.55E-05	1.77
C9orf16	6701	9.38E-06	8.99E-05	1.77
AP1S1	476	6.31E-03	2.42E-02	1.77
TAF6	8636	4.04E-09	8.10E-08	1.77
REPIN1	14368	5.09E-05	3.97E-04	1.77
CTBP1-AS	405	9.48E-03	3.38E-02	1.77
CLUHP3	6816	3.92E-03	1.64E-02	1.77
IRF2BPL	2325	2.42E-04	1.53E-03	1.78
PSKH1	5091	7.34E-04	3.99E-03	1.78
LMF1	9683	2.96E-09	6.05E-08	1.78
DUS3L	7921	8.11E-07	1.01E-05	1.78
GATA3	22560	6.37E-09	1.23E-07	1.78
CTU2	1627	6.85E-05	5.15E-04	1.78
PTRH1	1343	2.93E-05	2.45E-04	1.78
ZNF672	3304	5.07E-06	5.21E-05	1.78
DDX51	6464	3.67E-05	2.97E-04	1.78
MYC	45352	2.26E-07	3.19E-06	1.78
MZF1	5634	7.50E-14	3.26E-12	1.78
FERMT3	23866	5.65E-11	1.58E-09	1.78
PRKD2	12173	9.63E-06	9.20E-05	1.78
PATJ	29684	3.98E-04	2.35E-03	1.78
STX5	3092	8.85E-06	8.55E-05	1.78
GMIP	10538	7.62E-05	5.64E-04	1.78
ZNF74	1381	2.80E-03	1.24E-02	1.78
GALK1	1852	1.61E-05	1.45E-04	1.79
HYPK	7730	9.89E-09	1.84E-07	1.79
SRSF1	31655	7.70E-08	1.20E-06	1.79

AHSA2	30556	3.74E-08	6.14E-07	1.79
RNF5	753	7.79E-03	2.88E-02	1.79
ZNF497	1009	8.66E-05	6.28E-04	1.79
DPP7	32795	7.38E-11	2.02E-09	1.79
LRRC47	7728	2.66E-14	1.20E-12	1.79
CIRBP-AS1	726	1.52E-03	7.39E-03	1.79
LINC00324	4115	2.25E-04	1.44E-03	1.79
ZNF500	5123	9.56E-08	1.46E-06	1.79
ANKRD13D	6112	1.05E-08	1.94E-07	1.79
ARSA	11223	9.14E-06	8.79E-05	1.79
CES2	9079	3.05E-10	7.48E-09	1.79
TIAF1	1884	5.02E-05	3.92E-04	1.79
ST6GALNAC1	3453	1.87E-03	8.80E-03	1.79
FAM200A	1368	1.25E-02	4.26E-02	1.79
CNKSR1	656	1.33E-02	4.46E-02	1.79
PRKAR1B	1953	7.41E-05	5.51E-04	1.79
AP1B1	6504	5.26E-07	6.85E-06	1.80
SLC39A3	4879	1.42E-08	2.56E-07	1.80
SLC25A23	3987	7.17E-04	3.91E-03	1.80
GATD1	4202	1.16E-07	1.74E-06	1.80
TSEN54	10402	3.97E-07	5.34E-06	1.80
SMG1P2	7700	1.20E-08	2.20E-07	1.80
ARMC10	6109	3.35E-12	1.13E-10	1.80
MEMO1	5050	8.08E-11	2.19E-09	1.80
RFX2	931	1.87E-03	8.81E-03	1.80
EDC4	13058	1.62E-04	1.09E-03	1.80
LOC646471	2095	1.82E-03	8.62E-03	1.80
CCDC97	9436	1.01E-15	5.51E-14	1.80
ZNF446	1858	1.02E-06	1.24E-05	1.80
ZGPAT	15182	5.63E-13	2.13E-11	1.80
MYBBP1A	7624	1.33E-05	1.22E-04	1.80
HDAC7	8754	5.08E-07	6.63E-06	1.80
ITGA5	12778	2.91E-04	1.79E-03	1.80
EEF2KMT	1660	4.09E-05	3.27E-04	1.81
ELOVL1	6353	1.51E-08	2.70E-07	1.81
ZC3H7B	2755	3.85E-08	6.30E-07	1.81
C16orf86	559	4.52E-03	1.84E-02	1.81
RCCD1	1273	2.12E-03	9.76E-03	1.81
FAM96B	7603	1.45E-08	2.62E-07	1.81
LOC285074	5557	1.45E-06	1.72E-05	1.81
DHX34	2926	4.42E-04	2.57E-03	1.81
ZNF322	2765	1.47E-02	4.83E-02	1.81
TSSC4	6851	4.36E-06	4.55E-05	1.81
COL6A1	1296	6.73E-03	2.55E-02	1.81
SLC25A42	4536	1.61E-06	1.87E-05	1.81
LFNG	1187	9.36E-03	3.35E-02	1.81

MOSPD3	1876	1.34E-05	1.23E-04	1.81
AAMP	10299	1.46E-11	4.49E-10	1.81
LOC152048	1080	2.45E-04	1.55E-03	1.81
SCNN1D	655	6.21E-03	2.39E-02	1.81
TMBIM1	19615	2.39E-05	2.06E-04	1.81
GPR35	621	4.97E-03	1.98E-02	1.81
MOB2	5215	3.31E-07	4.51E-06	1.82
RFNG	2361	4.80E-04	2.74E-03	1.82
SIRT7	11435	1.11E-09	2.42E-08	1.82
MAP4K2	8214	3.77E-08	6.18E-07	1.82
LOC100129203	2545	3.36E-04	2.03E-03	1.82
CLDN15	1185	1.21E-06	1.45E-05	1.82
TNFRSF14	12966	2.25E-09	4.66E-08	1.82
C11orf24	2145	8.10E-04	4.33E-03	1.82
SLC27A3	1531	3.04E-04	1.86E-03	1.82
PCNX3	2643	3.05E-05	2.53E-04	1.82
RAB11FIP5	1785	1.12E-04	7.89E-04	1.82
TUBG2	2792	1.89E-04	1.24E-03	1.82
RGS12	1352	7.23E-03	2.71E-02	1.82
PPP6R1	15153	9.23E-05	6.65E-04	1.82
KLHL22	3582	4.72E-05	3.71E-04	1.82
LOC728673	279	1.36E-02	4.54E-02	1.82
PGS1	7553	5.20E-08	8.31E-07	1.82
ABCD1	512	1.22E-02	4.17E-02	1.82
P2RX7	4542	3.13E-05	2.59E-04	1.82
SLC2A4RG	2344	2.08E-04	1.35E-03	1.82
ATP6V0C	7209	1.68E-09	3.57E-08	1.83
TNFSF10	15459	4.86E-06	5.02E-05	1.83
ARHGEF39	1797	1.32E-04	9.08E-04	1.83
HGS	18672	3.99E-07	5.36E-06	1.83
TBC1D24	689	9.33E-05	6.71E-04	1.83
ELL	3436	3.84E-06	4.07E-05	1.83
SAMD12	2077	4.86E-03	1.95E-02	1.83
ACBD4	3002	8.65E-06	8.40E-05	1.83
CEBPB	4849	8.43E-06	8.21E-05	1.83
PIGBOS1	3217	5.63E-07	7.29E-06	1.83
CHPF2	7558	3.22E-04	1.96E-03	1.83
ULK3	30270	4.56E-07	6.04E-06	1.83
MFSD3	2404	4.97E-07	6.52E-06	1.83
POMT1	8461	4.22E-10	1.00E-08	1.83
CCDC130	12994	6.61E-11	1.84E-09	1.84
SLC26A11	5146	2.60E-08	4.37E-07	1.84
C8orf82	1496	7.53E-05	5.59E-04	1.84
SCRIB	998	1.61E-03	7.76E-03	1.84
LRRC61	1649	4.17E-04	2.44E-03	1.84
LOC100506123	1169	2.03E-04	1.32E-03	1.84

CTBP1	15858	2.87E-05	2.41E-04	1.84
USP19	9752	1.11E-06	1.34E-05	1.84
TOR2A	2729	6.34E-07	8.08E-06	1.84
NAALADL1	2083	3.60E-04	2.16E-03	1.84
AGAP6	3640	1.06E-05	1.01E-04	1.84
ENDOG	1767	1.26E-04	8.74E-04	1.84
TBC1D17	3842	5.19E-12	1.68E-10	1.84
TIMM23B	5050	1.70E-08	3.00E-07	1.84
PPP2R3B	2156	6.86E-07	8.67E-06	1.84
VIPR1	7431	9.72E-06	9.28E-05	1.84
ARL8A	1926	2.00E-05	1.76E-04	1.84
DGKQ	6083	2.51E-03	1.13E-02	1.84
CRTC1	1851	2.83E-09	5.80E-08	1.84
RTEL1	2557	4.02E-06	4.24E-05	1.84
LLPH	8054	8.06E-07	1.01E-05	1.85
HELZ2	1820	1.58E-06	1.84E-05	1.85
EHD4	3316	4.63E-06	4.81E-05	1.85
SLC38A7	2375	1.36E-07	2.01E-06	1.85
RTL8A	3813	2.08E-07	2.96E-06	1.85
JAK3	32073	3.74E-13	1.45E-11	1.85
MCOLN1	1245	1.29E-04	8.88E-04	1.85
TMEM191A	1442	7.96E-05	5.84E-04	1.85
SLC19A1	2068	5.15E-05	4.02E-04	1.85
BMP8A	963	4.23E-04	2.47E-03	1.85
PKN3	461	2.63E-03	1.17E-02	1.85
TRIM62	2771	3.87E-05	3.11E-04	1.85
LOC154761	5623	8.46E-06	8.23E-05	1.86
SRP9	74060	1.66E-16	9.92E-15	1.86
ZNF48	1709	1.62E-03	7.77E-03	1.86
LOC100996724	1703	6.38E-03	2.44E-02	1.86
CDAN1	3123	1.22E-05	1.14E-04	1.86
PPDPF	7225	9.27E-05	6.67E-04	1.86
SPATA6	1039	7.58E-03	2.81E-02	1.86
PLD3	7204	2.71E-07	3.76E-06	1.86
GPAT2	753	7.51E-03	2.79E-02	1.86
TAPBP	100746	1.23E-11	3.81E-10	1.86
MYO1C	2066	2.55E-04	1.61E-03	1.86
LSR	12509	2.68E-07	3.73E-06	1.86
GMPPB	3982	3.44E-07	4.67E-06	1.86
ZNF764	1517	6.71E-04	3.69E-03	1.86
CHRNB1	1674	2.26E-04	1.45E-03	1.86
RGS3	1033	1.03E-02	3.62E-02	1.86
BICDL1	4547	7.85E-06	7.69E-05	1.86
AP5B1	4709	5.73E-07	7.41E-06	1.87
MBD6	1575	1.69E-05	1.51E-04	1.87
CZ1P-ASNS	8836	1.23E-05	1.14E-04	1.87

CAPN10-AS1	476	6.25E-03	2.40E-02	1.87
CDK13	18233	1.51E-13	6.24E-12	1.87
ZNF420	8719	1.63E-06	1.89E-05	1.87
HSPA1A	4120	3.37E-05	2.76E-04	1.87
TMEM86B	735	3.70E-04	2.20E-03	1.87
MIR4697HG	786	1.82E-03	8.62E-03	1.87
SYTL1	29408	3.07E-10	7.51E-09	1.87
FAM161B	826	1.23E-04	8.55E-04	1.87
GON7	2986	1.94E-05	1.71E-04	1.87
HIP1R	6657	5.52E-05	4.27E-04	1.87
MICAL3	1590	6.81E-04	3.73E-03	1.87
CD58	9242	1.20E-04	8.40E-04	1.87
ARL16	10082	9.71E-05	6.95E-04	1.87
RPL23AP64	451	6.25E-04	3.46E-03	1.87
ZNF319	1599	1.24E-03	6.20E-03	1.88
RPL10	117473	3.86E-09	7.77E-08	1.88
SPSB3	24348	1.45E-09	3.10E-08	1.88
KLHL26	1230	1.03E-03	5.32E-03	1.88
LINC00528	2104	3.29E-07	4.49E-06	1.88
TRIM3	929	9.85E-04	5.11E-03	1.88
GOLGA7B	1865	3.09E-06	3.33E-05	1.88
CANT1	1818	9.74E-05	6.97E-04	1.88
SLC27A4	1259	4.10E-04	2.41E-03	1.88
RCN1	2518	1.15E-06	1.38E-05	1.88
RHOF	12379	2.36E-09	4.86E-08	1.88
PNPLA2	7988	2.46E-07	3.45E-06	1.88
MEGF8	852	5.42E-06	5.52E-05	1.88
TMEM161A	4879	1.46E-08	2.63E-07	1.88
PAGR1	9309	1.47E-14	6.99E-13	1.88
SUN2	95826	4.01E-06	4.23E-05	1.88
TBCCD1	5038	1.01E-05	9.63E-05	1.89
D2HGDH	3928	1.54E-08	2.75E-07	1.89
CCDC9	1617	2.33E-05	2.02E-04	1.89
EML2	4269	6.34E-09	1.22E-07	1.89
ZNF335	10199	7.85E-06	7.69E-05	1.89
SPIN2B	3775	8.49E-09	1.60E-07	1.89
MED15	18723	1.83E-11	5.56E-10	1.89
ZNF276	27541	6.34E-06	6.36E-05	1.89
RPL29	44576	4.43E-08	7.17E-07	1.89
BET1	5665	1.42E-08	2.57E-07	1.89
TXNRD2	2795	1.54E-07	2.24E-06	1.89
JMJD4	5566	2.31E-13	9.23E-12	1.89
H1FX	22176	2.03E-06	2.30E-05	1.90
STK11IP	3753	1.61E-10	4.18E-09	1.90
FBXO2	825	2.16E-03	9.95E-03	1.90
CDIP1	3877	9.06E-08	1.39E-06	1.90

PGBD2	3720	6.30E-06	6.33E-05	1.90
LPAR5	1783	3.38E-04	2.04E-03	1.90
ACTR3C	3454	3.21E-07	4.40E-06	1.90
YIPF4	11686	2.09E-12	7.28E-11	1.90
HES6	403	1.01E-02	3.56E-02	1.90
SLC17A9	435	7.55E-03	2.80E-02	1.90
BCAT2	2907	7.14E-10	1.61E-08	1.90
FBXL18	1338	7.09E-06	7.03E-05	1.90
MYO1F	24775	1.24E-05	1.15E-04	1.90
MVD	3357	9.76E-09	1.82E-07	1.90
RHBDF2	3533	2.10E-06	2.37E-05	1.91
SOCS2	6296	2.73E-03	1.21E-02	1.91
BLOC1S5	5918	6.87E-07	8.68E-06	1.91
MICAL1	17991	1.26E-05	1.16E-04	1.91
HEXDC	9025	7.32E-08	1.14E-06	1.91
ARFRP1	10599	1.25E-25	1.47E-23	1.91
ARID3B	5563	1.50E-07	2.19E-06	1.91
LOC728743	7386	1.79E-15	9.32E-14	1.91
GFOD2	2667	2.98E-05	2.49E-04	1.91
SMIM30	8203	4.54E-05	3.58E-04	1.91
IRF5	2292	6.20E-04	3.43E-03	1.91
PIK3R2	2704	8.79E-06	8.51E-05	1.91
MUC20	1081	4.47E-03	1.82E-02	1.91
RAB35	2455	4.20E-04	2.46E-03	1.92
SHC1	5603	7.83E-06	7.68E-05	1.92
HECTD3	8610	7.35E-09	1.40E-07	1.92
IRF1	60543	1.05E-05	9.92E-05	1.92
UBE2T	995	2.80E-04	1.74E-03	1.92
KCTD17	1495	1.94E-05	1.71E-04	1.92
FAM160B2	10210	1.94E-06	2.21E-05	1.92
DVL1	1696	1.66E-07	2.40E-06	1.92
OR2A20P	617	6.30E-03	2.41E-02	1.92
ARHGAP1	17745	2.76E-09	5.66E-08	1.92
STK11	6777	3.58E-07	4.84E-06	1.92
HMGB1	102460	1.83E-28	2.58E-26	1.92
SEMA4C	1682	1.90E-06	2.17E-05	1.92
RFXAP	3872	8.43E-08	1.30E-06	1.92
GIMAP1	24056	1.49E-15	7.89E-14	1.92
MMP25-AS1	3882	7.09E-07	8.94E-06	1.92
WDR81	8400	4.12E-05	3.29E-04	1.93
CYSLTR2	1140	4.30E-03	1.76E-02	1.93
LOC148413	2761	2.19E-05	1.91E-04	1.93
ZNF540	3445	2.51E-04	1.59E-03	1.93
CCDC102A	1050	2.69E-03	1.19E-02	1.93
RDH13	4664	1.92E-08	3.36E-07	1.93
ZNF428	3017	1.82E-10	4.67E-09	1.93

SLC9A3R1	39348	3.33E-15	1.70E-13	1.93
TYMP	5435	6.46E-04	3.56E-03	1.93
CUL7	2348	3.28E-04	1.99E-03	1.93
GRINA	1244	9.86E-05	7.05E-04	1.93
DCAF15	1792	3.44E-07	4.67E-06	1.94
LOC101927151	1300	3.07E-05	2.54E-04	1.94
ZNF771	429	3.84E-03	1.61E-02	1.94
KATNB1	5673	3.06E-10	7.49E-09	1.94
PTCD1	2906	5.30E-05	4.12E-04	1.94
CNTNAP1	688	8.05E-04	4.31E-03	1.94
E4F1	4655	2.70E-10	6.70E-09	1.94
NLRP1	72915	8.86E-05	6.42E-04	1.94
RAB11FIP3	2733	5.88E-05	4.50E-04	1.94
RPS25	199070	7.34E-09	1.40E-07	1.94
EPHA1	4693	3.49E-05	2.85E-04	1.94
SORBS3	7947	9.93E-07	1.21E-05	1.94
TMUB1	6571	1.15E-09	2.50E-08	1.94
LINC01000	4677	1.11E-06	1.34E-05	1.94
SNAPC2	1408	2.48E-05	2.13E-04	1.94
LOC105376805	1717	1.13E-03	5.77E-03	1.94
TRIM65	4071	1.34E-07	1.98E-06	1.94
PTGER2	30078	2.07E-07	2.95E-06	1.94
MOB3A	14258	6.18E-08	9.75E-07	1.95
DMPK	734	1.97E-04	1.29E-03	1.95
C14orf132	2165	8.01E-04	4.29E-03	1.95
OSBPL7	4533	4.25E-07	5.67E-06	1.95
ZNF574	4173	6.07E-06	6.13E-05	1.95
MOGS	17181	8.27E-12	2.61E-10	1.95
PIM1	42840	2.12E-06	2.38E-05	1.95
TICAM1	1776	7.05E-05	5.28E-04	1.95
HSF1	5944	1.35E-06	1.60E-05	1.95
FAM222B	5068	1.04E-09	2.29E-08	1.95
INPPL1	2895	2.70E-06	2.96E-05	1.95
ASAP1-IT2	879	1.22E-02	4.17E-02	1.95
ARHGAP33	481	4.40E-04	2.56E-03	1.95
RHBDD3	1259	2.86E-07	3.96E-06	1.95
CBX6	6343	4.27E-10	1.01E-08	1.95
WBP2	16656	1.71E-14	8.04E-13	1.96
ANGPT2	1144	1.16E-05	1.09E-04	1.96
LOC441242	1007	4.77E-07	6.27E-06	1.96
DEXI	3765	2.43E-09	4.99E-08	1.96
LDOC1	4113	4.88E-05	3.83E-04	1.96
TIMM29	2851	2.26E-08	3.89E-07	1.96
CLPTM1	3974	2.29E-07	3.22E-06	1.96
ANKRD20A5P	1710	1.16E-03	5.85E-03	1.96
ST6GALNAC4	2186	2.43E-06	2.70E-05	1.96

ENKD1	523	4.06E-04	2.39E-03	1.96
FOXP3	3087	1.93E-04	1.26E-03	1.96
TBL3	8807	1.75E-10	4.51E-09	1.96
PHLDA1	1669	4.33E-03	1.77E-02	1.97
ALG12	7358	7.85E-11	2.13E-09	1.97
EPN1	1647	8.33E-05	6.08E-04	1.97
NAIP	6105	9.58E-06	9.16E-05	1.97
SLA2	3425	2.22E-09	4.61E-08	1.97
PFAS	2942	1.08E-05	1.02E-04	1.97
PMS2P3	2950	1.32E-08	2.39E-07	1.97
FPGS	7687	1.05E-10	2.79E-09	1.98
HSCB	1210	9.74E-05	6.97E-04	1.98
INTS1	7858	1.22E-04	8.51E-04	1.98
KIAA0895L	782	4.69E-03	1.89E-02	1.98
CHERP	4532	4.78E-07	6.29E-06	1.98
RGL2	9772	8.87E-07	1.10E-05	1.98
TMEM121	648	3.87E-03	1.62E-02	1.98
CAMTA2	6283	1.48E-06	1.74E-05	1.98
CD200R1	5724	5.54E-05	4.28E-04	1.98
CCDC106	233	1.08E-02	3.76E-02	1.98
NCLN	8448	1.70E-09	3.61E-08	1.98
SLC16A3	210	1.22E-02	4.17E-02	1.98
QTRT1	9231	1.78E-10	4.58E-09	1.98
LINC00092	245	1.51E-02	4.91E-02	1.98
WDR6	43026	2.24E-07	3.16E-06	1.98
APBA3	7183	5.49E-09	1.07E-07	1.98
PITPNM1	8722	5.41E-05	4.19E-04	1.98
GPR22	1436	3.30E-03	1.42E-02	1.99
IKBKE	15514	9.26E-09	1.73E-07	1.99
ELK1	2428	1.01E-05	9.63E-05	1.99
PTOV1-AS2	622	1.85E-04	1.22E-03	1.99
PWWP2B	583	2.88E-05	2.42E-04	1.99
RASGRP2	44726	2.32E-10	5.82E-09	1.99
ALDH16A1	3850	1.17E-10	3.11E-09	1.99
FAM86C1	886	9.74E-05	6.97E-04	1.99
PLEKHH2	476	5.34E-03	2.11E-02	1.99
USP5	9150	4.05E-10	9.69E-09	1.99
SHKBP1	20422	1.61E-13	6.62E-12	1.99
IBA57	1783	1.91E-05	1.69E-04	1.99
GNG10	10943	5.28E-09	1.04E-07	1.99
DDA1	5090	5.91E-07	7.61E-06	1.99
PRR5	4171	1.03E-18	7.34E-17	1.99
ASAH2B	758	2.64E-03	1.18E-02	1.99
MIIP	6856	1.10E-12	3.96E-11	1.99
HID1	1360	4.12E-04	2.42E-03	1.99
SPTB	287	1.30E-02	4.38E-02	1.99

RNR2	4650973	2.01E-05	1.77E-04	2.00
PARD6A	1067	8.80E-04	4.65E-03	2.00
IL10RA	93421	5.70E-07	7.38E-06	2.00
DND1	1271	1.42E-04	9.66E-04	2.00
ZNF613	1312	1.31E-03	6.51E-03	2.00
C4orf48	1769	7.41E-04	4.02E-03	2.00
CACTIN	3465	6.53E-07	8.30E-06	2.00
GPR82	984	1.32E-03	6.54E-03	2.00
TEF	1297	2.04E-03	9.48E-03	2.00
TNFRSF25	31118	3.30E-16	1.87E-14	2.00
VAC14	11494	9.79E-14	4.15E-12	2.00
AES	75124	6.83E-12	2.17E-10	2.00
LRFN3	297	7.62E-04	4.12E-03	2.00
IER2	64963	4.64E-04	2.67E-03	2.00
ZNF785	1552	2.66E-05	2.25E-04	2.00
NPDC1	2952	7.99E-06	7.82E-05	2.01
SPCS2	19777	1.37E-09	2.95E-08	2.01
MAN2C1	14797	9.32E-03	3.33E-02	2.01
ACSS1	16938	2.56E-08	4.32E-07	2.01
AGFG2	3536	1.53E-06	1.79E-05	2.01
CLCN7	8862	3.65E-07	4.93E-06	2.01
AGAP7P	1138	2.72E-03	1.20E-02	2.01
ZNF696	1560	1.69E-06	1.95E-05	2.01
MED22	2900	6.70E-11	1.86E-09	2.01
CERS4	1133	1.52E-03	7.39E-03	2.01
GPR68	1414	3.06E-05	2.54E-04	2.01
ANKS3	2230	3.50E-09	7.11E-08	2.01
MINK1	10905	2.61E-05	2.22E-04	2.01
LRRC56	315	5.53E-03	2.17E-02	2.02
KLC4	1719	5.30E-07	6.90E-06	2.02
FASTK	14692	2.85E-12	9.67E-11	2.02
PQLC1	4041	1.14E-06	1.37E-05	2.02
TNFAIP8L2	3909	3.81E-05	3.07E-04	2.02
ST3GAL2	6606	5.19E-07	6.76E-06	2.02
IRAIN	629	1.16E-03	5.85E-03	2.02
HRAS	1548	2.98E-07	4.11E-06	2.02
GGT7	4309	3.77E-10	9.10E-09	2.02
CPNE2	1350	6.26E-10	1.43E-08	2.02
Sep-01	32088	2.77E-14	1.24E-12	2.02
DNASE1	2035	1.28E-07	1.91E-06	2.02
BIK	771	7.34E-04	3.99E-03	2.02
CALHM2	3223	5.05E-04	2.87E-03	2.03
DBP	6607	1.96E-10	4.98E-09	2.03
TSPAN4	617	1.35E-04	9.26E-04	2.03
TEDC1	1679	1.79E-06	2.05E-05	2.03
SPATC1L	1420	2.66E-03	1.18E-02	2.03

CD6	64557	1.27E-11	3.90E-10	2.03
SDHAP2	8788	5.04E-08	8.07E-07	2.03
LIME1	27974	7.34E-11	2.01E-09	2.03
POLR2J3	15161	1.88E-09	3.94E-08	2.03
RABL2B	5355	2.20E-08	3.81E-07	2.04
MC1R	613	7.42E-03	2.76E-02	2.04
HILPDA	600	8.02E-03	2.95E-02	2.04
PIEZO1	16914	4.09E-06	4.30E-05	2.04
PLA2G15	1871	5.81E-06	5.89E-05	2.04
WIZ	952	3.54E-05	2.87E-04	2.04
SPNS1	10844	1.32E-10	3.48E-09	2.04
FLJ42627	1679	1.00E-04	7.15E-04	2.04
PRPF40B	1145	2.85E-04	1.76E-03	2.05
LY6E	25247	2.77E-09	5.68E-08	2.05
EME2	185	7.67E-03	2.84E-02	2.05
MBOAT7	3343	3.57E-09	7.24E-08	2.05
MINOS1-NBL1	521	2.97E-03	1.30E-02	2.05
LLGL2	7182	2.96E-10	7.28E-09	2.05
DPP3	5284	5.70E-08	9.04E-07	2.05
RTCA-AS1	349	3.45E-03	1.47E-02	2.05
TMEM185A	3149	3.21E-07	4.40E-06	2.05
SF3A2	3764	3.14E-11	9.23E-10	2.05
CROCC	4060	9.33E-07	1.15E-05	2.05
PTMS	240	1.47E-02	4.82E-02	2.06
ZNF784	444	2.59E-04	1.63E-03	2.06
EBLN2	2203	1.35E-04	9.24E-04	2.06
CAPN10	5723	2.32E-06	2.59E-05	2.06
DNAJC30	4337	1.51E-08	2.70E-07	2.06
CCDC85B	8192	1.44E-07	2.12E-06	2.06
CD2BP2	11321	6.02E-13	2.25E-11	2.06
MAP1LC3A	493	1.54E-02	4.99E-02	2.06
DNASE2	5819	2.72E-14	1.23E-12	2.07
HDAC5	5027	1.62E-06	1.89E-05	2.07
PPP2R5B	1539	7.16E-05	5.34E-04	2.07
PLCH2	1838	3.90E-04	2.31E-03	2.07
RECQL5	5047	1.39E-09	2.98E-08	2.07
SCAMP4	2144	7.85E-09	1.49E-07	2.07
ARL17A	24426	6.46E-06	6.47E-05	2.07
SPRYD3	8004	1.34E-13	5.56E-12	2.07
RASAL3	28889	7.22E-08	1.13E-06	2.07
PVRIG	10030	2.11E-19	1.59E-17	2.07
RUNDC1	4587	5.32E-11	1.50E-09	2.07
KLC2	1603	8.10E-09	1.53E-07	2.07
LOC100630923	5944	5.09E-04	2.89E-03	2.08
DNAI2	618	1.55E-03	7.51E-03	2.08
GIPC1	3974	2.41E-08	4.11E-07	2.08

TNK1	4297	5.72E-06	5.80E-05	2.08
RSG1	323	3.94E-03	1.64E-02	2.08
ADAM15	3263	1.12E-06	1.35E-05	2.08
RBM14	16116	2.89E-10	7.12E-09	2.08
TBC1D2	1524	1.57E-06	1.83E-05	2.08
THAP8	689	4.02E-05	3.22E-04	2.08
E2F2	551	5.37E-03	2.12E-02	2.08
ST6GALNAC6	14993	1.92E-06	2.18E-05	2.09
EFEMP2	934	2.36E-04	1.50E-03	2.09
MYO6	2578	3.32E-04	2.02E-03	2.09
ZC3H10	691	6.49E-03	2.47E-02	2.09
SPATA41	433	1.12E-02	3.86E-02	2.09
TRAF2	4254	4.80E-10	1.13E-08	2.09
NOXA1	764	2.64E-04	1.65E-03	2.09
KRBA2	2322	2.66E-04	1.66E-03	2.09
RPS10	29042	1.16E-07	1.73E-06	2.09
RPL17	44159	9.13E-10	2.02E-08	2.09
FRG1-DT	513	1.98E-03	9.25E-03	2.09
C19orf44	651	3.79E-04	2.25E-03	2.09
PHF23	1469	2.72E-06	2.98E-05	2.10
MFSD13A	1157	1.40E-05	1.28E-04	2.10
RELT	1838	8.34E-07	1.04E-05	2.10
GTPBP3	2507	3.58E-12	1.19E-10	2.10
TMEM187	1636	1.51E-04	1.02E-03	2.10
BCL2L1	1836	5.37E-05	4.17E-04	2.10
FDXR	813	6.72E-05	5.06E-04	2.10
PLEKHG3	4044	8.84E-06	8.55E-05	2.10
LPCAT4	2860	7.33E-09	1.40E-07	2.10
HOMEZ	787	1.45E-02	4.77E-02	2.11
SIT1	10710	1.72E-09	3.64E-08	2.11
B3GAT3	6695	4.28E-11	1.23E-09	2.11
MVP	35415	6.00E-14	2.62E-12	2.11
ZYX	15016	1.88E-06	2.15E-05	2.11
TBC1D10B	2292	1.06E-08	1.97E-07	2.11
UBE2M	1729	1.01E-06	1.23E-05	2.11
CDK2AP2	12156	2.60E-08	4.37E-07	2.11
PCYT2	7547	4.57E-12	1.50E-10	2.11
DNAJB6	19191	1.59E-31	2.62E-29	2.11
DLG4	204	1.15E-02	3.95E-02	2.11
GTF2H2C_2	3394	8.21E-09	1.55E-07	2.11
FAM173A	1949	3.72E-05	3.00E-04	2.12
LOC202181	6018	9.04E-07	1.12E-05	2.12
SMAD9	790	1.20E-04	8.35E-04	2.12
ORAI2	8985	6.80E-11	1.88E-09	2.12
SELENOO	2897	1.33E-07	1.97E-06	2.12
FANCA	1352	1.31E-06	1.56E-05	2.12

ZNF513	2280	1.14E-08	2.10E-07	2.12
FTH1	158668	3.47E-11	1.01E-09	2.12
LAG3	1161	1.81E-04	1.20E-03	2.12
ARHGEF5	686	1.45E-03	7.08E-03	2.12
PAIP2B	1646	5.12E-07	6.68E-06	2.12
OGFOD2	4305	5.51E-11	1.55E-09	2.12
HYAL2	540	1.07E-03	5.47E-03	2.12
TMC8	110605	2.45E-08	4.17E-07	2.13
CAPN15	3917	9.52E-07	1.17E-05	2.13
ZNF816	2945	6.19E-05	4.72E-04	2.13
ANKRD23	1183	4.03E-05	3.22E-04	2.13
ZBTB7A	8746	6.02E-13	2.25E-11	2.13
TNF	3299	6.48E-04	3.57E-03	2.13
LOC391322	1918	1.18E-03	5.94E-03	2.13
CCDC126	3438	7.72E-06	7.59E-05	2.13
RGS19	7957	1.28E-08	2.32E-07	2.13
TRMT61A	3046	6.74E-09	1.29E-07	2.13
CSF1	1824	2.37E-05	2.05E-04	2.13
MAP3K6	1855	1.73E-05	1.55E-04	2.14
SLC1A5	2143	1.09E-03	5.58E-03	2.14
HDAC11	1102	7.16E-05	5.34E-04	2.14
ARHGEF1	75226	1.02E-07	1.55E-06	2.14
LIPE	744	8.86E-06	8.56E-05	2.14
NUDT3	2701	5.47E-08	8.71E-07	2.14
ZNF692	8336	3.69E-12	1.22E-10	2.14
CDK16	2473	6.50E-09	1.25E-07	2.14
PCSK1N	809	3.23E-03	1.40E-02	2.14
PRDM11	731	5.28E-04	2.99E-03	2.15
SLC2A6	1183	1.15E-03	5.81E-03	2.15
NDUFA6-AS1	801	9.22E-08	1.41E-06	2.15
LZTS2	2267	1.65E-09	3.51E-08	2.15
TFEB	1253	8.12E-04	4.33E-03	2.15
PARGP1	847	4.79E-04	2.74E-03	2.15
ATP13A1	17844	9.27E-08	1.42E-06	2.15
LOC102724814	11554	2.30E-10	5.76E-09	2.15
LINC01355	1098	2.20E-04	1.41E-03	2.15
HPS6	2337	3.34E-10	8.08E-09	2.16
ZC2HC1C	645	8.19E-03	3.00E-02	2.16
ARFGAP1	5130	2.27E-09	4.69E-08	2.16
CRIP2	11719	1.41E-04	9.61E-04	2.16
DOK1	1664	1.06E-07	1.60E-06	2.16
DPY19L2P2	954	1.55E-04	1.05E-03	2.16
CHAC2	1085	1.12E-04	7.90E-04	2.16
TOP3B	7364	1.65E-06	1.92E-05	2.16
GNB1L	1266	2.45E-08	4.17E-07	2.16
COQ8B	2715	2.25E-06	2.51E-05	2.17

HOOK2	876	2.04E-05	1.79E-04	2.17
HAUS5	6905	5.67E-09	1.10E-07	2.17
FAS-AS1	355	6.27E-03	2.41E-02	2.17
TMEM56-RWDD3	321	3.30E-03	1.42E-02	2.17
RAB40C	3430	4.78E-10	1.12E-08	2.17
CCDC120	542	1.38E-04	9.40E-04	2.17
FAM3A	4969	2.10E-14	9.69E-13	2.17
ZFYVE28	4200	1.47E-13	6.09E-12	2.17
EPHB4	576	1.14E-04	8.03E-04	2.17
ABCC10	7370	1.35E-10	3.55E-09	2.17
AMIGO3	637	1.93E-04	1.27E-03	2.17
CITED4	4093	6.72E-07	8.52E-06	2.17
RPS2P32	1092	1.27E-07	1.90E-06	2.17
CD82	16993	4.12E-13	1.59E-11	2.18
STAG3L5P-PVRIG2P-PILRB	25540	1.03E-06	1.25E-05	2.18
RGS14	16384	5.01E-22	4.64E-20	2.18
CCDC22	5128	4.74E-16	2.64E-14	2.18
F2RL2	2077	8.99E-05	6.50E-04	2.18
S100A11	31150	6.14E-06	6.19E-05	2.18
INE1	451	8.36E-04	4.45E-03	2.18
FCHO1	7087	5.03E-08	8.06E-07	2.18
UBA7	27277	5.12E-09	1.01E-07	2.18
LTBP3	11194	6.45E-10	1.47E-08	2.19
PGAP3	6490	1.17E-09	2.55E-08	2.19
PRR7	1344	9.09E-14	3.88E-12	2.19
NUTM2G	568	1.00E-03	5.18E-03	2.19
GPR157	224	5.93E-03	2.30E-02	2.19
RGS16	453	1.84E-03	8.69E-03	2.19
LOC100996741	1123	3.98E-05	3.19E-04	2.19
SMG9	2340	4.08E-09	8.15E-08	2.19
ANO9	12198	1.26E-14	6.04E-13	2.19
ARHGAP27	15498	5.18E-13	1.98E-11	2.20
ATXN7L2	1993	7.12E-08	1.12E-06	2.20
PDLIM2	17462	7.47E-16	4.07E-14	2.20
MUS81	7446	2.16E-12	7.50E-11	2.20
ZNF444	2286	1.62E-08	2.88E-07	2.20
PIDD1	4415	2.45E-08	4.17E-07	2.20
LOC100506990	4013	1.19E-09	2.59E-08	2.20
TREML2	862	7.11E-04	3.87E-03	2.20
ALS2CL	11228	2.42E-05	2.08E-04	2.21
MROH6	206	3.87E-03	1.62E-02	2.21
APTR	2852	6.09E-10	1.40E-08	2.21
NAGPA-AS1	387	7.06E-03	2.65E-02	2.21
POLM	5006	2.82E-12	9.60E-11	2.21
LOC100507551	437	5.54E-05	4.28E-04	2.21

LOC100288778	2349	1.11E-05	1.05E-04	2.21
RASSF1-AS1	671	3.05E-05	2.54E-04	2.21
TOM1	7642	1.54E-16	9.22E-15	2.22
DTX2	3057	4.68E-10	1.10E-08	2.22
CD7	14133	6.02E-13	2.25E-11	2.22
TNFRSF18	1586	1.35E-04	9.24E-04	2.22
NUMBL	733	3.03E-06	3.27E-05	2.23
SREBF1	7432	2.00E-06	2.28E-05	2.23
ATP13A2	9029	1.76E-13	7.14E-12	2.23
TEPSIN	3178	8.31E-08	1.28E-06	2.23
LZTR1	9540	3.88E-08	6.34E-07	2.23
GLIDR	777	4.92E-04	2.81E-03	2.23
RARA	2105	1.28E-05	1.18E-04	2.23
TVP23B	9358	6.97E-10	1.58E-08	2.23
FUZ	683	4.44E-04	2.58E-03	2.23
IGHV1OR15-1	5084	4.95E-05	3.87E-04	2.24
LOC102724760	5084	4.95E-05	3.87E-04	2.24
MRM1	1079	6.79E-06	6.76E-05	2.24
TGFB1	10469	8.59E-09	1.62E-07	2.24
SCO2	876	9.35E-03	3.34E-02	2.24
ZNF786	3415	1.54E-08	2.75E-07	2.24
KCNC4	682	1.26E-04	8.71E-04	2.24
PSD4	8565	4.95E-08	7.97E-07	2.24
ZNF668	1290	1.18E-05	1.10E-04	2.25
RAP1B	71669	2.13E-16	1.26E-14	2.25
RLN3	229	4.32E-04	2.52E-03	2.25
ZBTB42	399	1.48E-02	4.84E-02	2.25
FCHSD1	6058	8.64E-09	1.62E-07	2.25
SLX1A-SULT1A3	3137	2.23E-08	3.84E-07	2.25
SLX1B-SULT1A4	3137	2.23E-08	3.84E-07	2.25
ZNF213	516	1.63E-03	7.81E-03	2.25
CEP131	1255	7.57E-07	9.48E-06	2.26
PFKL	24660	5.82E-12	1.87E-10	2.26
TBC1D10C	68072	4.21E-16	2.36E-14	2.26
TYK2	20316	2.14E-10	5.40E-09	2.26
DBNDD1	1032	2.65E-05	2.24E-04	2.26
DVL2	3219	2.54E-07	3.55E-06	2.27
LRP10	24814	1.50E-15	7.93E-14	2.27
FAM234A	5647	2.54E-15	1.31E-13	2.27
GHDC	2604	1.54E-14	7.30E-13	2.27
APBB3	5363	6.02E-09	1.16E-07	2.27
RAD9A	1755	5.85E-12	1.88E-10	2.27
SH3GL1	4386	1.47E-10	3.83E-09	2.27
SAMD4B	3843	2.15E-07	3.05E-06	2.28
POLRMT	8077	1.43E-12	5.06E-11	2.28
RNPEPL1	17665	6.12E-09	1.18E-07	2.28

BANF1	8034	1.48E-18	1.04E-16	2.28
PNPLA6	3386	3.24E-09	6.60E-08	2.28
ARHGAP27P1	1134	2.08E-06	2.35E-05	2.28
BRICD5	327	3.30E-03	1.42E-02	2.28
HAGHL	494	2.08E-04	1.35E-03	2.28
NOTCH2NL	20231	8.63E-07	1.07E-05	2.29
GPSM1	513	4.69E-03	1.89E-02	2.29
SH3GL1P1	434	2.32E-04	1.48E-03	2.29
FRMD6-AS1	241	8.06E-03	2.96E-02	2.29
LIN7C	11054	7.78E-11	2.12E-09	2.29
CCR8	1777	4.60E-03	1.87E-02	2.29
FBXW5	25225	7.88E-13	2.89E-11	2.29
MAPRE3	322	3.88E-03	1.62E-02	2.30
SSSCA1-AS1	660	1.37E-04	9.38E-04	2.30
SRGAP2B	3640	3.14E-10	7.66E-09	2.30
ZNF66	3681	8.13E-06	7.94E-05	2.30
JMJD7-PLA2G4B	2251	5.51E-06	5.59E-05	2.30
FAM213B	3669	5.48E-09	1.07E-07	2.30
ZBTB22	1964	1.22E-06	1.46E-05	2.30
ITGB2-AS1	28911	2.25E-11	6.74E-10	2.30
LOC729732	398	7.92E-04	4.25E-03	2.30
CENPT	19533	8.91E-13	3.24E-11	2.30
LOXL1	1378	6.59E-07	8.37E-06	2.30
PPP1R3F	1898	8.10E-09	1.53E-07	2.30
ADCK5	2788	2.64E-10	6.58E-09	2.30
SPPL2B	5708	1.15E-14	5.52E-13	2.31
HIST2H2BC	260	3.46E-04	2.08E-03	2.31
LOC100506023	3297	1.99E-10	5.05E-09	2.31
AEBP1	2306	1.44E-06	1.70E-05	2.31
PIGQ	3650	4.34E-09	8.63E-08	2.31
JUNB	171950	9.92E-04	5.14E-03	2.31
MAP3K10	279	4.64E-04	2.67E-03	2.31
WDR90	1195	1.42E-05	1.29E-04	2.31
NEK8	1987	7.95E-08	1.23E-06	2.31
PKD1P5-	1782	1.09E-04	7.69E-04	2.31
LTB	140580	3.93E-19	2.89E-17	2.32
PKD1P3-NPIPA1	6461	3.78E-06	4.01E-05	2.32
C16orf58	9453	1.22E-14	5.85E-13	2.32
IL27RA	19777	7.09E-22	6.46E-20	2.32
ZNF28	2971	2.10E-07	2.98E-06	2.33
KLHL17	1992	5.27E-10	1.22E-08	2.33
INO80B	1165	2.28E-05	1.98E-04	2.34
SLC10A3	5974	1.39E-15	7.38E-14	2.34
ATAD3A	2290	2.26E-08	3.89E-07	2.34
NFKB2	5560	2.67E-16	1.56E-14	2.34
AK1	3103	9.58E-07	1.18E-05	2.34

SAMD10	2801	4.70E-08	7.60E-07	2.34
TRADD	24129	3.40E-10	8.21E-09	2.35
RTL8C	3637	1.49E-23	1.53E-21	2.35
CYP3A5	491	1.57E-05	1.42E-04	2.35
PEX6	4267	4.65E-03	1.88E-02	2.35
LRFN1	880	3.02E-04	1.85E-03	2.35
FRMD5	970	5.45E-05	4.22E-04	2.35
AIDA	5400	3.06E-20	2.45E-18	2.35
SGSM2	15414	8.41E-07	1.05E-05	2.35
FAM43A	1623	5.21E-06	5.32E-05	2.35
IFNG-AS1	1705	7.71E-04	4.16E-03	2.35
SH3TC1	3591	2.92E-06	3.16E-05	2.36
GASAL1	305	5.66E-04	3.17E-03	2.36
FAM207A	466	7.07E-06	7.02E-05	2.36
RPUSD1	4718	1.65E-13	6.75E-12	2.36
GDI1	12569	1.56E-10	4.07E-09	2.36
PPIA	91607	1.57E-26	1.97E-24	2.36
MFSD10	8805	4.35E-13	1.68E-11	2.36
MIB2	5162	3.22E-12	1.08E-10	2.36
ZNF853	778	8.69E-04	4.60E-03	2.37
PLEKHF1	2854	1.77E-06	2.04E-05	2.37
TMC6	44730	5.56E-16	3.07E-14	2.37
PABPN1	7669	4.68E-14	2.07E-12	2.37
KCNJ13	752	7.08E-05	5.29E-04	2.37
IDUA	4331	2.84E-16	1.65E-14	2.37
TMEM150C	278	1.46E-02	4.80E-02	2.37
TMEM201	725	3.07E-05	2.55E-04	2.37
DDX11L2	501	9.26E-03	3.32E-02	2.38
C21orf59-TCP10L	3392	7.94E-05	5.84E-04	2.38
E2F6	1200	1.49E-08	2.68E-07	2.38
HMGN1	47934	4.40E-28	6.10E-26	2.38
ZDHHC8	2056	1.61E-09	3.42E-08	2.38
TUBGCP6	24720	4.04E-09	8.11E-08	2.38
SMG1P3	32604	3.06E-06	3.30E-05	2.38
LOC100130992	830	2.94E-05	2.46E-04	2.38
BMS1P2	4155	2.21E-09	4.59E-08	2.38
MIF-AS1	13148	4.21E-09	8.39E-08	2.38
NOS3	1729	1.24E-15	6.69E-14	2.38
ZNF252P-AS1	302	1.30E-02	4.37E-02	2.39
STX1A	364	3.04E-03	1.33E-02	2.39
RPL23A	153915	6.59E-16	3.61E-14	2.39
LOC101927124	414	7.38E-03	2.75E-02	2.39
PPP1R15A	13958	7.40E-08	1.15E-06	2.39
FIZ1	718	2.20E-07	3.11E-06	2.39
ATP6V0E2-AS1	984	4.59E-05	3.62E-04	2.40
PTGES2	7887	8.20E-21	6.77E-19	2.40

NRAV	203	1.28E-02	4.31E-02	2.40
KCNH3	472	1.06E-02	3.69E-02	2.40
FBXO44	5642	1.09E-09	2.39E-08	2.41
PML	7748	5.17E-11	1.46E-09	2.41
RRP7BP	8346	5.66E-13	2.14E-11	2.41
SGSM3	29219	4.33E-11	1.24E-09	2.41
MROH1	6848	1.95E-12	6.83E-11	2.41
ARHGAP4	35451	5.50E-17	3.46E-15	2.41
PLA2G6	3408	2.42E-11	7.21E-10	2.42
SPINT1	965	6.32E-04	3.49E-03	2.42
RIN1	788	1.37E-04	9.38E-04	2.42
ZNF710	762	2.70E-06	2.96E-05	2.42
ATP2A3	20470	1.33E-07	1.98E-06	2.42
FZR1	4073	3.32E-11	9.71E-10	2.42
ZGLP1	420	7.81E-04	4.20E-03	2.42
FXYD7	840	2.28E-05	1.98E-04	2.43
TMEM175	12629	2.93E-16	1.68E-14	2.43
USP20	15133	6.33E-08	9.98E-07	2.43
CLASRP	3579	3.94E-09	7.91E-08	2.43
LINC00891	563	3.80E-04	2.26E-03	2.43
LOC100132741	563	3.80E-04	2.26E-03	2.43
KCTD7	10252	1.42E-10	3.70E-09	2.43
CORO6	380	3.09E-04	1.89E-03	2.44
B3GNT9	863	3.57E-04	2.14E-03	2.44
LOC388692	1107	4.01E-06	4.23E-05	2.44
TFE3	2352	2.33E-12	8.03E-11	2.44
CCDC9B	233	7.72E-03	2.86E-02	2.44
NDOR1	3951	2.80E-10	6.92E-09	2.44
C19orf25	4148	2.71E-17	1.75E-15	2.44
SPHK2	2093	1.30E-10	3.42E-09	2.44
STOML1	1589	4.83E-08	7.79E-07	2.44
RAB11B	9682	3.03E-11	8.93E-10	2.45
TMEM191B	555	4.21E-03	1.74E-02	2.45
CORO1B	5136	9.52E-11	2.54E-09	2.45
TPBG	341	6.79E-03	2.57E-02	2.45
ILVBL	3730	2.43E-17	1.57E-15	2.45
FIGNL2	96	1.41E-02	4.66E-02	2.46
TRIM56	12928	8.74E-17	5.35E-15	2.46
ZBED3	589	4.05E-03	1.68E-02	2.46
PLEKHA4	231	6.07E-04	3.37E-03	2.46
ZFPM1	369	9.38E-05	6.74E-04	2.46
LRCH4	30109	1.07E-14	5.20E-13	2.47
PDZD7	176	1.37E-02	4.56E-02	2.47
PYGO2	5813	1.82E-10	4.67E-09	2.47
ENGASE	9907	2.49E-08	4.22E-07	2.47
PDCD1	1748	2.64E-06	2.90E-05	2.47

GPSM3	18285	7.16E-10	1.62E-08	2.47
SCAF1	434	7.96E-05	5.84E-04	2.47
C1D	6922	1.03E-03	5.32E-03	2.48
ZNF208	4344	5.15E-03	2.04E-02	2.48
MAP3K11	5470	1.54E-11	4.71E-10	2.48
HCFC1R1	1513	2.74E-10	6.78E-09	2.48
ZNF547	329	3.24E-03	1.40E-02	2.48
LOC729737	1155	5.89E-04	3.28E-03	2.48
OLFM2	493	3.38E-03	1.45E-02	2.48
TERF1	6965	1.66E-36	3.62E-34	2.48
MFSD12	7459	1.63E-12	5.74E-11	2.48
WNT1	530	7.68E-04	4.15E-03	2.49
TTC22	781	1.72E-05	1.54E-04	2.49
SPDYE17	241	4.28E-04	2.50E-03	2.49
LOC729218	2670	4.04E-12	1.33E-10	2.49
KCTD13	3943	7.52E-15	3.75E-13	2.49
GLI4	2465	9.09E-12	2.84E-10	2.49
RPL23AP7	1606	4.99E-10	1.16E-08	2.49
FLYWCH1	8979	2.84E-08	4.76E-07	2.49
LPAR6	28703	1.05E-07	1.59E-06	2.50
ZNF837	1550	4.32E-06	4.52E-05	2.50
SAP25	2703	1.79E-13	7.22E-12	2.50
RENBP	382	2.29E-05	1.99E-04	2.51
MTX1	2463	5.27E-17	3.33E-15	2.51
CACNA1H	544	2.07E-04	1.35E-03	2.51
NLRP6	1756	5.85E-05	4.48E-04	2.51
NARFL	3345	2.02E-10	5.12E-09	2.51
FAM133B	3310	3.36E-08	5.56E-07	2.51
CD151	1584	3.56E-06	3.79E-05	2.51
FADS3	784	2.09E-04	1.35E-03	2.52
FOS	116920	6.37E-03	2.44E-02	2.52
LOC101929165	632	1.87E-04	1.23E-03	2.52
LIMD2	62956	1.29E-19	9.88E-18	2.52
ARHGDIA	15141	2.46E-14	1.12E-12	2.53
ATAD3B	5458	4.15E-10	9.90E-09	2.54
LOC100132215	210	3.06E-03	1.33E-02	2.54
GGT1	1303	7.05E-06	7.00E-05	2.55
SH2D3A	11710	3.40E-21	2.91E-19	2.55
C15orf53	710	3.70E-03	1.56E-02	2.55
CORO7	36519	2.72E-13	1.07E-11	2.55
ANKRD18CP	628	3.18E-04	1.94E-03	2.55
KCNH2	728	1.57E-06	1.83E-05	2.55
POM121L10P	136	1.13E-02	3.90E-02	2.55
CDK10	14663	1.71E-17	1.11E-15	2.56
OGFR	8539	5.03E-16	2.78E-14	2.56
MAMDC4	288	2.02E-03	9.37E-03	2.56

NCDN	4431	9.72E-18	6.42E-16	2.56
C1orf159	2437	2.72E-10	6.74E-09	2.56
GDPD5	5785	1.21E-06	1.44E-05	2.56
C18orf32	13928	4.75E-13	1.82E-11	2.56
POLD1	4987	7.69E-10	1.73E-08	2.56
CC2D1A	3810	4.25E-10	1.01E-08	2.56
CNOT3	2244	2.51E-08	4.25E-07	2.56
ADORA2A	4522	6.95E-05	5.22E-04	2.56
BAK1	5793	5.45E-11	1.53E-09	2.56
CAPS	3406	1.53E-14	7.24E-13	2.57
ZNF404	734	2.75E-04	1.71E-03	2.57
TAF1C	9589	5.44E-19	3.98E-17	2.57
RPS6KA4	2123	8.57E-09	1.61E-07	2.57
MICALL2	233	2.80E-03	1.24E-02	2.57
BBC3	1029	2.87E-08	4.80E-07	2.57
TRIM47	1279	2.09E-06	2.36E-05	2.57
PKN1	11705	3.43E-11	1.00E-09	2.57
SH2D3C	9421	2.49E-22	2.36E-20	2.58
USP35	555	2.82E-04	1.75E-03	2.58
GNB2	3968	5.92E-10	1.36E-08	2.58
PLSCR3	19405	2.49E-31	4.04E-29	2.58
CASP1P2	1858	1.51E-03	7.34E-03	2.59
B3GNT8	348	8.05E-04	4.31E-03	2.59
FAM157B	1144	1.68E-07	2.42E-06	2.59
SLC27A1	1403	5.03E-12	1.64E-10	2.59
ZDHHC11B	1859	1.82E-04	1.20E-03	2.59
KRTAP16-1	252	6.00E-03	2.32E-02	2.59
LOC101409256	122	1.50E-02	4.90E-02	2.59
INPP5E	3547	5.03E-15	2.53E-13	2.59
ARHGEF4	818	3.43E-06	3.66E-05	2.59
LYL1	812	1.94E-06	2.21E-05	2.60
TMEM229B	2904	2.93E-10	7.20E-09	2.60
HGH1	2660	1.46E-16	8.79E-15	2.60
RPS2	202248	6.51E-26	7.80E-24	2.60
KIFC2	3891	1.69E-13	6.89E-12	2.60
ISY1-RAB43	4404	6.63E-11	1.84E-09	2.60
UC.134	288	3.70E-03	1.56E-02	2.61
LOC100288162	319	1.23E-02	4.18E-02	2.61
MYO5B	540	2.83E-03	1.25E-02	2.61
SLC7A5P2	8315	2.68E-16	1.56E-14	2.61
C7orf43	2291	8.28E-13	3.03E-11	2.61
TONSL	587	1.19E-02	4.07E-02	2.62
ZNF775	649	3.16E-05	2.61E-04	2.62
SLC35C1	3259	1.18E-12	4.21E-11	2.62
SCARF2	309	1.24E-03	6.21E-03	2.63
KLHL25	608	5.16E-06	5.29E-05	2.63

NEFM	360	2.17E-03	9.99E-03	2.63
C2CD4D	305	5.47E-04	3.07E-03	2.63
INTS5	3561	2.54E-07	3.54E-06	2.63
NAPRT	8169	5.20E-10	1.21E-08	2.63
UNC13D	16425	4.67E-13	1.80E-11	2.63
GOLGA6L9	1819	3.12E-07	4.28E-06	2.63
FBXL16	4429	1.90E-22	1.82E-20	2.64
KCNK6	3397	2.83E-12	9.62E-11	2.64
NFKBID	2355	1.92E-14	8.92E-13	2.64
TBC1D8B	416	1.26E-02	4.26E-02	2.64
LPAL2	269	1.50E-02	4.90E-02	2.64
SLC29A2	2017	6.74E-09	1.29E-07	2.65
DTX3	2597	1.83E-08	3.20E-07	2.65
COL6A2	5126	8.68E-11	2.34E-09	2.65
PRICKLE3	779	1.13E-06	1.36E-05	2.65
LINC01547	418	1.42E-04	9.68E-04	2.65
FSTL3	572	2.25E-03	1.03E-02	2.66
RECQL4	728	1.19E-03	5.99E-03	2.66
CEP19	916	2.79E-11	8.26E-10	2.66
ABCA7	18266	3.76E-08	6.17E-07	2.66
POU5F1P4	237	2.47E-03	1.11E-02	2.66
ZNF324	7945	4.77E-12	1.56E-10	2.67
LRFN4	448	9.64E-04	5.02E-03	2.67
WNT10A	1162	2.23E-08	3.84E-07	2.67
ZNF575	645	7.01E-08	1.10E-06	2.67
CYB561	7710	1.78E-09	3.75E-08	2.67
HERC2P7	1269	5.79E-06	5.87E-05	2.68
FBXL6	3639	4.93E-12	1.61E-10	2.68
FAAP100	5824	1.80E-18	1.25E-16	2.68
JOSD2	1475	1.96E-11	5.90E-10	2.68
RAB1B	2667	1.69E-13	6.89E-12	2.69
TMEM8A	12769	2.89E-08	4.83E-07	2.69
LMF1-AS1	756	6.19E-07	7.90E-06	2.69
CCDC61	1496	3.17E-13	1.24E-11	2.69
RHPN1	3937	8.49E-12	2.67E-10	2.70
KLF16	434	1.76E-05	1.57E-04	2.70
ZNF341	1095	7.96E-10	1.79E-08	2.70
FOXD2-AS1	271	1.62E-03	7.78E-03	2.71
EVI5L	877	1.21E-05	1.12E-04	2.71
MIER2	963	1.17E-08	2.15E-07	2.71
ARHGAP45	152797	2.88E-12	9.74E-11	2.71
AP5Z1	8243	3.34E-27	4.36E-25	2.72
TMEM129	8359	3.35E-18	2.28E-16	2.72
TBC1D3F	197	5.27E-05	4.10E-04	2.72
GPR25	669	2.41E-03	1.09E-02	2.72
SLC38A10	18769	1.25E-19	9.63E-18	2.72

ADAMTS10	1886	4.16E-07	5.57E-06	2.72
NPIPB5	13121	3.39E-12	1.14E-10	2.72
RASSF7	12068	5.53E-29	8.20E-27	2.73
SAC3D1	855	3.18E-11	9.32E-10	2.73
CEACAM1	1607	2.06E-05	1.80E-04	2.73
IRF7	13135	6.46E-10	1.47E-08	2.74
IL4I1	330	1.40E-02	4.63E-02	2.74
SLC12A7	6037	7.49E-07	9.40E-06	2.75
EIF4E	11961	3.73E-61	2.25E-58	2.75
ZAP70	45342	2.22E-12	7.68E-11	2.75
ZNF414	2505	4.00E-13	1.55E-11	2.76
TRMT2A	9930	2.88E-19	2.13E-17	2.76
LTBP4	7668	7.03E-14	3.06E-12	2.76
PCBP4	2151	8.49E-10	1.89E-08	2.76
B2M	3222748	3.01E-27	3.96E-25	2.77
PPP1R16A	1514	1.38E-10	3.62E-09	2.77
TMA7	44776	6.09E-13	2.27E-11	2.77
QRICH2	517	8.63E-05	6.27E-04	2.77
BRSK2	178	8.63E-04	4.57E-03	2.78
LOC100996385	204	7.88E-03	2.90E-02	2.78
ADAMTS4	204	3.38E-03	1.45E-02	2.78
FKBP8	13175	7.39E-13	2.73E-11	2.79
DOHH	1197	2.71E-10	6.73E-09	2.79
CLSTN3	1388	4.80E-08	7.76E-07	2.79
ABCB9	561	1.86E-04	1.23E-03	2.79
PLEKHG4	3987	5.87E-09	1.14E-07	2.80
SRGAP2C	2778	2.31E-25	2.70E-23	2.80
EML3	6278	8.37E-11	2.26E-09	2.80
TBXA2R	346	1.82E-03	8.61E-03	2.80
SIPA1	18903	1.88E-16	1.11E-14	2.81
VWDE	1140	1.25E-02	4.25E-02	2.81
FNTB	445	5.40E-05	4.19E-04	2.81
AMDHD2	3301	6.43E-22	5.89E-20	2.81
RNASEK-C17orf49	441	1.06E-02	3.71E-02	2.81
TLNRD1	1699	7.00E-12	2.22E-10	2.81
ZC3H3	4338	2.09E-10	5.27E-09	2.82
MIGA2	7544	2.41E-15	1.25E-13	2.82
PRSS53	361	9.63E-07	1.18E-05	2.82
C2CD2L	3142	3.13E-10	7.64E-09	2.82
TMEM259	48312	7.03E-18	4.71E-16	2.82
TNFRSF14-AS1	1745	1.83E-16	1.09E-14	2.82
GSDMD	13941	1.12E-12	4.02E-11	2.83
TOB2	5113	6.38E-17	3.96E-15	2.83
MPV17L2	1099	3.27E-10	7.93E-09	2.83
RHOT2	39422	6.94E-19	5.01E-17	2.83
SPSB2	1896	5.74E-07	7.41E-06	2.83
RAVER1	4237	6.58E-12	2.10E-10	2.83

ACADS	3392	1.17E-21	1.02E-19	2.84
LINC00685	1435	7.90E-10	1.77E-08	2.84
TOR4A	199	4.57E-03	1.86E-02	2.84
NSMF	5219	3.05E-07	4.20E-06	2.85
FAM133DP	3658	3.54E-11	1.03E-09	2.85
CCDC88B	16407	1.11E-10	2.96E-09	2.85
ITPK1-AS1	1055	3.55E-05	2.88E-04	2.85
SSH3	955	5.50E-12	1.78E-10	2.86
PC	685	1.20E-05	1.12E-04	2.86
SLC22A17	2193	2.32E-13	9.26E-12	2.86
PLCB3	750	5.92E-07	7.62E-06	2.86
DYRK1B	1266	5.30E-11	1.50E-09	2.86
S1PR4	46336	7.97E-45	2.45E-42	2.87
FBXL19-AS1	592	1.12E-05	1.05E-04	2.87
MIR133A1HG	351	6.12E-03	2.36E-02	2.87
GPR152	694	3.64E-04	2.18E-03	2.87
FKBPL	227	8.09E-03	2.97E-02	2.88
LRRC14	11622	1.52E-15	7.98E-14	2.88
MAMLD1	561	6.96E-03	2.62E-02	2.88
SNRPG	12236	2.43E-18	1.68E-16	2.88
C9orf139	322	4.54E-03	1.84E-02	2.89
AMN	250	2.36E-04	1.50E-03	2.89
SIRPG-AS1	1839	6.18E-03	2.38E-02	2.89
STAG3L3	13292	4.82E-65	3.25E-62	2.89
LMNTD2	891	1.81E-08	3.18E-07	2.90
TPPP	944	2.28E-03	1.04E-02	2.90
HSF4	652	8.85E-06	8.55E-05	2.90
CXCR3	8466	1.08E-07	1.62E-06	2.90
ZNF653	358	5.15E-06	5.27E-05	2.90
PI16	5464	1.87E-08	3.28E-07	2.91
PYCR3	1326	1.54E-11	4.71E-10	2.91
LRRC45	2384	2.85E-12	9.67E-11	2.91
ARRDC1	11856	6.70E-36	1.38E-33	2.91
ACAP3	4171	1.72E-09	3.64E-08	2.91
RABEP2	1265	2.67E-13	1.06E-11	2.91
CCDC142	2538	5.77E-14	2.54E-12	2.91
LMTK3	135	1.01E-03	5.24E-03	2.91
RARG	2454	4.33E-11	1.24E-09	2.91
PLEKHO2	1937	1.39E-08	2.52E-07	2.92
FBXL8	2494	2.69E-13	1.06E-11	2.92
LRWD1	2205	1.26E-20	1.03E-18	2.92
RPL36A	23547	4.80E-12	1.57E-10	2.92
SOCS1	3623	1.19E-08	2.19E-07	2.93
PGGHG	58226	3.63E-12	1.21E-10	2.93
STMN3	19590	2.40E-24	2.70E-22	2.93
SH3RF3-AS1	355	2.50E-05	2.14E-04	2.93

LOC105370333	245	2.32E-04	1.48E-03	2.93
ZFP41	3616	2.08E-14	9.59E-13	2.93
ABHD16B	589	1.80E-06	2.07E-05	2.94
NR1H2	12780	3.26E-25	3.77E-23	2.95
CYP24A1	273	6.06E-03	2.34E-02	2.95
IGSF8	7773	2.18E-12	7.55E-11	2.95
SSBP4	8178	4.77E-13	1.82E-11	2.95
SOAT2	219	1.30E-03	6.44E-03	2.95
RASAL1	174	1.15E-02	3.96E-02	2.96
CCDC114	231	3.37E-03	1.45E-02	2.96
MUC1	262	3.39E-03	1.45E-02	2.96
AGAP4	2062	6.98E-11	1.92E-09	2.96
B3GNT4	270	1.23E-03	6.16E-03	2.96
DUSP19	371	3.34E-03	1.43E-02	2.97
CHTF18	2538	6.08E-14	2.65E-12	2.98
ZNF526	1972	4.88E-10	1.14E-08	2.98
CACNB1	1270	2.00E-11	5.99E-10	2.98
SLC52A2	4931	3.86E-19	2.85E-17	2.98
WASH7P	3258	4.71E-17	2.99E-15	2.99
PRKAR2A-AS1	325	2.11E-03	9.75E-03	3.00
KMT5C	568	6.17E-07	7.90E-06	3.01
SIPA1L3	3091	3.64E-14	1.61E-12	3.01
PRRT2	1220	1.21E-13	5.05E-12	3.02
LMF2	10406	9.44E-17	5.75E-15	3.02
PDLIM7	621	1.03E-08	1.91E-07	3.02
ZBTB45	974	8.84E-08	1.36E-06	3.03
LOC100288203	8089	2.05E-29	3.10E-27	3.03
DMTN	1957	5.59E-09	1.09E-07	3.03
ADAM8	20806	4.24E-11	1.22E-09	3.03
LINC00260	585	5.15E-07	6.72E-06	3.03
FAM218A	921	2.53E-04	1.60E-03	3.04
C9orf66	1039	4.04E-06	4.25E-05	3.04
KCNN4	4547	8.95E-13	3.25E-11	3.04
ABCB8	3635	5.81E-18	3.91E-16	3.04
RPA4	253	2.88E-04	1.78E-03	3.05
ADAT3	446	6.15E-06	6.19E-05	3.05
HDAC10	3187	6.16E-12	1.97E-10	3.05
DGKZ	11543	9.49E-22	8.52E-20	3.06
KISS1R	232	1.66E-04	1.11E-03	3.06
UBALD1	562	5.46E-09	1.07E-07	3.06
FRS3	610	2.35E-07	3.30E-06	3.07
MAP1S	3948	2.78E-13	1.09E-11	3.07
ACP5	1211	3.52E-08	5.79E-07	3.07
GOLGA6L5P	503	1.36E-05	1.25E-04	3.08
SNAI3	1595	5.83E-07	7.52E-06	3.08
SLC9A3	970	4.97E-08	7.99E-07	3.08

SPDYE10P	166	1.50E-03	7.30E-03	3.08
HYAL1	198	9.23E-04	4.84E-03	3.08
REXO1	3347	8.24E-13	3.02E-11	3.09
UAP1L1	1115	3.39E-09	6.88E-08	3.09
SLC22A31	141	8.20E-03	3.00E-02	3.10
LINC00824	546	1.34E-05	1.23E-04	3.10
FKBP9	151	6.12E-04	3.40E-03	3.11
SLC35E4	691	2.63E-06	2.90E-05	3.11
TREX2	244	1.36E-03	6.70E-03	3.11
DNM1P46	465	1.36E-08	2.47E-07	3.11
IRF2BP1	1319	1.68E-10	4.35E-09	3.12
SYNE4	390	3.30E-07	4.49E-06	3.12
LTB4R2	517	7.43E-12	2.35E-10	3.14
TELO2	5935	8.42E-13	3.08E-11	3.15
ACE	1647	1.49E-08	2.68E-07	3.15
ESRRA	1584	6.66E-16	3.64E-14	3.15
LOC100190986	19216	9.48E-13	3.43E-11	3.15
PIANP	274	4.23E-04	2.47E-03	3.16
PPAN	3671	1.18E-20	9.65E-19	3.16
LOC100129917	4179	2.35E-08	4.01E-07	3.16
TNFSF14	1387	3.13E-06	3.37E-05	3.17
SEMA6C	278	1.94E-04	1.27E-03	3.17
CA5BP1	1395	2.12E-12	7.37E-11	3.17
ANKRD13B	99	1.00E-02	3.53E-02	3.18
RELL2	3687	1.21E-16	7.33E-15	3.19
TRIM46	1096	6.35E-12	2.03E-10	3.20
PDGFB	1218	1.64E-05	1.47E-04	3.21
TMEM79	2075	1.02E-15	5.54E-14	3.22
CATSPER2P1	975	1.94E-09	4.05E-08	3.23
DAPK3	2584	1.08E-11	3.36E-10	3.23
PRRT3	2340	3.22E-15	1.65E-13	3.24
LINC00957	1811	7.74E-18	5.15E-16	3.24
PGAM1	14165	5.61E-30	8.61E-28	3.24
TCIRG1	48612	1.74E-20	1.40E-18	3.24
METRN	923	2.67E-14	1.21E-12	3.24
LINC00537	1153	1.62E-07	2.34E-06	3.25
ZBTB7B	1785	1.55E-11	4.72E-10	3.26
PRR22	182	4.18E-05	3.32E-04	3.26
CARMIL2	13090	3.77E-11	1.09E-09	3.26
ARMC5	2530	7.75E-20	6.10E-18	3.27
SGK3	3888	3.58E-12	1.19E-10	3.27
ZSWIM4	332	3.48E-04	2.10E-03	3.27
IFFO1	12500	8.77E-25	1.00E-22	3.28
LMNA	6391	1.96E-09	4.09E-08	3.28
TPGS1	2932	1.98E-19	1.50E-17	3.29
MMP25	1109	1.06E-13	4.48E-12	3.30

PMS2P9	650	2.88E-18	1.97E-16	3.31
FASLG	379	3.87E-03	1.62E-02	3.31
ACTL10	548	1.38E-05	1.26E-04	3.33
FAM226A	171	1.99E-04	1.30E-03	3.36
FAM226B	171	1.99E-04	1.30E-03	3.36
LOC389199	149	1.04E-03	5.34E-03	3.36
DACT1	2363	2.95E-03	1.30E-02	3.36
RTN4R	350	4.83E-05	3.78E-04	3.36
FAM103A1	5965	2.58E-27	3.45E-25	3.37
TNFRSF4	4932	2.28E-14	1.04E-12	3.37
UPK3B	360	3.45E-06	3.68E-05	3.37
RNF207	791	5.46E-06	5.55E-05	3.37
MXRA8	193	4.28E-04	2.50E-03	3.37
TRABD	30888	4.60E-24	4.97E-22	3.37
TMCC2	210	1.22E-03	6.12E-03	3.39
LOC100506472	337	4.29E-05	3.40E-04	3.40
MAMSTR	435	4.31E-09	8.57E-08	3.40
AIRE	146	4.87E-03	1.95E-02	3.44
SOWAHD	274	9.16E-06	8.80E-05	3.46
LOC100506551	1026	5.14E-10	1.20E-08	3.47
PAK4	389	4.26E-07	5.69E-06	3.47
UBTD1	279	8.98E-06	8.65E-05	3.49
RPS27	180961	6.07E-21	5.05E-19	3.50
BOP1	4871	6.49E-38	1.49E-35	3.53
LOC400927	241	1.15E-04	8.07E-04	3.54
RPL9	61595	1.51E-21	1.31E-19	3.54
CLEC18A	196	8.21E-05	6.00E-04	3.54
F12	122	3.57E-04	2.14E-03	3.55
SLC26A1	1095	8.81E-10	1.95E-08	3.61
LHFPL4	311	1.27E-04	8.75E-04	3.61
CKS1B	2240	3.29E-23	3.29E-21	3.62
WDR24	3059	1.16E-21	1.02E-19	3.62
RAPGEF3	125	3.99E-03	1.66E-02	3.62
SLC9A3R2	124	1.13E-02	3.92E-02	3.65
CHPF	993	1.79E-09	3.77E-08	3.66
ADAMTSL5	1332	9.50E-14	4.04E-12	3.67
SPDYE16	325	3.24E-08	5.36E-07	3.71
CPTP	3361	1.36E-17	8.86E-16	3.72
PIK3CD-AS1	2626	8.25E-12	2.61E-10	3.73
SLC25A22	2577	3.68E-15	1.86E-13	3.73
CYP4F12	211	7.50E-03	2.79E-02	3.74
RAB43	2109	4.96E-08	7.98E-07	3.76
PFN1P2	1468	7.69E-17	4.75E-15	3.76
CACFD1	519	2.67E-07	3.72E-06	3.79
SLC4A2	2804	9.08E-15	4.44E-13	3.80
BOLA2-SMG1P6	4731	6.62E-19	4.81E-17	3.80

WDR86	700	1.64E-08	2.91E-07	3.80
OCLN	1093	5.82E-08	9.23E-07	3.81
RUNX1-IT1	4862	4.22E-16	2.36E-14	3.83
MIRLET7BHG	435	1.98E-05	1.74E-04	3.85
PHC1	12949	1.11E-12	4.00E-11	3.85
SPDYE7P	42	1.11E-02	3.85E-02	3.86
TMEM132E	109	9.99E-04	5.17E-03	3.89
FUT7	2800	1.26E-10	3.32E-09	3.91
CTU1	1035	1.28E-15	6.87E-14	3.92
TSC22D4	6274	1.07E-21	9.42E-20	3.94
PHLDA3	139	6.66E-03	2.53E-02	3.95
CASTOR2	1626	1.05E-21	9.33E-20	3.97
LOC102724238	354	2.63E-03	1.17E-02	3.97
SH3BP5L	1212	3.10E-16	1.77E-14	3.98
DMWD	2944	4.21E-11	1.21E-09	3.99
JMJD1C-AS1	206	2.68E-05	2.27E-04	4.04
SRGAP2-AS1	164	1.78E-03	8.45E-03	4.06
ID3	1866	3.23E-12	1.09E-10	4.07
MRC2	584	1.75E-06	2.02E-05	4.08
LRRC37A2	7408	1.03E-17	6.76E-16	4.09
TMEM102	2021	7.76E-14	3.36E-12	4.09
HMGN2	59303	1.69E-53	7.60E-51	4.11
TP53I13	7157	1.75E-28	2.48E-26	4.11
LOC440300	223	5.93E-05	4.53E-04	4.15
THEM6	4079	1.24E-19	9.59E-18	4.18
FGF17	61	8.93E-03	3.22E-02	4.20
JAG2	121	5.61E-03	2.20E-02	4.21
BRAT1	11858	6.04E-37	1.36E-34	4.24
LINC01138	3882	1.04E-36	2.30E-34	4.24
LOC284454	538	8.67E-07	1.07E-05	4.24
EGLN2	13405	2.79E-26	3.39E-24	4.26
LOC100133331	2190	2.15E-16	1.27E-14	4.26
PKD1P1	5903	1.62E-13	6.64E-12	4.27
SMA4	2549	1.10E-14	5.34E-13	4.28
TMEM250	5470	3.20E-23	3.22E-21	4.28
VWA7	431	2.17E-06	2.44E-05	4.33
LOC100132249	1374	1.25E-08	2.29E-07	4.37
BAIAP3	1128	8.94E-07	1.11E-05	4.43
CD70	272	1.30E-05	1.20E-04	4.43
ANKRD18A	3968	5.42E-10	1.26E-08	4.43
LOC108783654	886	9.28E-08	1.42E-06	4.44
PARP10	25943	8.60E-23	8.45E-21	4.48
FOXJ1	84	4.98E-03	1.99E-02	4.57
FERMT2	131	1.46E-02	4.79E-02	4.58
GCSH	659	1.27E-15	6.84E-14	4.60
LOC102725126	1712	1.32E-18	9.34E-17	4.61

RPS3A	133112	8.69E-47	2.76E-44	4.64
CDC26	8233	1.77E-50	6.57E-48	4.71
LOC440461	344	8.42E-07	1.05E-05	4.72
NBPF15	14230	5.62E-42	1.56E-39	4.73
LOC101926935	413	1.87E-05	1.66E-04	4.74
EFNA3	79	2.71E-03	1.20E-02	4.77
ANKRD20A4-ANKRD20A20P	183	1.25E-02	4.24E-02	4.84
LARGE2	892	4.29E-12	1.41E-10	4.87
CEMP1	2686	1.04E-23	1.08E-21	4.90
LOC110384692	35	1.15E-02	3.96E-02	4.91
TMSB4X	1107041	1.16E-55	5.80E-53	4.92
TEN1-CDK3	494	2.53E-05	2.16E-04	4.93
NPIPB3	6933	4.37E-16	2.44E-14	4.96
EEF1A1	446671	9.16E-89	1.78E-85	4.96
RASA4	5670	1.32E-48	4.45E-46	4.99
H3F3A	60885	2.05E-39	5.20E-37	5.00
FAM201A	1197	5.45E-13	2.07E-11	5.07
RTN4RL1	144	1.34E-02	4.47E-02	5.07
RNF217	248	6.64E-03	2.52E-02	5.09
EGR1	1611	9.20E-08	1.41E-06	5.11
FGF7P3	1076	2.40E-11	7.18E-10	5.13
SMG1P1	24476	1.73E-14	8.08E-13	5.14
ANKRD20A4	200	3.33E-03	1.43E-02	5.14
ESM1	147	8.42E-03	3.07E-02	5.14
BORCS8-MEF2B	543	1.25E-04	8.65E-04	5.19
RPL7	105319	6.09E-55	2.88E-52	5.28
GJD3	97	5.96E-04	3.31E-03	5.32
SLC29A4	56	9.41E-03	3.36E-02	5.33
LINC02256	2020	3.12E-36	6.50E-34	5.34
HIST1H4J	746	1.09E-11	3.37E-10	5.34
LINC00574	98	4.36E-03	1.78E-02	5.44
TUBA3FP	171	1.38E-03	6.81E-03	5.53
IL9R	376	7.54E-07	9.46E-06	5.66
FAM41C	210	3.78E-10	9.11E-09	5.69
NHLRC4	372	2.54E-06	2.81E-05	5.73
CCR10	1763	8.15E-17	5.02E-15	5.74
SLC13A3	86	8.99E-03	3.24E-02	5.78
LINGO3	3618	1.30E-25	1.53E-23	5.78
CCDC92B	79	1.25E-02	4.24E-02	5.85
ABHD17A	11753	4.84E-53	2.11E-50	6.04
RIMBP3	282	4.60E-04	2.66E-03	6.21
LINC01362	83	3.14E-04	1.92E-03	6.41
KRT17P2	536	1.05E-05	9.94E-05	6.48
ADGRB2	245	3.92E-05	3.15E-04	6.62
SMA5	1317	4.32E-31	6.93E-29	6.73

IKBKG	4300	7.65E-77	7.43E-74	6.92
TBC1D3C	54	2.12E-04	1.37E-03	7.01
MALAT1	9092279	7.91E-36	1.61E-33	7.11
UBE2S	2012	9.53E-42	2.60E-39	7.19
CTAGE6	289	5.31E-05	4.12E-04	7.29
LINC02495	91	1.08E-02	3.75E-02	7.34
TBC1D3D	141	1.44E-08	2.59E-07	7.57
TBC1D3I	141	1.44E-08	2.59E-07	7.57
TBC1D3K	141	1.44E-08	2.59E-07	7.57
TBC1D3L	102	7.30E-07	9.18E-06	7.62
SPATA2L	836	4.67E-17	2.97E-15	7.82
TMEM183B	2532	1.83E-04	1.21E-03	7.89
TBC1D3E	147	5.70E-09	1.11E-07	7.92
H3F3AP4	10116	1.50E-34	2.65E-32	8.44
LOC100132062	235	2.89E-11	8.54E-10	8.65
LOC100132287	235	2.89E-11	8.54E-10	8.65
LOC150051	41	5.61E-03	2.20E-02	9.18
GUSBP3	1967	5.47E-56	2.81E-53	9.19
RPL21	74102	4.65E-68	3.70E-65	9.33
NBL1	570	8.44E-18	5.59E-16	9.57
NUDT4P2	2278	6.83E-25	7.86E-23	9.91
CISH	23703	4.43E-35	8.07E-33	10.83
LOC102724580	40	1.27E-04	8.75E-04	10.98
CTAGE8	64	7.11E-03	2.67E-02	11.11
LOC100996740	4800	6.84E-117	2.00E-113	11.37
LOC101927827	127	5.63E-07	7.29E-06	11.42
LOC107984035	908	1.28E-29	1.94E-27	12.99
PPIAL4A	1333	3.39E-41	9.12E-39	13.30
TMEM52	38	1.00E-02	3.53E-02	13.33
RPS26P11	6685	2.19E-44	6.62E-42	14.84
LINC02470	80	5.64E-03	2.21E-02	15.25
TRPC6	30	7.63E-03	2.83E-02	16.40
LOC100134317	30	7.05E-03	2.65E-02	16.62
LOC101928605	169	7.80E-08	1.21E-06	17.88
PAK6	33	1.49E-02	4.86E-02	18.12
RPL41	121666	3.39E-115	8.48E-112	19.16
LINC01189	38	1.53E-04	1.03E-03	20.92
LOC100505915	748	4.41E-38	1.06E-35	21.92
POTEE	3978	4.75E-66	3.46E-63	23.94
**FABP5**	**769**	**9.89E-31**	**1.57E-28**	**25.61**
**LOC441081**	**3000**	**1.59E-80**	**2.14E-77**	**27.37**
**FGF7P6**	**1067**	**1.34E-37**	**3.05E-35**	**47.40**
**PVRIG2P**	**927**	**3.11E-14**	**1.39E-12**	**58.68**
**RPS26**	**21855**	**1.16E-241**	**1.02E-237**	**115.65**
**RPL21P28**	**41177**	**1.03E-106**	**2.25E-103**	**310.84**
**SPDYE8P**	**742**	**1.51E-35**	**2.81E-33**	**407.09**

**LINC02067**	**3907**	**6.90E-58**	**4.03E-55**	**571.09**
**UPK3BL1**	**7050**	**1.34E-53**	**6.16E-51**	**1441.98**
**RPL13AP5**	**15920**	**1.17E-83**	**1.87E-80**	**7165.35**
