# Supplementary material for: Dysregulated lipid metabolism networks modulate T-cell function in people with relapsing-remitting multiple sclerosis
Source: Clin Exp Immunol. 2024 Apr 16;217(2):204–18. doi: 10.1093/cei/uxae032 (PMC11239565; doi:10.1093/cei/uxae032)
Supplement: uxae032_suppl_Supplementary_Data_S2 [file uxae032_suppl_supplementary_data_s2.pdf]

RefSeq	Total counts	P-value (MS-	FDR step up (RRMS vs. HEALTHY)	Fold change (RRMS vs. HEALTHY)
Gene Symbol	Total Counts	p-value	FDR	Fold change
TIMM23	8709.73	1.82E-04	1.21E-03	-1.50
SEC24B	14670.11	8.29E-04	4.41E-03	-1.50
TMEM147	11311.44	4.22E-04	2.47E-03	-1.50
ZBTB14	7035.63	6.79E-06	6.77E-05	-1.50
SMG1	24639.98	3.29E-04	2.00E-03	-1.50
CHD1L	7186.65	5.87E-04	3.27E-03	-1.50
LINC00426	2384.18	6.15E-03	2.37E-02	-1.50
SUCLG2	18169.54	1.18E-06	1.41E-05	-1.50
MRPL49	8359.41	2.65E-05	2.24E-04	-1.50
DIEXF	4689.08	4.24E-03	1.74E-02	-1.50
TTC32	8280.28	6.29E-04	3.48E-03	-1.50
FRG1HP	8396.55	1.23E-03	6.14E-03	-1.50
KANSL2	13976.33	1.25E-04	8.64E-04	-1.50
LNPEP	16801.50	4.04E-03	1.68E-02	-1.50
TRIM5	3039.88	9.21E-04	4.83E-03	-1.50
UTP4	9297.56	3.59E-07	4.86E-06	-1.50
TGOLN2	46884.31	2.18E-05	1.90E-04	-1.50
TRMT5	3456.21	2.41E-03	1.09E-02	-1.50
CYLD	96385.76	2.48E-06	2.75E-05	-1.50
SUMO3	13525.11	2.19E-07	3.09E-06	-1.50
CDC42SE2	162987.96	1.01E-03	5.20E-03	-1.50
DUSP12	9816.69	6.91E-04	3.78E-03	-1.50
GABPB1-IT1	17205.85	3.97E-04	2.34E-03	-1.51
MTCH2	7534.08	1.20E-05	1.12E-04	-1.51
WASL	3616.64	2.13E-03	9.84E-03	-1.51
CD44	133298.64	3.37E-06	3.61E-05	-1.51
TMEM199	6690.09	7.37E-05	5.48E-04	-1.51
ATXN1	5647.64	9.49E-04	4.96E-03	-1.51
NUDT13	767.80	4.62E-03	1.87E-02	-1.51
VSIG1	5152.21	3.99E-05	3.19E-04	-1.51
HEATR3	2789.41	9.06E-03	3.26E-02	-1.51
EIF1B	19167.78	4.57E-04	2.65E-03	-1.51
CTNNBL1	12492.38	5.07E-07	6.63E-06	-1.51
PTPRA	16130.76	3.35E-05	2.74E-04	-1.51
PRKD3	12106.95	2.24E-04	1.44E-03	-1.51
PAPD5	9108.82	1.17E-06	1.40E-05	-1.51
HSDL2	4820.63	3.46E-05	2.82E-04	-1.51
WASHC5	8890.80	3.48E-03	1.48E-02	-1.51
ATMIN	13174.92	8.03E-04	4.30E-03	-1.51
SECISBP2	20708.33	8.41E-05	6.13E-04	-1.51
NT5DC1	11260.69	2.93E-04	1.81E-03	-1.51
PHF10	6288.26	7.71E-05	5.69E-04	-1.51
RARRES3	24599.85	1.03E-03	5.30E-03	-1.51
TPK1	4200.89	1.19E-03	5.98E-03	-1.51
SRP72	34189.06	1.12E-07	1.68E-06	-1.51
EXOC6	5130.66	4.32E-03	1.77E-02	-1.51
EXOSC7	6124.76	1.47E-05	1.33E-04	-1.51
LANCL1	10511.01	6.06E-05	4.63E-04	-1.51
OSBPL9	12080.95	4.29E-06	4.49E-05	-1.51
URB1	2559.78	7.57E-03	2.81E-02	-1.51
WARS	12392.00	2.27E-03	1.04E-02	-1.51
DHRS4	3167.71	3.36E-04	2.03E-03	-1.51

**CAPN3**	1394.01	2.28E-03	1.04E-02	-1.51
**ZNF622**	9966.70	1.82E-05	1.62E-04	-1.51
**RAP1GDS1**	9280.31	6.35E-11	1.77E-09	-1.51
**ATP6V1E2**	1554.74	1.18E-03	5.94E-03	-1.51
**EDEM2**	5326.40	3.93E-05	3.16E-04	-1.51
**ADNP**	9715.06	4.18E-07	5.60E-06	-1.51
**VCL**	12815.44	1.27E-02	4.28E-02	-1.51
**C12orf10**	2917.21	1.47E-03	7.17E-03	-1.51
**PIAS1**	47416.65	6.11E-11	1.71E-09	-1.52
**RICTOR**	29789.85	3.02E-07	4.16E-06	-1.52
**G6PD**	991.64	7.82E-03	2.88E-02	-1.52
**CNOT1**	20932.53	3.80E-03	1.60E-02	-1.52
**NOP14**	7612.79	2.53E-06	2.80E-05	-1.52
**RPL37A**	96421.39	3.09E-03	1.35E-02	-1.52
**THG1L**	1771.29	4.75E-04	2.72E-03	-1.52
**RBMS2**	853.54	1.32E-02	4.43E-02	-1.52
**FBXL5**	30011.66	2.06E-04	1.34E-03	-1.52
**SATB1-AS1**	1339.24	9.52E-03	3.39E-02	-1.52
**SMARCA2**	24134.17	6.06E-06	6.12E-05	-1.52
**TSPAN14**	41011.57	2.83E-06	3.08E-05	-1.52
**EIF2D**	7356.57	2.01E-04	1.31E-03	-1.52
**EIF6**	13166.94	6.41E-04	3.53E-03	-1.52
**MDM4**	33714.84	3.23E-07	4.41E-06	-1.52
**KTI12**	5841.19	9.81E-05	7.01E-04	-1.52
**ZNF639**	13896.19	1.39E-04	9.48E-04	-1.52
**FAM213A**	3013.15	3.72E-03	1.57E-02	-1.52
**SETD1B**	7432.60	1.85E-04	1.22E-03	-1.52
**ZNF169**	1563.60	1.10E-03	5.60E-03	-1.52
**SPIN1**	6846.87	7.83E-04	4.21E-03	-1.52
**TMPO**	19772.08	2.07E-07	2.94E-06	-1.52
**ARG2**	929.40	6.08E-03	2.35E-02	-1.52
**WDR11**	7190.97	6.37E-04	3.52E-03	-1.52
**PDZD8**	3172.94	4.88E-03	1.95E-02	-1.52
**OLA1**	8004.55	7.66E-05	5.66E-04	-1.52
**NR2C2**	9025.98	9.55E-05	6.85E-04	-1.53
**SHMT2**	4014.68	2.10E-03	9.71E-03	-1.53
**SDHAF4**	1136.89	1.07E-02	3.74E-02	-1.53
**POLR2C**	7062.08	6.97E-04	3.81E-03	-1.53
**SNHG17**	5750.09	5.72E-03	2.23E-02	-1.53
**IGBP1**	12694.53	9.57E-04	4.99E-03	-1.53
**FRG1BP**	8944.11	5.88E-03	2.28E-02	-1.53
**EIF2AK3**	4614.31	2.22E-03	1.01E-02	-1.53
**GTF2B**	12090.13	1.31E-03	6.48E-03	-1.53
**PBX3**	6116.55	6.29E-04	3.47E-03	-1.53
**CYP2R1**	2309.74	1.06E-03	5.45E-03	-1.53
**SMCR8**	3062.57	6.38E-04	3.52E-03	-1.53
**FCHSD2**	11662.67	2.28E-04	1.46E-03	-1.53
**REV1**	15268.66	1.01E-05	9.60E-05	-1.53
**ANKRD13A**	19460.95	1.30E-05	1.20E-04	-1.53
**HECA**	20860.84	1.55E-07	2.26E-06	-1.53
**RBL2**	100564.47	2.96E-03	1.30E-02	-1.53
**AUH**	2447.29	9.57E-04	4.99E-03	-1.53
**IPCEF1**	24471.43	2.94E-05	2.46E-04	-1.53
**SLC25A3**	34602.60	9.70E-08	1.47E-06	-1.53
**PVT1**	6463.11	2.11E-05	1.85E-04	-1.53
**SBDS**	33928.94	4.62E-04	2.66E-03	-1.53
**FLJ37453**	5674.34	4.82E-04	2.75E-03	-1.53

**ATG5**	12421.78	1.15E-07	1.73E-06	-1.53
**NEK3**	1666.96	6.88E-03	2.60E-02	-1.53
**BACH1**	7962.22	1.39E-05	1.28E-04	-1.53
**CTDSPL2**	14795.45	3.28E-04	1.99E-03	-1.53
**SATB1**	78612.21	7.21E-06	7.14E-05	-1.54
**ATP6V1A**	8002.05	1.60E-03	7.70E-03	-1.54
**STOML2**	9127.13	6.19E-07	7.90E-06	-1.54
**POLR1B**	5676.49	1.57E-03	7.59E-03	-1.54
**IKZF3**	26407.54	9.45E-06	9.06E-05	-1.54
**MAP3K7**	12042.25	3.25E-05	2.67E-04	-1.54
**ATIC**	14349.46	4.08E-08	6.65E-07	-1.54
**ADSL**	19583.96	2.35E-08	4.01E-07	-1.54
**DENND5A**	15958.97	6.90E-04	3.77E-03	-1.54
**PDSS1**	1247.72	3.99E-05	3.19E-04	-1.54
**PABPC4**	6752.62	8.01E-05	5.87E-04	-1.54
**CD3G**	33778.70	1.51E-03	7.37E-03	-1.54
**IFIT5**	8163.59	6.80E-04	3.73E-03	-1.54
**C12orf42**	1446.81	8.56E-03	3.11E-02	-1.54
**PINK1-AS**	2304.56	5.42E-04	3.05E-03	-1.54
**TMEM256**	2814.25	7.04E-03	2.64E-02	-1.54
**MRPL44**	6458.13	4.46E-04	2.59E-03	-1.54
**COX18**	3068.70	1.22E-04	8.51E-04	-1.54
**CEBPZOS**	10767.14	1.43E-04	9.69E-04	-1.54
**C16orf87**	6936.24	1.33E-03	6.57E-03	-1.54
**ITGA6**	41743.23	6.93E-05	5.21E-04	-1.54
**YY1**	12325.93	5.21E-05	4.06E-04	-1.54
**TMED10**	56900.80	3.03E-05	2.52E-04	-1.54
**RPL23**	63063.83	5.84E-04	3.26E-03	-1.54
**CDS2**	12142.10	8.42E-10	1.88E-08	-1.54
**PCF11**	20046.18	1.80E-06	2.07E-05	-1.55
**MIER3**	3663.83	1.40E-03	6.89E-03	-1.55
**ELF1**	62792.16	4.03E-06	4.25E-05	-1.55
**RNASEH1**	12229.32	4.56E-07	6.03E-06	-1.55
**KPNA2**	3996.57	1.10E-02	3.81E-02	-1.55
**ZBTB1**	22328.94	7.13E-06	7.07E-05	-1.55
**FIP1L1**	11041.88	1.44E-03	7.03E-03	-1.55
**NDUFS3**	7948.69	3.77E-04	2.24E-03	-1.55
**SLC5A3**	8326.04	7.11E-04	3.88E-03	-1.55
**TNRC6B**	51031.14	2.18E-08	3.78E-07	-1.55
**ZNF213-AS1**	1480.95	1.67E-05	1.50E-04	-1.55
**ADD3**	140829.32	2.14E-06	2.41E-05	-1.55
**PITRM1**	10453.18	1.25E-04	8.68E-04	-1.55
**ATF6**	16329.78	6.73E-07	8.52E-06	-1.55
**PLEKHA1**	22462.38	7.51E-06	7.39E-05	-1.55
**AFG3L2**	8253.27	4.78E-07	6.28E-06	-1.55
**FOXO3B**	4033.44	1.56E-03	7.53E-03	-1.55
**MRPS23**	6678.47	3.00E-03	1.31E-02	-1.55
**UBXN1**	15540.32	6.39E-05	4.85E-04	-1.55
**DMXL1**	12167.87	8.10E-07	1.01E-05	-1.55
**MFN1**	3901.51	1.51E-03	7.36E-03	-1.55
**TADA2B**	4807.43	2.97E-03	1.30E-02	-1.55
**GPD2**	2429.92	1.99E-04	1.30E-03	-1.55
**SMAGP**	1772.73	4.39E-03	1.79E-02	-1.55
**CD99P1**	1936.02	3.75E-05	3.03E-04	-1.55
**PPM1A**	7241.01	1.13E-07	1.70E-06	-1.55
**DDX1**	13512.68	6.25E-10	1.43E-08	-1.55
**H2AFY**	22519.24	9.93E-05	7.08E-04	-1.55

**ZBTB18**	3133.67	8.94E-03	3.22E-02	-1.55
**TEX10**	5349.41	7.36E-06	7.25E-05	-1.55
**RWDD2A**	2264.39	1.32E-03	6.54E-03	-1.56
**CBLB**	15003.03	1.42E-05	1.29E-04	-1.56
**TSR2**	12778.86	1.76E-07	2.53E-06	-1.56
**ITGA4**	80896.98	4.79E-05	3.76E-04	-1.56
**GLA**	1540.33	2.99E-03	1.31E-02	-1.56
**ZDHHC3**	6845.87	1.84E-06	2.11E-05	-1.56
**SLC16A7**	11079.73	1.13E-03	5.75E-03	-1.56
**ADPRM**	18670.01	8.66E-04	4.58E-03	-1.56
**ANKIB1**	6442.44	3.16E-08	5.25E-07	-1.56
**ERBIN**	33797.01	3.62E-05	2.93E-04	-1.56
**LYSMD4**	2938.71	1.27E-02	4.29E-02	-1.56
**CXorf65**	2550.48	8.18E-05	5.98E-04	-1.56
**TMEM135**	3961.56	8.44E-04	4.48E-03	-1.56
**RPL12**	48521.58	5.24E-04	2.97E-03	-1.56
**L3MBTL3**	5547.18	2.92E-03	1.28E-02	-1.56
**DYNLT1**	17481.98	8.41E-04	4.47E-03	-1.56
**RTCB**	11579.09	2.83E-05	2.38E-04	-1.56
**SLC6A6**	3096.67	8.95E-04	4.72E-03	-1.56
**VOPP1**	10668.17	2.00E-05	1.76E-04	-1.56
**TBC1D5**	11170.15	6.08E-06	6.13E-05	-1.56
**ARHGAP19**	3056.46	4.12E-03	1.70E-02	-1.56
**MATR3**	99981.64	9.94E-08	1.51E-06	-1.56
**RPS3**	179125.39	2.79E-05	2.35E-04	-1.56
**SLC30A9**	13707.12	1.35E-06	1.60E-05	-1.56
**SHISAL2A**	1294.28	3.86E-03	1.62E-02	-1.56
**SLC9A7**	2146.84	2.20E-03	1.01E-02	-1.56
**SOD2**	24903.96	4.13E-05	3.30E-04	-1.56
**SLC25A26**	6509.65	2.79E-06	3.05E-05	-1.56
**OSBPL8**	39442.95	7.19E-08	1.13E-06	-1.56
**PEPD**	10767.22	2.42E-05	2.08E-04	-1.56
**LINC01089**	6898.88	7.54E-04	4.08E-03	-1.57
**CD47**	78992.19	2.49E-11	7.41E-10	-1.57
**GCFC2**	7842.12	3.80E-04	2.26E-03	-1.57
**CD55**	28899.17	9.72E-06	9.28E-05	-1.57
**STX3**	1829.00	4.76E-03	1.92E-02	-1.57
**GMPR2**	12025.73	1.36E-09	2.92E-08	-1.57
**AHCTF1P1**	6471.22	5.14E-03	2.04E-02	-1.57
**DPCD**	387.26	1.42E-02	4.68E-02	-1.57
**NUDT21**	13939.47	1.48E-06	1.74E-05	-1.57
**MAML2**	13643.16	4.35E-06	4.54E-05	-1.57
**CTAGE1**	669.20	2.76E-03	1.22E-02	-1.57
**NCL**	95913.35	2.92E-07	4.03E-06	-1.57
**TRIM52-AS1**	7131.31	7.66E-05	5.66E-04	-1.57
**TWISTNB**	9574.27	1.33E-04	9.14E-04	-1.57
**PSMC2**	19709.91	1.49E-09	3.18E-08	-1.57
**ZMAT2**	11103.52	1.66E-07	2.39E-06	-1.57
**FBXO25**	10030.33	7.25E-05	5.40E-04	-1.57
**C1orf43**	16190.81	5.68E-05	4.37E-04	-1.57
**ACSM3**	946.94	9.87E-03	3.50E-02	-1.57
**MSTO2P**	4301.66	1.73E-03	8.25E-03	-1.57
**TRAF3IP2**	2315.97	1.05E-03	5.42E-03	-1.57
**FUCA2**	3287.85	2.37E-04	1.51E-03	-1.57
**LRRC37A**	3043.12	1.37E-02	4.55E-02	-1.57
**SOCS4**	3510.85	2.53E-03	1.13E-02	-1.57
**ARL2BP**	33881.73	1.17E-08	2.16E-07	-1.57

**COPRS**	1325.43	1.59E-03	7.65E-03	-1.58
**NCOA3**	19841.82	4.60E-04	2.66E-03	-1.58
**MCM6**	5271.73	1.39E-04	9.49E-04	-1.58
**BRF2**	2058.55	7.31E-03	2.73E-02	-1.58
**RBM3**	48310.13	2.57E-05	2.19E-04	-1.58
**CSNK1G3**	8365.76	1.04E-04	7.37E-04	-1.58
**ALOX12-AS1**	586.19	1.07E-02	3.74E-02	-1.58
**WDR48**	16763.29	2.37E-06	2.64E-05	-1.58
**DDX47**	15029.10	1.47E-06	1.74E-05	-1.58
**MTURN**	2128.70	1.46E-04	9.94E-04	-1.58
**WHAMMP1**	5891.08	1.09E-02	3.78E-02	-1.58
**CXorf56**	3511.49	1.77E-04	1.17E-03	-1.58
**FGD5-AS1**	20175.41	3.37E-08	5.57E-07	-1.58
**FAM160B1**	10368.29	3.49E-07	4.74E-06	-1.58
**PGM1**	4713.80	8.98E-05	6.50E-04	-1.58
**SHMT1**	2752.55	6.87E-04	3.76E-03	-1.58
**MAN2A1**	16475.76	2.34E-05	2.03E-04	-1.58
**ZNF738**	3638.65	2.03E-04	1.32E-03	-1.58
**LRMP**	13554.94	1.61E-04	1.08E-03	-1.58
**PPT1**	48709.49	3.68E-05	2.98E-04	-1.58
**MTPN**	12860.73	4.59E-04	2.66E-03	-1.58
**LUZP6**	12860.73	4.59E-04	2.66E-03	-1.58
**SENP2**	4612.15	4.61E-04	2.66E-03	-1.59
**POLR2B**	25148.21	1.68E-07	2.42E-06	-1.59
**VWA8**	4304.88	9.90E-05	7.07E-04	-1.59
**SLC35F6**	2571.43	1.50E-06	1.77E-05	-1.59
**TTPAL**	4416.71	2.11E-04	1.36E-03	-1.59
**GARS**	12011.94	8.76E-11	2.36E-09	-1.59
**MT1X**	4829.02	5.13E-03	2.04E-02	-1.59
**FAM107B**	91243.04	2.20E-09	4.58E-08	-1.59
**SP1**	5739.30	7.50E-05	5.57E-04	-1.59
**BRPF3**	3053.02	1.36E-02	4.52E-02	-1.59
**UBR4**	17011.40	8.71E-08	1.34E-06	-1.59
**TALDO1**	16712.23	3.75E-06	3.98E-05	-1.59
**MSANTD3**	1962.96	3.00E-03	1.31E-02	-1.59
**UBE2J1**	20446.99	6.87E-06	6.83E-05	-1.59
**KBTBD4**	9485.24	4.76E-05	3.74E-04	-1.59
**MBNL3**	6778.90	7.18E-03	2.69E-02	-1.59
**PIGC**	14988.78	4.25E-06	4.45E-05	-1.59
**AK5**	11891.33	3.01E-03	1.31E-02	-1.59
**HIPK1**	14572.50	1.31E-10	3.46E-09	-1.60
**OXCT1**	10988.74	5.77E-09	1.12E-07	-1.60
**GRK5**	3309.28	3.55E-04	2.13E-03	-1.60
**CDK2AP1**	2409.67	1.27E-02	4.30E-02	-1.60
**XYLT1**	2770.59	3.42E-04	2.06E-03	-1.60
**STX17**	16949.28	1.92E-06	2.19E-05	-1.60
**TMEM2**	13583.18	6.58E-03	2.50E-02	-1.60
**SMURF2**	4282.44	2.69E-04	1.68E-03	-1.60
**ZNF836**	3986.89	1.78E-04	1.18E-03	-1.60
**NDUFB9**	31929.90	1.43E-04	9.73E-04	-1.60
**GLO1**	21709.60	2.27E-08	3.90E-07	-1.60
**GTF3A**	69039.50	1.64E-05	1.47E-04	-1.60
**ABCB7**	4403.13	2.01E-06	2.29E-05	-1.60
**IMPACT**	6531.78	9.19E-03	3.30E-02	-1.60
**ATP8A1**	9738.70	6.52E-05	4.94E-04	-1.60
**PTPRM**	3127.52	3.13E-03	1.36E-02	-1.60
**SWAP70**	1999.29	1.91E-03	8.98E-03	-1.60

**FAU**	44174.31	3.85E-04	2.28E-03	-1.60
**BAG1**	8599.72	3.67E-09	7.42E-08	-1.60
**ZKSCAN8**	3273.51	1.41E-02	4.66E-02	-1.60
**RABL2A**	3669.52	5.22E-04	2.96E-03	-1.60
**GHITM**	55410.91	1.27E-06	1.52E-05	-1.60
**SNRPF**	12374.87	2.20E-03	1.01E-02	-1.60
**PITHD1**	10435.08	1.54E-07	2.25E-06	-1.60
**AKAP2**	6049.21	1.49E-03	7.29E-03	-1.60
**CNOT2**	29889.49	7.21E-11	1.97E-09	-1.60
**G3BP2**	38498.97	1.59E-06	1.85E-05	-1.61
**DDB1**	15080.70	7.04E-04	3.84E-03	-1.61
**LOC104968399**	2273.95	8.18E-05	5.98E-04	-1.61
**NME4**	4550.17	2.30E-03	1.04E-02	-1.61
**APMAP**	18465.53	6.77E-05	5.10E-04	-1.61
**ACSL6**	4695.33	1.07E-03	5.49E-03	-1.61
**PTPN9**	5349.96	2.27E-04	1.46E-03	-1.61
**TFAM**	12698.98	5.30E-06	5.41E-05	-1.61
**HDAC1**	28048.19	7.20E-08	1.13E-06	-1.61
**TMEM159**	1760.70	3.73E-03	1.57E-02	-1.61
**PLCL2**	28246.67	1.86E-04	1.23E-03	-1.61
**LRPPRC**	15237.25	4.66E-05	3.67E-04	-1.61
**SCML1**	4737.13	7.86E-04	4.22E-03	-1.61
**CCNG2**	3894.85	2.01E-03	9.35E-03	-1.61
**CLINT1**	23504.31	5.05E-12	1.64E-10	-1.61
**UBA52**	63799.42	4.74E-04	2.72E-03	-1.61
**GTDC1**	3421.35	4.77E-04	2.73E-03	-1.61
**DNAJB11**	6904.91	1.24E-06	1.48E-05	-1.61
**QARS**	14706.41	6.38E-06	6.39E-05	-1.61
**STMN1**	22597.77	8.58E-04	4.55E-03	-1.61
**FDPS**	7746.23	6.37E-08	1.00E-06	-1.61
**ARHGAP27P1-BPTFP1-KPNA2P3**	3443.78	2.52E-05	2.14E-04	-1.61
**LOC100133315**	786.15	4.27E-03	1.75E-02	-1.61
**NCOA4**	31967.15	2.10E-04	1.36E-03	-1.61
**PDHX**	5779.12	3.09E-06	3.33E-05	-1.61
**LSM14B**	846.99	5.22E-03	2.07E-02	-1.61
**EPHA4**	8488.68	2.86E-04	1.77E-03	-1.62
**TBC1D15**	15678.28	1.04E-05	9.91E-05	-1.62
**PPIF**	3595.11	4.63E-04	2.67E-03	-1.62
**ARPC2**	106854.92	5.57E-09	1.09E-07	-1.62
**MAPKAPK2**	2167.39	1.45E-02	4.77E-02	-1.62
**FEN1**	2404.10	2.54E-03	1.14E-02	-1.62
**GABARAPL1**	3159.58	8.39E-04	4.46E-03	-1.62
**BOLA2**	6514.06	8.45E-03	3.07E-02	-1.62
**HS2ST1**	3534.06	1.05E-04	7.42E-04	-1.62
**HNRNPH2**	6644.58	3.14E-05	2.59E-04	-1.62
**BCL10**	6239.72	6.83E-06	6.79E-05	-1.62
**LST1**	3418.85	1.25E-02	4.24E-02	-1.62
**NAP1L5**	2074.68	4.24E-03	1.74E-02	-1.62
**GGNBP2**	44375.81	5.91E-10	1.36E-08	-1.62
**CA11**	989.96	7.23E-03	2.71E-02	-1.62
**UHRF2**	12258.51	5.28E-15	2.65E-13	-1.62
**NME1**	6939.09	3.59E-04	2.15E-03	-1.62
**PTAR1**	8982.28	1.11E-07	1.68E-06	-1.62
**CPNE3**	21226.87	7.19E-07	9.06E-06	-1.62
**SEL1L3**	14750.49	6.38E-07	8.12E-06	-1.62
**BUD23**	13043.61	4.50E-09	8.92E-08	-1.63

**SAMHD1**	76651.99	7.26E-07	9.14E-06	-1.63
**ITGAE**	5780.39	8.45E-05	6.16E-04	-1.63
**ZNF839**	3288.86	2.00E-03	9.29E-03	-1.63
**MAGED1**	7735.46	9.36E-04	4.90E-03	-1.63
**MT2A**	8467.96	6.63E-03	2.52E-02	-1.63
**SPTBN1**	37345.18	1.15E-03	5.83E-03	-1.63
**RPA1**	20185.66	9.87E-07	1.21E-05	-1.63
**KLF11**	3160.04	6.02E-03	2.33E-02	-1.63
**PARK7**	26973.53	3.54E-06	3.78E-05	-1.63
**COTL1**	65465.99	1.03E-07	1.56E-06	-1.63
**TOMM20**	25863.34	5.12E-06	5.25E-05	-1.63
**TSPAN3**	10443.07	3.36E-04	2.03E-03	-1.63
**DCTN4**	5559.40	2.29E-04	1.47E-03	-1.63
**HSPA14**	7192.22	2.65E-12	9.07E-11	-1.63
**TSPYL4**	9697.22	1.11E-05	1.04E-04	-1.63
**SNX9**	12542.69	1.26E-07	1.88E-06	-1.63
**PSMD14**	11049.16	4.15E-06	4.35E-05	-1.63
**ZNF496**	2478.87	3.44E-04	2.07E-03	-1.63
**NKAPD1**	4377.02	1.24E-06	1.48E-05	-1.63
**MXD1**	3022.83	9.04E-07	1.12E-05	-1.63
**EXT2**	6943.46	3.46E-05	2.82E-04	-1.63
**PELI2**	2338.49	3.95E-03	1.64E-02	-1.63
**ZNF664**	3031.44	7.48E-04	4.05E-03	-1.63
**ANXA2**	28185.22	8.05E-04	4.31E-03	-1.63
**DYNC1LI1**	6675.50	4.16E-08	6.76E-07	-1.63
**TIFA**	6839.39	1.54E-03	7.46E-03	-1.63
**SBDSP1**	22231.67	9.80E-07	1.20E-05	-1.63
**JOSD1**	3953.24	9.91E-04	5.13E-03	-1.63
**ATXN7L3B**	26374.27	1.41E-05	1.29E-04	-1.64
**RPL24**	33880.51	4.08E-04	2.40E-03	-1.64
**IGF2R**	12159.20	4.29E-06	4.49E-05	-1.64
**TMX4**	20441.71	9.04E-09	1.69E-07	-1.64
**SMAP2**	71677.98	5.06E-07	6.62E-06	-1.64
**COPS2**	11580.53	8.42E-09	1.59E-07	-1.64
**RGS10**	22104.50	2.72E-04	1.69E-03	-1.64
**JAK1**	105925.25	9.19E-09	1.72E-07	-1.64
**ATP5A1**	64651.49	2.46E-08	4.17E-07	-1.64
**FAM49B**	38111.66	1.29E-07	1.92E-06	-1.64
**INTS6-AS1**	5078.06	5.07E-05	3.96E-04	-1.64
**SLC25A16**	2816.70	7.38E-07	9.27E-06	-1.64
**MSN**	97392.15	3.64E-04	2.18E-03	-1.64
**PDK3**	22478.57	9.67E-09	1.80E-07	-1.64
**ZNF32**	10978.62	3.69E-07	4.98E-06	-1.64
**LRCH1**	3086.28	4.62E-06	4.80E-05	-1.64
**KCTD10**	4906.87	6.87E-04	3.76E-03	-1.64
**PPM1G**	14053.36	9.79E-19	6.99E-17	-1.64
**RPN1**	32579.65	1.01E-07	1.53E-06	-1.64
**PGPEP1**	2770.68	3.59E-04	2.15E-03	-1.64
**RBM14-RBM4**	2015.27	9.14E-03	3.28E-02	-1.64
**ADPGK**	17822.39	2.17E-06	2.44E-05	-1.64
**ASAH1**	14805.10	3.85E-09	7.76E-08	-1.64
**LINC01772**	1040.07	9.15E-04	4.81E-03	-1.64
**DLG3**	1624.82	2.34E-03	1.06E-02	-1.64
**AMD1**	24610.46	3.66E-08	6.02E-07	-1.65
**MLLT11**	2538.87	6.96E-04	3.80E-03	-1.65
**UQCRC2**	20486.51	1.33E-07	1.98E-06	-1.65
**CACNA1C-AS2**	641.88	1.41E-02	4.66E-02	-1.65

**SETD1A**	2222.21	4.62E-04	2.67E-03	-1.65
**UROS**	7424.02	8.05E-08	1.25E-06	-1.65
**PDIA5**	871.65	6.07E-04	3.37E-03	-1.65
**PCCA**	1974.26	2.66E-04	1.66E-03	-1.65
**USP54**	841.95	2.02E-03	9.40E-03	-1.65
**C7orf55-LUC7L2**	6808.83	1.54E-05	1.40E-04	-1.65
**ZC4H2**	2779.76	1.20E-04	8.36E-04	-1.65
**PDCD6IP**	29161.41	1.92E-10	4.90E-09	-1.65
**LANCL2**	2544.63	2.22E-03	1.02E-02	-1.65
**C10orf143**	2154.58	1.90E-04	1.24E-03	-1.65
**TIPARP**	7080.55	1.99E-03	9.29E-03	-1.65
**ELL2**	1588.59	5.41E-03	2.13E-02	-1.65
**USPL1**	12360.86	8.45E-10	1.88E-08	-1.65
**ZNF518B**	6035.19	2.68E-03	1.19E-02	-1.65
**MAP4K4**	25868.77	2.25E-06	2.52E-05	-1.65
**TMEM256-PLSCR3**	10594.62	5.92E-06	5.99E-05	-1.65
**SLC27A5**	926.51	1.08E-02	3.75E-02	-1.65
**SUMO1P3**	8809.00	1.16E-05	1.09E-04	-1.65
**TMEM191C**	565.59	4.30E-03	1.76E-02	-1.65
**ZDHHC20**	9896.96	6.27E-06	6.31E-05	-1.65
**ERICH6-AS1**	491.95	5.31E-03	2.10E-02	-1.65
**CD79A**	724.05	3.43E-03	1.47E-02	-1.66
**ZFP91**	8733.11	1.66E-07	2.39E-06	-1.66
**SERPINB6**	8182.24	1.33E-04	9.16E-04	-1.66
**SNW1**	17922.76	3.65E-09	7.38E-08	-1.66
**SLC39A6**	8721.25	3.19E-04	1.95E-03	-1.66
**LRRC8D**	12799.92	2.51E-04	1.58E-03	-1.66
**USP53**	11247.57	5.08E-04	2.89E-03	-1.66
**NUTM2A-AS1**	1578.09	1.51E-03	7.34E-03	-1.66
**MINCR**	2398.07	2.01E-03	9.37E-03	-1.66
**ETS2**	3276.01	9.37E-04	4.90E-03	-1.66
**PCCB**	3757.75	1.06E-05	9.99E-05	-1.66
**CSNK2A2**	801.57	1.10E-02	3.80E-02	-1.67
**HIVEP2**	18801.11	5.84E-05	4.48E-04	-1.67
**NUCKS1**	15751.25	2.62E-07	3.64E-06	-1.67
**SAP130**	5286.17	8.88E-06	8.57E-05	-1.67
**BECN1**	25642.23	3.74E-11	1.08E-09	-1.67
**FBXO33**	18561.03	1.35E-08	2.45E-07	-1.67
**RFX7**	6452.73	1.18E-04	8.23E-04	-1.67
**ATP11C**	4617.19	2.12E-05	1.86E-04	-1.67
**RPS11**	86247.88	1.10E-04	7.73E-04	-1.67
**TGFBR2**	56814.00	8.98E-08	1.38E-06	-1.67
**TMEM161B-AS1**	1794.19	2.16E-05	1.89E-04	-1.67
**RGP1**	904.58	5.10E-03	2.03E-02	-1.67
**SAT1**	33816.59	8.96E-06	8.63E-05	-1.67
**ZNF581**	3592.57	1.46E-06	1.72E-05	-1.67
**MTA2**	3824.12	6.91E-04	3.78E-03	-1.67
**LOC105369332**	443.58	1.29E-03	6.40E-03	-1.68
**RAB30**	3490.50	3.88E-03	1.62E-02	-1.68
**ITCH**	7857.81	6.87E-07	8.68E-06	-1.68
**YPEL5**	44916.22	3.12E-06	3.35E-05	-1.68
**DNAJC13**	8721.07	8.13E-07	1.02E-05	-1.68
**APLP2**	14766.92	1.80E-08	3.16E-07	-1.68
**LYRM4**	4218.98	5.88E-05	4.50E-04	-1.68
**RPL35A**	87919.68	2.08E-04	1.35E-03	-1.68
**YBX3**	1528.55	5.20E-03	2.06E-02	-1.68
**SFMBT1**	8186.05	7.67E-08	1.20E-06	-1.68

**FH**	5041.95	2.57E-07	3.58E-06	-1.68
**MSH6**	7328.38	5.68E-04	3.18E-03	-1.68
**CCT2**	19269.35	4.08E-12	1.35E-10	-1.68
**BBOF1**	1114.69	7.22E-04	3.93E-03	-1.68
**GLTP**	8002.73	4.52E-05	3.57E-04	-1.68
**SERTAD2**	5722.50	1.53E-04	1.03E-03	-1.68
**WASHC1**	1991.70	5.13E-03	2.04E-02	-1.68
**OSGIN2**	5593.97	5.44E-05	4.21E-04	-1.68
**RPS16**	74329.11	6.63E-05	5.01E-04	-1.69
**SESN1**	22224.13	1.64E-05	1.47E-04	-1.69
**BBX**	36686.34	5.10E-09	1.01E-07	-1.69
**LINC01184**	2150.51	3.17E-04	1.94E-03	-1.69
**NCOA6**	8892.93	1.27E-08	2.32E-07	-1.69
**HSF2**	9584.40	2.39E-05	2.06E-04	-1.69
**RNF114**	21208.61	2.64E-10	6.58E-09	-1.69
**CBLL1**	22794.88	1.74E-09	3.68E-08	-1.69
**MAN1A1**	5569.62	5.98E-05	4.57E-04	-1.69
**TPTE2P5**	615.39	2.08E-03	9.63E-03	-1.69
**BARD1**	2548.04	1.37E-05	1.26E-04	-1.69
**GANAB**	21390.03	1.09E-05	1.02E-04	-1.69
**ACVR1B**	828.28	1.40E-02	4.64E-02	-1.69
**SGPP2**	1061.61	1.15E-02	3.94E-02	-1.69
**AGO3**	6421.14	6.69E-04	3.68E-03	-1.69
**TCEA3**	6732.87	1.84E-05	1.63E-04	-1.69
**DHRS3**	7396.63	1.70E-05	1.52E-04	-1.69
**WDR76**	2130.73	5.72E-05	4.40E-04	-1.69
**DIP2B**	12348.49	9.78E-04	5.08E-03	-1.69
**FBP1**	2249.07	8.44E-03	3.07E-02	-1.70
**TTC5**	6406.58	4.90E-05	3.83E-04	-1.70
**LOC100506548**	3294.02	3.13E-05	2.59E-04	-1.70
**HSPA1L**	1419.45	1.46E-04	9.93E-04	-1.70
**HIST1H2BN**	413.26	1.22E-02	4.17E-02	-1.70
**ERLIN2**	2204.91	4.36E-06	4.55E-05	-1.70
**TLR2**	1135.98	8.95E-03	3.23E-02	-1.70
**CAB39L**	1267.03	7.68E-04	4.15E-03	-1.70
**ZSWIM6**	1741.04	3.97E-03	1.65E-02	-1.70
**LIMK2**	8894.82	7.69E-05	5.68E-04	-1.70
**SNX30**	1817.02	4.69E-03	1.89E-02	-1.70
**TMEM183A**	8774.46	2.91E-05	2.44E-04	-1.70
**FKTN**	1907.66	2.56E-04	1.61E-03	-1.70
**CSNK2A1**	5307.01	2.80E-07	3.89E-06	-1.70
**SFT2D2**	2978.27	3.02E-05	2.51E-04	-1.70
**CREBBP**	16853.43	6.83E-10	1.55E-08	-1.71
**FAM227B**	1149.62	2.98E-03	1.31E-02	-1.71
**FAM228B**	1217.85	2.08E-03	9.62E-03	-1.71
**OXA1L**	15325.37	1.84E-11	5.59E-10	-1.71
**MTHFD1**	4659.53	1.63E-05	1.47E-04	-1.71
**LOC101928111**	724.68	1.05E-02	3.69E-02	-1.71
**MTMR10**	3324.92	8.96E-04	4.72E-03	-1.71
**CAT**	20049.14	1.55E-07	2.26E-06	-1.71
**CEP85L**	23661.03	2.65E-06	2.91E-05	-1.72
**PCYT1A**	3739.19	6.55E-06	6.55E-05	-1.72
**HIF1A**	36460.46	8.55E-07	1.06E-05	-1.72
**VPS8**	9761.47	1.36E-04	9.31E-04	-1.72
**TMEM44-AS1**	586.30	1.44E-02	4.74E-02	-1.72
**IFT52**	4683.59	3.93E-07	5.28E-06	-1.72
**FEM1B**	11873.41	2.93E-07	4.04E-06	-1.72

**IARS2**	18267.14	1.46E-06	1.72E-05	-1.72
**GNS**	10677.29	6.30E-06	6.33E-05	-1.72
**ZBTB2**	6740.76	5.99E-04	3.33E-03	-1.72
**FHL1**	7639.49	8.86E-04	4.68E-03	-1.72
**IRS2**	6659.55	1.92E-04	1.26E-03	-1.72
**RMDN2**	932.08	3.20E-03	1.38E-02	-1.73
**UBE2E2**	2574.85	4.10E-03	1.70E-02	-1.73
**DUSP10**	2542.65	4.31E-05	3.42E-04	-1.73
**SGMS1**	6529.71	1.71E-04	1.14E-03	-1.73
**PIGA**	4041.16	2.39E-04	1.52E-03	-1.73
**ZNF557**	4768.71	6.92E-05	5.20E-04	-1.73
**CNOT6L**	25514.80	1.13E-14	5.43E-13	-1.73
**DTD1**	8489.86	1.40E-07	2.07E-06	-1.73
**DICER1**	18666.33	1.96E-11	5.90E-10	-1.73
**RCAN3AS**	1060.53	1.24E-03	6.18E-03	-1.74
**VWA5A**	2274.22	2.10E-03	9.71E-03	-1.74
**WHAMMP3**	4771.46	3.42E-05	2.79E-04	-1.74
**TBL1X**	6228.82	8.48E-07	1.05E-05	-1.74
**DDI2**	1101.49	1.14E-03	5.81E-03	-1.74
**AIFM1**	4053.37	2.10E-08	3.66E-07	-1.74
**ARHGAP26**	6735.27	4.02E-08	6.54E-07	-1.74
**PDP1**	8882.12	4.91E-04	2.80E-03	-1.74
**CCNB1IP1**	1997.27	2.57E-04	1.61E-03	-1.74
**CENPV**	1900.60	3.30E-05	2.71E-04	-1.74
**TPP2**	32389.13	1.75E-06	2.02E-05	-1.74
**DDX28**	7709.40	6.16E-07	7.90E-06	-1.74
**PIK3IP1**	148320.04	2.08E-06	2.35E-05	-1.74
**TRIM59**	5338.74	3.96E-03	1.65E-02	-1.74
**CDK7**	3322.47	8.40E-06	8.18E-05	-1.74
**KDM3A**	20226.22	5.20E-05	4.05E-04	-1.74
**OSBPL1A**	1165.18	1.35E-02	4.50E-02	-1.74
**SNHG3**	16339.59	2.14E-06	2.40E-05	-1.75
**RAPGEF2**	1883.66	2.78E-03	1.23E-02	-1.75
**IMPA2**	1566.64	3.45E-03	1.47E-02	-1.75
**C12orf57**	15064.09	6.81E-04	3.73E-03	-1.75
**EXOSC9**	9137.08	2.63E-06	2.89E-05	-1.75
**ABHD17B**	4433.93	1.57E-06	1.83E-05	-1.75
**TRIM59-IFT80**	8428.04	2.15E-04	1.38E-03	-1.75
**LBHD1**	1242.37	5.44E-03	2.14E-02	-1.75
**ITPKB-IT1**	356.39	6.09E-03	2.35E-02	-1.75
**STX11**	3465.06	5.04E-03	2.01E-02	-1.76
**YRDC**	6896.89	4.38E-04	2.55E-03	-1.76
**CFL2**	3553.75	1.04E-03	5.34E-03	-1.76
**C6orf229**	268.49	1.44E-02	4.73E-02	-1.76
**ZFAS1**	22046.80	7.95E-06	7.79E-05	-1.76
**ZCCHC2**	3004.68	5.51E-05	4.27E-04	-1.76
**PRKCQ**	27799.89	1.14E-09	2.49E-08	-1.76
**MAPK9**	5936.20	3.64E-05	2.94E-04	-1.76
**SSFA2**	2921.76	4.93E-06	5.09E-05	-1.76
**FIS1**	13153.71	3.11E-05	2.58E-04	-1.76
**XRN2**	32706.46	4.92E-16	2.73E-14	-1.76
**ZNF460**	3703.11	6.46E-04	3.56E-03	-1.76
**SKAP2**	5408.53	3.07E-03	1.34E-02	-1.76
**LOC101929718**	799.68	6.00E-05	4.58E-04	-1.77
**TRIM66**	1650.14	1.97E-03	9.19E-03	-1.77
**BZW2**	10952.18	4.43E-07	5.89E-06	-1.77
**TENM1**	6396.85	8.93E-08	1.37E-06	-1.77

**LOC100507006**	1311.02	4.22E-03	1.74E-02	-1.77
**KATNAL2**	379.97	1.46E-02	4.80E-02	-1.77
**KLHL15**	2730.00	2.55E-03	1.14E-02	-1.77
**SNRK**	31454.19	1.82E-10	4.67E-09	-1.77
**RAB12**	594.40	8.30E-03	3.03E-02	-1.77
**SNORA81**	641.51	1.95E-03	9.13E-03	-1.77
**CTSS**	37663.16	1.11E-06	1.34E-05	-1.77
**ARHGAP32**	2299.15	4.40E-03	1.80E-02	-1.77
**VHL**	12123.85	5.54E-11	1.55E-09	-1.77
**TYW1B**	3276.40	1.20E-02	4.12E-02	-1.77
**MCM4**	3057.56	5.71E-03	2.23E-02	-1.77
**LIPA**	23489.48	1.89E-11	5.73E-10	-1.77
**MED13**	11377.15	6.18E-07	7.90E-06	-1.78
**XPOT**	16039.05	4.57E-06	4.75E-05	-1.78
**SLC35F2**	2111.43	3.31E-03	1.42E-02	-1.78
**SCARNA12**	306.20	4.43E-03	1.81E-02	-1.78
**LOC100291105**	725.25	8.23E-04	4.39E-03	-1.78
**SMAD7**	1012.01	9.40E-03	3.35E-02	-1.78
**ARF1**	64109.71	9.69E-12	3.02E-10	-1.78
**RTFDC1**	17007.16	8.14E-11	2.20E-09	-1.78
**INTS14**	4220.33	1.05E-06	1.27E-05	-1.78
**B4GALT1**	7891.43	3.33E-05	2.73E-04	-1.78
**RPN2**	50027.45	3.81E-17	2.43E-15	-1.78
**PARL**	12813.24	4.23E-07	5.66E-06	-1.79
**IFI6**	9496.18	4.46E-06	4.64E-05	-1.79
**ENPP2**	1132.80	2.68E-03	1.19E-02	-1.79
**SSR2**	31347.74	2.54E-08	4.28E-07	-1.79
**ZNF410**	8209.42	1.73E-06	1.99E-05	-1.79
**IFNGR2**	6384.18	1.09E-09	2.39E-08	-1.79
**CTAGE7P**	466.53	7.29E-03	2.72E-02	-1.79
**HIVEP1**	11144.77	2.86E-05	2.40E-04	-1.79
**DAP3**	16251.00	9.27E-12	2.90E-10	-1.79
**TRIAP1**	8311.56	6.19E-07	7.90E-06	-1.79
**CDKN1B**	12408.52	4.33E-07	5.76E-06	-1.79
**UXT**	18692.21	3.93E-06	4.15E-05	-1.79
**RIC1**	7515.45	8.45E-06	8.22E-05	-1.80
**SNHG19**	487.76	1.14E-02	3.94E-02	-1.80
**RYBP**	4472.16	1.75E-05	1.56E-04	-1.80
**EPB41L2**	1526.79	1.42E-02	4.69E-02	-1.80
**ATP1A1**	15480.25	6.52E-10	1.48E-08	-1.80
**LRRC37A3**	1807.25	5.06E-03	2.02E-02	-1.80
**LOC100130476**	8161.10	2.18E-03	1.00E-02	-1.80
**PELO**	2543.35	2.41E-05	2.08E-04	-1.80
**EIF4EBP2**	24174.23	1.29E-08	2.34E-07	-1.80
**NME2**	18426.80	1.56E-05	1.41E-04	-1.80
**MCCC1**	6912.13	1.90E-07	2.71E-06	-1.80
**GMPS**	10527.32	1.58E-07	2.29E-06	-1.80
**NDUFV2-AS1**	1759.79	2.33E-04	1.49E-03	-1.80
**C6orf48**	21723.88	2.81E-06	3.07E-05	-1.80
**MIEF1**	7940.66	9.38E-08	1.43E-06	-1.80
**TRMT12**	1601.00	4.21E-04	2.46E-03	-1.80
**ATL1**	648.87	1.49E-02	4.86E-02	-1.80
**TFDP2**	7972.87	1.70E-06	1.96E-05	-1.80
**LCP1**	187247.17	9.47E-09	1.77E-07	-1.81
**SARAF**	301153.35	2.11E-13	8.49E-12	-1.81
**GRK3**	1300.87	3.63E-03	1.53E-02	-1.81
**AGTPBP1**	13000.13	1.05E-09	2.31E-08	-1.81

**PEMT**	2606.72	1.57E-05	1.42E-04	-1.81
**SLC11A1**	866.97	2.02E-04	1.31E-03	-1.81
**IL7R**	241365.56	2.03E-08	3.53E-07	-1.81
**TMEM123**	179503.38	5.82E-12	1.87E-10	-1.81
**PCBP1-AS1**	916.23	2.08E-06	2.35E-05	-1.81
**VNN2**	5348.17	2.43E-07	3.40E-06	-1.81
**ENO1**	94999.40	2.63E-15	1.35E-13	-1.81
**CREM**	6927.45	1.66E-03	7.94E-03	-1.82
**TOMM7**	36453.67	2.63E-04	1.65E-03	-1.82
**PTPN12**	2315.54	2.38E-03	1.08E-02	-1.82
**RBMXL1**	12666.45	1.32E-19	1.01E-17	-1.82
**MIR181A1HG**	499.54	5.13E-03	2.04E-02	-1.82
**CIDECP**	1148.60	2.49E-04	1.57E-03	-1.82
**RSC1A1**	748.86	6.46E-03	2.46E-02	-1.82
**SGTB**	12923.87	4.58E-08	7.41E-07	-1.82
**EIF3H**	40904.87	8.12E-10	1.82E-08	-1.82
**GSPT2**	3316.32	1.93E-07	2.77E-06	-1.83
**KLHL12**	6120.60	2.96E-06	3.21E-05	-1.83
**RCSD1**	45218.88	9.81E-14	4.15E-12	-1.83
**AGO1**	3962.56	9.42E-07	1.16E-05	-1.83
**REL**	9256.07	3.34E-07	4.54E-06	-1.83
**PRRC1**	10931.51	6.19E-11	1.73E-09	-1.83
**ST7**	1261.88	1.48E-03	7.22E-03	-1.83
**F5**	7573.82	6.47E-04	3.56E-03	-1.83
**ARHGEF12**	1257.95	9.17E-03	3.29E-02	-1.83
**C6orf106**	5128.48	5.71E-12	1.84E-10	-1.83
**CCNK**	6079.35	1.15E-04	8.06E-04	-1.84
**CRYL1**	5095.54	4.87E-07	6.39E-06	-1.84
**CERS6**	9771.86	1.20E-05	1.12E-04	-1.84
**RPL18**	94057.24	1.63E-06	1.89E-05	-1.84
**NUP93**	8156.83	1.07E-09	2.34E-08	-1.84
**RPL8**	127776.39	1.59E-05	1.44E-04	-1.84
**CYB5R4**	8058.78	9.47E-11	2.54E-09	-1.84
**YES1**	1483.19	7.95E-05	5.84E-04	-1.84
**HDAC3**	11481.30	9.37E-22	8.45E-20	-1.84
**DDX19A**	9702.66	4.00E-12	1.33E-10	-1.84
**ME2**	10413.09	1.48E-07	2.16E-06	-1.84
**UBE2F**	12187.40	1.01E-13	4.26E-12	-1.84
**MTHFD2L**	4128.95	2.84E-06	3.09E-05	-1.84
**CD38**	3035.07	9.17E-03	3.29E-02	-1.85
**SETD3**	7582.16	1.30E-09	2.80E-08	-1.85
**AGBL2**	1137.86	1.51E-03	7.34E-03	-1.85
**INTS10**	18514.57	8.80E-09	1.65E-07	-1.85
**PASK**	9302.24	3.00E-04	1.84E-03	-1.85
**SNORA63**	415.34	6.85E-03	2.59E-02	-1.85
**SETD7**	2990.98	3.26E-04	1.98E-03	-1.85
**OSER1**	14703.41	5.56E-06	5.65E-05	-1.85
**LOC101928069**	318.48	3.86E-03	1.62E-02	-1.85
**MIOS**	4798.38	6.04E-07	7.77E-06	-1.86
**ZNF818P**	1042.64	6.71E-03	2.54E-02	-1.86
**CDCA7L**	8075.71	1.32E-04	9.05E-04	-1.86
**IKZF2**	4252.51	4.74E-03	1.91E-02	-1.86
**PSAP**	96834.10	1.86E-05	1.65E-04	-1.86
**ANKRD55**	7487.53	2.05E-04	1.33E-03	-1.86
**ICA1L**	4669.96	4.21E-05	3.35E-04	-1.86
**HERPUD1**	31668.68	2.09E-07	2.96E-06	-1.86
**RABGGTB**	6628.41	1.14E-10	3.02E-09	-1.86

**EXT1**	1637.64	2.85E-07	3.95E-06	-1.86
**MAN1A2**	21940.18	8.18E-08	1.27E-06	-1.86
**RALGPS2**	2637.48	5.24E-04	2.97E-03	-1.87
**CANX**	60513.42	1.45E-07	2.13E-06	-1.87
**ZNF417**	3054.95	1.14E-04	8.03E-04	-1.87
**ADNP2**	5568.06	2.07E-10	5.24E-09	-1.87
**CMTM8**	2974.64	2.39E-03	1.08E-02	-1.87
**GPR3**	254.28	9.92E-03	3.51E-02	-1.87
**FOXP1-AS1**	1077.05	3.02E-06	3.27E-05	-1.87
**LOC102723885**	536.06	2.03E-03	9.44E-03	-1.87
**IGIP**	1122.09	7.91E-03	2.91E-02	-1.87
**TRAK2**	17263.89	4.25E-08	6.90E-07	-1.88
**RPL27**	72076.09	2.04E-06	2.31E-05	-1.88
**FCRL3**	2587.81	6.42E-03	2.45E-02	-1.88
**FAM209A**	375.38	1.13E-02	3.91E-02	-1.88
**BAG5**	13614.27	3.87E-09	7.79E-08	-1.88
**RPL11**	89776.31	2.03E-07	2.89E-06	-1.88
**IPO5**	11817.39	3.39E-07	4.61E-06	-1.88
**LINC01259**	1122.94	5.00E-04	2.85E-03	-1.88
**DUSP16**	6182.41	3.18E-07	4.36E-06	-1.88
**PHLPP1**	331.33	5.80E-03	2.26E-02	-1.88
**BCL2L11**	4122.71	1.58E-09	3.38E-08	-1.88
**DPYSL2**	7793.00	1.61E-05	1.45E-04	-1.89
**NEK11**	658.64	4.50E-03	1.83E-02	-1.89
**PAPSS2**	887.79	3.43E-03	1.47E-02	-1.89
**PIAS2**	13407.39	8.55E-13	3.12E-11	-1.89
**KPNA5**	5186.12	2.26E-06	2.53E-05	-1.89
**DLAT**	5416.82	5.12E-06	5.25E-05	-1.89
**EEF1E1-BLOC1S5**	5695.48	2.21E-06	2.48E-05	-1.89
**IL24**	984.63	4.59E-04	2.66E-03	-1.89
**CBR3-AS1**	1381.78	1.15E-05	1.07E-04	-1.89
**TSC22D3**	217302.77	6.01E-06	6.08E-05	-1.89
**ZNF24**	36945.94	1.64E-13	6.71E-12	-1.89
**SLC24A1**	1461.47	5.40E-04	3.05E-03	-1.89
**HBP1**	27966.30	3.42E-08	5.64E-07	-1.89
**UBR7**	7471.56	2.62E-07	3.65E-06	-1.90
**NOMO1**	10787.32	1.91E-05	1.69E-04	-1.90
**NOC2LP2**	746.30	2.84E-04	1.76E-03	-1.90
**PTBP3**	28389.71	3.23E-10	7.86E-09	-1.90
**CELF2-AS1**	1391.83	3.48E-03	1.48E-02	-1.90
**CENPB**	1038.44	1.13E-03	5.77E-03	-1.90
**ANXA9**	185.94	9.70E-03	3.44E-02	-1.90
**GDF9**	478.42	1.06E-03	5.45E-03	-1.90
**IGF1R**	7024.00	8.30E-06	8.10E-05	-1.90
**LTA4H**	13692.44	3.76E-22	3.50E-20	-1.90
**HLA-DPA1**	40688.53	1.25E-02	4.24E-02	-1.90
**ZNF605**	3464.85	2.12E-04	1.37E-03	-1.91
**ANO6**	12807.64	6.41E-13	2.38E-11	-1.91
**ALYREF**	3667.41	4.99E-08	8.01E-07	-1.91
**PRDM1**	25862.14	4.51E-05	3.56E-04	-1.91
**SLC25A36**	18262.18	1.29E-13	5.35E-12	-1.91
**TMEM107**	9009.27	6.74E-04	3.70E-03	-1.92
**ZNF438**	1465.37	1.03E-04	7.32E-04	-1.92
**NBPF25P**	8891.94	6.34E-17	3.95E-15	-1.92
**PER1**	6632.40	2.99E-04	1.84E-03	-1.92
**LOC100288798**	609.95	6.39E-03	2.44E-02	-1.92
**RACK1**	147869.43	8.82E-12	2.77E-10	-1.92

**ZNF8**	1766.30	1.15E-02	3.96E-02	-1.92
**TCTN2**	556.82	4.43E-03	1.81E-02	-1.92
**PLAUR**	1694.39	1.35E-02	4.50E-02	-1.92
**CSNK2A3**	2616.07	2.35E-10	5.88E-09	-1.92
**RASA2**	30213.30	2.04E-12	7.09E-11	-1.93
**JARID2**	9029.06	4.38E-21	3.70E-19	-1.93
**LSM11**	642.84	1.47E-03	7.18E-03	-1.93
**EIF3K**	28223.16	2.90E-06	3.15E-05	-1.93
**ANKRD36BP2**	2008.38	5.21E-05	4.06E-04	-1.93
**CBL**	11816.31	8.91E-14	3.81E-12	-1.93
**GALNT11**	9916.26	8.76E-15	4.32E-13	-1.93
**ZNF354C**	4043.47	2.72E-05	2.30E-04	-1.93
**DCAF4**	3242.34	8.40E-05	6.13E-04	-1.93
**KLRB1**	11423.28	9.14E-04	4.80E-03	-1.94
**Sep-02**	38694.17	1.83E-09	3.83E-08	-1.94
**SAE1**	17199.02	1.95E-13	7.84E-12	-1.94
**PCBP2**	23637.85	1.99E-11	5.96E-10	-1.94
**HSP90AB1**	65960.56	7.41E-18	4.95E-16	-1.94
**HIST2H2AC**	1833.48	9.39E-03	3.35E-02	-1.94
**EPHX1**	641.56	1.05E-03	5.40E-03	-1.95
**ARHGEF6**	28522.61	9.73E-08	1.48E-06	-1.95
**TRMT10A**	908.17	3.87E-04	2.30E-03	-1.96
**DENND6A**	12457.07	4.98E-10	1.16E-08	-1.96
**SLC25A15**	1836.61	5.21E-05	4.06E-04	-1.96
**TAOK1**	13600.45	6.37E-08	1.00E-06	-1.96
**RNF126P1**	501.82	7.46E-04	4.04E-03	-1.96
**AHCY**	4023.04	1.23E-08	2.26E-07	-1.96
**APH1A**	8817.10	3.62E-04	2.16E-03	-1.96
**RGS2**	14922.45	2.52E-04	1.59E-03	-1.97
**RNF157-AS1**	422.32	9.78E-03	3.47E-02	-1.97
**SUSD1**	3644.58	5.89E-03	2.29E-02	-1.97
**TBC1D31**	2408.33	9.61E-04	5.01E-03	-1.97
**RCAN3**	34630.40	1.45E-12	5.13E-11	-1.97
**NDUFS1**	8466.51	5.42E-18	3.66E-16	-1.97
**FBXO36**	639.94	9.87E-03	3.50E-02	-1.97
**SCPEP1**	5559.07	7.56E-15	3.75E-13	-1.97
**LOC100506985**	5126.87	2.41E-07	3.38E-06	-1.97
**LAP3**	7933.48	1.24E-12	4.42E-11	-1.98
**LINC01578**	42737.44	2.31E-08	3.95E-07	-1.98
**CXorf40A**	4434.12	1.13E-12	4.04E-11	-1.98
**DPH3P1**	200.88	1.32E-02	4.44E-02	-1.98
**SNHG6**	24637.21	2.08E-05	1.82E-04	-1.98
**MTCP1**	679.29	1.17E-03	5.89E-03	-1.98
**C8orf44-SGK3**	3764.56	3.38E-04	2.04E-03	-1.98
**AOAH**	4470.42	3.02E-04	1.85E-03	-1.98
**ARRDC4**	1451.14	1.82E-03	8.62E-03	-1.98
**ZNF525**	7910.05	1.21E-09	2.62E-08	-1.98
**LINC00467**	402.38	4.24E-03	1.74E-02	-1.99
**PTPRJ**	9870.67	3.69E-14	1.63E-12	-1.99
**EXTL2**	1000.95	8.32E-03	3.04E-02	-1.99
**USP28**	3564.62	7.94E-05	5.84E-04	-1.99
**MREG**	1359.98	2.49E-04	1.57E-03	-1.99
**RPS12**	100437.37	2.10E-08	3.66E-07	-1.99
**FNDC3B**	5737.40	7.00E-11	1.92E-09	-1.99
**ADGRE1**	1460.80	1.30E-02	4.37E-02	-1.99
**GRPEL1**	10064.80	4.08E-09	8.15E-08	-1.99
**RNF144B**	1581.75	3.46E-03	1.48E-02	-2.00

**AAR2**	7192.85	4.60E-11	1.31E-09	-2.00
**SSH1**	4130.91	3.32E-05	2.72E-04	-2.00
**SNORA48**	271.87	1.67E-03	8.01E-03	-2.00
**PIK3CA**	13648.54	3.48E-16	1.96E-14	-2.00
**ARF4-AS1**	330.31	3.90E-03	1.63E-02	-2.00
**CA6**	825.13	4.42E-03	1.80E-02	-2.00
**YPEL2**	10729.66	1.92E-10	4.91E-09	-2.00
**PILRA**	1357.74	2.21E-03	1.01E-02	-2.00
**NUDT17**	742.29	1.14E-03	5.81E-03	-2.01
**SLC16A6**	1446.86	9.70E-04	5.05E-03	-2.01
**ZNF121**	3540.55	6.13E-07	7.87E-06	-2.01
**BNIP1**	1602.19	3.87E-05	3.11E-04	-2.01
**SLC41A3**	3302.12	2.17E-09	4.53E-08	-2.02
**DSTNP2**	849.05	2.34E-06	2.61E-05	-2.02
**PPTC7**	6949.02	2.42E-09	4.98E-08	-2.02
**HIST1H1E**	11957.86	8.34E-03	3.04E-02	-2.02
**RBM12**	10683.36	1.96E-14	9.09E-13	-2.02
**PMAIP1**	5893.20	7.00E-06	6.96E-05	-2.03
**SLC2A9**	295.67	1.48E-02	4.84E-02	-2.03
**RPUSD2**	3761.35	4.14E-10	9.88E-09	-2.03
**SLC15A4**	5423.43	2.27E-06	2.53E-05	-2.03
**STAG3L5P**	628.07	5.82E-03	2.26E-02	-2.03
**ENOX2**	4616.96	1.51E-04	1.02E-03	-2.04
**HLA-F-AS1**	1555.12	5.38E-06	5.48E-05	-2.04
**TBC1D3P1-DHX40P1**	1144.13	4.72E-04	2.71E-03	-2.04
**DHX9**	26704.77	2.03E-17	1.32E-15	-2.04
**LOC101927780**	720.40	5.28E-04	2.99E-03	-2.05
**SSH2**	15842.44	1.31E-11	4.01E-10	-2.05
**FAM21EP**	641.45	1.10E-02	3.80E-02	-2.05
**ALDH18A1**	7512.69	7.69E-07	9.63E-06	-2.05
**SERPINE2**	1596.07	4.53E-03	1.84E-02	-2.05
**PTDSS1**	15553.26	3.20E-23	3.22E-21	-2.05
**CDC37L1-AS1**	411.19	1.66E-03	7.96E-03	-2.06
**GINS2**	612.62	9.92E-03	3.51E-02	-2.06
**GNE**	2435.10	5.93E-08	9.39E-07	-2.06
**CBX3P2**	1171.61	9.86E-04	5.11E-03	-2.07
**LOC107984974**	22064.60	1.75E-08	3.08E-07	-2.07
**THUMPD2**	3583.53	1.27E-07	1.89E-06	-2.07
**PRDX6**	20914.10	4.85E-08	7.82E-07	-2.07
**HAR1B**	662.41	8.00E-03	2.94E-02	-2.07
**RNF32**	487.81	3.77E-03	1.59E-02	-2.07
**CDNF**	280.44	3.55E-03	1.51E-02	-2.07
**CCDC17**	223.82	5.42E-03	2.14E-02	-2.07
**ERGIC3**	12688.55	1.88E-12	6.61E-11	-2.07
**FASTKD5**	8743.40	9.17E-13	3.32E-11	-2.08
**RNF139**	26172.69	4.18E-10	9.96E-09	-2.08
**ZFAND2A**	7488.53	4.78E-06	4.95E-05	-2.08
**ZNF256**	2015.31	4.39E-05	3.48E-04	-2.08
**TLDC2**	1924.35	1.69E-07	2.43E-06	-2.08
**HLA-H**	36079.39	4.75E-10	1.12E-08	-2.08
**BTG3**	3811.90	1.33E-05	1.22E-04	-2.08
**FLNB**	9826.54	1.35E-07	2.00E-06	-2.09
**TPI1P2**	547.80	4.90E-06	5.06E-05	-2.09
**CSDE1**	95167.82	7.30E-13	2.70E-11	-2.09
**RLIM**	10615.44	3.70E-24	4.05E-22	-2.09
**ZBTB37**	968.60	1.51E-04	1.02E-03	-2.09
**CD209**	475.27	1.30E-03	6.45E-03	-2.09

**FRS2**	5529.95	2.37E-09	4.88E-08	-2.10
**ESYT2**	30857.16	1.63E-06	1.89E-05	-2.10
**TOX**	5499.92	2.04E-07	2.91E-06	-2.10
**PTCH2**	310.21	3.81E-03	1.60E-02	-2.10
**LOC101927755**	470.24	5.35E-05	4.15E-04	-2.10
**GALNT1**	11472.21	1.38E-10	3.62E-09	-2.11
**AIF1**	10507.83	1.79E-04	1.19E-03	-2.11
**HIST1H4C**	36240.24	5.55E-03	2.18E-02	-2.12
**RAB20**	456.45	9.05E-04	4.76E-03	-2.12
**NDRG3**	12090.91	1.90E-14	8.82E-13	-2.12
**WAC**	37829.41	9.74E-28	1.34E-25	-2.12
**LOC101927178**	368.78	7.52E-04	4.07E-03	-2.13
**CCDC39**	253.31	6.76E-03	2.56E-02	-2.13
**LOC100287049**	794.24	6.61E-06	6.60E-05	-2.13
**TMEM110-MUSTN1**	659.04	4.78E-03	1.92E-02	-2.13
**PSMD13**	16190.00	1.59E-23	1.62E-21	-2.14
**UHMK1**	34429.42	9.04E-15	4.43E-13	-2.14
**EYS**	549.54	8.12E-05	5.94E-04	-2.14
**HIST1H1C**	8543.46	2.39E-04	1.52E-03	-2.15
**RPL22L1**	4335.35	4.90E-06	5.06E-05	-2.15
**MLLT10P1**	394.88	1.15E-02	3.97E-02	-2.15
**PDE9A**	702.61	5.44E-04	3.06E-03	-2.15
**ADPGK-AS1**	577.68	7.06E-05	5.29E-04	-2.16
**LINC01215**	3092.00	9.91E-07	1.21E-05	-2.16
**ATXN1L**	3231.59	5.57E-09	1.09E-07	-2.16
**PIPSL**	4509.86	2.11E-27	2.84E-25	-2.16
**NRIP1**	14547.52	4.06E-09	8.12E-08	-2.16
**MRFAP1**	22695.20	3.34E-15	1.70E-13	-2.17
**RBM26-AS1**	596.91	8.97E-04	4.73E-03	-2.17
**LINC00544**	158.36	4.62E-03	1.87E-02	-2.17
**TCP11L2**	9640.60	4.87E-12	1.59E-10	-2.17
**CCDC25**	9630.73	1.48E-18	1.04E-16	-2.17
**PELI1**	13885.36	1.03E-10	2.75E-09	-2.18
**RHOBTB3**	531.35	7.41E-03	2.76E-02	-2.18
**RBM43**	3829.66	7.63E-05	5.65E-04	-2.18
**MED1**	11118.42	5.20E-09	1.02E-07	-2.18
**LOC646214**	2530.23	2.33E-07	3.28E-06	-2.18
**LOC101927365**	699.11	7.07E-05	5.29E-04	-2.18
**CLP1**	3946.65	1.12E-05	1.05E-04	-2.18
**SDAD1P1**	2446.07	1.51E-07	2.20E-06	-2.19
**GUSBP2**	573.83	3.18E-04	1.94E-03	-2.19
**ATP1B1**	755.60	2.66E-05	2.25E-04	-2.19
**ZNF890P**	948.12	2.75E-03	1.22E-02	-2.19
**NELL2**	28133.77	1.45E-10	3.79E-09	-2.19
**EPB41L4A**	2124.88	1.11E-05	1.04E-04	-2.19
**NPIPA7**	1543.78	6.96E-05	5.23E-04	-2.20
**SNHG25**	2493.31	1.50E-03	7.32E-03	-2.20
**CDR2**	19468.50	8.94E-15	4.39E-13	-2.20
**LRRC8C-DT**	896.61	4.65E-06	4.83E-05	-2.20
**CTNND1**	1394.84	5.27E-03	2.09E-02	-2.20
**SMG8**	4330.33	5.54E-08	8.82E-07	-2.20
**PRKAR2B**	576.75	1.42E-02	4.69E-02	-2.20
**PAK1**	3105.20	1.32E-06	1.57E-05	-2.21
**BCRP3**	272.75	1.17E-02	4.01E-02	-2.21
**CMIP**	844.16	3.00E-04	1.84E-03	-2.21
**LINC01281**	586.09	9.28E-03	3.32E-02	-2.22
**CORO1C**	4887.36	8.48E-05	6.17E-04	-2.22

**SCOC-AS1**	185.01	3.15E-03	1.37E-02	-2.22
**LEPR**	809.43	3.17E-03	1.37E-02	-2.22
**MPP1**	3838.76	5.84E-09	1.13E-07	-2.22
**SNHG5**	14090.26	2.98E-03	1.31E-02	-2.24
**BCL9**	2022.32	7.96E-05	5.84E-04	-2.25
**MAML3**	559.62	5.71E-04	3.19E-03	-2.25
**LINC01891**	1121.81	1.31E-05	1.21E-04	-2.25
**NFKBIA**	101279.71	3.50E-06	3.73E-05	-2.25
**CD9**	787.49	6.67E-03	2.53E-02	-2.25
**EGFL8**	447.55	1.28E-02	4.33E-02	-2.25
**SNX29P1**	280.81	4.82E-03	1.93E-02	-2.25
**STON1**	262.66	1.44E-02	4.74E-02	-2.25
**KLF4**	978.02	1.47E-02	4.82E-02	-2.26
**MCTP1**	1171.89	1.38E-04	9.40E-04	-2.26
**MT1E**	608.09	5.46E-03	2.15E-02	-2.26
**CDKN2AIP**	13213.95	8.23E-24	8.62E-22	-2.26
**RMI2**	975.95	1.14E-04	8.00E-04	-2.26
**LINC01619**	1459.89	7.95E-05	5.84E-04	-2.26
**FAM239A**	912.84	4.39E-03	1.80E-02	-2.26
**KDM4A**	2585.83	3.32E-06	3.56E-05	-2.27
**PIK3R1**	58965.45	4.74E-13	1.82E-11	-2.27
**MAP3K8**	4163.33	3.33E-04	2.02E-03	-2.27
**NACA2**	14595.19	1.90E-11	5.75E-10	-2.28
**CCDC144CP**	2259.31	1.51E-02	4.91E-02	-2.28
**ITGA1**	572.88	8.21E-03	3.00E-02	-2.29
**FBL**	10240.47	6.73E-10	1.53E-08	-2.29
**UBOX5-AS1**	165.70	3.45E-03	1.47E-02	-2.29
**MYLIP**	29240.30	8.29E-17	5.09E-15	-2.30
**MED13L**	12656.32	1.04E-19	8.15E-18	-2.30
**AZIN1-AS1**	175.66	3.22E-03	1.39E-02	-2.31
**PMEL**	313.47	3.64E-04	2.18E-03	-2.31
**EPB41L4A-AS1**	5168.37	3.25E-10	7.89E-09	-2.31
**CHURC1-FNTB**	1249.75	9.88E-05	7.06E-04	-2.31
**SMIM3**	2433.87	2.51E-08	4.25E-07	-2.31
**PECAM1**	3369.22	1.26E-04	8.74E-04	-2.31
**GAS5**	15200.06	7.17E-11	1.97E-09	-2.31
**HIST1H1D**	18531.80	8.80E-03	3.18E-02	-2.32
**EIF3L**	41826.80	1.25E-22	1.20E-20	-2.32
**CCNT1**	12087.49	1.89E-14	8.81E-13	-2.32
**CD244**	1355.15	2.13E-04	1.37E-03	-2.32
**SNHG1**	12290.83	3.69E-11	1.07E-09	-2.32
**PDE4D**	9590.67	6.46E-06	6.47E-05	-2.32
**LY86**	1272.56	7.09E-03	2.66E-02	-2.32
**ODC1**	25954.70	1.66E-10	4.31E-09	-2.33
**RPS5**	71448.31	1.32E-14	6.27E-13	-2.33
**LOC407835**	1701.07	2.62E-08	4.41E-07	-2.33
**PTPRS**	1734.63	4.67E-03	1.89E-02	-2.33
**CABP4**	890.28	3.43E-05	2.80E-04	-2.33
**LOC101929089**	166.30	1.88E-03	8.84E-03	-2.33
**LINC02035**	810.78	2.63E-05	2.23E-04	-2.33
**EIF3D**	23354.89	8.85E-23	8.65E-21	-2.34
**PA2G4P4**	5193.14	2.62E-21	2.27E-19	-2.34
**PRPH2**	215.12	6.01E-03	2.32E-02	-2.35
**TSSK4**	239.44	1.05E-03	5.42E-03	-2.35
**NPIPB11**	1700.54	3.61E-05	2.93E-04	-2.35
**PGD**	5112.33	3.89E-10	9.34E-09	-2.35
**REPS2**	175.15	1.00E-02	3.53E-02	-2.36

**ZNF844**	2410.14	9.43E-04	4.92E-03	-2.36
**RASGEF1B**	1877.04	3.55E-05	2.88E-04	-2.36
**SULT1B1**	2002.72	4.18E-08	6.78E-07	-2.36
**CXCR4**	153620.32	3.04E-07	4.18E-06	-2.37
**Mar-01**	2046.88	3.12E-03	1.36E-02	-2.37
**CTBP2**	909.61	2.48E-05	2.12E-04	-2.38
**KIAA1147**	15751.53	1.62E-14	7.64E-13	-2.38
**HCG4B**	2559.40	2.41E-05	2.07E-04	-2.38
**FGR**	3702.07	7.64E-03	2.83E-02	-2.39
**EP400NL**	866.14	2.08E-06	2.35E-05	-2.39
**C5orf66**	948.25	2.13E-05	1.86E-04	-2.39
**USP30**	3112.69	2.30E-06	2.57E-05	-2.40
**MYH10**	1126.36	1.32E-06	1.57E-05	-2.40
**RPSAP9**	899.82	1.07E-06	1.30E-05	-2.40
**ACTG1P20**	3988.73	8.92E-12	2.80E-10	-2.40
**GMCL1P1**	680.92	4.65E-07	6.13E-06	-2.41
**QKI**	10821.91	2.58E-18	1.78E-16	-2.42
**NOMO3**	7398.47	2.87E-07	3.98E-06	-2.42
**LOC105377102**	218.99	3.26E-03	1.41E-02	-2.42
**RIBC1**	142.37	1.22E-02	4.15E-02	-2.42
**CCNY**	11836.38	2.38E-12	8.16E-11	-2.42
**RPL13AP3**	5175.89	1.04E-09	2.30E-08	-2.42
**RNF130**	9703.26	6.67E-06	6.65E-05	-2.42
**PPIH**	5083.06	4.52E-11	1.29E-09	-2.43
**MPEG1**	9172.96	8.35E-03	3.05E-02	-2.43
**CEMIP**	149.32	1.96E-03	9.17E-03	-2.43
**NCBP2-AS1**	212.05	2.26E-03	1.03E-02	-2.43
**SCARNA9L**	301.10	2.63E-05	2.23E-04	-2.44
**SNORA64**	194.03	2.86E-04	1.77E-03	-2.45
**GBP1P1**	467.89	1.99E-03	9.29E-03	-2.46
**RNF212**	739.26	1.78E-04	1.18E-03	-2.46
**ANP32C**	284.79	2.75E-06	3.01E-05	-2.47
**LOC105370489**	361.28	4.28E-05	3.39E-04	-2.47
**SESTD1**	2100.60	1.53E-03	7.43E-03	-2.48
**LINC00441**	137.28	4.71E-03	1.90E-02	-2.48
**NPIPA5**	1705.99	1.75E-07	2.51E-06	-2.48
**USP7**	14141.00	6.07E-38	1.42E-35	-2.48
**CDK5R1**	5338.40	2.89E-05	2.42E-04	-2.48
**GTF2IP1**	10567.09	1.34E-13	5.55E-12	-2.49
**LOC728024**	884.09	1.53E-06	1.79E-05	-2.49
**LOC339539**	433.26	2.42E-05	2.08E-04	-2.49
**CTSH**	5231.86	4.86E-10	1.14E-08	-2.50
**SYK**	5055.04	1.99E-03	9.26E-03	-2.50
**RPS10P7**	2516.82	2.21E-14	1.01E-12	-2.50
**VAV3**	2739.44	1.86E-06	2.13E-05	-2.50
**FLJ31104**	349.47	6.59E-03	2.50E-02	-2.51
**ATP5L2**	1474.09	3.34E-06	3.58E-05	-2.51
**LOC101928617**	1187.89	2.48E-07	3.47E-06	-2.52
**MGP**	329.78	3.28E-04	2.00E-03	-2.52
**NCF2**	2679.06	9.79E-04	5.08E-03	-2.52
**PTENP1**	3315.82	5.31E-23	5.27E-21	-2.52
**LAT2**	1261.28	2.17E-04	1.40E-03	-2.52
**MOK**	236.50	5.27E-03	2.09E-02	-2.53
**CIB2**	530.13	1.43E-02	4.71E-02	-2.53
**GJA9-MYCBP**	889.92	9.39E-05	6.75E-04	-2.53
**TMED6**	156.65	5.86E-03	2.28E-02	-2.54
**SCCPDH**	2123.06	8.48E-06	8.24E-05	-2.54

**SIK2**	670.78	1.43E-06	1.69E-05	-2.54
**UMODL1**	163.06	7.14E-03	2.68E-02	-2.54
**HLA-DRA**	76347.60	7.68E-03	2.84E-02	-2.55
**LOC101930114**	172.17	6.40E-03	2.45E-02	-2.55
**TAF13**	192.08	1.15E-02	3.94E-02	-2.56
**KLHL11**	5711.56	1.47E-09	3.14E-08	-2.58
**SNORD10**	357.11	2.84E-05	2.38E-04	-2.58
**SNORA57**	289.70	1.54E-03	7.48E-03	-2.59
**SNORD141A**	4867.73	8.66E-14	3.72E-12	-2.59
**SNORD141B**	4867.73	8.66E-14	3.72E-12	-2.59
**RNF103**	7722.30	6.84E-15	3.42E-13	-2.60
**EIF5AL1**	8043.48	5.07E-08	8.12E-07	-2.60
**LOC653653**	2383.44	5.98E-10	1.37E-08	-2.60
**SNORA73B**	124.16	1.09E-02	3.78E-02	-2.60
**SMG1P7**	1203.20	1.04E-06	1.26E-05	-2.61
**AXL**	948.44	8.77E-05	6.36E-04	-2.62
**GOLM1**	889.13	1.28E-04	8.83E-04	-2.63
**TRIO**	409.34	7.31E-03	2.73E-02	-2.63
**LAMB1**	129.62	2.05E-03	9.52E-03	-2.63
**TGFBI**	6904.58	1.39E-03	6.85E-03	-2.63
**LOC101927974**	350.93	5.83E-03	2.27E-02	-2.63
**HIST1H2AK**	509.38	4.04E-04	2.38E-03	-2.63
**TXNIP**	1166700.26	2.65E-19	1.97E-17	-2.64
**ATP10D**	739.08	1.74E-03	8.28E-03	-2.64
**GRK6P1**	1599.54	8.00E-11	2.17E-09	-2.65
**MEX3C**	4388.25	5.01E-11	1.42E-09	-2.65
**GLUD2**	6898.46	2.88E-16	1.65E-14	-2.66
**TP53INP2**	253.29	9.17E-03	3.29E-02	-2.66
**POC1B-GALNT4**	546.32	1.08E-02	3.76E-02	-2.68
**PIP5K1P1**	197.66	9.55E-03	3.40E-02	-2.68
**MGST1**	598.71	3.31E-03	1.42E-02	-2.69
**CD109**	281.23	2.41E-03	1.09E-02	-2.70
**LOC401127**	180.31	5.01E-04	2.86E-03	-2.70
**MYO5C**	238.86	1.05E-02	3.68E-02	-2.71
**LOC440311**	6299.78	8.84E-21	7.26E-19	-2.72
**ADGRE2**	1693.88	7.60E-11	2.07E-09	-2.72
**PLTP**	157.41	7.89E-03	2.90E-02	-2.72
**ABCA10**	222.31	9.42E-04	4.92E-03	-2.72
**ZBTB20-AS4**	119.16	7.51E-03	2.79E-02	-2.72
**HLA-DRB5**	16900.97	6.15E-03	2.37E-02	-2.72
**GTF2IP4**	9277.44	5.15E-14	2.27E-12	-2.72
**SNHG4**	152.77	3.78E-03	1.59E-02	-2.74
**PSPH**	843.32	3.62E-03	1.53E-02	-2.74
**LOC105369340**	146.07	1.53E-02	4.97E-02	-2.75
**LINC01176**	247.85	3.25E-03	1.40E-02	-2.75
**SDHAP3**	1746.10	1.75E-18	1.22E-16	-2.75
**CYP2T1P**	297.01	1.17E-03	5.89E-03	-2.76
**NPIPB2**	756.34	2.32E-04	1.48E-03	-2.77
**CD83**	3599.30	1.06E-05	9.99E-05	-2.77
**ANKDD1B**	217.07	1.04E-02	3.66E-02	-2.77
**LOC105375218**	255.14	1.49E-03	7.26E-03	-2.77
**FAM49A**	1972.60	1.25E-02	4.25E-02	-2.78
**MYOF**	331.18	8.25E-03	3.02E-02	-2.78
**PCBP2-OT1**	271.03	7.58E-05	5.63E-04	-2.79
**SNORA20**	74.41	6.08E-03	2.35E-02	-2.79
**ERRFI1**	304.87	4.67E-03	1.89E-02	-2.80
**TNFSF13**	1354.61	7.19E-04	3.91E-03	-2.81

**TVP23C**	4502.74	1.21E-11	3.74E-10	-2.81
**LOC103908605**	175.44	1.99E-03	9.28E-03	-2.81
**CD33**	897.47	4.64E-03	1.88E-02	-2.82
**KLRF1**	1679.07	1.01E-02	3.54E-02	-2.83
**SNORA67**	404.76	2.49E-05	2.13E-04	-2.83
**OSTCP1**	494.46	1.63E-07	2.36E-06	-2.83
**GTF2H2C**	710.27	2.50E-03	1.12E-02	-2.83
**FOXN3-AS1**	95.40	1.46E-02	4.79E-02	-2.84
**LOC103091866**	1799.13	8.19E-29	1.19E-26	-2.84
**SPON2**	1643.93	5.30E-03	2.10E-02	-2.84
**SBF1P1**	245.64	1.83E-04	1.21E-03	-2.84
**MIR6723**	4805.90	2.88E-14	1.29E-12	-2.85
**ZNF286B**	1000.41	6.77E-11	1.87E-09	-2.86
**TBC1D16**	126.09	1.41E-03	6.92E-03	-2.87
**CLPB**	1163.75	7.04E-07	8.89E-06	-2.87
**SCARNA9**	2295.94	3.94E-07	5.29E-06	-2.87
**TIMP2**	487.28	1.15E-03	5.84E-03	-2.87
**SNORA9**	207.18	2.06E-03	9.55E-03	-2.88
**MTMR11**	417.57	2.40E-04	1.52E-03	-2.90
**BTBD3**	471.77	1.43E-02	4.72E-02	-2.90
**LOC100129534**	224.01	3.89E-04	2.30E-03	-2.91
**SNORD104**	226.87	5.77E-05	4.43E-04	-2.91
**NAPA-AS1**	464.07	4.88E-08	7.85E-07	-2.91
**COL4A4**	250.19	1.83E-04	1.21E-03	-2.92
**DACH1**	323.03	1.17E-02	4.02E-02	-2.93
**LOC107985033**	139.57	3.26E-03	1.41E-02	-2.94
**USF1**	2701.28	3.91E-05	3.14E-04	-2.94
**USP6**	475.18	1.90E-05	1.68E-04	-2.95
**FGD6**	229.49	6.13E-03	2.36E-02	-2.95
**ANP32AP1**	574.52	5.56E-10	1.29E-08	-2.96
**CASC1**	183.36	1.26E-03	6.28E-03	-2.96
**DCAF13P3**	584.07	7.16E-07	9.02E-06	-2.96
**GJB7**	334.87	1.60E-03	7.72E-03	-2.98
**KCTD12**	1470.60	1.15E-04	8.06E-04	-2.99
**LOC100506321**	295.50	1.46E-06	1.72E-05	-2.99
**RPL34-AS1**	197.85	3.73E-03	1.57E-02	-2.99
**SYNJ2BP-COX16**	1835.42	7.60E-06	7.47E-05	-2.99
**APOOP5**	238.49	1.99E-05	1.75E-04	-3.00
**KDELR3**	225.53	4.99E-03	1.99E-02	-3.00
**FLT3**	2324.17	6.71E-03	2.54E-02	-3.01
**FCRL6**	3108.29	1.31E-02	4.40E-02	-3.01
**RPL31P11**	481.90	5.20E-06	5.32E-05	-3.02
**MICALCL**	175.06	8.93E-03	3.22E-02	-3.02
**LOC100303749**	145.91	2.32E-03	1.05E-02	-3.03
**RNF138P1**	3681.95	5.95E-21	4.98E-19	-3.03
**SRGAP2D**	521.66	1.05E-05	9.94E-05	-3.04
**UQCRBP1**	6346.18	8.13E-15	4.02E-13	-3.05
**SNORD31**	159.39	6.91E-05	5.20E-04	-3.05
**LOC101928865**	138.21	4.67E-03	1.89E-02	-3.06
**LMO7-AS1**	158.38	8.17E-05	5.97E-04	-3.06
**UQCRHL**	5009.57	1.10E-13	4.63E-12	-3.06
**MAP2K4P1**	887.81	3.92E-13	1.52E-11	-3.07
**MKRN9P**	621.52	9.49E-11	2.54E-09	-3.07
**GTF2IP23**	82.47	3.96E-03	1.65E-02	-3.07
**CA14**	240.82	4.30E-04	2.50E-03	-3.07
**KANTR**	13518.53	3.79E-08	6.20E-07	-3.08
**LAMA2**	170.73	7.23E-03	2.71E-02	-3.08

**KLF9**	9314.03	6.84E-10	1.55E-08	-3.08
**TNFAIP3**	117964.56	8.58E-06	8.33E-05	-3.08
**GALNT5**	329.48	3.61E-03	1.53E-02	-3.08
**PLBD1**	2614.98	4.19E-03	1.73E-02	-3.09
**AHNAK2**	577.90	2.18E-03	1.00E-02	-3.10
**ERMN**	2719.55	2.87E-07	3.98E-06	-3.10
**PEAK1**	1054.90	1.46E-03	7.15E-03	-3.12
**MOB3B**	209.14	6.19E-03	2.38E-02	-3.13
**CERKL**	393.60	1.40E-04	9.53E-04	-3.14
**TYROBP**	6381.03	9.80E-04	5.08E-03	-3.14
**ACER2**	123.23	1.58E-03	7.65E-03	-3.15
**ANKRD19P**	7056.37	8.03E-19	5.76E-17	-3.16
**TFEC**	371.27	1.13E-02	3.91E-02	-3.16
**RSPH9**	81.92	7.76E-03	2.87E-02	-3.16
**LOC101928663**	104.80	3.18E-03	1.38E-02	-3.16
**GAS6-AS1**	229.60	6.00E-04	3.34E-03	-3.16
**LOC440700**	80.66	7.82E-03	2.88E-02	-3.16
**ZNF442**	491.13	6.71E-04	3.69E-03	-3.17
**SNORA25**	126.20	8.11E-04	4.33E-03	-3.17
**FBXL2**	162.60	1.30E-03	6.45E-03	-3.19
**NME8**	343.69	1.51E-02	4.90E-02	-3.19
**SULT1A2**	348.54	1.40E-05	1.28E-04	-3.19
**NAPSA**	822.99	1.04E-04	7.40E-04	-3.19
**CREB5**	373.51	1.47E-02	4.82E-02	-3.20
**METRNL**	476.19	2.32E-03	1.05E-02	-3.20
**LINC01136**	114.98	1.09E-03	5.58E-03	-3.21
**LINC02332**	105.52	4.84E-03	1.94E-02	-3.21
**POLR2J4**	4359.88	3.07E-24	3.40E-22	-3.21
**FCGR2A**	1123.54	7.06E-03	2.65E-02	-3.22
**A2M**	1784.21	2.28E-08	3.90E-07	-3.24
**STAG3**	573.47	2.68E-05	2.26E-04	-3.24
**HNRNPA1P33**	733.08	1.72E-14	8.08E-13	-3.24
**HIST1H4E**	1428.97	4.72E-06	4.89E-05	-3.26
**SCARNA3**	57.69	1.16E-02	3.99E-02	-3.26
**PEX12**	676.73	7.63E-05	5.65E-04	-3.26
**MILR1**	261.79	2.30E-03	1.04E-02	-3.26
**RP9P**	650.80	7.27E-10	1.64E-08	-3.26
**HIST1H3I**	181.35	1.54E-02	4.99E-02	-3.27
**ZNF462**	183.97	3.49E-03	1.49E-02	-3.27
**SNORA71D**	51.90	1.26E-02	4.26E-02	-3.28
**KIT**	158.57	6.31E-03	2.42E-02	-3.28
**RNU12**	111.02	9.85E-03	3.49E-02	-3.29
**PLK2**	623.62	1.61E-04	1.08E-03	-3.29
**POLR2J2**	6440.63	7.42E-07	9.32E-06	-3.30
**SNORD13E**	258.60	4.86E-07	6.38E-06	-3.30
**EDARADD**	11046.90	2.55E-29	3.81E-27	-3.30
**ATP8B4**	198.46	6.41E-03	2.45E-02	-3.30
**ZBED6**	933.90	1.00E-04	7.15E-04	-3.31
**LOC110091777**	101.14	9.17E-04	4.82E-03	-3.31
**LAIR2**	1384.47	4.17E-03	1.72E-02	-3.33
**CHI3L2**	3233.07	8.77E-10	1.95E-08	-3.34
**TBC1D12**	193.35	1.84E-03	8.70E-03	-3.34
**SPATA21**	65.82	1.34E-02	4.47E-02	-3.34
**STIL**	411.86	8.57E-04	4.54E-03	-3.34
**MYL5**	477.72	1.55E-05	1.41E-04	-3.34
**ZNF492**	410.93	2.21E-05	1.93E-04	-3.35
**CTAGE11P**	317.52	1.21E-09	2.62E-08	-3.36

**GDF7**	1501.12	6.74E-07	8.53E-06	-3.36
**GAS5-AS1**	245.85	5.83E-05	4.47E-04	-3.36
**PABPC3**	10786.81	4.52E-39	1.11E-36	-3.36
**LGALS9C**	91.92	4.96E-03	1.98E-02	-3.36
**SMAD1**	511.83	7.27E-03	2.72E-02	-3.37
**ADGRA3**	636.75	8.37E-05	6.11E-04	-3.37
**TALAM1**	453.91	1.21E-04	8.42E-04	-3.39
**PTCSC1**	425.96	3.50E-05	2.85E-04	-3.40
**EFCAB14-AS1**	163.54	7.71E-05	5.69E-04	-3.40
**HNRNPA3P1**	1468.86	1.43E-23	1.48E-21	-3.40
**PZP**	498.60	1.03E-05	9.76E-05	-3.42
**ZEB2**	4079.59	7.03E-05	5.28E-04	-3.42
**LINC00886**	183.51	3.12E-03	1.36E-02	-3.43
**CCDC144A**	2887.59	4.29E-03	1.76E-02	-3.43
**SNORA104**	457.07	2.09E-05	1.83E-04	-3.44
**NPIPA2**	767.16	8.90E-14	3.81E-12	-3.44
**LINC01635**	156.12	1.75E-03	8.34E-03	-3.45
**EBF1**	69.62	7.40E-03	2.76E-02	-3.45
**LOC100128568**	87.37	4.19E-03	1.73E-02	-3.45
**SCARNA10**	395.15	3.06E-04	1.87E-03	-3.46
**LINC00926**	176.87	2.41E-04	1.53E-03	-3.46
**LOC148709**	102.05	7.77E-03	2.87E-02	-3.47
**MIR6891**	317.52	1.10E-04	7.74E-04	-3.47
**HNRNPCL4**	447.29	9.49E-12	2.96E-10	-3.48
**LOC105369632**	102.50	1.24E-03	6.19E-03	-3.48
**LSP1P3**	757.71	6.98E-13	2.59E-11	-3.50
**BUB1B**	268.76	3.74E-03	1.57E-02	-3.50
**UBE2E4P**	1704.65	4.21E-21	3.57E-19	-3.51
**SNORD13F**	144.47	5.35E-04	3.02E-03	-3.51
**PTPRK**	281.89	3.14E-03	1.36E-02	-3.52
**TCL1A**	5529.98	2.15E-03	9.90E-03	-3.52
**KLHL13**	274.59	7.49E-03	2.79E-02	-3.53
**DGUOK-AS1**	317.09	4.87E-06	5.03E-05	-3.53
**OSBPL10**	234.25	9.73E-04	5.06E-03	-3.53
**BIVM-ERCC5**	1802.75	2.23E-03	1.02E-02	-3.56
**UBXN10-AS1**	203.18	6.31E-04	3.48E-03	-3.56
**SIRPB2**	323.11	2.50E-03	1.12E-02	-3.56
**HSP90B2P**	8545.93	1.01E-80	1.48E-77	-3.56
**PIN1P1**	287.55	5.82E-10	1.34E-08	-3.57
**RBM47**	667.55	1.35E-02	4.50E-02	-3.57
**BCAT1**	712.66	3.13E-04	1.91E-03	-3.57
**CC2D2A**	190.04	6.27E-05	4.77E-04	-3.58
**TMED10P1**	2933.02	3.30E-16	1.87E-14	-3.58
**CACHD1**	864.51	1.32E-05	1.22E-04	-3.59
**ARHGAP24**	342.14	6.71E-03	2.54E-02	-3.60
**MIR4691**	64.37	8.54E-03	3.10E-02	-3.60
**JDP2**	225.26	5.49E-04	3.08E-03	-3.62
**GSTM2P1**	604.54	5.02E-07	6.58E-06	-3.63
**FRMD3**	195.20	9.24E-03	3.31E-02	-3.64
**SPATA13-AS1**	104.35	2.85E-04	1.77E-03	-3.64
**HAVCR1P1**	101.89	3.57E-04	2.14E-03	-3.64
**SNORA53**	82.17	8.36E-03	3.05E-02	-3.65
**WBP11P1**	1436.84	2.09E-31	3.42E-29	-3.65
**TST**	141.91	6.41E-03	2.45E-02	-3.66
**PDE7B**	384.40	8.10E-04	4.33E-03	-3.66
**SNORA63B**	352.07	6.62E-07	8.40E-06	-3.66
**CLIC4**	549.84	9.73E-04	5.06E-03	-3.67

**RPL19P12**	5313.59	6.37E-27	8.07E-25	-3.67
**CD8B**	555.53	1.19E-03	5.98E-03	-3.67
**PRNCR1**	3405.76	1.05E-22	1.02E-20	-3.67
**DSE**	8720.24	2.69E-26	3.29E-24	-3.67
**TMCC3**	748.96	1.53E-02	4.98E-02	-3.67
**LOC606724**	3867.58	1.46E-20	1.18E-18	-3.68
**RPL23P8**	7078.04	2.88E-21	2.47E-19	-3.68
**LILRA5**	330.58	3.44E-03	1.47E-02	-3.68
**TLR8**	213.17	1.11E-02	3.85E-02	-3.69
**ST5**	114.97	5.19E-03	2.06E-02	-3.70
**CCDC144NL-AS1**	219.26	5.43E-07	7.06E-06	-3.70
**SLC3A1**	147.22	5.41E-04	3.05E-03	-3.70
**DNAH11**	47.43	1.36E-02	4.54E-02	-3.71
**LOC643802**	156.24	1.43E-02	4.73E-02	-3.73
**MKRN2OS**	81.35	2.62E-03	1.17E-02	-3.73
**BANK1**	669.31	1.30E-02	4.38E-02	-3.75
**DPP6**	90.21	2.86E-03	1.26E-02	-3.77
**TAF1L**	569.29	1.67E-08	2.96E-07	-3.78
**CFAP45**	231.57	2.69E-04	1.68E-03	-3.80
**RNF216-IT1**	59.56	1.15E-02	3.95E-02	-3.80
**ZSCAN9**	186.64	1.45E-03	7.08E-03	-3.82
**KCNS1**	224.57	1.84E-04	1.22E-03	-3.82
**C5AR1**	269.81	2.64E-04	1.65E-03	-3.84
**LANCL1-AS1**	180.46	1.16E-04	8.11E-04	-3.85
**SKP1P2**	8592.75	4.25E-35	7.83E-33	-3.85
**TCAF2P1**	1538.91	1.40E-03	6.89E-03	-3.85
**ZNF185**	742.85	4.15E-04	2.43E-03	-3.87
**LOC101927402**	211.18	5.63E-03	2.20E-02	-3.88
**BTBD9-AS1**	87.43	2.34E-03	1.06E-02	-3.88
**EZR-AS1**	46.26	1.25E-02	4.25E-02	-3.89
**IGBP1P1**	735.17	6.05E-20	4.79E-18	-3.91
**MIR3661**	230.81	2.40E-06	2.68E-05	-3.91
**CLMP**	143.84	4.78E-05	3.76E-04	-3.92
**YBX3P1**	134.50	3.51E-05	2.85E-04	-3.97
**PTTG3P**	220.27	6.65E-08	1.05E-06	-3.98
**CSTA**	977.00	1.89E-03	8.90E-03	-3.99
**SNORA27**	105.99	2.34E-04	1.49E-03	-3.99
**FAM85B**	124.40	1.12E-04	7.87E-04	-4.00
**HIST1H2AL**	229.40	1.67E-04	1.12E-03	-4.01
**SNORD17**	69.38	3.61E-03	1.53E-02	-4.02
**MIR663AHG**	1194.83	1.36E-15	7.23E-14	-4.02
**LOC344967**	95.43	1.27E-04	8.78E-04	-4.02
**PPP1R2P3**	5923.88	2.18E-52	8.67E-50	-4.04
**CCND2-AS1**	118.30	2.10E-04	1.36E-03	-4.04
**LINC02044**	109.73	5.46E-03	2.15E-02	-4.05
**IFITM3**	17339.72	2.60E-04	1.63E-03	-4.05
**HLA-DQB2**	1426.37	8.09E-04	4.32E-03	-4.06
**LOC554249**	202.97	2.65E-04	1.66E-03	-4.07
**LYN**	5638.60	1.32E-04	9.09E-04	-4.08
**GPX2**	90.13	7.85E-04	4.22E-03	-4.08
**HSPA7**	82.22	1.27E-02	4.28E-02	-4.08
**TPH1**	66.99	7.74E-03	2.86E-02	-4.08
**SLFN14**	209.02	9.69E-05	6.94E-04	-4.09
**SNORA8**	179.84	3.47E-06	3.70E-05	-4.10
**HORMAD2-AS1**	86.47	1.41E-03	6.92E-03	-4.10
**SRRM2-AS1**	53.23	1.31E-02	4.41E-02	-4.12
**FPGT-TNNI3K**	284.99	3.91E-04	2.31E-03	-4.12

**GPR27**	273.76	1.96E-03	9.18E-03	-4.13
**SCARNA5**	334.03	1.15E-05	1.07E-04	-4.14
**AR**	140.38	1.50E-02	4.89E-02	-4.14
**EDRF1-AS1**	92.99	3.41E-04	2.06E-03	-4.15
**CD19**	86.96	9.88E-03	3.50E-02	-4.15
**RPEL1**	495.61	2.47E-13	9.85E-12	-4.16
**SHE**	164.91	3.56E-04	2.14E-03	-4.19
**SNORA24**	187.37	2.48E-06	2.75E-05	-4.20
**LINC02399**	363.84	1.48E-04	1.00E-03	-4.21
**PLS3**	204.39	7.08E-03	2.66E-02	-4.21
**TTC23L**	51.44	4.51E-03	1.84E-02	-4.21
**VTRNA1-1**	144.32	2.31E-03	1.05E-02	-4.21
**GNG3**	54.37	1.39E-02	4.60E-02	-4.21
**RPS18P9**	20484.24	1.23E-32	2.10E-30	-4.22
**MIR616**	66.49	4.45E-03	1.82E-02	-4.23
**ANKRD18B**	446.92	5.04E-05	3.94E-04	-4.26
**THNSL2**	108.15	9.27E-03	3.32E-02	-4.29
**RBAK-RBAKDN**	85.79	1.17E-02	4.02E-02	-4.29
**FCGR2C**	556.41	1.32E-03	6.52E-03	-4.30
**USP32P3**	302.60	2.53E-03	1.13E-02	-4.30
**CD300E**	799.34	9.23E-03	3.31E-02	-4.32
**DIP2A-IT1**	87.03	1.90E-03	8.93E-03	-4.34
**MSRB3**	239.47	2.81E-03	1.24E-02	-4.34
**HIST1H2BF**	408.10	5.14E-06	5.27E-05	-4.34
**GOLGA6D**	49.16	1.48E-02	4.84E-02	-4.34
**TNFSF12-TNFSF13**	653.15	8.89E-07	1.10E-05	-4.35
**ENC1**	1269.17	3.49E-04	2.10E-03	-4.35
**TTK**	144.85	7.17E-03	2.69E-02	-4.35
**LOC100289495**	77.01	6.06E-03	2.34E-02	-4.37
**SNORA72**	110.16	1.56E-04	1.05E-03	-4.37
**SNORD11**	100.74	5.10E-04	2.90E-03	-4.38
**METTL7A**	1285.49	6.71E-11	1.86E-09	-4.41
**SERHL**	361.26	1.96E-06	2.22E-05	-4.43
**LOC105379393**	67.78	3.70E-03	1.56E-02	-4.43
**SNORA4**	354.47	1.24E-08	2.27E-07	-4.45
**NCAM1**	213.17	1.61E-03	7.75E-03	-4.46
**LINC01399**	168.82	1.67E-05	1.50E-04	-4.48
**ZBTB20-AS3**	64.01	3.53E-03	1.50E-02	-4.49
**CD36**	3895.86	1.05E-04	7.43E-04	-4.49
**LOC644762**	1991.66	1.38E-35	2.70E-33	-4.54
**CYP1B1**	303.56	7.37E-03	2.75E-02	-4.54
**LOC101926943**	557.25	6.42E-22	5.89E-20	-4.55
**SIGLEC6**	197.47	5.85E-03	2.28E-02	-4.55
**GCOM1**	441.76	5.92E-05	4.53E-04	-4.55
**KSR2**	90.99	6.38E-03	2.44E-02	-4.55
**SNORA6**	61.62	1.27E-03	6.33E-03	-4.56
**CTAGE9**	307.67	3.53E-07	4.79E-06	-4.57
**SLC1A2**	98.11	3.45E-03	1.47E-02	-4.58
**UBE2Q1-AS1**	40.83	1.30E-02	4.38E-02	-4.58
**THSD1**	234.25	3.59E-04	2.15E-03	-4.60
**ANKRD65**	76.73	7.75E-03	2.86E-02	-4.61
**LRRC37A6P**	585.28	2.59E-13	1.03E-11	-4.61
**TAPT1-AS1**	5403.22	3.42E-22	3.22E-20	-4.61
**SLC16A14**	129.66	3.60E-03	1.53E-02	-4.62
**SNORD25**	44.98	7.43E-03	2.77E-02	-4.62
**RNF103-CHMP3**	128.17	3.60E-05	2.92E-04	-4.63
**MIR497HG**	479.83	2.74E-18	1.88E-16	-4.65

**FAM35DP**	1318.89	1.19E-16	7.20E-15	-4.67
**MAGOH2P**	976.12	1.03E-21	9.20E-20	-4.68
**NFIL3**	1381.02	5.60E-13	2.12E-11	-4.68
**MTRNR2L3**	467.22	3.22E-05	2.65E-04	-4.69
**CD163**	449.63	1.26E-02	4.26E-02	-4.71
**BEND6**	210.60	2.25E-03	1.03E-02	-4.72
**ARHGEF38**	173.32	2.75E-08	4.62E-07	-4.72
**IGF2BP3**	80.14	3.92E-03	1.64E-02	-4.73
**NBPF19**	1113.54	2.57E-19	1.92E-17	-4.74
**C1QTNF3-AMACR**	236.42	1.52E-03	7.39E-03	-4.75
**LINC01694**	47.48	6.91E-03	2.61E-02	-4.76
**MIR3917**	82.60	3.31E-04	2.01E-03	-4.76
**GOLGA8J**	115.20	2.04E-03	9.46E-03	-4.77
**EMBP1**	8349.98	3.16E-24	3.48E-22	-4.77
**CFLAR-AS1**	157.35	1.87E-06	2.14E-05	-4.79
**GSTA4**	162.03	6.94E-04	3.79E-03	-4.79
**LINC02362**	32.07	1.22E-02	4.16E-02	-4.79
**MAFB**	516.65	8.51E-05	6.19E-04	-4.81
**PANDAR**	1038.19	1.14E-24	1.29E-22	-4.81
**VSTM1**	55.23	1.29E-02	4.34E-02	-4.81
**LIMS3**	1677.28	2.52E-34	4.37E-32	-4.83
**LIMS4**	1677.28	2.52E-34	4.37E-32	-4.83
**SNORA70**	943.03	5.24E-24	5.63E-22	-4.83
**SNORD110**	61.07	4.07E-04	2.40E-03	-4.83
**SNORA21**	53.42	2.92E-03	1.28E-02	-4.84
**LOC339529**	59.79	2.67E-03	1.19E-02	-4.84
**RPS14P3**	34325.63	1.76E-36	3.81E-34	-4.84
**QPCT**	391.11	4.64E-05	3.65E-04	-4.85
**CSNK1A1L**	1018.62	6.27E-24	6.69E-22	-4.85
**LOC101927809**	51.69	3.36E-03	1.44E-02	-4.85
**TMEM232**	86.46	5.59E-03	2.19E-02	-4.85
**MIR3671**	38.39	1.27E-02	4.30E-02	-4.88
**TMCC1-AS1**	54.22	6.02E-03	2.33E-02	-4.89
**SNORD102**	38.76	8.28E-03	3.02E-02	-4.90
**KCNMB3**	544.85	1.72E-09	3.64E-08	-4.90
**PGAM4**	3464.58	2.06E-43	5.90E-41	-4.91
**LINC00266-3**	60.41	1.06E-03	5.46E-03	-4.91
**ORM2**	34.53	9.52E-03	3.39E-02	-4.92
**NMUR1**	177.83	5.74E-04	3.21E-03	-4.94
**SNORA26**	112.15	2.44E-05	2.09E-04	-4.94
**SGMS2**	136.70	4.63E-03	1.87E-02	-4.94
**RASA4B**	1426.41	3.53E-11	1.03E-09	-4.96
**PAPOLB**	321.51	1.81E-09	3.81E-08	-5.00
**SETSIP**	1156.74	5.73E-29	8.43E-27	-5.00
**LINC01374**	121.00	5.97E-03	2.31E-02	-5.01
**LOC729080**	128.14	2.11E-06	2.37E-05	-5.01
**FRG1DP**	397.95	3.14E-08	5.21E-07	-5.01
**GPX3**	129.06	9.62E-04	5.01E-03	-5.01
**MIR570**	60.05	2.70E-03	1.20E-02	-5.02
**GZMH**	11869.24	1.13E-03	5.75E-03	-5.02
**MIR5047**	3301.23	3.85E-41	1.00E-38	-5.03
**NLRC4**	115.93	7.85E-04	4.22E-03	-5.04
**SOX4**	2795.30	1.23E-06	1.47E-05	-5.05
**TTC3P1**	2181.91	4.46E-41	1.15E-38	-5.07
**ATP8B5P**	1062.68	1.20E-26	1.50E-24	-5.08
**B3GNT7**	77.35	2.41E-03	1.09E-02	-5.08
**RPL13AP6**	11024.85	2.68E-36	5.65E-34	-5.10

**LINC01623**	87.72	3.94E-04	2.33E-03	-5.11
**SNORA12**	1367.88	4.00E-08	6.52E-07	-5.12
**RAD51L3-RFFL**	501.24	7.00E-03	2.64E-02	-5.13
**SNORD94**	61.83	1.68E-03	8.02E-03	-5.14
**SAXO2**	328.56	1.63E-03	7.81E-03	-5.14
**RN7SL3**	19076.80	1.62E-20	1.30E-18	-5.15
**SORD2P**	322.17	9.37E-11	2.52E-09	-5.15
**ZNF729**	69.12	4.64E-04	2.67E-03	-5.17
**RSPH10B**	102.49	2.28E-04	1.46E-03	-5.18
**RSPH10B2**	102.49	2.28E-04	1.46E-03	-5.18
**LOC101928008**	48.81	8.11E-03	2.98E-02	-5.18
**SH3GL1P2**	181.59	1.83E-07	2.62E-06	-5.19
**CD160**	686.52	2.01E-09	4.20E-08	-5.19
**BLOC1S5-TXNDC5**	1731.02	7.91E-19	5.69E-17	-5.20
**FAM183BP**	74.64	4.86E-03	1.95E-02	-5.20
**OPRM1**	294.61	1.32E-05	1.22E-04	-5.22
**LOC100335030**	490.80	1.07E-09	2.36E-08	-5.24
**OPA1-AS1**	55.41	1.13E-03	5.75E-03	-5.24
**CRYM**	323.55	3.72E-03	1.57E-02	-5.26
**FPR2**	79.97	3.90E-03	1.63E-02	-5.26
**PLA2G4A**	80.00	9.95E-03	3.51E-02	-5.31
**AKR7A2P1**	277.92	5.60E-10	1.29E-08	-5.32
**NPIPA3**	781.24	1.35E-19	1.03E-17	-5.33
**SLC7A7**	807.52	4.02E-06	4.24E-05	-5.34
**SNORA33**	124.80	7.43E-06	7.31E-05	-5.35
**SH3D19**	574.03	1.19E-11	3.70E-10	-5.36
**CD14**	2492.17	1.84E-06	2.12E-05	-5.40
**LOC339666**	45.54	7.32E-03	2.73E-02	-5.40
**CCR9**	418.91	1.70E-05	1.52E-04	-5.42
**LOC441454**	4645.68	1.02E-25	1.21E-23	-5.44
**SLC10A1**	61.69	5.75E-04	3.21E-03	-5.49
**GRAPL**	442.28	2.07E-06	2.34E-05	-5.49
**ARMCX1**	218.41	1.02E-03	5.26E-03	-5.50
**VEGFA**	224.50	3.38E-03	1.45E-02	-5.51
**HBA2**	1219.46	1.13E-03	5.74E-03	-5.53
**C15orf38-AP3S2**	325.04	1.69E-03	8.08E-03	-5.58
**SNORA10B**	62.18	5.37E-04	3.03E-03	-5.58
**TOP1P1**	685.35	4.47E-10	1.06E-08	-5.59
**MIP**	37.53	1.18E-02	4.03E-02	-5.60
**DPYD-AS1**	50.33	1.22E-03	6.10E-03	-5.61
**RACGAP1P**	121.03	5.06E-07	6.62E-06	-5.62
**MYL6B**	1202.80	1.37E-15	7.27E-14	-5.67
**SNORD83B**	33.19	6.91E-03	2.61E-02	-5.68
**FRG1JP**	4742.22	2.26E-32	3.83E-30	-5.69
**ERVFRD-1**	53.99	4.30E-03	1.76E-02	-5.72
**TGIF2-C20orf24**	1426.09	5.15E-08	8.24E-07	-5.72
**MTRNR2L8**	100971.41	3.62E-22	3.38E-20	-5.75
**ASB12**	136.95	6.60E-06	6.59E-05	-5.76
**DCLRE1CP1**	84.81	3.04E-04	1.86E-03	-5.76
**HLA-J**	6744.69	1.77E-15	9.26E-14	-5.76
**LINC01132**	83.22	9.53E-04	4.98E-03	-5.76
**RAB6D**	1571.71	2.16E-57	1.22E-54	-5.77
**IGF2BP2**	51.85	3.61E-03	1.53E-02	-5.78
**GOLGA8M**	276.07	9.71E-05	6.95E-04	-5.79
**TLN2**	72.73	1.88E-03	8.84E-03	-5.80
**SNORA78**	65.48	1.82E-04	1.20E-03	-5.81
**SH2D1B**	685.53	1.07E-02	3.75E-02	-5.82

**CYP2F1**	79.33	2.29E-04	1.47E-03	-5.83
**RPGRIP1**	77.90	1.84E-03	8.69E-03	-5.85
**GNAZ**	135.67	6.20E-07	7.91E-06	-5.91
**AGAP12P**	459.79	5.31E-06	5.42E-05	-5.92
**FAM87A**	265.88	1.64E-10	4.26E-09	-5.96
**LRRK2**	721.50	7.02E-04	3.83E-03	-5.96
**HLA-L**	7013.23	6.15E-17	3.84E-15	-5.99
**LOC101928119**	33.76	1.05E-02	3.67E-02	-6.00
**P2RY1**	83.75	2.04E-03	9.48E-03	-6.02
**CCDC181**	28.34	1.50E-02	4.90E-02	-6.04
**LOC400684**	53.47	1.82E-03	8.60E-03	-6.05
**ALDH1A1**	190.77	2.00E-03	9.33E-03	-6.05
**TREM1**	474.96	1.15E-04	8.06E-04	-6.06
**ERBB3**	98.67	2.83E-04	1.75E-03	-6.07
**LOC494127**	253.88	3.46E-11	1.01E-09	-6.09
**SNORD35A**	78.02	9.88E-04	5.12E-03	-6.09
**H3F3C**	39076.99	1.64E-48	5.42E-46	-6.10
**CBWD6**	4266.16	2.70E-39	6.76E-37	-6.16
**INTS4P1**	654.54	1.51E-06	1.77E-05	-6.17
**CPS1**	56.38	6.21E-03	2.39E-02	-6.18
**SRD5A1P1**	71.33	1.86E-04	1.23E-03	-6.19
**LOC105378683**	28.87	5.88E-03	2.28E-02	-6.19
**LOC641746**	52.28	1.79E-03	8.50E-03	-6.20
**LOC101927272**	40.03	5.13E-03	2.04E-02	-6.21
**CRYBA1**	28.48	1.16E-02	3.97E-02	-6.22
**NBPF7**	112.96	3.83E-06	4.06E-05	-6.22
**LOC102724050**	55.01	5.73E-04	3.20E-03	-6.22
**PNMA6F**	40.93	6.02E-03	2.33E-02	-6.23
**SNORA32**	84.72	2.23E-04	1.43E-03	-6.24
**EIF3IP1**	349.58	1.23E-18	8.68E-17	-6.26
**MIR3916**	40.97	1.65E-03	7.91E-03	-6.31
**LINC01191**	85.58	2.31E-04	1.48E-03	-6.33
**SNORA44**	54.62	1.27E-03	6.31E-03	-6.35
**SNORD21**	49.34	1.23E-03	6.16E-03	-6.36
**NT5C1B-RDH14**	146.00	3.76E-04	2.24E-03	-6.36
**FGFBP2**	4629.06	5.15E-04	2.92E-03	-6.37
**LOC644936**	8093.86	1.39E-55	6.75E-53	-6.41
**CDA**	179.91	7.87E-04	4.23E-03	-6.42
**HCG14**	61.21	7.22E-04	3.93E-03	-6.43
**RIN2**	50.60	7.78E-03	2.87E-02	-6.45
**KCNE2**	30.78	1.26E-02	4.26E-02	-6.45
**TUBA1C**	11057.66	6.93E-49	2.43E-46	-6.47
**LOC110091776**	49.46	4.26E-03	1.75E-02	-6.48
**RPL17-C18orf32**	13581.56	1.54E-12	5.44E-11	-6.49
**ANXA2P1**	1650.32	4.08E-28	5.70E-26	-6.50
**P2RY2**	78.32	5.69E-03	2.22E-02	-6.54
**ANP32D**	28.82	1.45E-02	4.77E-02	-6.56
**UBE2F-SCLY**	504.96	2.84E-06	3.09E-05	-6.58
**LOC400682**	78.13	5.88E-03	2.28E-02	-6.59
**LOC441455**	501.23	1.90E-12	6.65E-11	-6.60
**ARPIN**	120.85	1.40E-04	9.57E-04	-6.61
**WFDC21P**	137.25	1.31E-04	9.01E-04	-6.62
**LINC01291**	58.97	5.47E-03	2.15E-02	-6.68
**LOC101928100**	39.68	1.27E-02	4.28E-02	-6.69
**S100A8**	11686.27	5.34E-05	4.15E-04	-6.71
**LOC646030**	78.62	5.49E-05	4.25E-04	-6.74
**MGC4859**	4832.30	5.75E-43	1.62E-40	-6.75

**SNORA3A**	117.67	3.16E-06	3.40E-05	-6.77
**SNORD22**	427.10	5.94E-17	3.72E-15	-6.79
**SNORA28**	179.74	1.28E-09	2.77E-08	-6.82
**MIR7-1**	56.84	1.68E-04	1.12E-03	-6.83
**SNORD116-2**	76.87	6.20E-04	3.43E-03	-6.83
**FAM231A**	38.56	5.57E-03	2.19E-02	-6.85
**SNORD5**	25.68	5.52E-03	2.17E-02	-6.86
**LOC100128164**	276.44	1.81E-07	2.60E-06	-6.86
**EPB42**	44.43	1.51E-03	7.36E-03	-6.88
**LOC100129484**	55.74	1.25E-03	6.24E-03	-6.90
**MIR663A**	442.50	4.11E-15	2.07E-13	-6.91
**MIR3687-1**	69.50	1.66E-04	1.11E-03	-6.91
**MIR3687-2**	69.50	1.66E-04	1.11E-03	-6.91
**ABCC3**	42.19	1.36E-02	4.52E-02	-6.92
**RNU4ATAC**	571.54	5.67E-08	9.01E-07	-6.93
**UBE2NL**	1800.79	3.46E-41	9.18E-39	-6.94
**NDUFC2-KCTD14**	116.18	3.23E-03	1.40E-02	-6.95
**SNORD76**	123.82	4.51E-07	5.98E-06	-6.97
**LOC102723753**	173.13	2.55E-05	2.17E-04	-7.03
**HBB**	2403.07	2.39E-04	1.52E-03	-7.06
**NEUROD2**	181.92	4.91E-04	2.80E-03	-7.07
**SNORD70**	38.98	9.29E-04	4.86E-03	-7.08
**SNORA14B**	48.70	1.09E-03	5.57E-03	-7.09
**SNORD72**	21.39	1.11E-02	3.86E-02	-7.09
**LRRC17**	78.85	1.03E-02	3.62E-02	-7.12
**HLA-DRB6**	6972.82	2.14E-08	3.71E-07	-7.12
**SNORD19**	46.93	8.78E-04	4.64E-03	-7.14
**MIR5690**	90.04	2.22E-05	1.93E-04	-7.16
**SNORD45A**	82.36	1.05E-05	9.99E-05	-7.16
**NCMAP**	31.88	5.57E-03	2.19E-02	-7.16
**VCAN**	4465.85	2.36E-04	1.50E-03	-7.21
**CPEB1-AS1**	449.74	1.12E-19	8.75E-18	-7.25
**ZNF750**	31.96	6.35E-03	2.43E-02	-7.25
**FAM238B**	39.12	2.77E-03	1.22E-02	-7.28
**FNDC5**	26.85	1.50E-02	4.90E-02	-7.30
**MIR186**	20.96	1.27E-02	4.30E-02	-7.31
**SNORD80**	79.63	3.36E-04	2.03E-03	-7.31
**MIR3064**	1066.00	2.83E-24	3.15E-22	-7.38
**ZNF716**	27.08	3.64E-03	1.54E-02	-7.39
**KCNH1**	37.32	3.34E-03	1.43E-02	-7.42
**CSNK1A1P1**	502.10	1.39E-28	1.99E-26	-7.44
**FUNDC2P2**	1019.05	1.57E-27	2.13E-25	-7.44
**LOC101929577**	32.09	1.09E-02	3.78E-02	-7.47
**SNORD13C**	17.21	1.50E-02	4.90E-02	-7.49
**FPR1**	1752.29	1.72E-05	1.53E-04	-7.49
**KLRK1**	2511.69	2.52E-06	2.79E-05	-7.54
**S100A9**	16876.97	1.11E-06	1.34E-05	-7.58
**NRG1**	52.91	1.17E-02	4.02E-02	-7.59
**MITF**	93.38	3.08E-03	1.34E-02	-7.60
**PADI2**	237.70	9.63E-03	3.42E-02	-7.61
**FGFR2**	101.35	3.90E-03	1.63E-02	-7.63
**FAM20C**	47.31	1.31E-02	4.41E-02	-7.64
**MIR7847**	194.47	4.01E-10	9.61E-09	-7.66
**HLA-DQB1-AS1**	77.77	3.59E-04	2.15E-03	-7.66
**NACAP1**	6895.22	4.18E-67	3.18E-64	-7.66
**LAMC1**	54.23	2.64E-03	1.18E-02	-7.71
**C6orf132**	23.21	4.53E-03	1.84E-02	-7.72

**SNORD58C**	82.41	7.16E-06	7.10E-05	-7.73
**SLC24A4**	173.49	2.78E-04	1.73E-03	-7.73
**MIR590**	27.79	6.27E-03	2.41E-02	-7.75
**IGFBPL1**	37.15	3.54E-03	1.50E-02	-7.77
**LINC02363**	22.68	6.43E-03	2.45E-02	-7.79
**SNORD59A**	158.48	1.56E-08	2.79E-07	-7.81
**PGAM1P5**	122.43	2.80E-05	2.36E-04	-7.81
**MAP2**	28.72	8.75E-03	3.17E-02	-7.81
**MASP2**	461.78	5.97E-14	2.62E-12	-7.89
**ANXA2P3**	618.18	2.49E-19	1.87E-17	-7.90
**CCDC38**	48.93	9.00E-04	4.74E-03	-7.90
**ETV3L**	59.85	1.37E-04	9.37E-04	-7.91
**BEX1**	398.25	3.92E-11	1.13E-09	-7.92
**DNMBP-AS1**	28.84	5.09E-03	2.03E-02	-7.92
**TMSB15A**	44.53	4.19E-03	1.73E-02	-7.93
**PYGL**	351.94	2.92E-05	2.44E-04	-7.97
**HBA1**	513.75	5.00E-06	5.15E-05	-7.98
**IMPA1P1**	23.11	1.42E-02	4.69E-02	-7.98
**KREMEN1**	18.74	1.21E-02	4.14E-02	-8.01
**SNORD84**	29.00	4.47E-03	1.82E-02	-8.02
**LOXL4**	64.58	1.02E-03	5.28E-03	-8.04
**DGCR6**	2512.51	4.41E-21	3.71E-19	-8.04
**CYP27A1**	139.11	3.68E-04	2.19E-03	-8.08
**LINC01907**	51.70	3.16E-04	1.93E-03	-8.16
**SNORD4A**	43.60	6.98E-04	3.81E-03	-8.16
**HS3ST3A1**	172.47	2.26E-10	5.67E-09	-8.20
**NAT8L**	30.16	4.49E-03	1.83E-02	-8.25
**GAB1**	182.53	7.78E-06	7.64E-05	-8.25
**HIST1H3B**	29.73	2.08E-03	9.62E-03	-8.26
**LOC101929117**	23.88	4.57E-03	1.86E-02	-8.26
**LOC441155**	4446.51	9.98E-36	2.01E-33	-8.26
**CD300H**	74.49	4.23E-03	1.74E-02	-8.26
**SNORD13J**	579.69	2.21E-09	4.60E-08	-8.27
**SNORD98**	45.76	8.05E-04	4.31E-03	-8.28
**RPLP0P2**	1811.21	1.09E-47	3.54E-45	-8.31
**FLJ44635**	6139.58	2.40E-50	8.73E-48	-8.34
**GPR75-ASB3**	499.98	8.77E-06	8.50E-05	-8.36
**PLG**	184.79	2.64E-06	2.90E-05	-8.37
**IGLL1**	36.01	3.06E-03	1.34E-02	-8.37
**ACOT1**	1098.40	1.13E-15	6.11E-14	-8.37
**TMEM17**	24.15	4.98E-03	1.99E-02	-8.42
**SNORA3B**	118.43	3.23E-07	4.42E-06	-8.43
**SNORD50A**	1767.69	2.15E-08	3.72E-07	-8.44
**ZNF728**	38.59	9.01E-04	4.74E-03	-8.50
**SNORD116-26**	19.89	8.01E-03	2.94E-02	-8.57
**PRSS23**	1614.72	4.61E-04	2.66E-03	-8.59
**JADE3**	38.84	5.97E-03	2.31E-02	-8.59
**MIR4712**	54.74	1.12E-03	5.73E-03	-8.60
**RBP7**	164.22	1.42E-05	1.30E-04	-8.61
**AGAP11**	268.50	5.04E-10	1.17E-08	-8.61
**TNS1**	25.34	1.03E-02	3.62E-02	-8.63
**SNORD50B**	105.96	1.76E-05	1.57E-04	-8.65
**SORBS2**	24.93	4.57E-03	1.86E-02	-8.66
**TMEM178A**	25.32	7.26E-03	2.71E-02	-8.75
**LOC105373378**	19.99	1.10E-02	3.81E-02	-8.76
**LINC00431**	101.54	9.98E-06	9.52E-05	-8.84
**FCRL1**	235.58	1.55E-05	1.40E-04	-8.88

**HBD**	158.80	1.60E-03	7.73E-03	-8.94
**SPX**	57.55	2.92E-03	1.28E-02	-8.96
**SNORD15A**	20.17	1.13E-02	3.91E-02	-8.99
**GBP7**	159.21	7.17E-08	1.12E-06	-9.01
**GEM**	32.33	5.17E-03	2.05E-02	-9.02
**SPRY2**	59.49	6.14E-04	3.41E-03	-9.16
**LOC105372672**	21.21	2.66E-03	1.19E-02	-9.17
**SNORD45B**	66.24	3.12E-05	2.58E-04	-9.19
**HIGD2B**	26.84	2.32E-03	1.05E-02	-9.22
**MSX2**	122.04	4.28E-06	4.49E-05	-9.22
**LOC100129138**	117.27	2.57E-08	4.32E-07	-9.23
**SNORD34**	21.10	9.49E-03	3.38E-02	-9.24
**AFAP1L1**	52.37	3.48E-03	1.48E-02	-9.31
**SNORD46**	140.90	7.37E-05	5.48E-04	-9.36
**PRICKLE1**	60.97	1.24E-03	6.17E-03	-9.41
**SNORD44**	33.86	3.66E-04	2.19E-03	-9.42
**ZNF816-ZNF321P**	107.27	2.90E-03	1.27E-02	-9.42
**NPIPB13**	1827.54	1.11E-14	5.37E-13	-9.44
**PDCD6IPP2**	547.35	5.11E-26	6.17E-24	-9.45
**MID1**	51.85	1.52E-02	4.94E-02	-9.48
**IFIT1B**	53.88	3.41E-03	1.46E-02	-9.51
**METTL21C**	42.18	1.88E-03	8.83E-03	-9.52
**LIN28A**	33.93	1.10E-03	5.63E-03	-9.56
**DPY19L2P1**	22.37	8.19E-03	3.00E-02	-9.59
**SNORD54**	27.58	1.59E-03	7.67E-03	-9.63
**GUCY1B3**	49.68	2.46E-03	1.11E-02	-9.65
**HIST1H2BE**	79.87	3.03E-05	2.52E-04	-9.66
**SPDYE14P**	43.03	3.88E-04	2.30E-03	-9.68
**SPDYE13P**	43.03	3.88E-04	2.30E-03	-9.68
**SPDYE15P**	43.03	3.88E-04	2.30E-03	-9.68
**SNORD19B**	94.20	1.74E-07	2.49E-06	-9.71
**LINC01630**	17.51	9.67E-03	3.43E-02	-9.78
**ATXN8OS**	17.71	9.49E-03	3.38E-02	-9.78
**HOXA5**	17.53	7.03E-03	2.64E-02	-9.79
**MIR1204**	17.63	1.38E-02	4.59E-02	-9.84
**MIR1254-1**	53.13	6.35E-05	4.83E-04	-9.91
**LRRC66**	51.17	1.35E-04	9.26E-04	-9.92
**DDX11L5**	44.24	6.44E-04	3.55E-03	-9.92
**SNORA68**	53.16	3.67E-04	2.19E-03	-9.93
**C18orf15**	55.25	9.70E-04	5.05E-03	-9.97
**LGALS9B**	17.74	1.41E-02	4.66E-02	-9.98
**S100A12**	955.02	3.58E-06	3.81E-05	-9.99
**POU3F2**	17.73	6.46E-03	2.46E-02	-9.99
**NEBL**	73.21	9.16E-04	4.81E-03	-10.01
**GOLGA8G**	29.30	1.95E-03	9.13E-03	-10.02
**LINC02285**	71.89	8.23E-04	4.39E-03	-10.11
**SNORD139**	28.81	1.41E-03	6.93E-03	-10.12
**LINC02289**	44.77	2.89E-04	1.79E-03	-10.12
**GOLGA8S**	167.09	7.62E-09	1.45E-07	-10.12
**LIPE-AS1**	221.34	6.00E-13	2.25E-11	-10.12
**MIR7113**	22.91	2.07E-03	9.59E-03	-10.16
**SNORD36B**	18.37	7.62E-03	2.83E-02	-10.18
**LOC728739**	522.76	1.05E-24	1.19E-22	-10.18
**SNORD36C**	45.75	3.33E-04	2.02E-03	-10.29
**DSG2**	87.24	2.07E-04	1.34E-03	-10.30
**GOLGA8T**	36.34	4.28E-04	2.50E-03	-10.32
**MIR491**	23.70	2.43E-03	1.10E-02	-10.36

**ZNF722P**	18.58	6.49E-03	2.47E-02	-10.36
**THRSP**	29.14	1.84E-03	8.68E-03	-10.37
**ITGA9-AS1**	90.43	1.32E-06	1.57E-05	-10.38
**LIN7A**	56.63	1.26E-03	6.28E-03	-10.43
**RPS15AP10**	3692.18	3.83E-30	5.93E-28	-10.44
**LOC613266**	18.89	1.32E-02	4.43E-02	-10.44
**LINC01119**	44.33	5.26E-03	2.09E-02	-10.45
**RGPD4**	1955.76	4.19E-27	5.36E-25	-10.47
**SLC9A9-AS1**	30.11	2.73E-03	1.21E-02	-10.47
**MIR4426**	7498.67	2.15E-52	8.67E-50	-10.62
**SLC7A11-AS1**	19.14	1.15E-02	3.97E-02	-10.65
**FPR3**	36.71	1.15E-02	3.95E-02	-10.68
**LOC101927604**	37.15	2.38E-03	1.08E-02	-10.70
**LOC646548**	19.25	9.36E-03	3.34E-02	-10.76
**MTRNR2L5**	181.13	2.35E-09	4.86E-08	-10.77
**IGLL5**	603.60	7.82E-12	2.48E-10	-10.77
**SNORD116-15**	19.43	1.28E-02	4.32E-02	-10.78
**ZNF33BP1**	19.35	9.37E-03	3.35E-02	-10.82
**RPSAP52**	19.45	9.51E-03	3.39E-02	-10.86
**CYCSP52**	30.90	7.48E-03	2.79E-02	-10.93
**LOC100132831**	188.55	1.70E-13	6.91E-12	-10.95
**PIGZ**	66.11	1.25E-04	8.66E-04	-10.95
**ADGRD1**	31.66	1.25E-02	4.25E-02	-10.95
**NEGR1**	96.55	1.10E-04	7.75E-04	-10.96
**FAM189A1**	19.73	7.30E-03	2.73E-02	-10.99
**GREB1**	19.56	9.90E-03	3.51E-02	-11.00
**SNORD81**	563.53	3.15E-10	7.68E-09	-11.03
**YWHAEP1**	211.91	1.09E-13	4.57E-12	-11.05
**SNORA2B**	38.80	9.70E-04	5.05E-03	-11.07
**SNORA2C**	31.36	1.07E-03	5.50E-03	-11.08
**SLC25A52**	111.18	6.21E-07	7.91E-06	-11.10
**AKR1C4**	59.11	4.15E-05	3.31E-04	-11.11
**LOC646813**	19.73	9.94E-03	3.51E-02	-11.14
**HLA-G**	13495.48	1.46E-77	1.60E-74	-11.19
**SMOC1**	38.68	3.24E-04	1.97E-03	-11.20
**LOC646938**	25.37	3.15E-03	1.37E-02	-11.22
**PEG3**	26.14	8.74E-03	3.17E-02	-11.25
**OR7E156P**	25.62	7.90E-04	4.24E-03	-11.26
**SCARNA22**	153.81	2.72E-06	2.97E-05	-11.29
**IRAK3**	598.87	3.00E-05	2.50E-04	-11.30
**EFNA2**	26.30	1.32E-02	4.44E-02	-11.33
**MRPS30-DT**	20.30	6.15E-03	2.37E-02	-11.34
**SH3PXD2B**	20.44	3.55E-03	1.51E-02	-11.41
**PTPRR**	20.74	2.88E-03	1.27E-02	-11.46
**CTSL3P**	20.84	2.10E-03	9.69E-03	-11.49
**SMPDL3A**	102.60	3.39E-05	2.78E-04	-11.59
**KRT77**	26.54	5.42E-03	2.14E-02	-11.65
**SNORD89**	450.55	1.32E-09	2.85E-08	-11.66
**SCARNA21**	1008.83	6.33E-24	6.71E-22	-11.77
**SHOX**	26.76	2.18E-03	1.00E-02	-11.78
**TSPAN7**	535.87	1.23E-13	5.15E-12	-11.79
**RMRP**	1457.00	2.72E-22	2.57E-20	-11.79
**SNORD100**	33.43	3.68E-04	2.19E-03	-11.79
**TRAM1L1**	315.51	6.42E-21	5.33E-19	-11.84
**CCDC26**	21.22	1.33E-02	4.44E-02	-11.87
**DDX11L16**	119.53	1.66E-06	1.93E-05	-11.92
**HNRNPKP3**	98.44	3.19E-08	5.29E-07	-11.95

**STEAP4**	67.02	2.95E-05	2.46E-04	-11.96
**SLMO2-ATP5E**	446.40	5.44E-08	8.67E-07	-11.97
**ZNF99**	42.61	6.34E-05	4.82E-04	-11.98
**TM4SF19-TCTEX1D2**	51.38	1.10E-03	5.60E-03	-12.04
**IQGAP3**	21.76	1.45E-02	4.76E-02	-12.07
**PKP4-AS1**	33.94	3.38E-04	2.04E-03	-12.08
**SNORD143**	27.73	9.31E-04	4.87E-03	-12.09
**ACTG1P4**	1188.02	1.20E-52	4.99E-50	-12.13
**SNORD18C**	21.85	4.55E-03	1.85E-02	-12.16
**MTRNR2L4**	187.32	9.13E-10	2.02E-08	-12.18
**SNORD47**	798.89	3.38E-13	1.32E-11	-12.20
**PKHD1L1**	22.07	1.03E-03	5.33E-03	-12.25
**KIR2DL1**	96.80	4.32E-05	3.42E-04	-12.32
**RPL29P2**	127.20	1.45E-09	3.12E-08	-12.34
**LOC100506679**	28.12	4.35E-04	2.53E-03	-12.43
**ANKRD20A8P**	90.93	6.48E-07	8.25E-06	-12.44
**HOXA10**	43.78	4.80E-04	2.75E-03	-12.51
**ARNTL2**	78.25	3.62E-04	2.16E-03	-12.51
**LINC01781**	44.23	2.31E-03	1.05E-02	-12.54
**CASC15**	35.92	1.12E-03	5.73E-03	-12.63
**MECOM**	54.69	4.57E-05	3.60E-04	-12.64
**ZNF860**	105.78	3.43E-08	5.66E-07	-12.71
**AMY2A**	480.36	2.91E-30	4.58E-28	-12.72
**MIR223**	23.06	4.86E-03	1.95E-02	-12.76
**TNFRSF19**	80.07	4.74E-04	2.72E-03	-12.81
**WLS**	23.25	4.75E-03	1.91E-02	-12.85
**SNORD96A**	22.98	1.22E-03	6.11E-03	-12.87
**LIMS3-LOC440895**	130.28	3.34E-05	2.74E-04	-12.97
**NPIPB8**	357.20	3.55E-12	1.19E-10	-13.02
**SUPT20HL2**	29.31	5.06E-04	2.88E-03	-13.03
**FCGR3B**	590.47	8.84E-05	6.41E-04	-13.03
**BVES**	29.94	2.45E-04	1.55E-03	-13.04
**LPL**	23.67	8.38E-03	3.05E-02	-13.09
**LPAR1**	23.62	1.36E-03	6.72E-03	-13.17
**EGR3**	23.62	6.49E-04	3.57E-03	-13.18
**BNC2**	58.58	5.48E-04	3.08E-03	-13.23
**TUBB2B**	543.54	3.75E-18	2.54E-16	-13.25
**KLRC3**	84.70	1.98E-05	1.75E-04	-13.26
**SNORD27**	172.86	1.77E-08	3.11E-07	-13.29
**FAM107A**	24.12	1.09E-02	3.78E-02	-13.37
**OR7D2**	30.40	2.69E-03	1.20E-02	-13.43
**CHRND**	23.99	4.08E-04	2.40E-03	-13.44
**SNORD57**	374.98	7.36E-09	1.40E-07	-13.45
**SNORD36A**	57.05	4.34E-06	4.54E-05	-13.45
**INTS4P2**	514.52	1.80E-26	2.24E-24	-13.45
**SNORD38A**	57.80	8.14E-05	5.96E-04	-13.47
**XRCC6P5**	1032.35	1.09E-63	7.08E-61	-13.48
**KLRD1**	2571.72	7.72E-08	1.20E-06	-13.50
**BST1**	342.60	1.26E-11	3.87E-10	-13.70
**TNXA**	24.58	7.55E-03	2.80E-02	-13.70
**GOLGA8F**	31.65	5.57E-04	3.12E-03	-13.72
**CHEK2P2**	24.72	4.20E-04	2.46E-03	-13.78
**POM121L9P**	65.84	4.79E-05	3.76E-04	-13.78
**ANTXRL**	96.66	6.02E-08	9.52E-07	-13.83
**TMIGD3**	25.02	3.22E-03	1.39E-02	-13.85
**ZNF479**	25.20	4.95E-03	1.98E-02	-14.02
**SCARNA28**	744.72	6.35E-12	2.03E-10	-14.13

**HAS2**	25.73	6.02E-04	3.34E-03	-14.23
**TRIM50**	60.57	1.14E-05	1.06E-04	-14.25
**CATSPERD**	25.67	2.87E-03	1.27E-02	-14.25
**SNORD33**	60.05	1.28E-04	8.80E-04	-14.33
**MIR3198-2**	25.74	1.05E-02	3.68E-02	-14.35
**APOBEC3B**	299.10	1.26E-12	4.50E-11	-14.35
**TBL1Y**	61.48	4.70E-05	3.70E-04	-14.35
**SNORD29**	60.46	5.02E-06	5.16E-05	-14.37
**POTEI**	9586.27	2.79E-49	9.97E-47	-14.37
**MRAS**	40.34	1.68E-04	1.12E-03	-14.38
**RET**	59.97	7.91E-03	2.91E-02	-14.39
**SNORD59B**	73.82	3.59E-06	3.82E-05	-14.49
**ROCK1P1**	1140.48	3.27E-16	1.86E-14	-14.54
**CLGN**	26.65	1.23E-02	4.20E-02	-14.79
**POTEF**	1910.45	8.13E-70	6.77E-67	-14.82
**LINC01356**	51.61	5.07E-06	5.21E-05	-14.98
**MGAM**	26.90	5.93E-03	2.30E-02	-15.09
**NUAK1**	53.23	5.78E-03	2.25E-02	-15.13
**WIPF3**	65.36	2.17E-04	1.40E-03	-15.16
**FEZ1**	137.73	4.26E-05	3.38E-04	-15.19
**KIR2DL4**	42.16	2.22E-03	1.02E-02	-15.24
**CATSPER1**	27.56	1.96E-03	9.15E-03	-15.25
**ZSCAN5B**	68.49	7.33E-06	7.23E-05	-15.28
**TCEAL5**	27.25	4.13E-04	2.42E-03	-15.31
**CXCR1**	27.70	2.94E-03	1.29E-02	-15.40
**MIR4512**	34.78	4.18E-05	3.32E-04	-15.41
**TMEM37**	43.21	2.11E-03	9.75E-03	-15.45
**PGM5**	35.19	5.55E-05	4.28E-04	-15.52
**GOLGA6L3**	54.00	7.22E-06	7.15E-05	-15.57
**NTM**	81.98	2.01E-05	1.77E-04	-15.58
**ALAS2**	27.73	5.49E-03	2.16E-02	-15.64
**CD8B2**	81.38	6.08E-06	6.13E-05	-15.64
**PDHA2**	34.99	5.64E-05	4.35E-04	-15.67
**EFTUD1P1**	207.09	1.05E-14	5.12E-13	-15.77
**LOC101929555**	28.35	8.38E-03	3.05E-02	-15.82
**SCARNA13**	28.61	3.87E-03	1.62E-02	-15.86
**ADGRL2**	36.21	4.63E-04	2.67E-03	-15.92
**SNORD63**	180.96	1.14E-07	1.71E-06	-15.99
**SNORD35B**	115.11	5.16E-09	1.02E-07	-16.00
**VTRNA1-3**	221.80	3.69E-11	1.07E-09	-16.01
**ACTC1**	36.70	1.30E-04	8.92E-04	-16.11
**ILDR2**	56.20	1.08E-05	1.02E-04	-16.29
**LOC100131257**	284.56	6.09E-16	3.35E-14	-16.31
**SNORD12C**	134.89	5.69E-10	1.31E-08	-16.31
**ELK2AP**	69.08	2.91E-05	2.44E-04	-16.42
**ARHGEF28**	29.44	7.74E-03	2.86E-02	-16.49
**PKDREJ**	74.61	3.12E-03	1.36E-02	-16.83
**TMEM136**	38.39	2.13E-04	1.38E-03	-16.91
**FBN2**	30.40	2.62E-03	1.17E-02	-16.92
**PLA2G7**	89.34	7.60E-05	5.63E-04	-16.98
**TFAMP1**	447.06	5.61E-19	4.09E-17	-16.99
**TRIM6-TRIM34**	30.82	3.35E-03	1.44E-02	-17.13
**SNORD68**	127.23	2.98E-09	6.07E-08	-17.19
**TUBB3**	205.97	3.29E-07	4.49E-06	-17.22
**HLA-DPB2**	425.95	1.67E-15	8.74E-14	-17.31
**SNORD16**	38.99	1.16E-05	1.09E-04	-17.35
**KIR2DL3**	250.66	7.18E-04	3.91E-03	-17.38

**YY1P2**	38.69	5.35E-06	5.45E-05	-17.39
**SNORD2**	59.50	1.89E-06	2.16E-05	-17.45
**CD8A**	848.21	2.45E-05	2.10E-04	-17.46
**ST20-MTHFS**	31.55	3.44E-03	1.47E-02	-17.46
**SEPT7P9**	543.64	1.26E-31	2.09E-29	-17.52
**ZNF98**	186.36	5.59E-13	2.12E-11	-17.63
**LHFPL6**	93.33	2.80E-05	2.35E-04	-17.64
**VNN3**	31.60	3.62E-04	2.16E-03	-17.71
**S1PR3**	31.82	6.11E-03	2.35E-02	-17.75
**CFHR3**	31.76	3.00E-04	1.84E-03	-17.76
**PPM1H**	32.18	2.60E-03	1.16E-02	-17.93
**SLC9A7P1**	61.17	7.29E-06	7.20E-05	-17.95
**CRYBB2**	184.02	1.95E-11	5.89E-10	-17.97
**CNTNAP2**	32.15	1.19E-03	5.98E-03	-17.99
**EMP2**	32.48	9.25E-05	6.66E-04	-18.01
**MTRNR2L2**	6219.97	1.67E-56	9.14E-54	-18.02
**SNORD63B**	40.66	2.34E-05	2.03E-04	-18.05
**PRR4**	63.82	1.72E-03	8.20E-03	-18.19
**SLC24A2**	32.81	2.43E-05	2.09E-04	-18.24
**SNORD28**	111.35	2.27E-07	3.20E-06	-18.35
**TBC1D3P2**	32.73	4.39E-04	2.55E-03	-18.35
**CCDC144NL**	337.19	3.24E-10	7.88E-09	-18.63
**GOLGA8CP**	52.47	1.03E-05	9.75E-05	-18.68
**COL19A1**	53.06	4.15E-06	4.35E-05	-18.95
**SNORD116-6**	43.62	5.61E-05	4.32E-04	-19.30
**PRPS1L1**	135.92	7.08E-11	1.94E-09	-19.37
**SCN3A**	43.37	2.52E-05	2.15E-04	-19.47
**RAB6C**	2992.87	5.87E-66	4.11E-63	-19.58
**PRKCG**	34.95	1.80E-05	1.60E-04	-19.64
**MIR3654**	1798.84	3.28E-77	3.37E-74	-19.66
**LPAR4**	45.01	3.42E-05	2.79E-04	-20.03
**SNORD26**	379.06	2.00E-10	5.07E-09	-20.16
**MLC1**	45.09	9.24E-06	8.86E-05	-20.20
**CPE**	91.52	1.69E-04	1.13E-03	-20.20
**TCEAL6**	45.27	2.63E-06	2.90E-05	-20.22
**ZNF723**	144.88	3.21E-11	9.40E-10	-20.59
**RPL39L**	9016.94	1.66E-62	1.04E-59	-20.62
**SPDYE11**	37.05	1.75E-05	1.56E-04	-20.77
**ZNF732**	229.60	2.54E-16	1.49E-14	-21.00
**PDGFC**	46.81	3.05E-04	1.87E-03	-21.05
**SOX11**	38.25	9.70E-04	5.05E-03	-21.22
**LOC643387**	1587.39	2.19E-72	1.91E-69	-21.37
**GGTLC1**	39.09	2.43E-06	2.70E-05	-21.71
**TPTE**	38.91	6.52E-06	6.52E-05	-21.75
**PPIAP46**	91.29	3.33E-08	5.51E-07	-21.77
**SNORD99**	39.43	9.11E-05	6.57E-04	-21.86
**LOC654342**	604.95	3.56E-32	5.99E-30	-21.90
**CDC42P3**	651.12	6.92E-39	1.68E-36	-21.95
**CDHR1**	39.70	5.12E-05	3.99E-04	-21.99
**KCNJ2**	40.11	2.36E-05	2.04E-04	-22.14
**LINC02556**	40.46	2.48E-05	2.12E-04	-22.54
**SCGB3A1**	335.96	3.66E-03	1.55E-02	-22.68
**CES1P2**	41.77	1.41E-05	1.29E-04	-23.26
**SNORA16A**	246.92	4.29E-10	1.02E-08	-23.26
**HIST1H4K**	1222.49	2.33E-09	4.82E-08	-23.31
**DCAF8L2**	42.25	7.34E-05	5.47E-04	-23.67
**NBPF14**	98.80	1.40E-07	2.06E-06	-24.06

**CRYZL2P**	43.63	4.73E-04	2.72E-03	-24.37
**EN1**	44.09	6.50E-05	4.92E-04	-24.49
**MIR4721**	83.19	1.14E-08	2.12E-07	-24.55
**MOXD1**	44.02	2.38E-05	2.05E-04	-24.56
**SNORA54**	127.20	4.45E-07	5.91E-06	-24.69
**LOC645166**	1114.40	1.31E-48	4.45E-46	-24.75
**MTRNR2L7**	85.47	1.41E-07	2.07E-06	-25.11
**ANKRD62P1-PARP4P3**	56.20	6.56E-08	1.03E-06	-25.22
**FXYD6**	45.29	4.07E-06	4.28E-05	-25.27
**LINC00937**	85.95	1.25E-08	2.29E-07	-25.32
**LINC02190**	70.01	2.95E-08	4.92E-07	-25.58
**SNORD42A**	87.03	1.34E-04	9.21E-04	-25.62
**PABPC1P2**	449.29	1.23E-27	1.68E-25	-25.69
**PARD3**	71.50	1.37E-05	1.26E-04	-25.80
**MIR4444-1**	46.68	4.50E-07	5.98E-06	-25.87
**MIR4444-2**	46.68	4.50E-07	5.98E-06	-25.87
**CAMK2B**	47.27	3.24E-06	3.48E-05	-26.14
**CXCL14**	48.02	2.61E-04	1.64E-03	-26.61
**C16orf90**	1621.77	2.89E-56	1.53E-53	-26.75
**MIXL1**	60.44	3.79E-08	6.20E-07	-27.01
**AMY1B**	48.20	2.05E-07	2.92E-06	-27.16
**DSC2**	48.85	1.27E-04	8.78E-04	-27.32
**FABP5P3**	131.43	7.88E-10	1.77E-08	-27.43
**BMPR1B**	139.66	3.57E-09	7.24E-08	-27.67
**LINC01750**	50.93	1.09E-05	1.03E-04	-28.20
**KIR2DS4**	117.35	1.36E-07	2.01E-06	-28.67
**SCD5**	64.01	9.18E-08	1.40E-06	-28.85
**KCNJ3**	98.38	1.64E-08	2.91E-07	-29.13
**FLNC**	99.15	2.20E-10	5.53E-09	-29.30
**SNORD105B**	65.56	4.13E-04	2.42E-03	-29.51
**MIR922**	53.20	1.04E-07	1.57E-06	-29.73
**PAX5**	100.36	1.07E-07	1.61E-06	-29.82
**LOC101927284**	66.94	4.37E-06	4.55E-05	-29.90
**CR1L**	67.24	1.61E-07	2.33E-06	-30.51
**ANAPC1P1**	266.11	2.97E-12	1.00E-10	-30.76
**KLRC4-KLRK1**	210.23	4.48E-08	7.26E-07	-30.92
**LILRP2**	69.41	8.90E-09	1.67E-07	-31.06
**LOC105379511**	684.62	1.28E-35	2.54E-33	-31.29
**CD200R1L**	86.82	1.85E-09	3.89E-08	-31.68
**RGPD3**	1117.38	7.99E-53	3.41E-50	-31.73
**LOC100289656**	86.43	5.32E-09	1.04E-07	-31.75
**C9orf129**	58.14	7.27E-09	1.39E-07	-32.29
**AKR1C2**	112.37	1.45E-07	2.13E-06	-32.39
**LOC341056**	1190.32	2.96E-74	2.72E-71	-32.60
**THRB**	72.81	5.44E-07	7.07E-06	-32.71
**PPP1R2P2**	89.95	2.58E-10	6.46E-09	-32.83
**GABARAPL3**	74.07	4.73E-10	1.12E-08	-33.70
**EFNA5**	61.15	2.25E-07	3.17E-06	-33.84
**GGT3P**	76.10	3.95E-10	9.47E-09	-34.30
**LOC286059**	63.56	1.62E-08	2.88E-07	-35.70
**RPL10L**	703.69	6.55E-35	1.18E-32	-36.31
**SNORD79**	301.01	1.90E-10	4.86E-09	-36.40
**TUBA4B**	783.37	8.79E-44	2.61E-41	-36.61
**LOC101927314**	67.28	1.67E-08	2.96E-07	-37.27
**IQUB**	67.44	1.36E-10	3.56E-09	-37.63
**KLRC1**	250.94	1.67E-09	3.54E-08	-37.91
**SNORD87**	128.41	2.66E-10	6.61E-09	-38.11

**GPM6A**	69.15	2.98E-08	4.96E-07	-38.22
**RAB6C-AS1**	68.88	6.94E-10	1.57E-08	-38.40
**GLUD1P2**	85.59	8.40E-10	1.88E-08	-38.85
**PGK2**	109.97	2.23E-12	7.69E-11	-40.14
**CFHR1**	74.30	1.85E-08	3.24E-07	-41.46
**LOC101927429**	139.18	6.15E-13	2.29E-11	-41.91
**MIR6087**	3968.44	9.55E-80	1.19E-76	-41.96
**ZNF807**	632.34	5.83E-38	1.38E-35	-43.23
**TMSB4Y**	4217.12	7.56E-15	3.75E-13	-44.44
**CLEC4D**	81.60	1.02E-06	1.24E-05	-45.57
**LOC441601**	125.42	3.61E-13	1.41E-11	-46.01
**LOC100287072**	377.01	6.07E-23	5.99E-21	-48.14
**BAGE3**	87.21	2.87E-09	5.87E-08	-48.85
**BAGE2**	87.21	2.87E-09	5.87E-08	-48.85
**LINC02228**	109.84	5.48E-12	1.77E-10	-49.70
**TBC1D3B**	89.20	6.79E-09	1.30E-07	-49.94
**CLEC4E**	167.59	2.88E-10	7.10E-09	-50.32
**SNORA105C**	94.33	3.48E-13	1.36E-11	-52.48
**HMGN2P46**	466.44	2.54E-23	2.58E-21	-53.30
**ASNSP1**	119.86	2.24E-14	1.02E-12	-54.26
**HIST2H2BA**	99.67	2.25E-13	9.03E-12	-55.54
**LINC02088**	123.89	8.60E-14	3.71E-12	-56.00
**FAM35BP**	683.16	2.93E-30	4.58E-28	-57.90
**SNORA105A**	106.00	2.78E-14	1.24E-12	-59.11
**SNORA105B**	106.00	2.78E-14	1.24E-12	-59.11
**TSSC2**	106.86	2.17E-14	9.98E-13	-59.52
**SERF2-C15ORF63**	2638.93	1.37E-16	8.26E-15	-59.80
**TOP1P2**	109.17	2.50E-13	9.92E-12	-60.72
**BMS1P21**	363.86	1.13E-19	8.75E-18	-62.00
**FCN2**	139.64	2.03E-12	7.09E-11	-63.14
**CEP170P1**	116.04	3.41E-15	1.73E-13	-64.88
**EYA1**	118.09	1.54E-12	5.44E-11	-65.50
**TPI1P3**	1902.93	1.42E-77	1.60E-74	-67.65
**KLRC2**	121.39	9.46E-10	2.09E-08	-67.72
**OCM2**	131.46	2.91E-15	1.49E-13	-73.19
**FGF23**	164.29	1.30E-12	4.63E-11	-74.21
**ZASP**	138.00	3.91E-10	9.39E-09	-77.17
**PLGLA**	174.31	1.11E-17	7.28E-16	-79.18
**SPIN2A**	818.39	2.11E-36	4.51E-34	-84.62
**UBBP4**	18428.79	1.18E-254	2.06E-250	-94.85
**DBET**	608.93	7.29E-22	6.60E-20	-99.77
**LINC01002**	193.81	2.99E-17	1.92E-15	-108.48
**MEIS3P1**	1095.35	1.02E-34	1.82E-32	-115.37
**LOC100506675**	211.01	2.87E-16	1.65E-14	-117.39
**AMY1C**	215.89	3.65E-20	2.90E-18	-121.11
**KLF15**	301.03	2.85E-21	2.45E-19	-136.39
**MTRNR2L6**	15534.15	3.03E-166	1.32E-162	-151.98
**AMY1A**	271.65	1.05E-22	1.02E-20	-152.35
**SLC2A14**	2148.92	1.22E-50	4.64E-48	-160.84
**RPS4Y2**	443.81	1.01E-06	1.23E-05	-164.89
**ACTA1**	4497.16	4.67E-135	1.64E-131	-168.26
**BAGE4**	302.15	7.64E-24	8.05E-22	-168.67
**BAGE5**	308.04	4.23E-24	4.60E-22	-171.95
**HNRNPCL2**	377.90	4.20E-27	5.36E-25	-172.74
**GOLGA8DP**	450.37	2.36E-26	2.91E-24	-204.73
**CLDN11**	1135.48	1.05E-28	1.52E-26	-235.92
**HEPACAM**	670.09	3.71E-27	4.80E-25	-249.60

**H2BFS**	465.63	2.84E-27	3.76E-25	-259.07
**MIR1244-4**	1025.20	1.47E-35	2.76E-33	-312.83
**MIR1244-3**	1025.20	1.47E-35	2.76E-33	-312.83
**MIR1244-2**	1025.20	1.47E-35	2.76E-33	-312.83
**MIR1244-1**	1025.20	1.47E-35	2.76E-33	-312.83
**SNORD140**	5945.56	3.34E-88	5.85E-85	-521.47
**NCOR1P1**	1154.97	9.86E-44	2.88E-41	-644.21
**SNORA109**	3699.13	1.08E-51	4.18E-49	-644.68
**MIR1282**	1758.88	8.06E-46	2.52E-43	-980.74
**MTRNR2L10**	29679.44	1.87E-205	1.09E-201	-1106.81
