# Supplementary material for: Dysregulated lipid metabolism networks modulate T-cell function in people with relapsing-remitting multiple sclerosis
Source: Clin Exp Immunol. 2024 Apr 16;217(2):204–18. doi: 10.1093/cei/uxae032 (PMC11239565; doi:10.1093/cei/uxae032)
Supplement: uxae032_suppl_Supplementary_Materials [file uxae032_suppl_supplementary_materials.docx]

Supplementary Figures

Dysregulated Lipid Metabolism Networks Modulate T-cell Function in People with Relapsing Remitting Multiple Sclerosis

Lucia Martin-Gutierrez^1†,^ Kirsty E Waddington^1†^, Annalisa Maggio^1^, Leda Coelewij^1^, Alexandra Oppong^1^, Nina Yang^1^, Marsilio Adriani^1a^, Petra Nytrova^2^, Rachel Farrell^3^, Inés Pineda-Torra^4b#^ and Elizabeth C Jury^1#*^

†These authors contributed equally to this work and share first authorship.

# These authors share senior authorship.

*** Correspondence:** Professor Elizabeth Jury [e.jury@ucl.ac.uk](mailto:e.jury@ucl.ac.uk)

**Supplementary Table 1:** Demographic and clinical chararteristics of patient and healthy donor cohorts for each experiment**.** Cohort characteristics: Participants with relapsing remitting multiple sclerosis (RRMS) and healthy donors (HCs) between the ages of 18 and 66. All patients with RRMS were recruited before treatment with first disease modifying therapy. P-values are the result of an un-paired two-tailed t-test (~Mann Whitney) or chi-squared test (* Fishers test), as appropriate.

| **RNA-sequencing experiment** | **Healthy controls n=10** | **RRMS, n=10** | **P value** |
| --- | --- | --- | --- |
| **Age** years**, Mean (SD)** | 25.8 (5.3) | 41.2 (10.84) | 0.001 |
| **Sex, Male (%)/Female (%)** | 6 (60) / 4 (40) | 4 (40) / 6 (60) | 0.371 |
| **Ethnicity, White (%)** | 10 (100) | 10 (100) | 1 |
| **Smoking** |  |  | N/A |
| Current | 0 | 2 |  |
| Past | 0 | 2 |  |
| Never | 5 | 6 |  |
| Unknown | 5 | 0 |  |
| **BMI, Mean (SD)** | N/A | 28.2 (6.8) | - |
| **Years since diagnosis, Median (IQR)** | N/A | 0 (5) | - |
| **EDSS, Median (IQR)** | N/A | 1.75 (4.0) | - |
| **Relapses, Median (IQR)** | N/A | 1 (0) | - |
| **Vitamin D, Mean (SD)** | N/A | 13.10 (5.6) | - |
| **Assessing membrane lipids and in vitro experiments** | **Healthy controls N=13** | **RRMS, N=14** | **P value** |
| **Age** years**, Mean (SD)** | 31.77 (7.9) | 35.50 (10.5) | 0.311 |
| **Sex, Male (%)/Female (%)** | 4 (31) / 9 (69) | 3 (21.5) / 11 (78.5) | 0.58 |
| **Ethnicity** |  |  | 0.31 |
| **White (%)** | 11 (84.5) | 14 (100) |  |
| **Asian (%)** | 1 (<1) | 0 |  |
| **Mixed (%)** | 1 (<1) | 0 |  |
| **Smoking** |  |  | - |
| Current | 2 | 3 |  |
| Past | 0 | 6 |  |
| Never | 2 | 5 |  |
| Unknown | 10 | 0 |  |
| **BMI, Mean (SD)** | N/A | 24.62 (4.9) | - |
| **Years since diagnosis, Median (IQR)** | N/A | 1 (12) | - |
| **EDSS, Median (IQR)** | N/A | 2 (2) | - |
| **Relapses, Median (IQR)** | N/A | 1 (2) | - |
| **Vitamin D, Mean (SD)** | N/A | 27.29 (11.0) | - |
| **Metabolomics** | **Healthy controls** N=30 | RR**MS**, N=20 | **P value** |
| **Age years, Mean (SD)** | 36.0 (7.9) | 39.1 (10.5) | 0.352~ |
| **Ethnicity** |  |  |  |
| White (%) | 11 (84.5) | 20 (100) |  |
| Asian (%) | 1 (<1) | 0 | 0.312 |
| Mixed (%) | 1 (<1) | 0 |  |
| **Smoking** |  |  |  |
| Current | 2 | 4 | - |
| Past | 0 | 4 |  |
| Never | 10 | 12 |  |
| Unknown | 18 | 0 |  |
| **BMI, Mean (SD)** | N/A | 26 (5.81) | - |
| **Years since diagnosis, Median (IQR)** | N/A | 1 (14) | - |
| **EDSS, Median (IQR)** | N/A | 2 (4) | - |
| **Relapses, Median (IQR)** | N/A | 1 (2) | - |
| **Vitamin D, Mean (SD)** | N/A | 21.4 (12.77) | - |

Expanded disability status scale (EDSS) - used to quantify disability in MS patients from 0 (no disability) to 10 (death due to MS). Abbreviations: BMI – body mass index, SD - standard deviation, IQR – interquartile range, N/A – not available/applicable.

**Supplementary Figure 1: CD4+ T-cell gene expression in patients with RRMS is distinct from HCs.**

**
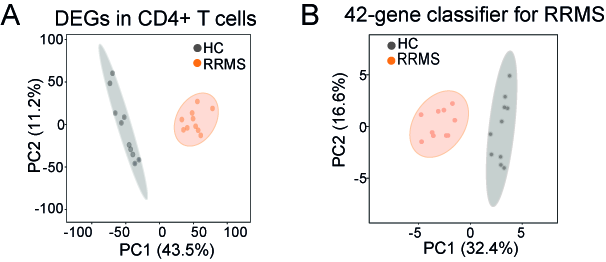
**

**Supplementary Figure 1: CD4+ T-cell gene expression in patients with RRMS is distinct from HCs.**

(**A**) FACS-sorted CD4+ T cells from healthy controls (HC, n=10) or RRMS patients (RRMS, n=10) were analysed by RNA-sequencing to assess differential gene expression. Principal component analysis (PCA) of differentially expressed genes (DEGs) clearly distinguishes HC and RRMS T cells.

**(B)** A 42-gene classifier developed to identify MS^3^ was applied to the DEG list from Figure 1A and Supplementary Data File 2. PCA analysis showing that HC and RRMS samples were correctly clustered.

**Supplementary Table 2.** Summary of the top ten most significantly up- and down- regulated genes in RRMS (n=10) compared to HCs (n=10). Genes were ranked according to FDR corrected p-values. Protein coding genes in bold.

| *Gene ID* | Description | Fold change | p-value |
| --- | --- | --- | --- |
| *RPS26* | ribosomal protein S26 | 115.65 | 1.02E-237 |
| *LOC100996740* | uncharacterized | 11.37 | 2.00E-113 |
| *RPL41* | ribosomal protein L41 | 19.16 | 8.48E-112 |
| *RPL21P28* | ribosomal protein L21 pseudogene 28 | 310.84 | 2.25E-103 |
| *EEF1A1* | eukaryotic translation elongation factor 1 alpha 1 | 4.96 | 1.78E-85 |
| *RPL13AP5* | ribosomal protein L13a pseudogene 5 | 7165.35 | 1.87E-80 |
| *LOC441081* | POM121 membrane glycoprotein (rat) pseudogene | 27.37 | 2.14E-77 |
| *IKBKG* | inhibitor of nuclear factor kappa B kinase regulatory subunit gamma | 6.92 | 7.43E-74 |
| *RPL21* | ribosomal protein L21 | 9.33 | 3.70E-65 |
| *POTEE* | POTE ankyrin domain family member E | 23.94 | 3.46E-63 |
| *UBBP4* | ubiquitin B pseudogene 4 | -94.85 | 2.06E-250 |
| *MTRNR2L10* | MT-RNR2 like 10 pseudogene | -1106.81 | 1.09E-201 |
| *MTRNR2L6* | MT-RNR2 like 6 pseudogene | -151.98 | 1.32E-162 |
| *ACTA1* | actin alpha 1, skeletal muscle | -168.26 | 1.64E-131 |
| *SNORD140* | small nucleolar RNA, C/D box 140 | -521.47 | 5.85E-85 |
| *HSP90B2P* | heat shock protein 90 beta family member 2, pseudogene | -3.56 | 1.48E-77 |
| *MIR6087* | microRNA 6087 | -41.96 | 1.19E-76 |
| *TPI1P3* | triosephosphate isomerase 1 pseudogene 3 | -67.95 | 1.60E-74 |
| *HLA-G* | major histocompatibility complex, class I, G | -11.19 | 1.60E-74 |
| *MIR3654* | microRNA 3654 | -19.66 | 3.37E-74 |

**
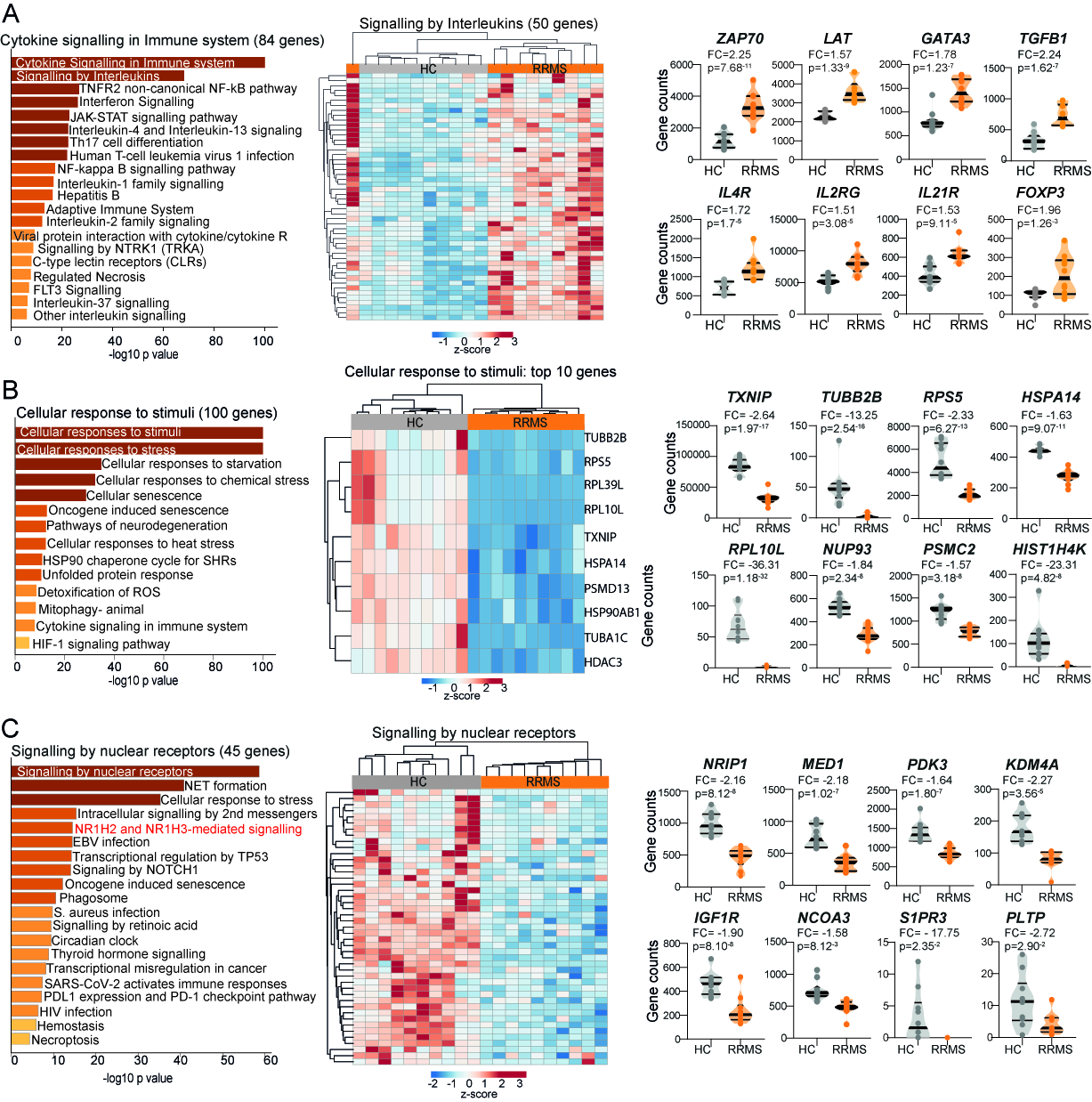
Supplementary Figure 2: Pathway analysis of up- and downregulated differentially expressed genes in CD4+ T cells from RRMS patients compared with HCs.**

**Supplementary Figure 2: Pathway analysis of up- and downregulated differentially expressed genes (DEGs) in CD4+ T cells from RRMS patients compared with HCs****.** FACS-sorted CD4+ T cells from healthy controls (HC, n=10) or RRMS patients (RRMS, n=10) were analysed by RNA-sequencing to assess DEGs. Pathway enrichment analysis of DEGs analysed by Metascape (1) to identify regulated pathways. (**A**) *Left panel*: Bar chart detailing ‘Cytokine Signalling in Immune System’ and member pathways (**from Figure 1C**). Member pathways ranked by p-value. *Middle panel:* Clustered heatmap of normalised gene counts in the ‘Signalling by Interleukins’ pathway. *Right panel*: Plots of normalised RNAseq gene counts of DEGs in the cytokine signalling pathway **see Figure 1D**. (**B**). *Left panel*: Bar chart detailing ‘Cellular Response to Stimuli’ and member pathways (**from Figure 1E**). Member pathways ranked by p-value. *Middle panel:* Clustered heatmap of top 10 normalised gene counts. *Right panel*: Plots of normalised RNAseq gene counts of top DEGs (**see Figure 1F**). **(C)** *Left panel*: Bar chart detailing ‘Signalling by Nuclear Receptors’ pathway and member pathways (**from Figure 1E**). DEGs are downregulated in RRMS patients compared to HCs. Member pathways ranked by p-value. *Middle panel:* Clustered heatmap of normalised gene counts. *Right panel*: Plots of normalised RNAseq gene counts of top DEGs.

**Supplementary Table 3: Transcription factor binding sequence enrichment.** Position weight matrix (PWM) transcription factor binding sequences enriched amongst genes downregulated in RRMS compared to HCs. Analysis conducted with Enrichr [https://amp.pharm.mssm.edu/Enrichr/] (2).

| PWM Term | Overlap | *P* | OR | Genes |
| --- | --- | --- | --- | --- |
| KRCTCNNNNMANAGC UNKNOWN | 11 / 66 | 0.07 | 1.62 | HIST1H2BN, HIST1H2AL, HIST1H2AK, HIST1H1D, HIST1H2BF, HIST1H1E, HIST1H2BE, HIST1H3I, HIST1H3B, HIST2H2AC, HIST1H1C |
| V$EVI1 06 | 5 / 23 | 0.08 | 2.11 | PDHA2, ARPC2, PCF11, MAP2, CREB5 |
| RYAAAKNNNNNNTTGW UNKNOWN | 11 / 87 | 0.28 | 1.23 | CTAGE1, GPM6A, SMG1, FLJ44635, ANXA2, GRK5, SWAP70, TBL1X, BZW2, HIPK1, CORO1C |
| V$LXR Q3 | **10 / 82** | **0.33** | **1.19** | **PRKCG, NFKBIA, MAFB, AMD1, HAS2, SPRY2, MITF, NRG1, JARID2, TMPO** |
| TTTNNANAGCYR UNKNOWN | 15 / 134 | 0.40 | 1.09 | HIST1H2BN, HIST1H2AL, NDRG3, HIST1H2AK, NAP1L5, DHRS3, HIST2H2AC, GRK5, HIST1H2BF, HIST1H1D, HIST1H2BE, HIST1H1E, PRKCQ, HIST1H3B, HIST1H1C |


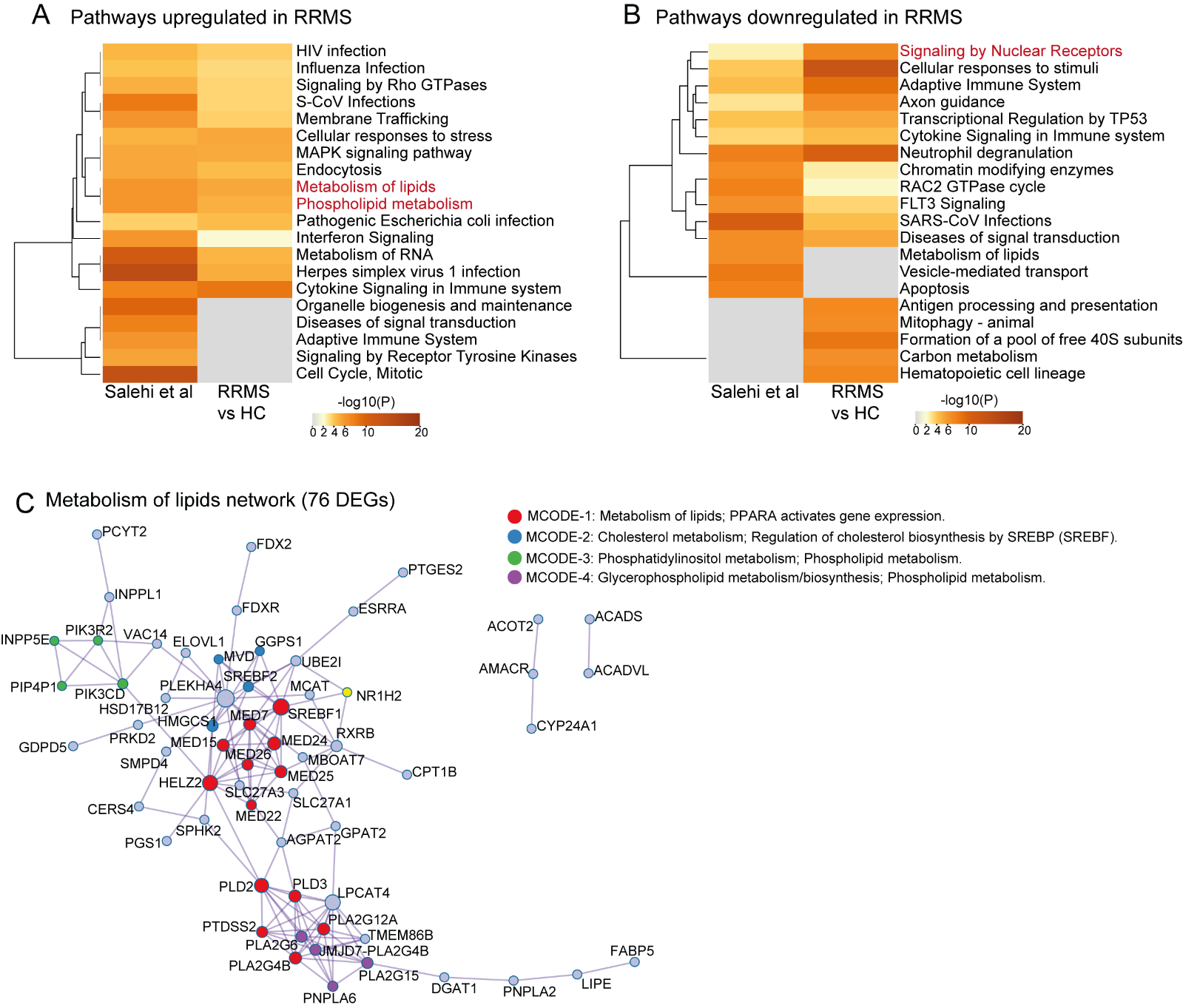
**Supplementary Figure 3: Common gene expression pathways validated across independent datasets**

**Supplementary Figure 3: Common gene expression pathways validated across independent datasets.** Multiple list enrichment (Metascape) was used to compare pathways enriched across two datasets. Heatmaps displaying -log10 p values of statistically significantly enriched genetic pathway ontology terms that overlap between CD4+ T-cell gene list analysis from RRMS patients at the relapse phase (3) (left)compared with RRMS patients vs HCs in this study (*right).* DEG lists using inclusion threshold (FC < -1.5 or >+1.5, p<0.05) were used. Upregulated **(A)** and downregulated **(B)** pathways are shown.

**(C)** Network diagram illustrates pathways associated with the genes significantly up and down regulated in the metabolism of lipids pathway (**Figure 1G**). Coloured nodes refer to significantly regulated pathways.

**Supplementary Table 4: Transcription factor enrichment within LXR-target gene list.** Position weight matrix (PWM) transcription factor binding sequences enriched amongst genes upregulated in RRMS compared to HCs. Analysis conducted with Enrichr^1^.

| PWM term | Overlap | *p* | OR | Genes |
| --- | --- | --- | --- | --- |
| ETS Q4 | 34 / 254 | 0.02 | 1.42 | RGS14, HM13, PDGFB, GATA3, ARHGAP4, DPP3, PPP1R9B, IKBKB, RAB43, ADAMTS4, SLC9A3R1, ESM1, RGS3, PDLIM2, AP1G2, FCHO1, HYAL2, JUNB, MAP4K1, MAP4K2, RPL41, SEMA4C, HMGA1, KCNAB2, DGKZ, PML, SIGIRR, CNKSR1, BIN3, LRFN4, TREML2, CD2BP2, RIN1, PLCB2 |
| V$ELK1 01 | 36 / 277 | 0.03 | 1.38 | SHC1, ARHGAP1, WAS, RASAL1, LTB4R, DPP3, CKS1B, RXRB, LYL1, ZNF408, CDC26, LZTS2, TBC1D17, VASP, MAP4K1, ESRRA, MAP4K2, TRAPPC1, C14ORF119, NR1H2, SH2D3C, SEMA4C, SYTL1, IRF2BP1, LDOC1, FOS, DGKZ, DOK1, TRAF7, ARHGEF4, TAGLN2, APBA3, CES2, SIPA1, LLGL2, BCL2L1 |
| V$P300 01 | 33 / 257 | 0.04 | 1.36 | RARG, RGS14, SHC1, ARHGAP1, PIK3CD, RASGRP2, DPP3, CKS1B, IKBKB, ZNF408, BAHD1, TRIM3, ENSA, JUNB, AAMP, WDTC1, MAP4K2, KCNH3, WNT10A, JUND, HMGCS1, C14ORF119, SEMA4C, RFX2, TFEB, LDOC1, KCNAB2, SREBF2, PML, TNFRSF1A, ADAM15, RARA, BCL2L1 |
| V$ETS1 B | 33 / 269 | 0.07 | 1.30 | RNF31, RGS14, HM13, PDGFB, DPP3, PPP1R9B, IKBKB, RAB43, ADAMTS4, SLC9A3R1, LYL1, ESM1, RGS3, PDLIM2, HYAL2, JUNB, DLGAP4, MAP4K2, LAG3, TFE3, SEMA4C, HMGA1, KCNAB2, RNF40, PML, CNKSR1, DOK1, BIN3, ADAM15, IMPDH1, KCNQ1, TREML2, PLCB2 |
| V$TEL2 Q6 | 30 / 242 | 0.07 | 1.32 | RGS14, SHC1, HM13, ARHGAP1, ARHGAP4, DPP3, CKS1B, IKBKB, RAB43, ADAMTS4, SLC9A3R1, RGS3, FCHO1, ZNF408, HYAL2, C1QTNF6, JUNB, ESRRA, MAP4K2, PPIL1, TRAPPC1, MCRS1, TFE3, SEMA4C, HMGA1, BIN3, CD2BP2, RIN1, SIPA1, MAP3K11 |
| V$MAZR 01 | 28 / 225 | 0.08 | 1.32 | KCNK6, RAB1B, SLC4A2, FXR2, PCBP4, SLC22A17, ENSA, MKNK2, PIM1, WNT1, JUNB, VASP, MBD3, KCNH2, MBD6, DMPK, TNFSF12, SEMA4C, HMGA1, RAB11B, CACNB1, FGF17, MOV10, AEBP2, GNB2, UST, ZNF513, RGL2 |
| V$SREBP1 02 | **12 / 89** | **0.13** | **1.43** | **CEBPB, OSBPL7, JUND, HYAL1, GNB2, RARA, PRKD2, PTMA, LOXL1, UNC13D, SREBF2, DLGAP4** |
| CRGAARNNNNCGA UNKNOWN | 07 / 48 | 0.16 | 1.55 | EEF1B2, RPS27, RABEP2, C14ORF119, PIK3R2, GATA3, UBE2M |
| V$AHRARNT 02 | 03 / 15 | 0.16 | 2.12 | EGR1, CBX6, AMPD2 |
| V$PEA3 Q6 | 30 / 264 | 0.16 | 1.21 | CD84, UNC93B1, RAB1B, INPPL1, GATA3, TCIRG1, SPATA6, IL18BP, DPP3, RPS6KA4, TRIM8, ADAMTS4, LZTS2, MARK2, C11ORF24, PSD, ESRRA, ELOVL1, MAP4K2, LAG3, SLC35C1, SYTL1, TYSND1, KCNAB2, DGKZ, PLCB3, LRFN4, KCNQ1, RIN1, SIPA1 |

**Supplementary Table 5: DEGs associated with cholesterol metabolism pathways.** All DEGs in the RRMS vs HC comparison were cross referenced with gene lists associated with cholesterol metabolism. R-HAS-191273: Cholesterol Biosynthesis; R-HAS-2426168: regulation of cholesterol biosynthesis by SREBP; R-HSA-6807047 cholesterol biosynthesis via desmosterol. Red-=upregulated; Blue=downregulated genes

| **Gene ID** | **Protein** | **RRMS vs HC** | | **Function** | **Previously identified role in neurological disease** | **Refs** |
| --- | --- | --- | --- | --- | --- | --- |
|  |  | **P value** | **FC** |  |  |  |
| MVD | mevalonate diphosphate decarboxylase | 1.82E-07 | 1.90 | Catalyzes the conversion of mevalonate pyrophosphate into isopentenyl pyrophosphate in one of the early steps in cholesterol biosynthesis. | Neurofibroma, Hernia of cerebellar tonsil into foramen magnum, Trigeminal neuralgia. | (4-7) |
| GGPS1 | geranylgeranyl diphosphate synthase 1 | 8.27E-05 | 1.52 | Catalyzes the synthesis of GGPP from farnesyl diphosphate and isopentenyl diphosphate. | Glioblastoma | (8, 9) |
| HELZ2 | helicase with zinc finger 2 | 1.84E-05 | 1.85 | Acts as a transcriptional coactivator for a number of nuclear receptors including PPARA, PPARG, THRA, THRB and RXRA. | Hyperlipidemia | (10) |
| SREBF1 | sterol regulatory element binding transcription factor 1 | 2.28E-05 | 2.23 | Binds the sterol regulatory element-1 (SRE1), a motif found in the low-density lipoprotein receptor gene promoter and other genes in sterol biosynthesis. | Neuroblastoma, Parkinson’s disease, Dementia | (11-15) |
| SREBF2 | sterol regulatory element binding transcription factor 2 | 1.24E-02 | 1.56 | Controls cholesterol homeostasis by regulating transcription of sterol-regulated genes. | Alzheimer’s disease, Vascular dementia | (16-20) |
| HMGCS1 | 3-hydroxy-3-methylglutaryl-CoA synthase 1 | 3.20E-02 | 1.50 | Catalyzes condensation of acetyl-CoA with acetoacetyl-CoA to form HMG-CoA, which is converted by HMG-CoA reductase to mevalonate, a cholesterol precursor | experimental autoimmune encephalomyelitis | (21, 22) |
| CREBBP | CREB binding protein | 1.55E-08 | -1.71 | Critical role in embryonic development, growth control, and homeostasis by coupling chromatin remodelling to transcription factor recognition. Intrinsic histone acetyltransferase activity and acts as a scaffold to stabilize protein interactions with the transcription complex. Acetylates histone and non-histone proteins. | Rubinstein-Taybi syndrome | (23) |
| MED1 | mediator complex subunit 1 | 1.02E-07 | -2.18 | Subunit of the CRSP (cofactor required for SP1 activation) complex. Component of other multi-subunit complexes e.g. thyroid hormone receptor-associated proteins which function on DNA templates in conjunction with initiation factors/cofactors. Regulates p53-dependent apoptosis; essential for adipogenesis. | Epilepsy, Parkinson’s disease | (24, 25) |
| NCOA6 | nuclear receptor coactivator 6 | 2.32E-07 | -1.69 | Transcriptional coactivator that interacts with nuclear hormone receptors to enhance transcriptional activator functions. Involved in hormone-dependent coactivation of several receptors, including prostanoid, retinoid, vitamin D3, thyroid hormone, steroid receptors. | Alzheimer’s disease | (26, 27) |
| FDPS | farnesyl diphosphate synthase | 1.00E-06 | -1.61 | Catalyzes production of geranyl pyrophosphate and farnesyl pyrophosphate from isopentenyl pyrophosphate and dimethylallyl pyrophosphate. Farnesyl pyrophosphate, is a key intermediate in cholesterol and sterol biosynthesis, a substrate for protein farnesylation and geranylgeranylation, and a ligand or agonist for hormone and growth receptors. | Neurodegenerative Disorder, Alzheimer’s disease | (28-30) |
| TBL1X | transducin beta like 1 X-linked | 1.05E-05 | -1.74 | Recruitment of ubiquitin/19S proteasome complex to nuclear receptor-regulated transcription units. Essential role in transcription activation by nuclear receptors. | Stroke | (31) |
| SP1 | Sp1 transcription factor | 5.57E-04 | -1.59 | Involved in many cellular processes, e.g. cell growth / differentiation, apoptosis, immune responses, response to DNA damage, and chromatin remodelling. | Multiple sclerosis; Parkinson’s disease; tauopathy; Epilepsy. | (32-35) |
| SEC24B | SEC24 homolog B, COPII coat complex component | 4.41E-03 | -1.50 | The protein encoded by this gene is a member of the SEC24 subfamily of the SEC23/SEC24 family, which is involved in vesicle trafficking. | Neural tube defects, Craniorachischisis | (36) |

**Supplementary Table 6: DEGs associated with Glycosphingolipid metabolism pathways.** All DEGs in the RRMS vs HC comparison were cross referenced with gene lists associated with glycosphingolipid metabolism. Glycosphingolipid metabolism pathways checked: R-HAS-1660662: Glycosphingolipid metabolism; Sphingolipid metabolism: has00600; Glycosphingolipid metabolic process: GO:0006687. Red-=upregulated; Blue=downregulated genes.

| **Gene ID** | **Protein** | **RRMS vs HC** | | **Function** | **Previously identified role in neurological disease** | **Refs** |
| --- | --- | --- | --- | --- | --- | --- |
|  |  | **P value** | **FC** |  |  |  |
| CPTP | ceramide-1-phosphate transfer protein | 8.86E-16 | 3.72 | Mediates intracellular transfer of ceramide-1-phosphate between organelle membranes and the cell membrane. |  |  |
| SPHK2 | sphingosine kinase 2 | 3.42E-09 | 2.44 | Catalyses phosphorylation of sphingosine into sphingosine 1-phosphate that mediates cellular processes including migration, proliferation, apoptosis. Can promote angiogenesis / tumorigenesis in cancer. | Neurodegenerative Disorder, Stroke, Ischemic stroke, Alzheimer’s disease | (37-41) |
| ELOVL1 | Elongation Of Very Long Chain Fatty Acids Protein 1 | 2.70E-07 | 1.81 | Catalyses the first and rate-limiting reaction in the long-chain fatty acids elongation cycle | Adrenoleukodystrophy- | (42, 43) |
| HTRA2 | HtrA serine peptidase 2 | 2.16E-06 | 1.56 | Promotes/induces cell death by direct binding to and inhibition of inhibitor of apoptosis proteins (IAPs), leading to increased caspase activity, or by IAP inhibition-independent, caspase-independent and serine protease activity-dependent mechanisms. | 3-Methylglutaconic Aciduria, Type Viii and Parkinson’s Disease, Alzheimer’s disease | (44-48) |
| ST6GALNAC6 | ST6 N-acetylgalactosaminide alpha-2,6-sialyltransferase 6 | 2.18E-05 | 2.09 | A sialyltransferase that modifies proteins and ceramides on the cell surface to alter cell-cell or cell-extracellular matrix interactions.Transfers the sialyl group (N-acetyl-alpha-neuraminyl or NeuAc) from CMP-NeuAc onto glycoproteins and glycolipids, forming an alpha-2,6-linkage. |  |  |
| CLN3 | CLN3 lysosomal/  endosomal transmembrane protein, battenin | 3.39E-05 | 1.55 | Mediates microtubule-dependent, anterograde transport connecting the Golgi network, endosomes, autophagosomes, lysosomes and plasma membrane, and participates in several cellular processes such as regulation of lysosomal pH, lysosome protein degradation, receptor-mediated endocytosis, autophagy, transport of proteins and lipids from the TGN, apoptosis and synaptic transmission. | Ceroid Lipofuscinosis Neuronal 3 and juvenile Neuronal Ceroid Lipofuscinosis. | (49) |
| PLA2G15 | phospholipase A2 group XV | 5.89E-05 | 2.04 | Hydrolyzes lysophosphatidylcholine to glycerophosphorylcholine and a free fatty acid. |  |  |
| ARSA | Arylsulfatase A | 8.79E-05 | 1.79 | Hydrolyses cerebroside sulphate to cerebroside and sulphate | metachromatic leucodystrophy Multiple sclerosis, Parkinson’s disease | (50-54) |
| PRKD2 | protein kinase D2 | 9.20E-05 | 1.78 | converts transient diacylglycerol signals into prolonged physiological effects downstream of PKC; involved in the regulation of cell proliferation via MAPK1/3 (ERK1/2) signaling, oxidative stress-induced NF-kappa-B activation, inhibition of HDAC7 transcriptional repression, signalling downstream of T-cell antigen receptor and cytokine production; plays a role in Golgi membrane trafficking, angiogenesis, secretory granule release and cell adhesion. |  |  |
| P2RX7 | purinergic receptor P2X 7 | 2.59E-04 | 1.82 | A ligand-gated ion channel responsible for ATP-dependent lysis of macrophages through the formation of membrane pores permeable to large molecules. Activation of this nuclear receptor by ATP in the cytoplasm may be a mechanism by which cellular activity can be coupled to changes in gene expression. | Multiple sclerosis | (55-58) |
| SMPD4 | sphingomyelin phosphodiesterase 4 | 3.17E-04 | 1.50 | Catalyses hydrolysis of membrane sphingomyelin to form phosphorylcholine and ceramide. | Neurodevelopmental disorder with microcephaly, arthrogryposis, and structural brain anomalies. | (59) |
| CERS4 | ceramide synthase 4 | 7.39E-03 | 2.01 | Catalyses formation of ceramide from sphinganine and acyl-CoA substrates, with high selectivity toward long and very-long chains (C18:0-C22:0) as acyl donor. |  |  |
| ASAH1 | N-acylsphingosine amidohydrolase 1 | 7.76E-08 | -1.64 | Catalyses the degradation of ceramide into sphingosine and free fatty acid. | Parkinson’s disease, Alzheimer’s disease | (60-63) |
| ESYT2 | Extended Synaptotagmin 2 | 1.89E-05 | -2.10 | Located in endoplasmic reticulum-plasma membrane contact site. Tethers endoplasmic reticulum to the cell membrane; involved in cellular lipid transport. |  |  |
| CERS6 | ceramide synthase 6 | 1.12E-04 | -1.84 | Degrades the bioactive signalling molecule sphingosine 1-phosphate. | experimental autoimmune encephalomyelitis | (64) |
| PSAP | Prosaposin | 1.65E-04 | -1.86 | Preproprotein- proteolytically cleaved to saposins A, B, C, and D. A myelinotrophic and neurotrophic factor | Gaucher disease (associated with saposin deficiency) and Parkinson’s disease | (65-68) |
| SMPDL3A | sphingomyelin phosphor-diesterase acid like 3A | 2.78E-04 | -11.59 | Enables phosphoric diester hydrolase activity and zinc ion binding activity. Involved in nucleoside triphosphate catabolic process. |  |  |
| PPT1 | palmitoyl-protein thioesterase 1 | 2.98E-04 | -1.58 | Involved in the catabolism of lipid-modified proteins during lysosomal degradation. | Alzheimer´s disease | (69) |
| GLTP | Glycolipid Transfer Protein | 3.57E-04 | -1.68 | Catalyzes transfer of glycosphingolipids between membranes. May be involved in the intracellular translocation of glucosylceramides. |  |  |
| ACER2 | alkaline ceramidase 2 | 7.65E-03 | -3.15 | Hydrolyzes very long chain ceramides to generate sphingosine. |  |  |
| SGMS1 | sphingomyelin synthase 1 | 1.14E-03 | -1.73 | Catalyses reversible transfer of phosphor-choline moiety in sphingomyelin biosynthesis: transfers phosphocholine head group of phosphatidylcholine (PC) to ceramide to form sphingomyelin and diacyl-glycerol (DAG). The reverse reaction transfers phosphocholine from sphingo-myelin to DAG to form PC and ceremide. | Lupus, Alzheimer disease | (24, 70, 71) |
| CREM | cAMP responsive element modulator | 7.94E-03 | -1.82 | Binds to the cAMP responsive element in viral and cellular promoters. Component of cAMP-mediated signal transduction during the spermatogenetic cycle, and other complex processes. | lupus, Transient Ischemic attack | (72, 73) |
| GLA | Galactosidase Alpha | 1.31E-02 | -1.56 | Catalyses the hydrolysis of glycosphingolipids and participates in their degradation in the lysosome. | Fabry disease, Hyper-trophic Cardiomyopathy.Multiple sclerosis, Parkinson’s disease | (74-77) |
| SGMS2 | sphingomyelin synthase 2 | 1.87E-02 | -4.94 | Sphingomyelin synthesis and homeostasis at plasma membrane. In sphingomyelin biosynthesis catalyses the transfer of phosphocholine head group of phosphatidylcholine (PC) to ceramide to form sphingomyelin and diacylglycerol. The reverse reaction transfers phosphocholine from sphingomyelin to diacylglycerol to form PC and ceremide. |  |  |
| KIT | KIT proto-oncogene, receptor tyrosine kinase | 2.42E-02 | -3.28 | Phosphorylates multiple intracellular proteins that play a role in in the proliferation, differentiation, migration and apoptosis of many cell types and thereby plays an important role in hematopoiesis, stem cell maintenance, gametogenesis, melanogenesis, and mast cell function. | Spinal muscular atrophy | (78) |
| SGPP2 | sphingosine-1-phosphate phosphatase 2 | 3.94E-02 | -1.69 | High phosphohydrolase activity against dihydrosphingosine-1-phosphate and sphingosine-1-phosphate in vitro. Sphingosine-1-phosphate phosphatase activity is needed for efficient recycling of sphingosine into the sphingolipid synthesis pathway. May attenuate intracellular sphingosine 1-phosphate signalling. |  |  |

**Supplementary Table 7: Correlations with clinical features.** Plasma membrane glycosphingolipid (top panel) and cholesterol (bottom panel) levels were correlated with clinical features of RRMS patients. Pearson’s correlation.

|  | **CTB vs. Age** | **CTB vs. EDSS** | **CTB vs. Duration** | **CTB vs. BMI** | **CTB vs. Relapses** | **CTB vs. vitD** |
| --- | --- | --- | --- | --- | --- | --- |
| r | -0.3209 | -0.177 | -0.1903 | -0.3705 | 0.08968 | 0.1617 |
| 95% CI | -0.7908 to  0.3869 | -0.7258 to 0.5094 | -0.7322 to 0.4991 | -0.8109 to 0.3380 | -0.5723 to 0.6809 | -0.5210 to 0.7182 |
| R squared | 0.103 | 0.03134 | 0.03622 | 0.1373 | 0.008042 | 0.02614 |
| P-value | 0.3659 | 0.6246 | 0.5984 | 0.292 | 0.8054 | 0.6554 |
|  |  |  |  |  |  |  |
|  | **Filipin vs. Age** | **Filipin vs. EDSS** | **Filipin vs. Duration** | **Filipin vs. BMI** | **Filipin vs. Relapses** | **Filipin vs. vitD** |
| r | 0.4158 | -0.04602 | -0.04793 | -0.05122 | 0.1794 | -0.3445 |
| 95% CI | -0.2897 to  0.8285 | -0.6566 to 0.6010 | -0.6577 to 0.5998 | -0.6596 to 0.5977 | -0.5076 to 0.7269 | -0.8005 to 0.3641 |
| R squared | 0.1729 | 0.002118 | 0.002297 | 0.002623 | 0.03218 | 0.1187 |
| P-value | 0.2321 | 0.8995 | 0.8954 | 0.8882 | 0.62 | 0.3297 |


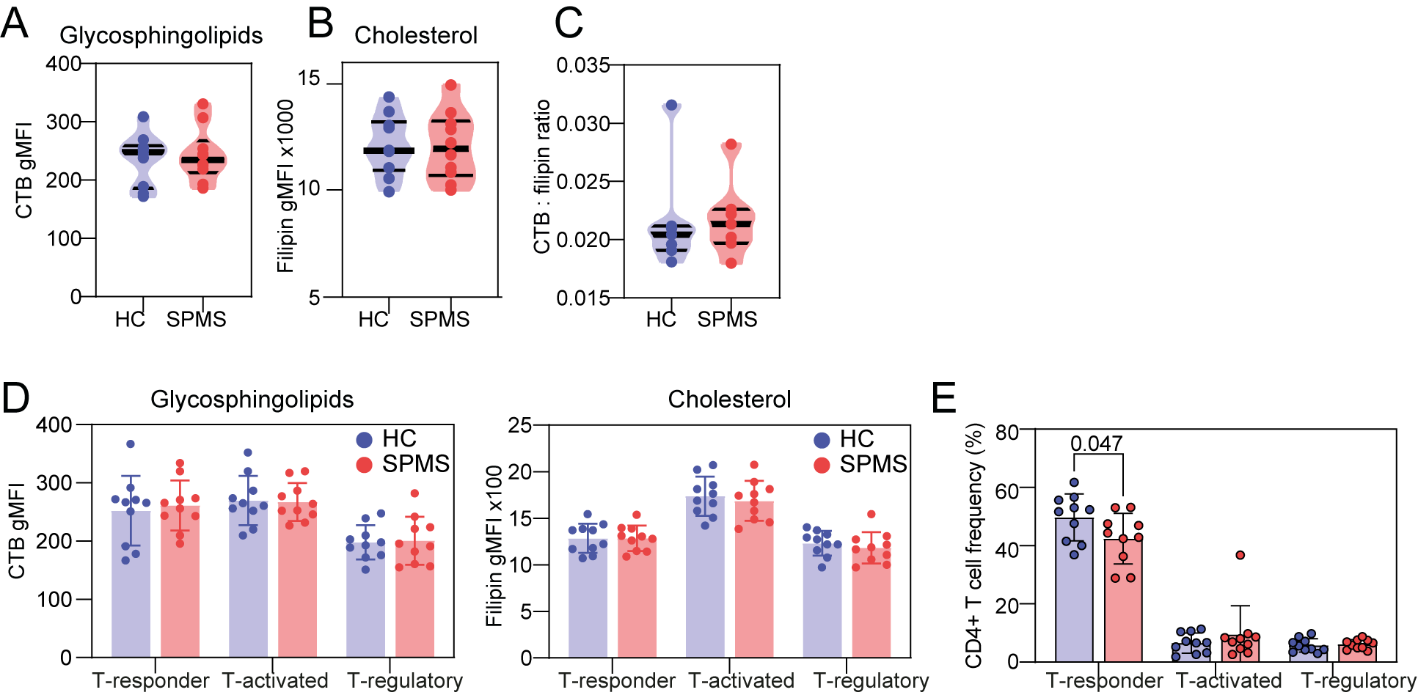
**Supplementary Figure 4: T cell plasma membrane lipid expression**

**Supplementary Figure 4: Plasma membrane lipid expression in patients with secondary progressive MS (SPMS).** CD4^+^ T cells from healthy controls (HC; n=10) (mean age 45.2; 50% female) or people with SPMS (n=10) (mean age 50.5; 60% female; mean disease duration 14.3 years, mean EDSS 6.7), were stained with cholera toxin B (CTB, glycosphingolipids) and filipin (cholesterol). Cumulative data shows CTB gMFI **(A)** and filipin gMFI **(B)**. **(C)** Violin plot showing the ratio of glycosphingolipid (CTB) to cholesterol (filipin) in HCs and SPMS patients. **(D)** Bar graphs showing expression of glycosphingolipid and cholesterol levels across T cell subsets: regulatory (T-reg, CD4+CD25+CD127−), responder (T-resp, CD4+CD25loCD127+) and activated (T-act, CD4+CD25+CD127+) in HCs vs SPMS patients **(E)** T cell subset frequency between HCs and SPMS patients. Mean +/-SE, two-tailed T test.

**Supplementary Table 8: Genes commonly regulated in T cells from RRMS patients vs HCs compared with TCR-activated HC T cells.** The fold changes of LXR-target genes differentially expressed in HC and RRMS T cells were compared to the fold changes between activated and resting T cells (TCR v no stim)(79) (**Figure 4A**). Genes commonly downregulated or upregulated are described.

| **Gene** | | **Name** | **Function** |
| --- | --- | --- | --- |
| **Upregulated** | SREBF1 | Sterol Regulatory Element Binding Transcription Factor 1 | Key transcription factor regulating expression of genes involved in cholesterol biosynthesis and lipid homeostasis. Precursor of transcription factor sterol regulatory element-binding protein 1, which is embedded in the endoplasmic reticulum membrane. Low sterol concentrations promote SREBF1 processing, releasing the transcription factor form that translocates into the nucleus and activates transcription of genes involved in cholesterol biosynthesis and lipid homeostasis. An important paralog of this gene is [SREBF2](https://www.genecards.org/cgi-bin/carddisp.pl?gene=SREBF2). |
|  | SREBF2 | Sterol Regulatory Element Binding Transcription Factor 2 | See above |
|  | HES6 | Hes Family BHLH Transcription Factor 6 | Member of a subfamily of basic helix-loop-helix transcription repressors. Members of this gene family regulate cell differentiation in numerous cell types. The protein encoded by this gene functions as a cofactor, interacting with other transcription factors. |
|  | TYMP | Thymidine Phosphorylase | Possible role in maintaining the integrity of blood vessels. Growth promoting activity on endothelial cells, angiogenic activity in vivo and chemotactic activity on endothelial cells in vitro. Catalyzes the reversible phosphorolysis of thymidine; involved nucleotide synthesis. |
| **Downregulated** | GAS7 | Growth Arrest Specific 7 | Expressed primarily in terminally differentiated brain cells and predominantly in mature cerebellar Purkinje neurons. Plays a putative role in neuronal development |
|  | HLA-DMB | Major Histocompatibility Complex, Class II, DM Beta | Peptide loading of MHC class II molecules, located in intracellular vesicles. Related pathways include TCR Signaling and CD28 co-stimulation. |
|  | A2M | Alpha-2-Macroglobulin | Protease inhibitor and cytokine transporter. |
|  | FGR | FGR Proto-Oncogene, Src Family Tyrosine Kinase | Non-receptor tyrosine-protein kinase; transmits signals from cell surface receptors and contributes to the regulation of immune responses, cyto-skeleton remodeling in response to extracellular stimuli, phagocytosis, cell adhesion and migration, |
|  | FBP1 | Fructose-Bisphosphatase 1 | Catalyzes hydrolysis of fructose 1,6-bisphosphate to fructose 6-phosphate, acts as a rate-limiting enzyme in gluconeogenesis. |
|  | MPEG1 | Macrophage Expressed 1 | Shows antibacterial activity against a wide spectrum of Gram-positive, Gram-negative and acid-fast bacteria |
|  | TGFB1 | Transforming Growth Factor Beta 1 | Precursor of the Latency-associated peptide (LAP) and TGF-beta-1 chains, which constitute the regulatory and active subunit of TGF-beta-1, respectively. regulates cell proliferation, differentiation, and growth, and can modulate expression and activation of other growth factors including interferon gamma and tumour necrosis factor alpha |
|  | MRAS | Muscle RAS Oncogene Homolog | Member of the Ras family of small GTPases. Membrane-associated protein that functions as signal transducer in multiple processes including cell growth and differentiation |
|  | PLA2G7 | Phospholipase A2 Group VII | Secreted enzyme that catalyses the degradation of platelet-activating factor to biologically inactive product TMEM121B |
|  | FAM20C | FAM20C Golgi Associated Secretory Pathway Kinase | Golgi serine/threonine protein kinase that phosphorylates secretory pathway proteins within Ser-x-Glu/pSer motifs. Constitutes the main protein kinase for extracellular proteins, generating the majority of the extracellular phosphoproteome |
|  | ALDH1A1 | Aldehyde Dehydrogenase 1 Family Member A1 | Enzyme in the major pathway of alcohol metabolism. Encodes the cytosolic isozyme in the liver. In mice has a role in retinol metabolism, this gene may also be involved in the regulation of the metabolic responses to high-fat diet |
|  | CD14 | CD14 Molecule | Preferentially expressed on monocytes/macrophages. Cooperates with other proteins to mediate the innate immune responses. Acts as a coreceptor for TLR2:TLR6 heterodimer in response to diacylated lipopeptides and for TLR2:TLR1 heterodimer in response to triacylated lipopeptides, these clusters trigger signaling from the cell surface and subsequently are targeted to the Golgi in a lipid-raft dependent pathway |

**Supplementary Figure 5: No differences in serum cytokine expression in HC vs RRMS patients**

**
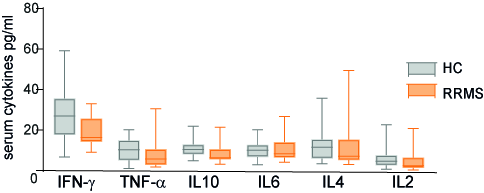
**

**Supplementary Figure 5: No differences in serum cytokine expression in HC vs RRMS patients.** Sera from HC (n=13) and RRMS (n=14) patients matched to patients included in this study were analysed for cytokine levels using the Human Th1/Th2 Cytokine bead Array kit (BD Bioscience, 560484) to measure expression of IFN-gamma, TNF, IL-6, IL-2, IL-4, IL-10 in 25 mL of undiluted serum. Sample were acquired on a BD FACSVerse. Values below the level of detection were removed. Box plots showing median and min/max points. Unpaired T tests.

**Supplementary Table 9: List of metabolites differentially expressed between HC and RRMS patients included in this study**

| Serum metabolites upregulated in RRMS patients | | | Serum metabolites downregulated in RRMS patients | | |
| --- | --- | --- | --- | --- | --- |
| **Metabolite name** | **p-value** | **Log_2_**  **Fold Change** | **Metabolite name** | **p-value** | **Log_2_**  **Fold Change** |
| **Low density lipoproteins (LDL)** | | | **Very low-density lipoproteins (VLDL)** | | |
| **LDL size** | 0.000025 | 0.006818 | **XL-VLDL-TG %** | 0.02271 | -0.21199 |
| **Very low-density lipoproteins (VLDL)** | | | **L-VLDL-TG %** | 0.031344 | -0.0993 |
| **XS-VLDL-PL %** | 0.019003 | 0.058479 | **VLDL size** | 0.038434 | -0.02941 |
| **M-VLDL-PL %** | 0.023216 | 0.079925 | **High density lipoproteins (HDL)** | | |
| **S-VLDL-CE %** | 0.028868 | 0.100272 | **XL-HDL-C %** | 0.01119 | -0.11848 |
| **M-VLDL-FC %** | 0.014212 | 0.130834 | **S-HDL-CE %** | 0.009498 | -0.06174 |
| **L-VLDL-C %** | 0.026028 | 0.150179 | **Other metabolites** | | |
| **L-VLDL-CE %** | 0.019062 | 0.220839 | **Omega-6/Omega-3** | 0.031316 | -0.47826 |
| **XL-VLDL-FC %** | 0.007134 | 0.234006 | **His** | 0.010209 | -0.18818 |
| **XS-VLDL-TG** | 0.032211 | 0.259876 | **Creatinine** | 0.024522 | -0.18431 |
| **XS-VLDL-P** | 0.013448 | 0.329906 | **Glucose** | 0.018029 | -0.1696 |
| **XS-VLDL-L** | 0.004211 | 0.38338 | **Albumin** | 0.048278 | -0.06545 |
| **XS-VLDL-FC** | 0.009902 | 0.396062 |  |  |  |
| **XS-VLDL-C** | 0.002203 | 0.401217 |  |  |  |
| **XS-VLDL-CE** | 0.002047 | 0.40351 |  |  |  |
| **XS-VLDL-PL** | 0.002753 | 0.438423 |  |  |  |
| **Intermediate density lipoproteins (IDL)** | | |  |  |  |
| **IDL-CE** | 0.041649 | 0.236276 |  |  |  |
| **IDL-C** | 0.046038 | 0.239221 |  |  |  |
| **IDL-L** | 0.02076 | 0.249836 |  |  |  |
| **IDL-PL** | 0.006743 | 0.269993 |  |  |  |
| **IDL-TG** | 0.010537 | 0.283667 |  |  |  |
| **High density lipoproteins (HDL)** | | |  |  |  |
| **L-HDL-P** | 0.046609 | 0.428539 |  |  |  |
| **XL-HDL-TG** | 0.048383 | 0.438203 |  |  |  |
| **L-HDL-FC** | 0.042721 | 0.466682 |  |  |  |
| **XL-HDL-PL** | 0.047203 | 0.554359 |  |  |  |
| **L-HDL-TG** | 0.001696 | 0.647471 |  |  |  |
| **Other metabolites** | | |  |  |  |
| **Unsaturation** | 0.038254 | 0.034928 |  |  |  |
| **Omega-3 %** | 0.025965 | 0.308684 |  |  |  |
| **Omega-3** | 0.015598 | 0.329578 |  |  |  |
| **Pyruvate** | 0.007289 | 0.450777 |  |  |  |

**Supplementary Figure 6. Summary of T cell dysregulated lipid metabolism pathways in pateints with RRMS compared to healthy controls**

**Supplementary Figure 6. Summary of T cell dysregulated lipid metabolism pathways in patients with RRMS compared to healthy controls**

Lipid metabolism pathways were dysregulated in CD4+ T-cells from patients with RRMS compared with HCs. *LXRB* was upregulated but LXR-signalling pathways were downregulated. Downregulated LXR-target genes included *IDOL* (which degrades the LDLR) potentially resulting in increased lipid influx. UGCG was also downregulated and was associated with reduced plasma membrane lipid raft glycosphingolipid levels. Genes controlling cholesterol biosynthesis were upregulated (*SREBF2, HMGCS1, MVD*) and associated with increased plasma membrane cholesterol levels. Genes associated with oxysterol biosynthesis (*CYP27A1*) and transport (*OSBPL10, OSBPL1A, OSBPL8, OSBPL9)* were also downregulated – supporting downregulation of the LXR-signalling pathway. Changes in lipid raft composition are known to disrupt CD4+ T-cell signalling and affect T-cell function (proliferation and cytokine production). T-cell activation and serum lipids could contribute to lipid metabolism disruption in CD4+ T-cells. Stimulation of LXR (via synthetic agonist GW3963) reduced plasma membrane cholesterol levels and restored T cell function in vitro.

**References**

1. Zhou Y, Zhou B, Pache L, Chang M, Khodabakhshi AH, Tanaseichuk O, et al. Metascape provides a biologist-oriented resource for the analysis of systems-level datasets. Nature Communications. 2019;10(1):1523.

2. Kuleshov MV, Jones MR, Rouillard AD, Fernandez NF, Duan Q, Wang Z, et al. Enrichr: a comprehensive gene set enrichment analysis web server 2016 update. Nucleic Acids Research. 2016;44(W1):W90-W7.

3. Salehi Z, Talebi S, Maleknia S, Palizban F, Naser Moghadasi A, Kavousi K, et al. RNA Sequencing of CD4(+) T Cells in Relapsing-Remitting Multiple Sclerosis Patients at Relapse: Deciphering the Involvement of Novel genes and Pathways. J Mol Neurosci. 2021;71(12):2628-45.

4. Aslan K, Gunbey HP, Tomak L, Ozmen Z, Incesu L. Magnetic Resonance Imaging of Intracranial Hypotension: Diagnostic Value of Combined Qualitative Signs and Quantitative Metrics. J Comput Assist Tomogr. 2018;42(1):92-9.

5. Satoh T, Yagi T, Onoda K, Kameda M, Sasaki T, Ichikawa T, et al. Hemodynamic features of offending vessels at neurovascular contact in patients with trigeminal neuralgia and hemifacial spasm. J Neurosurg. 2018:1-7.

6. Sun S, Jiang W, Wang J, Gao P, Zhang X, Jiao L, et al. Clinical analysis and surgical treatment of trigeminal neuralgia caused by vertebrobasilar dolichoectasia: A retrospective study. Int J Surg. 2017;41:183-9.

7. Wasa J, Nishida Y, Suzuki Y, Tsukushi S, Shido Y, Hosono K, et al. Differential expression of angiogenic factors in peripheral nerve sheath tumors. Clin Exp Metastasis. 2008;25(7):819-25.

8. Kim HY, Kim DK, Bae SH, Gwak H, Jeon JH, Kim JK, et al. Farnesyl diphosphate synthase is important for the maintenance of glioblastoma stemness. Exp Mol Med. 2018;50(10):1-12.

9. Abate M, Laezza C, Pisanti S, Torelli G, Seneca V, Catapano G, et al. Deregulated expression and activity of Farnesyl Diphosphate Synthase (FDPS) in Glioblastoma. Sci Rep. 2017;7(1):14123.

10. Yoshino S, Satoh T, Yamada M, Hashimoto K, Tomaru T, Katano-Toki A, et al. Protection against high-fat diet-induced obesity in Helz2-deficient male mice due to enhanced expression of hepatic leptin receptor. Endocrinology. 2014;155(9):3459-72.

11. Marwarha G, Claycombe-Larson K, Lund J, Ghribi O. Palmitate-Induced SREBP1 Expression and Activation Underlies the Increased BACE 1 Activity and Amyloid Beta Genesis. Mol Neurobiol. 2019;56(7):5256-69.

12. Lou F, Li M, Liu N, Li X, Ren Y, Luo X. The polymorphism of SREBF1 gene rs11868035 G/A is associated with susceptibility to Parkinson's disease in a Chinese population. Int J Neurosci. 2019;129(7):660-5.

13. Ivatt RM, Sanchez-Martinez A, Godena VK, Brown S, Ziviani E, Whitworth AJ. Genome-wide RNAi screen identifies the Parkinson disease GWAS risk locus SREBF1 as a regulator of mitophagy. Proc Natl Acad Sci U S A. 2014;111(23):8494-9.

14. Do CB, Tung JY, Dorfman E, Kiefer AK, Drabant EM, Francke U, et al. Web-based genome-wide association study identifies two novel loci and a substantial genetic component for Parkinson's disease. PLoS Genet. 2011;7(6):e1002141.

15. Reynolds CA, Hong MG, Eriksson UK, Blennow K, Wiklund F, Johansson B, et al. Analysis of lipid pathway genes indicates association of sequence variation near SREBF1/TOM1L2/ATPAF2 with dementia risk. Hum Mol Genet. 2010;19(10):2068-78.

16. Wang C, Zhao F, Shen K, Wang W, Siedlak SL, Lee HG, et al. The sterol regulatory element-binding protein 2 is dysregulated by tau alterations in Alzheimer disease. Brain Pathol. 2019;29(4):530-43.

17. de Dios C, Bartolessis I, Roca-Agujetas V, Barbero-Camps E, Mari M, Morales A, et al. Oxidative inactivation of amyloid beta-degrading proteases by cholesterol-enhanced mitochondrial stress. Redox Biol. 2019;26:101283.

18. Barbero-Camps E, Roca-Agujetas V, Bartolessis I, de Dios C, Fernández-Checa JC, Marí M, et al. Cholesterol impairs autophagy-mediated clearance of amyloid beta while promoting its secretion. Autophagy. 2018;14(7):1129-54.

19. Shah SA, Yoon GH, Chung SS, Abid MN, Kim TH, Lee HY, et al. Novel osmotin inhibits SREBP2 via the AdipoR1/AMPK/SIRT1 pathway to improve Alzheimer's disease neuropathological deficits. Mol Psychiatry. 2017;22(3):407-16.

20. Kim Y, Nam YJ, Lee C. Analysis of the SREBF2 gene as a genetic risk factor for vascular dementia. Am J Med Genet B Neuropsychiatr Genet. 2005;139b(1):19-22.

21. Voskuhl RR, Itoh N, Tassoni A, Matsukawa MA, Ren E, Tse V, et al. Gene expression in oligodendrocytes during remyelination reveals cholesterol homeostasis as a therapeutic target in multiple sclerosis. Proceedings of the National Academy of Sciences. 2019;116(20):10130-9.

22. Itoh N, Itoh Y, Tassoni A, Ren E, Kaito M, Ohno A, et al. Cell-specific and region-specific transcriptomics in the multiple sclerosis model: Focus on astrocytes. Proceedings of the National Academy of Sciences of the United States of America. 2018;115(2):E302-E9.

23. Bartsch O, Rasi S, Delicado A, Dyack S, Neumann LM, Seemanová E, et al. Evidence for a new contiguous gene syndrome, the chromosome 16p13.3 deletion syndrome alias severe Rubinstein-Taybi syndrome. Hum Genet. 2006;120(2):179-86.

24. Li JM, Wang XF, Xi ZQ, Gong Y, Liu FY, Sun JJ, et al. Decreased expression of thyroid receptor-associated protein 220 in temporal lobe tissue of patients with refractory epilepsy. Biochem Biophys Res Commun. 2006;348(4):1389-97.

25. Thenral ST, Vanisree AJ. Peripheral assessment of the genes AQP4, PBP and TH in patients with Parkinson's disease. Neurochem Res. 2012;37(3):512-5.

26. Pedersen NL, Posner SF, Gatz M. Multiple-threshold models for genetic influences on age of onset for Alzheimer disease: findings in Swedish twins. Am J Med Genet. 2001;105(8):724-8.

27. Schellenberg GD, Anderson L, O'Dahl S, Wisjman EM, Sadovnick AD, Ball MJ, et al. APP717, APP693, and PRIP gene mutations are rare in Alzheimer disease. Am J Hum Genet. 1991;49(3):511-7.

28. De Schutter JW, Park J, Leung CY, Gormley P, Lin YS, Hu Z, et al. Multistage screening reveals chameleon ligands of the human farnesyl pyrophosphate synthase: implications to drug discovery for neurodegenerative diseases. J Med Chem. 2014;57(13):5764-76.

29. Deane R, Singh I, Sagare AP, Bell RD, Ross NT, LaRue B, et al. A multimodal RAGE-specific inhibitor reduces amyloid β-mediated brain disorder in a mouse model of Alzheimer disease. J Clin Invest. 2012;122(4):1377-92.

30. Wollmer MA, Sleegers K, Ingelsson M, Zekanowski C, Brouwers N, Maruszak A, et al. Association study of cholesterol-related genes in Alzheimer's disease. Neurogenetics. 2007;8(3):179-88.

31. Ji LD, Hu SP, Li JY, Yao BB, Shen QJ, Xu J. Shared genetic etiology of hypertension and stroke: evidence from bioinformatics analysis of genome-wide association studies. J Hum Hypertens. 2017;32(1):34-9.

32. Menon R, Di Dario M, Cordiglieri C, Musio S, La Mantia L, Milanese C, et al. Gender-based blood transcriptomes and interactomes in multiple sclerosis: involvement of SP1 dependent gene transcription. J Autoimmun. 2012;38(2-3):J144-55.

33. Yao L, Dai X, Sun Y, Wang Y, Yang Q, Chen X, et al. Inhibition of transcription factor SP1 produces neuroprotective effects through decreasing MAO B activity in MPTP/MPP(+) Parkinson's disease models. J Neurosci Res. 2018;96(10):1663-76.

34. Santpere G, Nieto M, Puig B, Ferrer I. Abnormal Sp1 transcription factor expression in Alzheimer disease and tauopathies. Neurosci Lett. 2006;397(1-2):30-4.

35. Almeida AM, Murakami Y, Baker A, Maeda Y, Roberts IAG, Kinoshita T, et al. Targeted Therapy for Inherited GPI Deficiency. New England Journal of Medicine. 2007;356(16):1641-7.

36. Yang XY, Zhou XY, Wang QQ, Li H, Chen Y, Lei YP, et al. Mutations in the COPII vesicle component gene SEC24B are associated with human neural tube defects. Hum Mutat. 2013;34(8):1094-101.

37. Song DD, Zhou JH, Sheng R. Regulation and function of sphingosine kinase 2 in diseases. Histol Histopathol. 2018;33(5):433-45.

38. Wacker BK, Freie AB, Perfater JL, Gidday JM. Junctional protein regulation by sphingosine kinase 2 contributes to blood-brain barrier protection in hypoxic preconditioning-induced cerebral ischemic tolerance. J Cereb Blood Flow Metab. 2012;32(6):1014-23.

39. Song DD, Zhang TT, Chen JL, Xia YF, Qin ZH, Waeber C, et al. Sphingosine kinase 2 activates autophagy and protects neurons against ischemic injury through interaction with Bcl-2 via its putative BH3 domain. Cell Death Dis. 2017;8(7):e2912.

40. Lei M, Teo JD, Song H, McEwen HP, Yup Lee J, Couttas TA, et al. Sphingosine Kinase 2 Potentiates Amyloid Deposition but Protects against Hippocampal Volume Loss and Demyelination in a Mouse Model of Alzheimer's Disease. J Neurosci. 2019;39(48):9645-59.

41. Dominguez G, Maddelein ML, Pucelle M, Nicaise Y, Maurage CA, Duyckaerts C, et al. Neuronal sphingosine kinase 2 subcellular localization is altered in Alzheimer's disease brain. Acta Neuropathol Commun. 2018;6(1):25.

42. Schackmann MJ, Ofman R, Dijkstra IM, Wanders RJ, Kemp S. Enzymatic characterization of ELOVL1, a key enzyme in very long-chain fatty acid synthesis. Biochim Biophys Acta. 2015;1851(2):231-7.

43. Ofman R, Dijkstra IM, van Roermund CW, Burger N, Turkenburg M, van Cruchten A, et al. The role of ELOVL1 in very long-chain fatty acid homeostasis and X-linked adrenoleukodystrophy. EMBO Mol Med. 2010;2(3):90-7.

44. Lin CH, Chen PL, Tai CH, Lin HI, Chen CS, Chen ML, et al. A clinical and genetic study of early-onset and familial parkinsonism in taiwan: An integrated approach combining gene dosage analysis and next-generation sequencing. Mov Disord. 2019;34(4):506-15.

45. Fitzgerald JC, Zimprich A, Carvajal Berrio DA, Schindler KM, Maurer B, Schulte C, et al. Metformin reverses TRAP1 mutation-associated alterations in mitochondrial function in Parkinson's disease. Brain. 2017;140(9):2444-59.

46. Shao Y, Figeys D, Ning Z, Mailloux R, Chan HM. Methylmercury can induce Parkinson's-like neurotoxicity similar to 1-methyl-4- phenylpyridinium: a genomic and proteomic analysis on MN9D dopaminergic neuron cells. J Toxicol Sci. 2015;40(6):817-28.

47. Goo HG, Rhim H, Kang S. Pathogenic Role of Serine Protease HtrA2/Omi in Neurodegenerative Diseases. Curr Protein Pept Sci. 2017;18(7):746-57.

48. Westerlund M, Behbahani H, Gellhaar S, Forsell C, Belin AC, Anvret A, et al. Altered enzymatic activity and allele frequency of OMI/HTRA2 in Alzheimer's disease. FASEB J. 2011;25(4):1345-52.

49. Arntsen V, Strandheim J, Helland IB, Sand T, Brodtkorb E. Epileptological aspects of juvenile neuronal ceroid lipofuscinosis (CLN3 disease) through the lifespan. Epilepsy Behav. 2019;94:59-64.

50. Galla D, de Gemmis P, Anesi L, Berto S, Dolcetta D, Hladnik U. An Italian cohort study identifies four new pathologic mutations in the ARSA gene. J Mol Neurosci. 2013;50(2):284-90.

51. Cesani M, Lorioli L, Grossi S, Amico G, Fumagalli F, Spiga I, et al. Mutation Update of ARSA and PSAP Genes Causing Metachromatic Leukodystrophy. Hum Mutat. 2016;37(1):16-27.

52. Kappler J, Pötter W, Gieselmann V, Kiessling W, Friedl W, Propping P. Phenotypic consequences of low arylsulfatase A genotypes (ASAp/ASAp and ASA-/ASAp): does there exist an association with multiple sclerosis? Dev Neurosci. 1991;13(4-5):228-31.

53. Lee JS, Kanai K, Suzuki M, Kim WS, Yoo HS, Fu Y, et al. Arylsulfatase A, a genetic modifier of Parkinson's disease, is an α-synuclein chaperone. Brain. 2019;142(9):2845-59.

54. Pinel Ríos J, Madrid Navarro CJ, Pérez Navarro MJ, Cabello Tapia MJ, Piña Vera MJ, Campos Arillo V, et al. Association of Parkinson’s disease and treatment with aminosalicylates in inflammatory bowel disease: a cross-sectional study in a Spain drug dispensation records. BMJ Open. 2019;9(6):e025574.

55. Grygorowicz T, Strużyńska L. Early P2X7R-dependent activation of microglia during the asymptomatic phase of autoimmune encephalomyelitis. Inflammopharmacology. 2019;27(1):129-37.

56. Voo VTF, O'Brien T, Butzkueven H, Monif M. The role of vitamin D and P2X7R in multiple sclerosis. J Neuroimmunol. 2019;330:159-69.

57. Domercq M, Matute C. Targeting P2X4 and P2X7 receptors in multiple sclerosis. Curr Opin Pharmacol. 2019;47:119-25.

58. Zheng F, Zhou Q, Cao Y, Shi H, Wu H, Zhang B, et al. P2Y(12) deficiency in mouse impairs noradrenergic system in brain, and alters anxiety-like neurobehavior and memory. Genes Brain Behav. 2019;18(2):e12458.

59. Magini P, Smits DJ, Vandervore L, Schot R, Columbaro M, Kasteleijn E, et al. Loss of SMPD4 Causes a Developmental Disorder Characterized by Microcephaly and Congenital Arthrogryposis. Am J Hum Genet. 2019;105(4):689-705.

60. Kim MJ, Jeon S, Burbulla LF, Krainc D. Acid ceramidase inhibition ameliorates α-synuclein accumulation upon loss of GBA1 function. Hum Mol Genet. 2018;27(11):1972-88.

61. Taguchi YV, Liu J, Ruan J, Pacheco J, Zhang X, Abbasi J, et al. Glucosylsphingosine Promotes α-Synuclein Pathology in Mutant GBA-Associated Parkinson's Disease. J Neurosci. 2017;37(40):9617-31.

62. Robak LA, Jansen IE, van Rooij J, Uitterlinden AG, Kraaij R, Jankovic J, et al. Excessive burden of lysosomal storage disorder gene variants in Parkinson's disease. Brain. 2017;140(12):3191-203.

63. Torres S, García-Ruiz CM, Fernandez-Checa JC. Mitochondrial Cholesterol in Alzheimer's Disease and Niemann-Pick Type C Disease. Front Neurol. 2019;10:1168.

64. Eberle M, Ebel P, Mayer CA, Barthelmes J, Tafferner N, Ferreiros N, et al. Exacerbation of experimental autoimmune encephalomyelitis in ceramide synthase 6 knockout mice is associated with enhanced activation/migration of neutrophils. Immunol Cell Biol. 2015;93(9):825-36.

65. Tylki-Szymańska A, Czartoryska B, Vanier MT, Poorthuis BJ, Groener JA, Ługowska A, et al. Non-neuronopathic Gaucher disease due to saposin C deficiency. Clin Genet. 2007;72(6):538-42.

66. Schnabel D, Schröder M, Sandhoff K. Mutation in the sphingolipid activator protein 2 in a patient with a variant of Gaucher disease. FEBS Lett. 1991;284(1):57-9.

67. Ouled Amar Bencheikh B, Leveille E, Ruskey JA, Spiegelman D, Liong C, Fon EA, et al. Sequencing of the GBA coactivator, Saposin C, in Parkinson disease. Neurobiol Aging. 2018;72:187.e1-.e3.

68. Ambrosi G, Ghezzi C, Zangaglia R, Levandis G, Pacchetti C, Blandini F. Ambroxol-induced rescue of defective glucocerebrosidase is associated with increased LIMP-2 and saposin C levels in GBA1 mutant Parkinson's disease cells. Neurobiol Dis. 2015;82:235-42.

69. Young FB, Butland SL, Sanders SS, Sutton LM, Hayden MR. Putting proteins in their place: palmitoylation in Huntington disease and other neuropsychiatric diseases. Prog Neurobiol. 2012;97(2):220-38.

70. Wang C, Ming B, Wu X, Wu T, Cai S, Hu P, et al. Sphingomyelin synthase 1 enhances BCR signaling to promote lupus-like autoimmune response. EBioMedicine. 2019;45:578-87.

71. Lu MH, Ji WL, Xu DE, Yao PP, Zhao XY, Wang ZT, et al. Inhibition of sphingomyelin synthase 1 ameliorates alzheimer-like pathology in APP/PS1 transgenic mice through promoting lysosomal degradation of BACE1. Exp Neurol. 2019;311:67-79.

72. Koga T, Hedrich CM, Mizui M, Yoshida N, Otomo K, Lieberman LA, et al. CaMK4-dependent activation of AKT/mTOR and CREM-α underlies autoimmunity-associated Th17 imbalance. J Clin Invest. 2014;124(5):2234-45.

73. Raghavendra Rao VL, Bowen KK, Dhodda VK, Song G, Franklin JL, Gavva NR, et al. Gene expression analysis of spontaneously hypertensive rat cerebral cortex following transient focal cerebral ischemia. J Neurochem. 2002;83(5):1072-86.

74. Olivera S, Iñiguez C, García-Fernández L, Sierra JL, Camón AM, Menao S, et al. Usefulness of lyso-globotriaosylsphingosine in dried blood spots in the differential diagnosis between multiple sclerosis and Anderson-Fabry's disease. Mult Scler Relat Disord. 2020;38:101466.

75. Russo C, Riccio E, Pontillo G, Cocozza S, Tedeschi E, Centonze D, et al. Multiple sclerosis and fabry Disease, two sides of the coin? The case of an Italian family. Mult Scler Relat Disord. 2018;26:164-7.

76. Alcalay RN, Wolf P, Levy OA, Kang UJ, Waters C, Fahn S, et al. Alpha galactosidase A activity in Parkinson's disease. Neurobiol Dis. 2018;112:85-90.

77. Nelson MP, Boutin M, Tse TE, Lu H, Haley ED, Ouyang X, et al. The lysosomal enzyme alpha-Galactosidase A is deficient in Parkinson's disease brain in association with the pathologic accumulation of alpha-synuclein. Neurobiol Dis. 2018;110:68-81.

78. Wang S, Xing C, Wu H, Dai M, Zhao Y. Pancreatic schwannoma mimicking pancreatic cystadenoma: A case report and literature review of the imaging features. Medicine (Baltimore). 2019;98(24):e16095.

79. Waddington KE, Robinson GA, Rubio-Cuesta B, Chrifi-Alaoui E, Andreone S, Poon K-S, et al. LXR directly regulates glycosphingolipid synthesis and affects human CD4+ T cell function. Proceedings of the National Academy of Sciences. 2021;118(21):e2017394118.
